# Individualized treatment effects of a digital alcohol intervention and their associations with participant characteristics and engagement

**DOI:** 10.1093/alcalc/agae049

**Published:** 2024-07-21

**Authors:** Joel Crawford, Elizabeth S Collier, Marcus Bendtsen

**Affiliations:** Department of Health, Medicine and Caring Sciences, Linköping University, Sweden; Department of Health, Medicine and Caring Sciences, Linköping University, Sweden; Department of Health, Medicine and Caring Sciences, Linköping University, Sweden

**Keywords:** individualized treatment effects, randomized controlled trial, digital alcohol intervention, alcohol intervention, text-messaging based intervention

## Abstract

**Aims:**

Conditional average treatment effects are often reported in intervention studies, in which assumptions are made regarding how effects are similar across a heterogeneous sample. Nonetheless, differing factors, such as genetics, age, and sex, can impact an intervention’s effect on outcomes. The study aimed to estimate the individualized effects of a digital alcohol intervention among individuals looking online to reduce their drinking.

**Methods:**

We used data from a randomized controlled trial (RCT), including 2129 adults from the Swedish general population. The RCT concerned a text message-based alcohol intervention that sought to engender change through increasing knowledge on how to change and instilling confidence in changing behaviour. Outcomes were total weekly alcohol consumption and monthly heavy episodic drinking. Individualized treatment effects were modelled using baseline characteristics (age, gender, alcohol consumption, and psychosocial variables) and engagement with the intervention content.

**Results:**

We found evidence that the effects of the digital alcohol intervention were heterogeneous concerning participants’ age, baseline alcohol consumption, confidence, and importance. For heavy episodic drinking, there was evidence that effects were heterogeneous concerning age, sex, and baseline alcohol consumption. Overall, women, older individuals, and heavier drinkers benefitted more from the intervention in terms of effect size. In addition, participants who engaged more with the goal-setting and screening content reported better outcomes.

**Conclusions:**

The results highlight how different individuals respond differently to a digital alcohol intervention. This allows insight into who benefits the most and least from the intervention and highlights the potential merit of designing interventions adapted to different individuals’ needs.

## Teaser

**Short Summary:** Individualized treatment effects were discernible for older participants, women, and those who consumed higher quantities of alcohol and engaged with more heavy episodic drinking at baseline. Furthermore, those who deemed it of greater import to change behaviour and had more confidence in their ability to change benefited more from the intervention.

## Introduction

Seeking online for support to change lifestyle behaviours has increased over the last decade (Bendtsen et al. 2021). With improvements to communication networks and the near ubiquitous usage of mobile phones across the globe, the applicability of digital support is now more apparent than ever. One such means of support are digital alcohol interventions, which typically use mobile apps, text messages, or web pages to deliver support for health behaviour change (Bendtsen et al. 2021). Digital alcohol interventions have been shown to be effective in reducing alcohol consumption (Bewick et al. 2021) and their effects on the treatment of alcohol use disorders (AUDs) are comparable to those of face-to-face interventions (Johansson et al. 2021). This is particularly promising for treatment/prevention efforts because digital interventions are typically anonymous and have the potential to overcome the stigma and shame associated with seeking help for drinking behaviours (Riper et al. 2018).

In Sweden, despite several policy measures aimed at curbing alcohol consumption, such as high taxation and strict regulation on availability and advertising, the prevalence of alcohol consumption remains high: more than 30% of the adult population drinks at levels indicative of risky drinking (Ramstedt and Guttormsson 2024). While there is no safe alcohol consumption (Rehm et al. 2017), drinking at these levels increases the risk for experiencing health and social consequences (e.g. non-communicable disease, injuries, accidents) (Folkhälsomyndigheten 2024). Considering this, we developed and estimated the effects of a digital alcohol intervention for online help-seekers as a potential societal response to the high prevalence of risky drinking. The intervention was initiated via text messages, which contained hyperlinks to a personalized web-based application where participants were invited to record self-assessments and which also provided personalized and normative feedback on consumption patterns, along with interactive information on the associated risks of alcohol consumption. The intervention sought to engender change through increasing knowledge on how to change and instilling confidence in changing drinking behaviour, using techniques, such as behaviour substitution, goal setting, problem-solving, and reviewing of behavioural goals (Bendtsen and McCambridge 2019).

A randomized controlled trial (RCT) aimed to estimate the effectiveness of this intervention (Bendtsen et al. 2022). The trial found that those who were given access to the intervention reported consuming 23% less alcohol and engaging in 29% fewer heavy episodic drinking episodes (HED—4 (women)/5 (men) standard drinks per session, 1 standard glass contains 12 g of pure alcohol) over a 4-month period, compared to those who were given information on alcohol and health typically found online. Furthermore, a secondary mediation analysis found that effects on consumption and frequency of HED at 2- and 4-months post randomization were mediated by knowledge of how to change and confidence in being able to change (Bendtsen et al. 2023).

The results of the RCT are encouraging, and the mediation analysis provides a key insight into the underlying mechanisms that drive the intervention’s success. However, the effect estimates reported in the RCT are the conditional average treatment effects, i.e. an assumed similar effect across a heterogeneous sample. Nonetheless, differing participant characteristics, such as age, gender, comorbidities, and genetics can potentially impact the effect of an intervention on outcomes, and only part of the sample responds to the intervention in a manner that resembles the ‘average participant’ (Varadhan and Seeger 2013).

Extant literature demonstrates how individual differences impact treatment effects; across digital alcohol interventions, trials for adults in general have been shown to be more effective than trials for younger adults, while gender had no impact on outcomes (Kaner et al. 2018). Analyses assessing the individualized effects of single trials have shown these effects impact outcomes; Murphy *et al.* (2012) report that those with lower levels of substance-free reinforcement at baseline had fewer HEDs at 1-month follow-up, while Dennhardt *et al.* (2015) highlight that the effects of treatment conditions depended on the baseline reward value of alcohol. Furthermore, Kuhlemeier *et al.* (2021) report that baseline characteristics, such as demographics and psychosocial variables impacted the treatment effects of talking therapies on abstinence rates in patients with a diagnosed AUD.

The preceding literature highlights how individual differences impact treatment effects; nonetheless, the evidence regarding how these differences impact outcomes from digital intervention is limited. This study aims to address this issue by exploring the individualized treatment effects of the digital intervention tested by Bendtsen *et al.* (2022). To further understand the individualized effects of the intervention, the current study also aimed to assess if individual engagement with specific intervention components influenced the results, thereby enabling a greater understanding of whom this type of intervention may be most effective.

### Objectives

The primary objective of this study was to explore the individualized effects of a digital alcohol intervention through a contrast of outcome estimates in which comparisons between allocation to the intervention against control are made at the individual level. A secondary objective of this study was to explore the heterogeneity of the effects by examining associations between characteristics and microscopic engagement in the intervention content with the individualized effects.

## Methods

### Participants and settings

The target population was Swedish adults who were seeking online help to reduce alcohol consumption. The inclusion criteria were being 18 or older, having access to a mobile phone, and being classified as a risky drinker as defined by the Swedish National Board of Health and Welfare. This was defined as consuming 9 (women)/14 (men) or more standard drinks per week (total weekly consumption) or consuming 4 (women)/5 (men) or more standard drinks per drinking episode.

Ethical approval was granted on 6 November 2018 by the Regional Ethical Committee in Linköping, Sweden (Dnr 2018/417-31). Recruitment occurred between 25 April 2019 and 26 November 2020, during which 2129 participants were randomized. Participants were recruited using web-browser advertisements (Google, Bing, and Yahoo) and Facebook. Those interested in participating sent a text to a dedicated phone number. Within a few minutes, a response was sent with a hyperlink to a webpage containing informed consent. Those who consented were asked to complete a baseline survey, which was used to assess eligibility. Upon completion of the baseline survey, participants were randomized to receive immediate or delayed access to the intervention content.

### Interventions

Participants in the intervention condition were sent a text message with a link to the intervention and were granted immediate access to the content for 4 months. A text message was sent each Sunday reminding participants to access the web-based platform to record their consumption and complete the various modules (see Appendix A for a summary). For research purposes, access to the intervention was restricted to four months. Participants allocated to the control condition were sent a text message advising that they would receive information specifically designed to aid them in reducing their alcohol consumption, but also that after four months, they would get full access to the intervention. The information was accessed via a link to a national website that provides guidance on alcohol and health (https://www.iq.se). (For the protocol, see Bendtsen and McCambridge 2019).

### Outcomes and measures

Two primary outcomes were assessed in the trial:

(1) Total weekly alcohol consumption.
(2) Frequency of heavy episodic drinking (HED).

Total weekly consumption was measured using a short-term recall method, in which participants were asked to record how many standard drinks they consumed in the past week. Using a summary measure instead of a daily measure ensured the same item could be measured regardless of the collection mode. The frequency of HED was assessed by asking participants to record how many times they consumed 4 (women)/5 (men) or more standard drinks per session in the past month.

Outcomes from the trial were assessed at 2- and 4-months post-randomization. All follow-ups were initially completed via text messages, asking participants to click on a web link to the online questionnaires. For non-responders, two reminders were sent two days apart. If no response was received, a final text message was sent asking participants to reply to the text with responses for the two primary outcomes. Finally, if no response was received from the text messages, participants were called to collect responses, with a maximum of five calls.

The baseline characteristics assessed in the current study are comprised of demographics (gender and age), past drinking behaviour, and psychosocial factors believed to be important for behaviour change. Psychosocial factors were measured using three 10-point scales relating to how ‘important’ one believed making a change was, to what extent one had ‘knowledge’ of how to change (know-how), and how ‘confident’ one felt about making a change. Finally, we assessed the level of engagement in the intervention content. This refers to the specific modules that the participants accessed and engaged with on the web-based platform (see Appendix A).

### Randomisation and blinding

A fully computerized, simple randomization procedure was used; the neither researchers nor the participants had any knowledge of or the ability to manipulate the randomization sequence. Researchers were blinded to conditions prior to and after allocation; however, there was a risk of allocation being revealed when conducting telephone follow-ups with those who failed to respond to initial follow-up attempts. All study procedures were fully automated, and participants were informed about their allocation post-randomization.

### Statistical analyses

Following the approach outlined by (Hoogland et al. 2021), we aimed to model the observed outcome (Y) as a function of group allocation (G) and baseline variables (X) in order to estimate the individualized treatment effect $\delta \left({x}_i\right)=P\left({Y}_i|G=1,X={x}_i\right)-P\left({Y}_i=|G=0,X={x}_i\right)$. In other words, $\delta \left({x}_i\right)$ represents the difference between predicted alcohol consumption (${Y}_i$) for an individual ($i$) with some baseline characteristics ${x}_i$ if they had access to the intervention ($G=1$) versus if they did not ($G=0$). Having estimated ${\delta}_{\left({x}_i\right)}$ for each participant, we modelled these individualized treatment effects as a function of baseline covariates to study which individuals had the most and least benefit from the intervention. In a second analysis, we modelled the individualized treatment effects as a function of microscopic engagement data extracted from participants’ usage of the digital alcohol intervention, assessing various intervention components: weekly goal-setting, screening, reminders, normative comparison, timeline, tips for reducing consumption, and associated risk. This second analysis was among intervention group participants only.

Alcohol consumption outcomes at 2- and 4-months post-randomization were modelled using multilevel zero-inflated negative binomial regression with a time-by-group interaction term. We used available data to estimate the model and predict outcomes for all randomized participants. The models included participant-level adaptive intercepts, covariates for baseline characteristics, and covariate-by-time and group-by-covariate-by-time interactions. The baseline characteristics were sex, age, total weekly consumption, heavy episodic drinking, confidence, importance, and know-how. To promote a parsimonious model while at the same time not overly penalizing coefficients associated with non-interaction terms, we used priors that shrunk estimates towards the null for baseline characteristic interaction coefficients and Student’s *t* priors centred at 0 with 3 degrees of freedom and a scale of 2.5 for non-interaction coefficients (including the adaptive intercepts). For shrinkage, we used Cauchy priors centred at the null with a standard normal hyperprior for the scale parameter. We conducted sensitivity analyses using standard normal and Cauchy priors (location = 0, scale = 1) for non-interaction terms. Individualized treatment effects were calculated from posterior predictive draws, resulting in posterior distributions over treatment effects for each individual. The medians of these posterior predictive draws were used as point estimates of $\delta \left({x}_i\right)$, and we used the median absolute deviation (MAD) to illustrate the variation in the draws.

Linear regression was used to study associations between estimated individualized effects and baseline characteristics and microscopic engagement, respectively. We used Student’s *t* priors centred at 0 with 3 degrees of freedom and a scale of 2.5 for all coefficients in these models. We used the medians of posterior distributions of covariates as point estimates and created 95% compatibility intervals (CI) using the 2.5 and 97.5 percentiles of the posterior distributions.

All models were estimated using CmdStan 2.33.0, which is a shell interface for Stan (Stan Development Team 2023). The Stan code for estimating individualized effects can be found in Appendix B. Plots were generated using the ggplot2 library in R.

## Results

### Participants, retention, and missing data

In Table 1, baseline characteristics of the study population are presented. Outcome data were available from 1557 participants (73%) at the 2-month follow-up for total weekly alcohol consumption and from 1429 participants (67%) at the 4-month follow-up. For HED, outcome data were available from 1548 participants (73%) at the 2-month follow-up and from 1424 participants (67%) at the 4-month follow-up. These data were used to estimate the multilevel zero-inflated negative binomial regression models, which were subsequently used to estimate individualized treatment effects for all randomized participants (*n* = 2129). Summary statistics and trace plots from the Markov Chain Monte Carlo simulations indicated that chains had converged (see Appendix C).

**Table 1:** Baseline characteristics of participants.

	Total (*n* = 2129)	Intervention (*n* = 1063)	Control (*n* = 1066)
Total weekly alcohol consumption (standard drinks), median (quartiles)	17 (10; 25)	17 (10; 25)	16 (10; 25)
Frequency of heavy episodic drinking (per month), median (quartiles)	6 (4; 11)	6 (4; 10)	6 (4; 12)
Age, median years (quartiles)	45 (36; 54)	45 (35; 55)	46 (36; 54)
Sex, n (%)			
Women	1237 (58%)	612 (58%)	625 (59%)
Men	892 (42%)	451 (42%)	441 (41%)
Civil status, n (%)			
Living alone without kids at home	443 (21%)	219 (21%)	224 (21%)
Living alone with kids at home	215 (10%)	114 (11%)	101 (9%)
Living with somebody without kids	544 (26%)	267 (25%)	277 (26%)
Living with somebody with kids	756 (36%)	383 (36%)	373 (35%)
Have a partner but not living together	171 (8%)	80 (8%)	91 (9%)
Confidence, median score (quartiles)	6 (5; 8)	6 (5; 8)	6 (5; 8)
Importance, median score (quartiles)	10 (9; 10)	10 (9; 10)	10 (9; 10)
Know-how, median score (quartiles)	5 (2; 7)	5 (2; 7)	5 (2; 6)

### Individualized treatment effects and baseline characteristics

**Figure 1:**
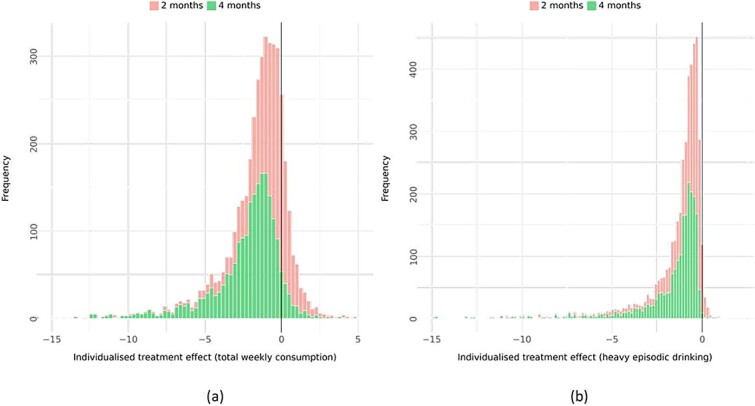
Distribution of individualized treatment effects on (a) total weekly consumption and (b) heavy episodic drinking.

In Fig. 1a, the distribution of effects for total weekly consumption is presented for both follow-up intervals at 2-month and 4-month post-randomization, and similarly for heavy episodic drinking in Fig. 1b. As can be seen, most participants were predicted to benefit from the intervention; however, more so after having access to the intervention for 4 months. The mean reduction of total weekly alcohol consumption at the 2-month interval was 0.6 standard drinks (SD = 1.4), while at the 4-month interval, the mean reduction was 1.7 standard drinks (SD = 2.2). Correspondingly, the mean reduction in heavy episodic drinking at the 2-month interval was 0.7 episodes (SD = 1.2), while at the 4-month interval, the mean reduction was 1.3 episodes (SD = 1.9). Sensitivity analyses using standard normal and Cauchy priors did not result in different findings (see Appendix D).

**Figure 2:**
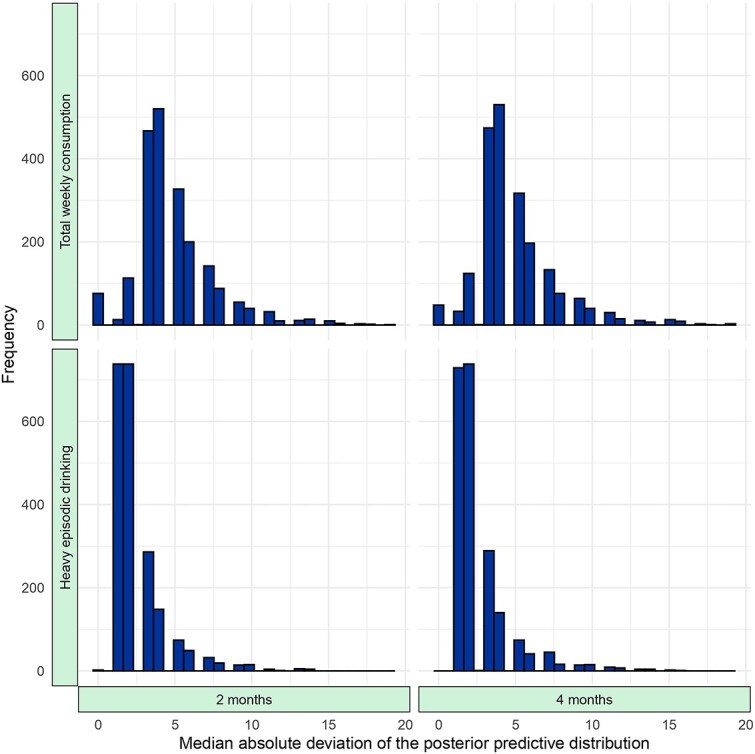
Distribution of median absolute deviation.

**Figure 3:**
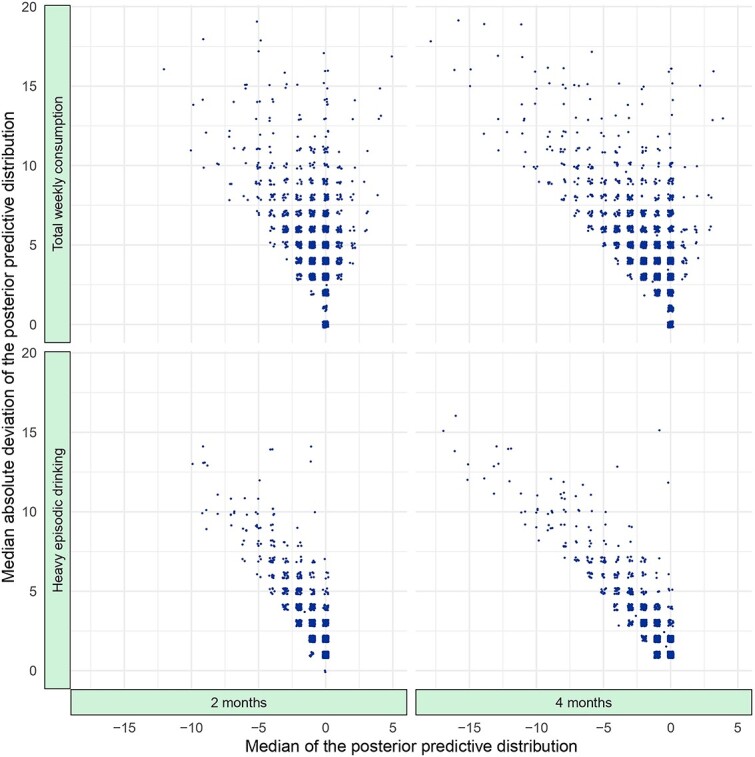
Relationship between median point estimates and median absolute deviation.

To illustrate the variability of the individualized treatment effects in Fig. 1, the MAD of posterior predictive draws was calculated for each outcome and time interval. Histograms of the MADs are shown in Fig. 2. For total weekly consumption, the mean MAD was 4.8 at both the 2- and 4-month intervals, while for heavy episodic drinking, the mean MAD was 2.4 at the 2-month interval and 2.5 at the 4-month interval. The statistical model that we used induces over-dispersion, which is evident in these MAD estimates, and the over-dispersion becomes more prominent as the estimated individualized effects increase in size. Thus, the right tails of the histograms show MADs where estimated individualized effects are larger. Fig. 3 further illustrates this, where MADs are plotted against the median of the posterior predictive distribution. Smaller estimated effects are coupled with higher certainty, and larger individualized effect sizes, while substantially fewer, are coupled with higher uncertainty.

In Table 2, associations between baseline characteristics and individualized treatment effects on total weekly alcohol consumption are presented. No strong evidence suggested an association between individualized effect and baseline sex or know-how. However, there was marked evidence for associations between individualized effect and baseline age, alcohol consumption, confidence, and importance (see Appendix E for figures).

**Table 2:** Associations between baseline characteristics and individualized treatment effects on total weekly alcohol consumption.

	**2 months**		**4 months**	
	**Median (95% CI)**	**Post. Prob. >/< null**	**Median (95% CI)**	**Post. Prob. >/< null**
**Man vs. woman**	0.07 (−0.02; 0.15)	94.3%	−0.02 (−0.15; 0.1)	64.1%
**Age**	−0.05 (−0.05; −0.04)	>99.9%	−0.06 (−0.07; −0.06)	>99.9%
**Total weekly consumption**	−0.03 (−0.03; −0.02)	>99.9%	−0.04 (−0.05; −0.04)	>99.9%
**Heavy episodic drinking**	−0.07 (−0.07; −0.06)	>99.9%	−0.14 (−0.15; −0.13)	>99.9%
**Confidence**	−0.17 (−0.18; −0.15)	>99.9%	−0.24 (−0.26; −0.22)	>99.9%
**Importance**	−0.07 (−0.1; −0.04)	>99.9%	−0.09 (−0.13; −0.05)	>99.9%
**Know-how**	0.01 (−0.01; 0.02)	86.8%	−0.01 (−0.03; 0.01)	78.6%

In Table 3, associations between baseline characteristics and individualized treatment effects on heavy episodic drinking are presented. There was no strong evidence for an association between individualized effect and baseline confidence, importance, or know-how. However, there was marked evidence for associations between individualized effects and baseline sex, age, and alcohol consumption. Women were more likely to benefit from the intervention than men, as were older and more heavy drinkers (see Appendix F for figures).

**Table 3:** Associations between baseline characteristics and individualized treatment effects on heavy episodic drinking.

	**2 months**		**4 months**	
	**Median (95% CI)**	**Post. Prob. >/< null**	**Median (95% CI)**	**Post. Prob. >/< null**
**Man vs. Woman**	0.08 (0.0; 0.16)	97.6%	0.14 (0.02; 0.26)	99.0%
**Age**	−0.02 (−0.02; −0.02)	>99.9%	−0.02 (−0.03; −0.02)	>99.9%
**Total weekly consumption**	−0.02 (−0.02; −0.01)	>99.9%	−0.03 (−0.04; −0.02)	>99.9%
**Heavy episodic drinking**	−0.09 (−0.09; −0.08)	>99.9%	−0.15 (−0.16; −0.14)	>99.9%
**Confidence**	0.01 (−0.01; 0.02)	89.5%	0.01 (−0.02; 0.03)	72.8%
**Importance**	0.02 (−0.01; 0.05)	94.3%	0.0 (−0.04; 0.04)	57.5%
**Know-how**	0.0 (−0.02; 0.01)	67.8%	−0.01 (−0.03; 0.01)	88.4%

### Individualized treatment effects and engagement

Table 4 reports the associations between participant engagement and individualized treatment effects on total weekly alcohol consumption. There was no evidence of marked associations between individualized effects and reminders, normative information, timelines, or tips. However, there was evidence for associations between individualized effects and goal-setting and screening, with more engagement in these being associated with greater effects. On the other hand, there was a reverse association with risk information (see Appendix G for figures).

**Table 4:** Associations between participant engagement and individualized treatment effects on total weekly consumption.

	**2 months**		**4 months**	
	**Median (95% CI)**	**Post. Prob. >/< null**	**Median (95% CI)**	**Post. Prob. >/< null**
**Total weekly drinking goal set**	−0.2 (−0.32; −0.08)	>99.9%	-0.32 (−0.51; −0.14)	99.9%
**Weekly screening**	−0.02 (−0.04; 0.0)	96.2%	−0.02 (−0.05; 0.01)	84.5%
**Reminder set**	0.01 (−0.02; 0.05)	72.0%	0.01 (−0.05; 0.07)	63.8%
**Visited normative comparison**	0.01 (−0.04; 0.06)	61.5%	0.03 (−0.04; 0.11)	80.7%
**Visited timeline**	0.0 (−0.04; .03)	53.1%	−0.01 (−0.07; 0.04)	68.9%
**Visited tips**	−0.06 (−0.14; 0.02)	94.1%	−0.06 (−0.18; 0.06)	82.5%
**Visited risks**	0.12 (0.05; 0.18)	>99.9%	0.14 (0.03; 0.24)	99.6%

Table 5 reports the association between participant engagement and individualized treatment effects on HED. There was no evidence of associations between individualized effects and goal-setting, reminders, normative information, timelines, or tips. However, there was marked evidence for associations between individualized effect and screening and risk information, indicating that more engagement with these components was associated with worse outcomes (see Appendix H for figures).

**Table 5:** Associations between participant engagement and individualized treatment effects on heavy episodic drinking.

	**2 months**		**4 months**	
	**Median (95% CI)**	**Post. Prob. >/< null**	**Median (95% CI)**	**Post. Prob. >/< null**
**Heavy episodic drinking goal set**	−0.05 (−0.15; 0.05)	83.6%	−0.03 (−0.19; 0.12)	65.8%
**Weekly screening**	0.02 (0.0; 0.04)	99.4%	0.04 (0.01; 0.06)	99.8%
**Reminder set**	−0.01 (−0.04; 0.02)	72.8%	−0.01 (−0.06; 0.03)	72..0%
**Visited normative comparison**	0.03 (−0.01; 0.07)	90.8%	0.05 (−0.01; 0.12)	94.8%
**Visited timeline**	−0.02 (−0.05; 0.01)	90.9%	−0.04 (−0.08; 0.01)	94.8%
**Visited tips**	−0.03 (−0.09; 0.04)	79.3%	−0.02 (−0.12; 0.08)	63.4%
**Visited risks**	0.06 (0.0; 0.11)	98.3%	0.06 (−0.02; 0.15)	93.3%

## Discussion

In assessing the individualized treatment effects of the digital alcohol intervention, we found that effects were more pronounced for those who reported consuming more alcohol and engaging with more HED at baseline, which may suggest the intervention is more effective for heavier drinkers. This result contrasts with findings from Collier *et al.* (2024), who assessed satisfaction with the intervention re-analysed here and highlighted that those most satisfied with the support tool were those who drank less at baseline. Furthermore, these participants also suggested that the type of support offered might be less helpful for heavier drinkers. The disparity could be a result of a temporal difference in experiencing benefits from the support; those who were lighter drinkers may have experienced benefits much sooner than heavier drinkers, as it may have taken less time and/or effort to change their drinking behaviour. We must also consider that those drinking at higher rates have more potential for change regarding their consumption than those reporting lower, albeit still hazardous, consumption patterns.

Furthermore, effects were more discernible for older than younger participants. This result is in line with extant literature highlighting that digital alcohol interventions are more effective for adults in general compared to trials focusing specifically on young adults (Kaner et al. 2018) and evidence highlighting that talking therapies are more effective for the treatment of AUDs for older than younger adults (Kuhlemeier et al. 2021). Our findings may be reflective of the difference in various life stressors between older and younger adults; e.g. older adults, by and large, will have more responsibility and commitments (e.g. children and mortgages), while younger adults may face more social pressures to consume alcohol (Conroy and Measham, 2019) and often report social motives for drinking (Kuntsche et al. 2005; Cooper et al. 2016). In contrast, older adults are motivated to consume alcohol for other purposes, often drinking for affective reasons, such as enhancement and coping (van Gils et al. 2020). It may be that the techniques used in the intervention were better suited to the needs of older adults in that planning and problem-solving techniques may be more applicable for those who drink for affective reasons than those who drink for social reasons. For example, social pressures to drink alcohol in group settings may override personal plans for reducing consumption as young adults want to avoid alienation (Conroy and de Visser 2014). The findings suggest that intervention efforts for younger adults may benefit from a different approach, such as focusing on their personal dimensions of behaviour (e.g. underlying motives for drinking). Nonetheless, this again contrasts with Collier *et al.* (2024), who highlight that older participants reported less satisfaction with the intervention support as they perceived the content was better suited to social drinkers and not those who are at risk of developing an AUD.

The results for the other factors are mixed; those higher in confidence and those who deemed it of greater importance to reduce their consumption at baseline benefited more in terms of reducing weekly consumption. This finding supports previous literature highlighting that these factors are associated with consuming less alcohol and greater abstinence (Bertholet et al. 2012, Müller et al. 2019, Meredith et al. 2021). In contrast, there was no association of either factor with HED. For importance, this disparity may be a result of the content of the intervention, which focused more on the chronic health consequences of drinking and less on the acute consequences typically associated with engagement in HED (Jones et al. 2020). Furthermore, confidence in ability to reduce consumption was instilled through techniques that focused on planned behaviour, such as goal setting, action planning, and behaviour substitution. These techniques may be more appropriate for reducing total consumption over a greater timeframe in which behaviour can be planned. Still, acute drinking episodes (i.e. HED) are often unplanned and occur as a result of the pharmacological and disinhibiting effects of alcohol, which leads to individuals consuming more than planned (George et al. 2005). This suggests this type of content may be beneficial in reducing total alcohol consumption for those who are confident in their ability to reduce drinking and who deem it important. Nonetheless, further research is needed to ascertain which socio-cognitive factors predispose individuals to greater success in reducing HED.

In terms of total weekly consumption, the intervention was equally effective for men and women. This is consistent with the results of Kaner *et al.* (Kaner et al. 2018), who found no impact of gender on the effectiveness of digital alcohol interventions. However, we found contrasting results for HED, with women benefiting more than men from the intervention for reducing their HED. This result is consistent with Kuhlemeier *et al.* (2021), who highlight that women benefit more than men from talking therapies for abstinence. A key component of the intervention was self-authored prompts that aimed to promote change. Self-authoring or writing plays a prominent role in the therapeutic process of various psychotherapies (Ruini and Mortara 2022), plus women were more likely to engage with the self-authored prompt module than men in the RCT (Collier et al. 2024). This suggests that for reducing HED, this type of intervention may be more beneficial for women, and that a different approach for men is needed, e.g. focusing on factors, such as men’s beliefs or expectancies regarding HED (Cooper et al. 1992). For know-how, there was no impact on either total weekly consumption or HED. However, the previously published mediation analysis identified know-how as a significant mediating factor for weekly consumption and HED (Bendtsen et al. 2023). This suggests that participants who perceived they had high levels of know-how for reducing alcohol consumption at baseline had, in fact, limited knowledge, and by participating in the intervention, they gained knowledge that could be used effectively in everyday life.

The results for engagement in the intervention content are mixed. For total weekly consumption, the effects were more discernible for those who engaged more with the screening and weekly goal-setting modules. This finding is consistent with evidence highlighting that planning behaviour techniques, such as goal-setting are effective components of a text-messaging-based intervention (Suffoletto et al. 2023). For both total consumption and HED, individualized effects were found for those who accessed the associated risk module less and for those who engaged less with the screening module. While this could potentially suggest that greater screening of behaviour and viewing of the associated risks resulted in greater alcohol consumption as a coping mechanism, this finding is more likely a result of a few extreme data points driving the association (see Fig. 3 in Appendix H). No effects on engagement in the other modules were found. This suggests the intervention may be more beneficial for conscientious people, a trait associated with being planful, task, and goal-directed (Leary and Hoyle 2009). These participants engaged in more planning behaviour, for which evidence suggests the order and self-discipline aspects of conscientiousness are associated with a reduced risk of heavy alcohol consumption (Luchetti et al. 2018); in addition, the trait is also linked to planning/action behaviour for health protection (Weston and Jackson 2016). It may be the case that engagement in this content benefited those higher in conscientiousness, as it provided tools and information for change that complement this trait. This suggests that a different approach may be warranted for those less conscientious (e.g. mindfulness), while future alcohol interventions should consider assessing personality factors as moderators.

### Limitations

While the results of the analysis provide an insight into the heterogeneous effects of the intervention, they should be interpreted with some consideration. All RCTs test the randomized component of a trial rather than comparing one treatment with another, meaning that individuals may have different levels of adherence and engagement in an intervention; hence, these results should be interpreted in terms of accessing the intervention rather than using it. Another limitation that should be borne in mind when interpreting findings is that a statistical model was used to predict and contrast outcomes, which means that the individualized effects are not based entirely on observed data but on predicted data. There may be considerable uncertainty regarding these predicted individualized effects, which are not readily quantifiable, as no individuals received both conditions; thus, no individual contrasts exist that predictions can be compared against. This means that when comparing individual effects against baseline characteristics and engagement data, some of the uncertainty regarding predictions is not accounted for. As others have emphasized previously, more research is needed to develop methods to estimate the uncertainty of the statistical approach taken (Hoogland et al. 2021).

### Conclusions

In conclusion, the findings from this study highlight how different individuals taking part in an RCT of a digital alcohol intervention had heterogeneous outcomes, which was not directly evident from the primary RCT analysis, which focused on comparing the intervention and control groups (Bendtsen et al. 2022). To our knowledge, this is a unique study regarding the individualized effects of a text-message-based alcohol intervention, and we recommend other researchers account for these potential effects when creating statistical plans and designing interventions. In sum, the digital alcohol intervention was best suited to older individuals, women, and those with confidence in their ability to reduce their consumption who deemed it important to change their drinking behaviour.

## Supplementary Material

Individualised_effects_Appendix_A_agae049

Individualised_effects_Appendix_B_agae049

Individualised_effects_Appendix_C_agae049

Individualised_effects_Appendix_D_agae049

Individualised_effects_Appendices_E_to_H_agae049

## Data Availability

Data is available upon reasonable request and signing of a data agreement.
