# Supplementary material for: Individualized treatment effects of a digital alcohol intervention and their associations with participant characteristics and engagement
Source: Alcohol Alcohol. 2024 Jul 21;59(5):agae049. doi: 10.1093/alcalc/agae049 (PMC11260484; doi:10.1093/alcalc/agae049)
Supplement: Individualised_effects_Appendix_A_agae049 [file individualised_effects_appendix_a_agae049.docx]

# Appendix A – Description of Intervention Content

The Bendtsen et al., (2022) intervention was comprised of six individual modules accessed via an interactive dashboard on a web platform. They included -

- **Normative feedback**. Participants compared their current consumption with others of the same age and gender (based on national Swedish data). This module also included an interactive component wherein consumption levels could be changed, resulting in a change of the normative feedback.
- **Information regarding associated general and disease risks of alcohol consumption.** This module provided risk information; participants were also able to adjust consumption levels to see how risks change across the various levels.
- **Self-authored prompts (reminders)**. Participants authored a message for themselves, (for example a plan to deal with a trigger situation), and then selected a date and time to send the message to themselves in the upcoming week.
- **Tips on how to increase knowledge on reducing consumption**. This module provided practical tips on how to reduce consumption (for example, avoiding cues, behaviour substitution and identification of triggers).
- **Progress timeline and goal-setting**. This module enabled participants to view their consumption overtime, with the option to set a goal for their consumption, which could then be reviewed visually to assess their performance.
- **Support SMS**. Participants could request additional support texts sent to them throughout the week. The texts included additional tips for reducing consumption.
