# Supplementary material for: Individualized treatment effects of a digital alcohol intervention and their associations with participant characteristics and engagement
Source: Alcohol Alcohol. 2024 Jul 21;59(5):agae049. doi: 10.1093/alcalc/agae049 (PMC11260484; doi:10.1093/alcalc/agae049)
Supplement: Individualised_effects_Appendix_B_agae049 [file individualised_effects_appendix_b_agae049.pdf]

Appendix – Stan code

Stan code for zero-inflated negative binomial regression model

```
functions {

  real zinb(int outcome, real theta, real eta, real phi) {

    /*
     * Calculate likelihood of data
     */
    real p0 = bernoulli_logit_lpmf(1 | theta); // Likelihood that it is a zero (1 = true that it is zero)
    real pn0 = bernoulli_logit_lpmf(0 | theta); // Likelihood that it is not a zero (0 = false that it is a zer
    real nbll = neg_binomial_2_log_lpmf(outcome | eta, phi);

    if (outcome == 0) {
      return log_sum_exp(p0, pn0 + nbll);
    } else {
      return pn0 + nbll;
    }

  }

}

data {

  // --  Estimation ------------------------------------- //

  /*
   * Design matrix with N rows and D columns.
   * Note that the design matrix should *not* contain
   * a column representing the main intercept.
   */
  int<lower=1> N;
  int<lower=1> D;
  matrix[N,D] DESIGN;

  /*
   * Level 1 values for each row in the design matrix,
   * with L1_D unique values.
   */
  int<lower=1> L1_D;
  array[N] int<lower=1> L1;

  /*
   * Outcome data
   */
  array[N] int<lower=0> OUTCOME;

  /*
   * Column indices containing non-interaction and interaction data.
   */
  int<lower=0> D_NINT;
  int<lower=0> D_INT;
  array[D_NINT] int<lower=1> NINT;
  array[D_INT] int<lower=1> INT;

  // --  Heterogeneous treatment effects ------------------ //

  /*
   * Two design matrices with contrast = 0 and contrast = 1
   *
   * Note that the design matrices should *not* contain
   * a column representing the main intercept.
   */
  int<lower=0> HTE_N;
  matrix[HTE_N,D] HTE_DESIGN_0;
  matrix[HTE_N,D] HTE_DESIGN_1;

  /*
   * Level 1 values for each row in HTE design matrices.
   */
  array[HTE_N] int<lower=0> HTE_L1;


}

parameters {
```

```
  /*
   * Main intercept
   */
  real alpha_theta;
  real alpha_eta;

  /*
   * Standardized L1 intercepts l1_z
   * with standard deviation l1_sd
   */
  vector[L1_D] l1_z_theta;
  vector[L1_D] l1_z_eta;
  real<lower=0> l1_sd_theta;
  real<lower=0> l1_sd_eta;

  /*
   * Covariate coefficients for non interaction covariates
   */
  vector[D_NINT] beta_theta_nint;
  vector[D_NINT] beta_eta_nint;

  /*
   * Re-parameterised interaction covariate coefficients
   * ( beta ~ cauchy(0, tau) )
   */
  vector<lower=-pi()/2, upper=pi()/2>[D_INT] beta_theta_int_unif;
  vector<lower=-pi()/2, upper=pi()/2>[D_INT] beta_eta_int_unif;
  real<lower=0> tau_theta_int;
  real<lower=0> tau_eta_int;

  /*
   * Dispersion parameter (for negative binomial part of model)
   */
  real<lower=0> phi;

}

transformed parameters {

  /*
   * Re-parameterisation of interaction covariate coefficients
   * ( beta ~ cauchy(0, tau) )
   */
  vector[D_INT] beta_theta_int = tau_theta_int * tan(beta_theta_int_unif);
  vector[D_INT] beta_eta_int = tau_eta_int * tan(beta_eta_int_unif);

  /*
   * Collect non interaction and interaction betas
   * into one matrix.
   */
  matrix[D, 2] beta;
  for (i in 1:D_NINT) {
    beta[NINT[i], 1] = beta_theta_nint[i];
    beta[NINT[i], 2] = beta_eta_nint[i];
  }
  for (i in 1:D_INT) {
    beta[INT[i], 1] = beta_theta_int[i];
    beta[INT[i], 2] = beta_eta_int[i];
  }

  /*
   * Theta = linear comb. logit
   * Eta = log(mu) is the linear combination of the
   * intercept and each row in the data set
   */
  vector[N] theta = alpha_theta + DESIGN * beta[,1];
  vector[N] eta = alpha_eta + DESIGN * beta[,2];

  /*
   * De-standardize adaptive l1 intercepts
   */
  vector[L1_D] l1_theta = l1_z_theta * l1_sd_theta;
  vector[L1_D] l1_eta = l1_z_eta * l1_sd_eta;

  /*
   * Add adaptive intercepts to eta and theta
   */
  for (n in 1:N) {
    theta[n] += l1_theta[L1[n]];
    eta[n] += l1_eta[L1[n]];
  }
```

```
}

model {

  // --  Priors ------------------------------------------ //

  /*
   * Main intercept
   */
  target += normal_lpdf(alpha_theta | 0, 1.5);
  target += normal_lpdf(alpha_eta | 0, 50);

  /*
   * Non interaction covariate coefficients
   */
  target += student_t_lpdf(beta_theta_nint | 3, 0, 2.5);
  target += student_t_lpdf(beta_eta_nint | 3, 0, 2.5);

  /*
   * Re-parameterised interaction covariate coefficients
   */
  target += uniform_lpdf(beta_theta_int_unif | -pi()/2, pi()/2);
  target += uniform_lpdf(beta_eta_int_unif | -pi()/2, pi()/2);
  target += normal_lpdf(tau_theta_int | 0, 1);
  target += normal_lpdf(tau_eta_int | 0, 1);

  /*
   * Dispersion
   */
  target += student_t_lpdf(phi | 3, 0, 2.5);

  /*
   * L1 intercepts
   */
  target += normal_lpdf(l1_z_theta | 0, 1);
  target += normal_lpdf(l1_z_eta | 0, 1);
  target += normal_lpdf(l1_sd_theta | 0, 1);
  target += normal_lpdf(l1_sd_eta | 0, 1);


  // --  Outcome model ---------------------------------- //
  for (n in 1:N) {
    target += zinb(OUTCOME[n], theta[n], eta[n], phi);
  }

}

generated quantities {

  // --  HTE --------------------------------------------- //

  vector[HTE_N] hte;

  /*
   * Calculate eta and theta for each individual for
   * both contrasts.
   */
  vector[HTE_N] contrast_0_theta = alpha_theta + HTE_DESIGN_0 * beta[,1];
  vector[HTE_N] contrast_0_eta = alpha_eta + HTE_DESIGN_0 * beta[,2];

  vector[HTE_N] contrast_1_theta = alpha_theta + HTE_DESIGN_1 * beta[,1];
  vector[HTE_N] contrast_1_eta = alpha_eta + HTE_DESIGN_1 * beta[,2];

  /*
   * Predict outcomes under both contrasts.
   */
  vector[HTE_N] contrast_0;
  vector[HTE_N] contrast_1;
  for (n in 1:HTE_N) {

    /*
     * Add adaptive intercepts to both eta and theta contrasts.
     */
    contrast_0_theta[n] += l1_theta[HTE_L1[n]];
    contrast_1_theta[n] += l1_theta[HTE_L1[n]];

    contrast_0_eta[n] += l1_eta[HTE_L1[n]];
    contrast_1_eta[n] += l1_eta[HTE_L1[n]];

    /*
     * First predict 0 or 1, and then predict from negbin if not 0.
     * NOTE NOTE NOTE: When the rng returns a 1 it has predicted a zero
```

```
     * (i.e. a value of "true" that it is zero).
     */
    int contrast_0_zero_true = bernoulli_logit_rng(contrast_0_theta[n]);
    int contrast_1_zero_true = bernoulli_logit_rng(contrast_1_theta[n]);

    if (contrast_0_zero_true == 1) {
      contrast_0[n] = 0;
    } else {
      contrast_0[n] = neg_binomial_2_log_rng(contrast_0_eta[n], phi);
    }

    if (contrast_1_zero_true == 1) {
      contrast_1[n] = 0;
    } else {
      contrast_1[n] = neg_binomial_2_log_rng(contrast_1_eta[n], phi);
    }

  }

  /*
   * Set the contrast (HTE) for each individual for this MCMC iteration.
   */
  hte = contrast_1 - contrast_0;

}
```
