# Supplementary material for: Individualized treatment effects of a digital alcohol intervention and their associations with participant characteristics and engagement
Source: Alcohol Alcohol. 2024 Jul 21;59(5):agae049. doi: 10.1093/alcalc/agae049 (PMC11260484; doi:10.1093/alcalc/agae049)

# Appendix c – Markov Chain Monte Carlo Output Analysis

## Heavy episodic drinking

In Table 1, we provide an overview of the summary statistics, effective sample size, and R-hat for the estimation of the zero-inflated negative binomial regression mode for heavy episodic drinking. The R-hat value, which was within the acceptable bounds of 1, indicates the convergence of the Markov chains. Convergence is further illustrated in the trace plots following the table, which include samples after warmup.

Table 1 - Summary statistics, effective sample size, and R-hat for estimation of the zero-inflated negative binomial regression mode for heavy episodic drinking

| **Parameter** | **Mean (exp)** | **5% (exp)** | **50% (exp)** | **95% (exp)** | **N_eff** | **N_eff/s** | **R-hat** |
| --- | --- | --- | --- | --- | --- | --- | --- |
| alpha_theta | 0.00326989 | 0.000941534 | 0.00337284 | 0.0102446 | 7266 | 0.9507 | 0.9999 |
| alpha_eta | 3.45907 | 3.18038 | 3.45907 | 3.76219 | 4657 | 0.6093 | 1.001 |
| beta[1,1] | 0.983065 | 0.26955 | 1.01334 | 3.24787 | 3693 | 0.4832 | 1 |
| beta[2,1] | 0.387515 | 0.0072265 | 0.444236 | 13.3164 | 8378 | 1.096 | 1 |
| beta[3,1] | 1.05293 | 0.813833 | 1.00467 | 1.59504 | 12396 | 1.622 | 0.9999 |
| beta[4,1] | 0.0523397 | 3.50057e-05 | 0.117068 | 5.84742 | 3131 | 0.4097 | 1.001 |
| beta[5,1] | 0.935859 | 0.242198 | 0.928848 | 3.651 | 10545 | 1.38 | 1 |
| beta[6,1] | 1.17951 | 0.0258098 | 1.32432 | 40.813 | 11245 | 1.471 | 1 |
| beta[7,1] | 0.933093 | 0.586724 | 0.994561 | 1.21094 | 13392 | 1.752 | 1 |
| beta[8,1] | 1.07152 | 1.01062 | 1.07011 | 1.14225 | 6094 | 0.7974 | 1.001 |
| beta[9,1] | 1.04017 | 0.96781 | 1.04042 | 1.11762 | 3917 | 0.5125 | 1.001 |
| beta[10,1] | 0.969272 | 0.869358 | 0.971047 | 1.07604 | 4153 | 0.5433 | 1 |
| beta[11,1] | 0.920729 | 0.732201 | 0.918632 | 1.16369 | 8220 | 1.076 | 1 |
| beta[12,1] | 1.4792 | 0.866667 | 1.41468 | 2.9388 | 2658 | 0.3478 | 1.001 |
| beta[13,1] | 1.20322 | 0.981405 | 1.2031 | 1.48112 | 8890 | 1.163 | 1 |
| beta[14,1] | 0.925186 | 0.829444 | 0.932235 | 1.00348 | 2447 | 0.3202 | 1 |
| beta[15,1] | 0.999696 | 0.92766 | 0.999085 | 1.08105 | 10866 | 1.422 | 1.001 |
| beta[16,1] | 1.24658 | 1.01742 | 1.24023 | 1.55053 | 1937 | 0.2535 | 1 |
| beta[17,1] | 0.9678 | 0.783018 | 0.989882 | 1.10871 | 11785 | 1.542 | 1 |
| beta[18,1] | 1.03604 | 0.870489 | 1.00671 | 1.37081 | 11725 | 1.534 | 1 |
| beta[19,1] | 0.987953 | 0.836273 | 0.995986 | 1.13712 | 15573 | 2.038 | 0.9999 |
| beta[20,1] | 1.04069 | 0.976032 | 1.04048 | 1.11027 | 10276 | 1.345 | 1 |
| beta[21,1] | 1.0334 | 0.953496 | 1.03301 | 1.12243 | 7122 | 0.9319 | 1 |
| beta[22,1] | 0.909582 | 0.796602 | 0.91545 | 1.02252 | 4480 | 0.5862 | 1.001 |
| beta[23,1] | 0.945634 | 0.69191 | 0.952372 | 1.2743 | 6365 | 0.8328 | 1 |
| beta[24,1] | 1.26554 | 0.71878 | 1.19315 | 2.66606 | 3328 | 0.4354 | 1.001 |
| beta[25,1] | 0.801236 | 0.600496 | 0.804367 | 1.0543 | 9551 | 1.25 | 1 |
| beta[26,1] | 1.01134 | 0.947612 | 1.00608 | 1.09474 | 6924 | 0.9059 | 1 |
| beta[27,1] | 0.979983 | 0.886477 | 0.989852 | 1.05169 | 6180 | 0.8086 | 1 |
| beta[28,1] | 1.06332 | 0.95301 | 1.03953 | 1.26706 | 8653 | 1.132 | 1 |
| beta[29,1] | 1.03753 | 0.889674 | 1.00972 | 1.32141 | 8229 | 1.077 | 1 |
| beta[30,1] | 1.11862 | 0.912242 | 1.02447 | 1.86489 | 7314 | 0.957 | 1.001 |
| beta[31,1] | 0.953258 | 0.733227 | 0.986295 | 1.09809 | 9177 | 1.201 | 1 |
| beta[1,2] | 0.935934 | 0.845354 | 0.936234 | 1.03605 | 7315 | 0.9571 | 1.001 |
| beta[2,2] | 1.40425 | 0.848827 | 1.39696 | 2.32263 | 4477 | 0.5859 | 1.001 |
| beta[3,2] | 1.00261 | 0.985752 | 1.00018 | 1.02698 | 13024 | 1.704 | 1 |
| beta[4,2] | 0.913383 | 0.606652 | 0.913191 | 1.3752 | 13650 | 1.786 | 1 |
| beta[5,2] | 1.01113 | 0.908964 | 1.01147 | 1.12401 | 18159 | 2.376 | 0.9998 |
| beta[6,2] | 0.881791 | 0.620394 | 0.883203 | 1.26049 | 13335 | 1.745 | 1 |
| beta[7,2] | 1.00644 | 0.987203 | 1.0004 | 1.0495 | 12873 | 1.684 | 1 |
| beta[8,2] | 1.00229 | 0.996234 | 1.0023 | 1.00828 | 5722 | 0.7487 | 1.002 |
| beta[9,2] | 1.02336 | 1.01704 | 1.02341 | 1.02967 | 7452 | 0.9751 | 1 |
| beta[10,2] | 1.04658 | 1.03744 | 1.04652 | 1.05608 | 6837 | 0.8946 | 1.001 |
| beta[11,2] | 0.945416 | 0.9238 | 0.944575 | 0.970494 | 7088 | 0.9274 | 1 |
| beta[12,2] | 0.974433 | 0.941454 | 0.974062 | 1.00998 | 6875 | 0.8996 | 1 |
| beta[13,2] | 1.02285 | 1.00357 | 1.02291 | 1.04261 | 10950 | 1.433 | 1 |
| beta[14,2] | 0.989535 | 0.98113 | 0.989327 | 0.998804 | 4811 | 0.6295 | 1.001 |
| beta[15,2] | 1.00041 | 0.99535 | 1.00019 | 1.00612 | 12855 | 1.682 | 1 |
| beta[16,2] | 0.997986 | 0.988734 | 0.999015 | 1.00429 | 9466 | 1.239 | 1 |
| beta[17,2] | 0.988072 | 0.951734 | 0.995731 | 1.00346 | 4957 | 0.6486 | 1.001 |
| beta[18,2] | 0.996427 | 0.972651 | 0.999416 | 1.00896 | 9623 | 1.259 | 1 |
| beta[19,2] | 0.99884 | 0.985792 | 0.999699 | 1.00906 | 19213 | 2.514 | 0.9999 |
| beta[20,2] | 1.00011 | 0.995201 | 1.00012 | 1.00503 | 17487 | 2.288 | 1 |
| beta[21,2] | 1.00278 | 0.996536 | 1.00282 | 1.00892 | 15642 | 2.047 | 1 |
| beta[22,2] | 0.998873 | 0.990463 | 0.998861 | 1.00736 | 15613 | 2.043 | 1 |
| beta[23,2] | 1.01199 | 0.988912 | 1.01163 | 1.03709 | 12747 | 1.668 | 1 |
| beta[24,2] | 1.00578 | 0.970863 | 1.00588 | 1.04172 | 17775 | 2.326 | 0.9999 |
| beta[25,2] | 0.991275 | 0.971484 | 0.991152 | 1.01159 | 19157 | 2.507 | 0.9999 |
| beta[26,2] | 1.00074 | 0.9958 | 1.00033 | 1.00669 | 16345 | 2.139 | 1 |
| beta[27,2] | 1.00145 | 0.996523 | 1.00084 | 1.00803 | 17467 | 2.285 | 0.9999 |
| beta[28,2] | 1.0002 | 0.993092 | 1.00008 | 1.00756 | 22904 | 2.997 | 1 |
| beta[29,2] | 0.992746 | 0.96254 | 0.998068 | 1.00525 | 10746 | 1.406 | 1 |
| beta[30,2] | 0.999395 | 0.983931 | 0.999905 | 1.0129 | 19348 | 2.532 | 1 |
| beta[31,2] | 0.998098 | 0.983537 | 0.999524 | 1.00813 | 21308 | 2.788 | 0.9999 |


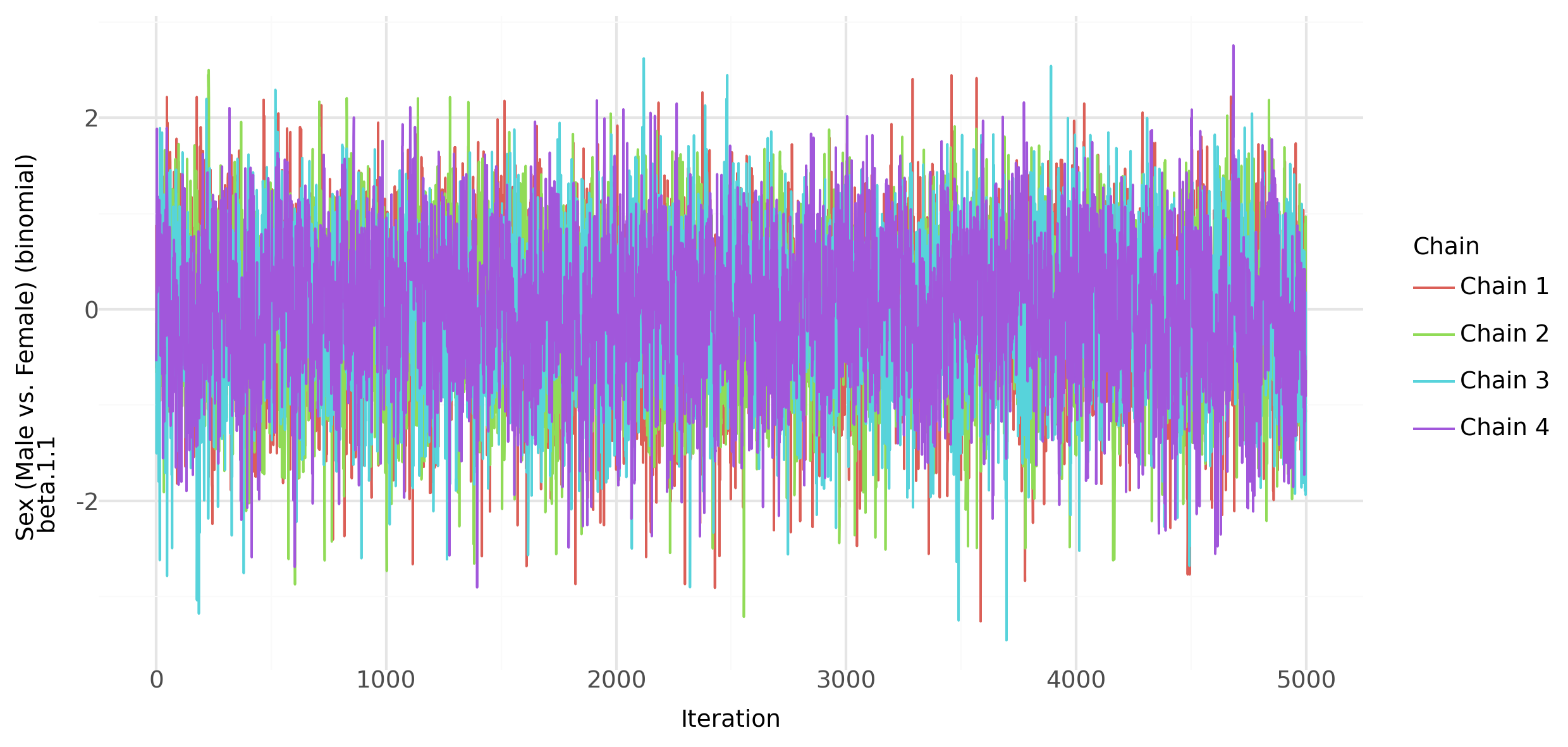


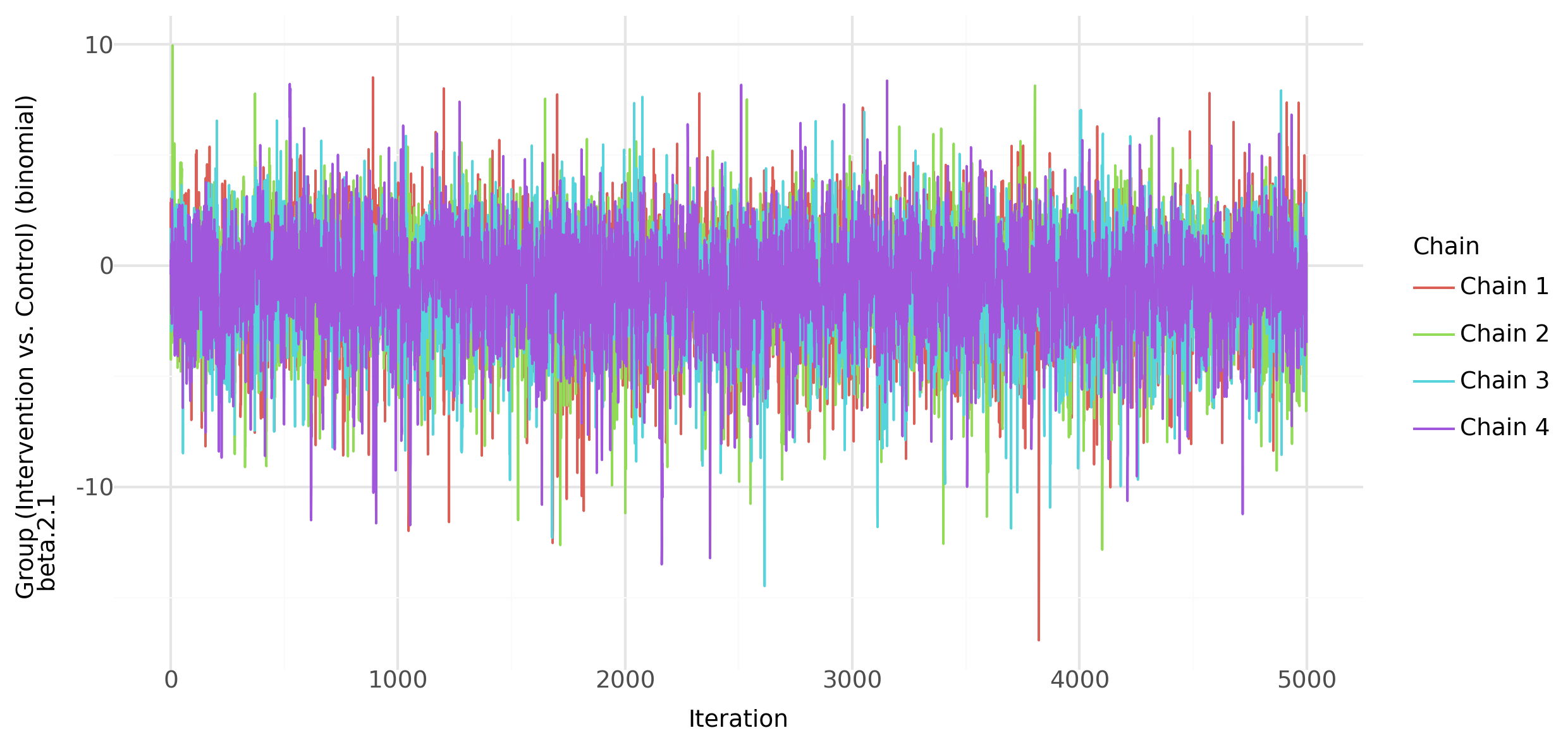


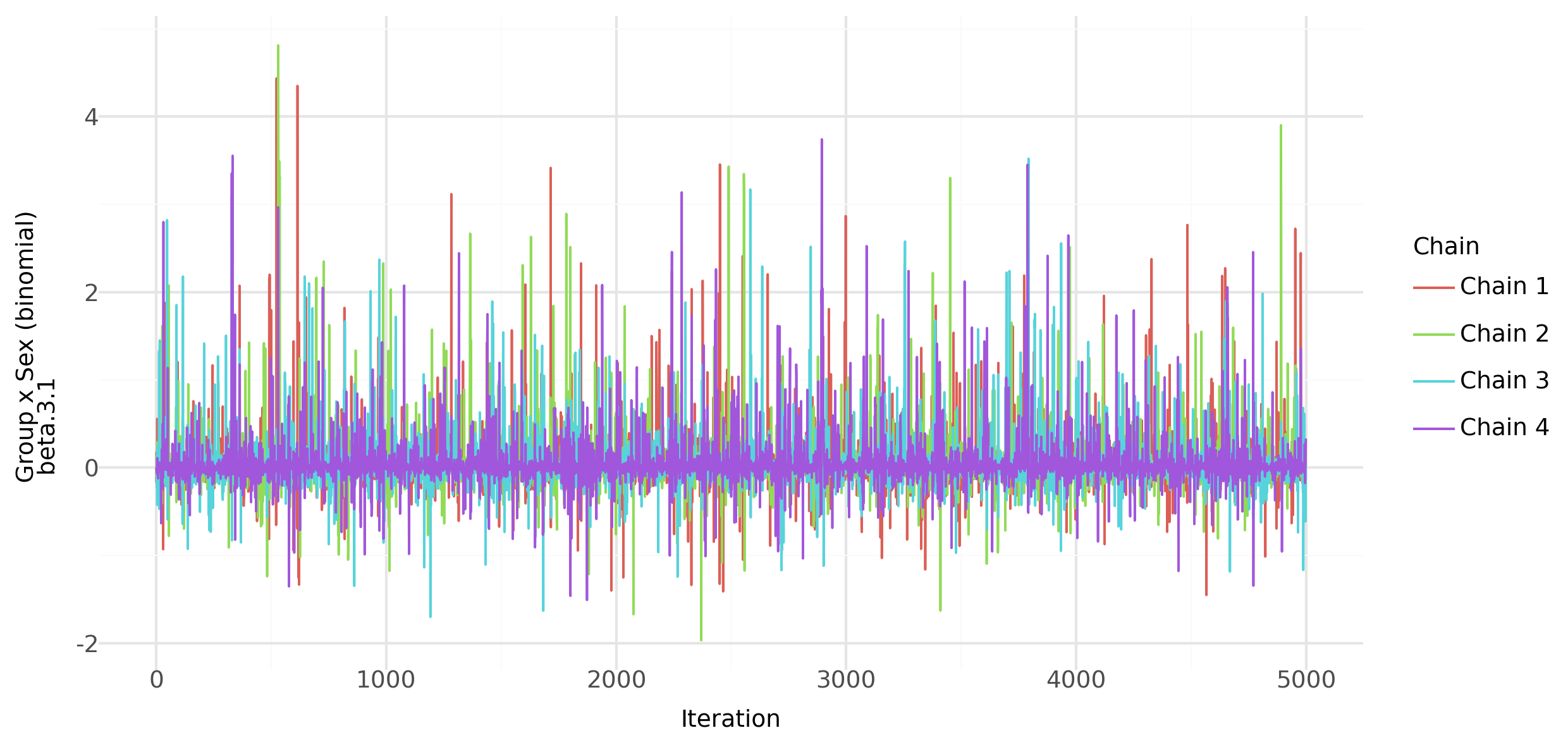


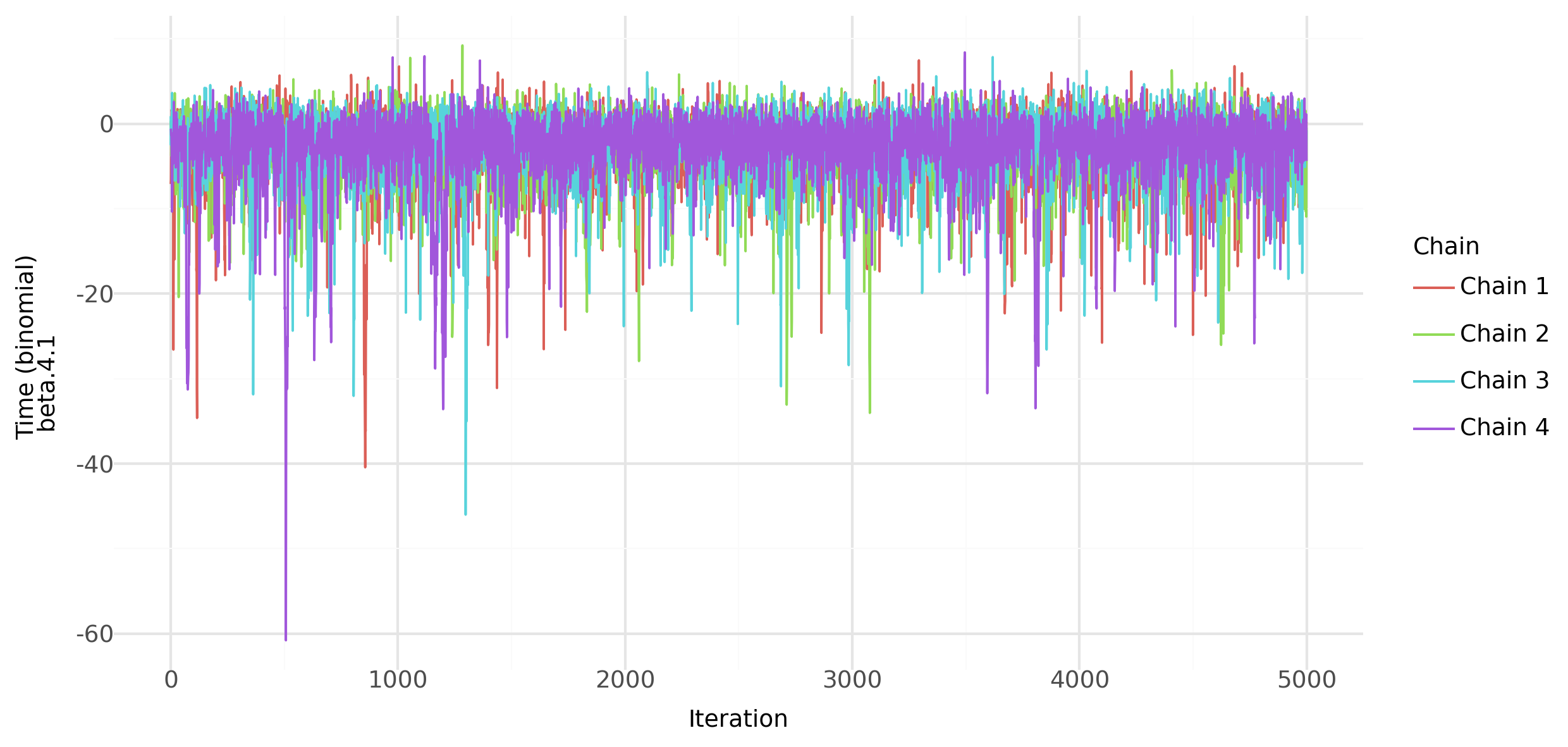


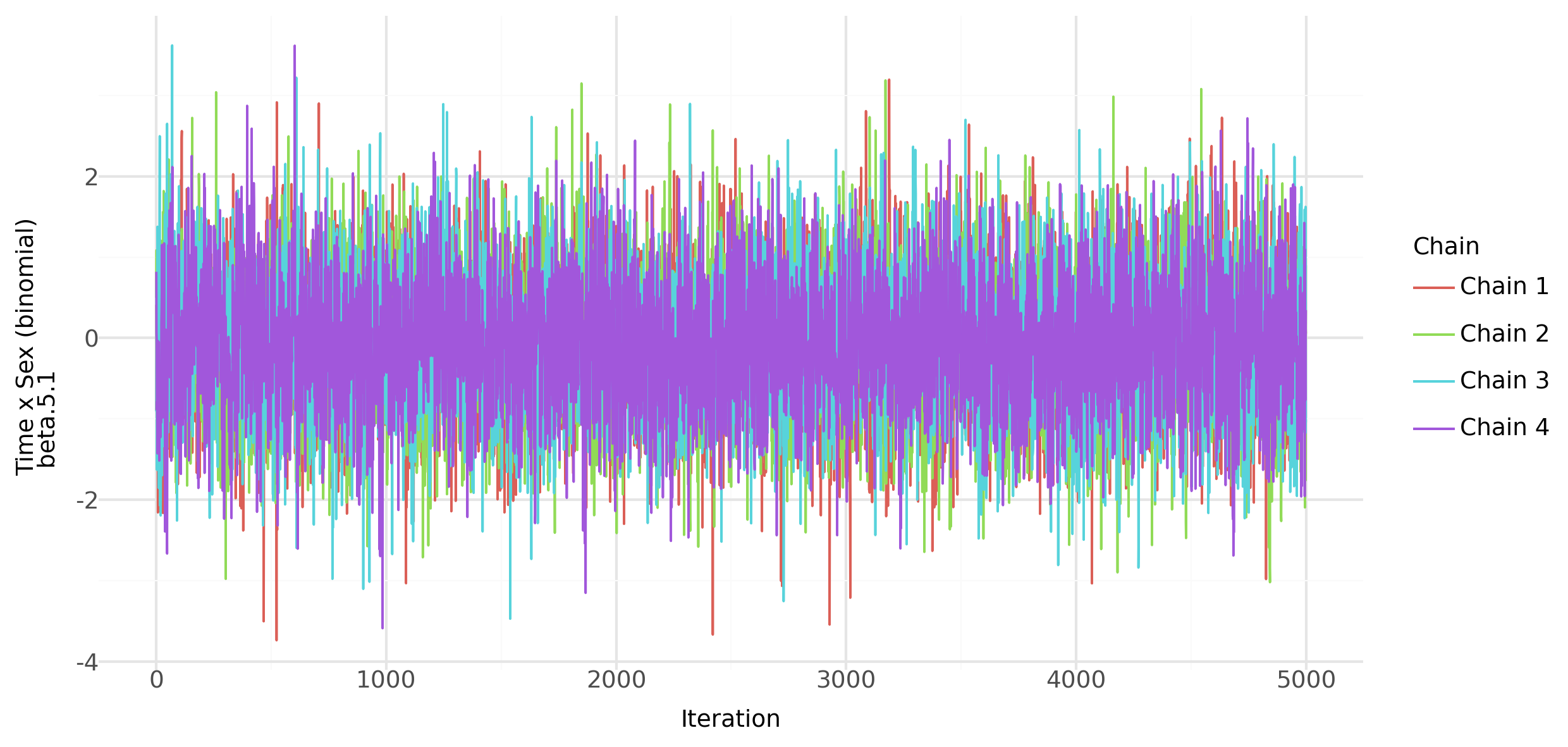


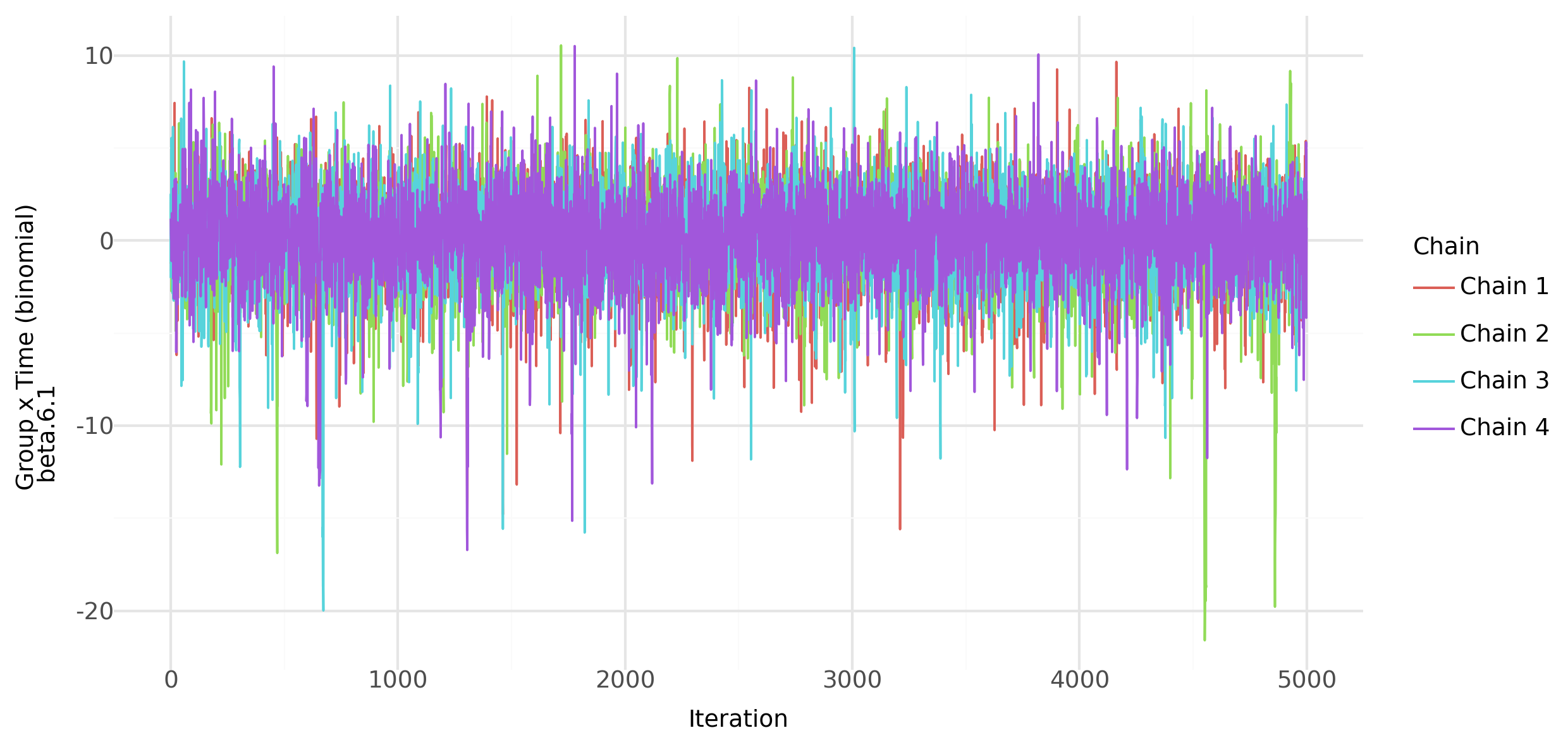


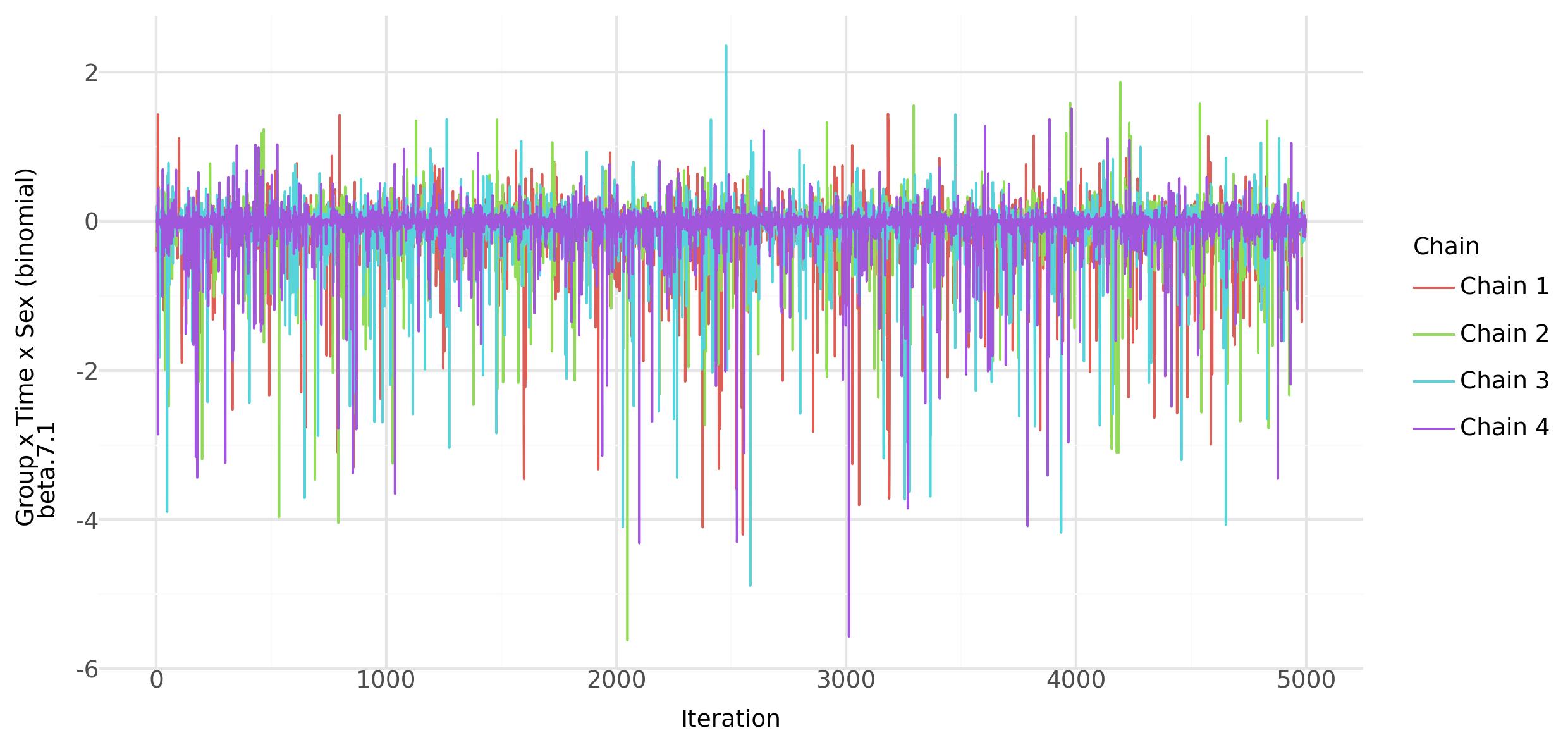


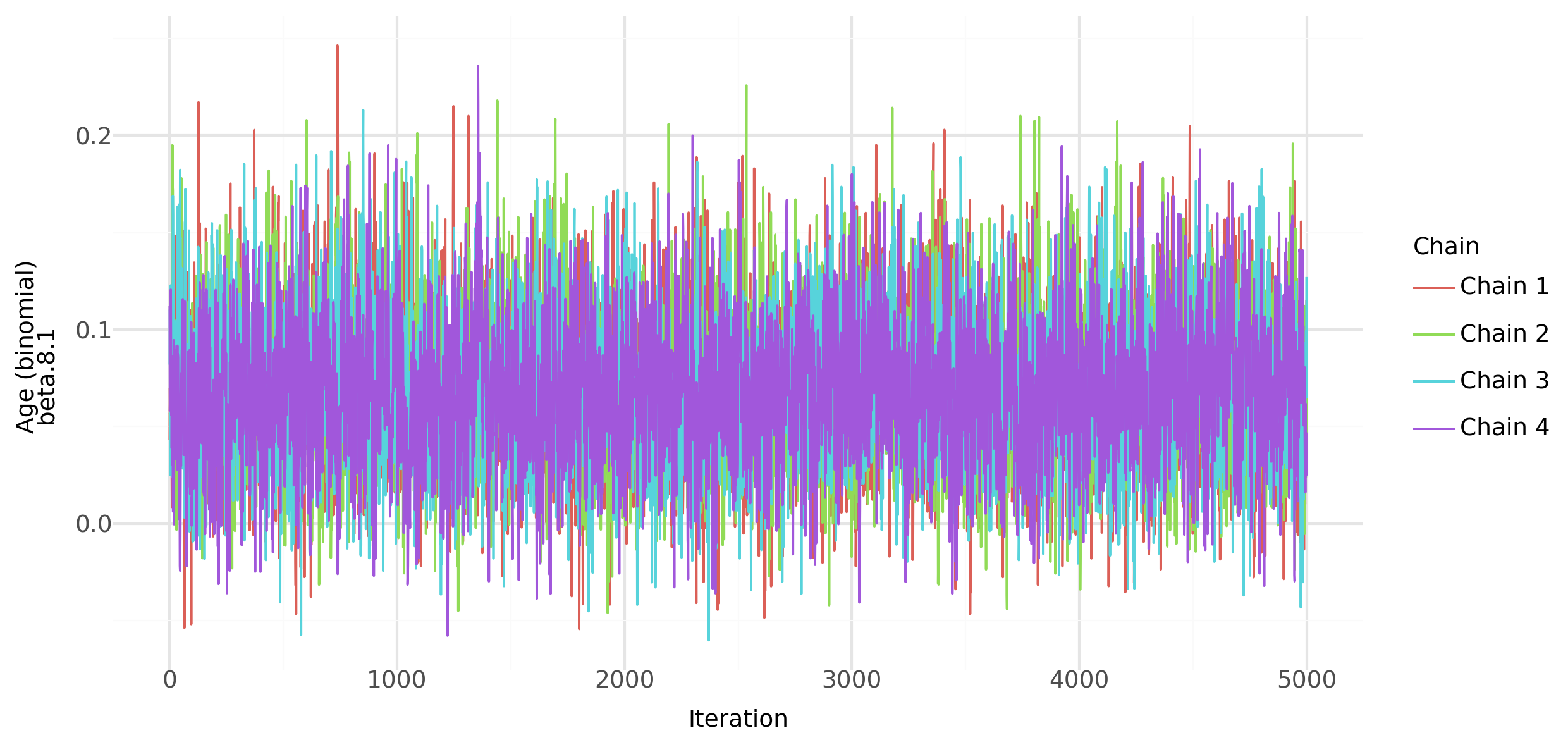


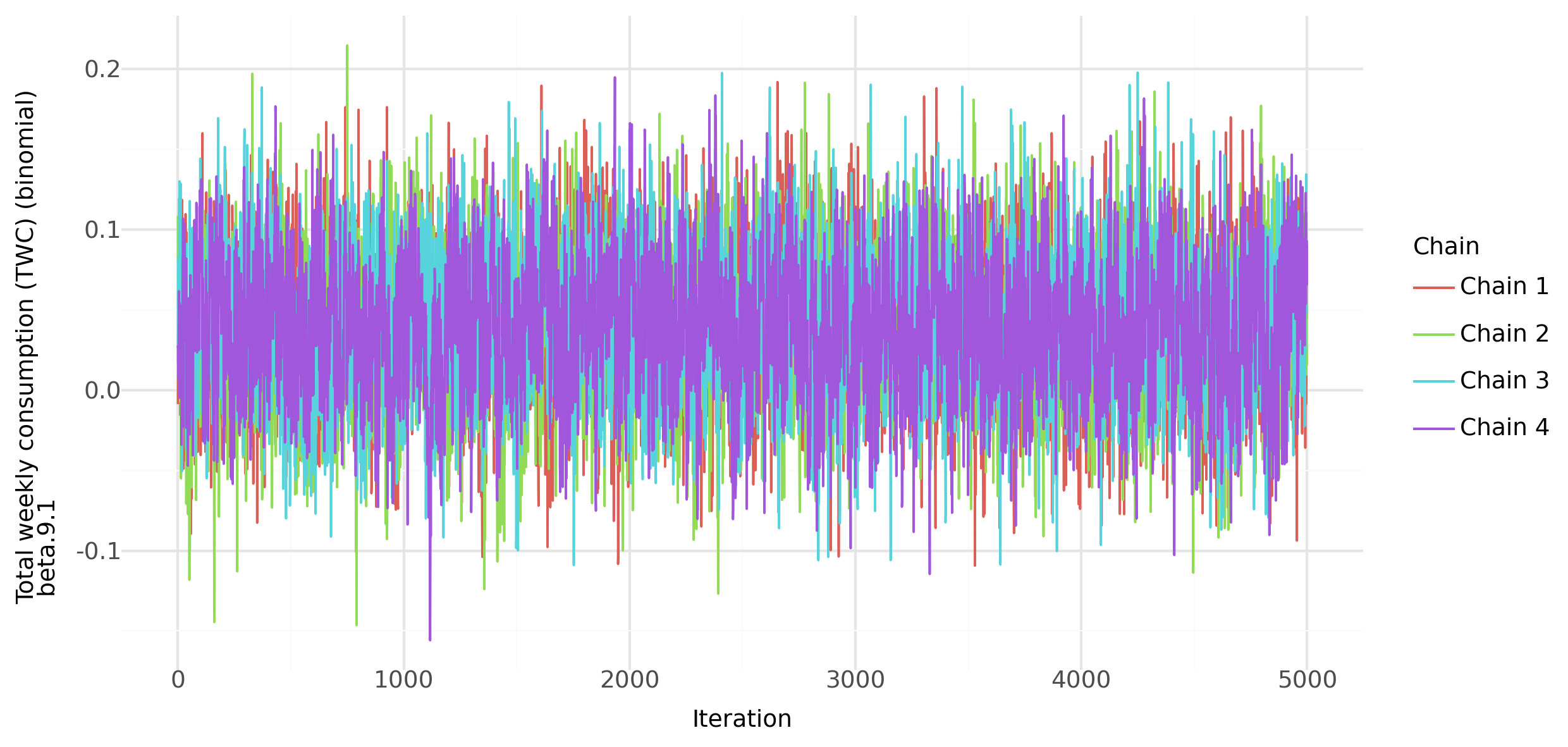


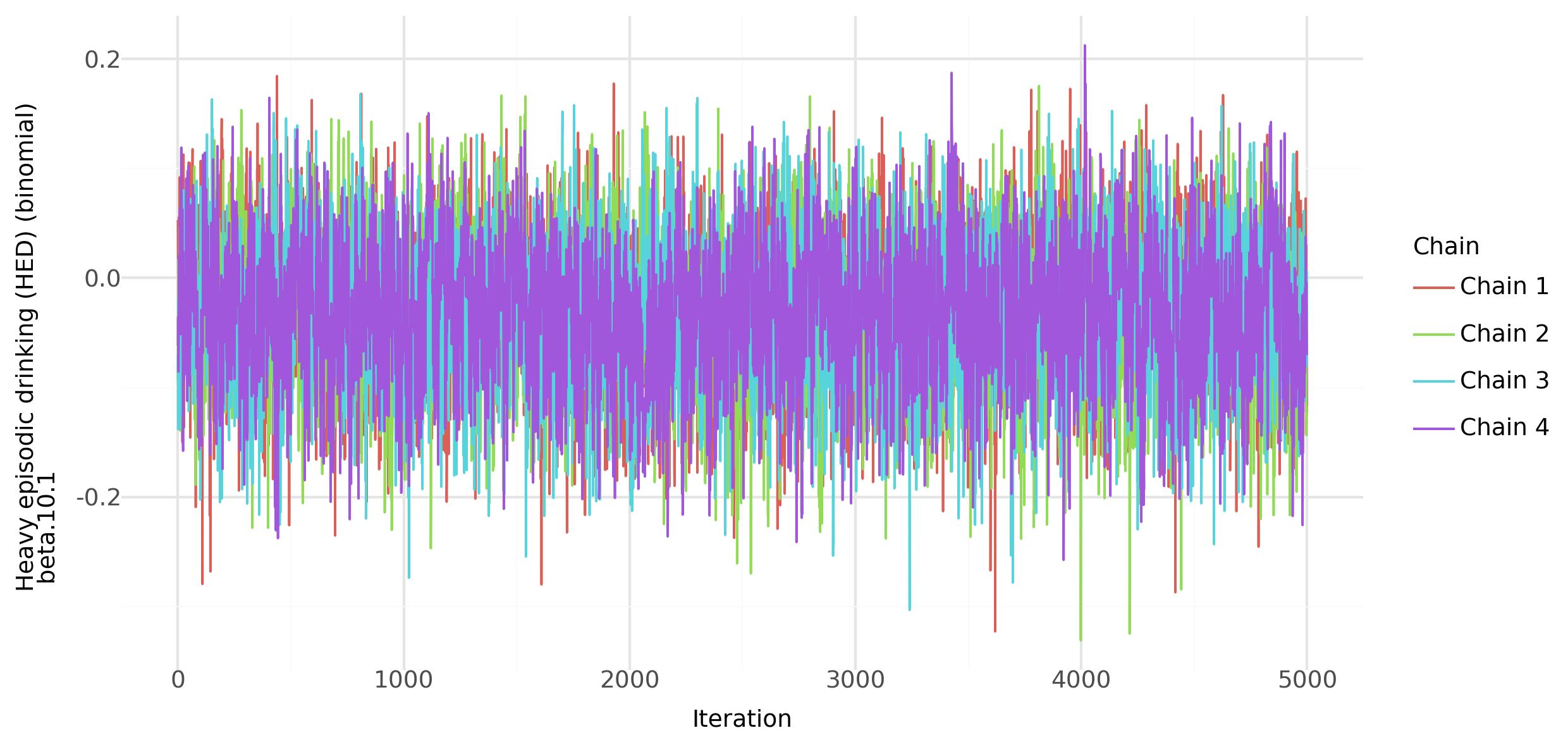


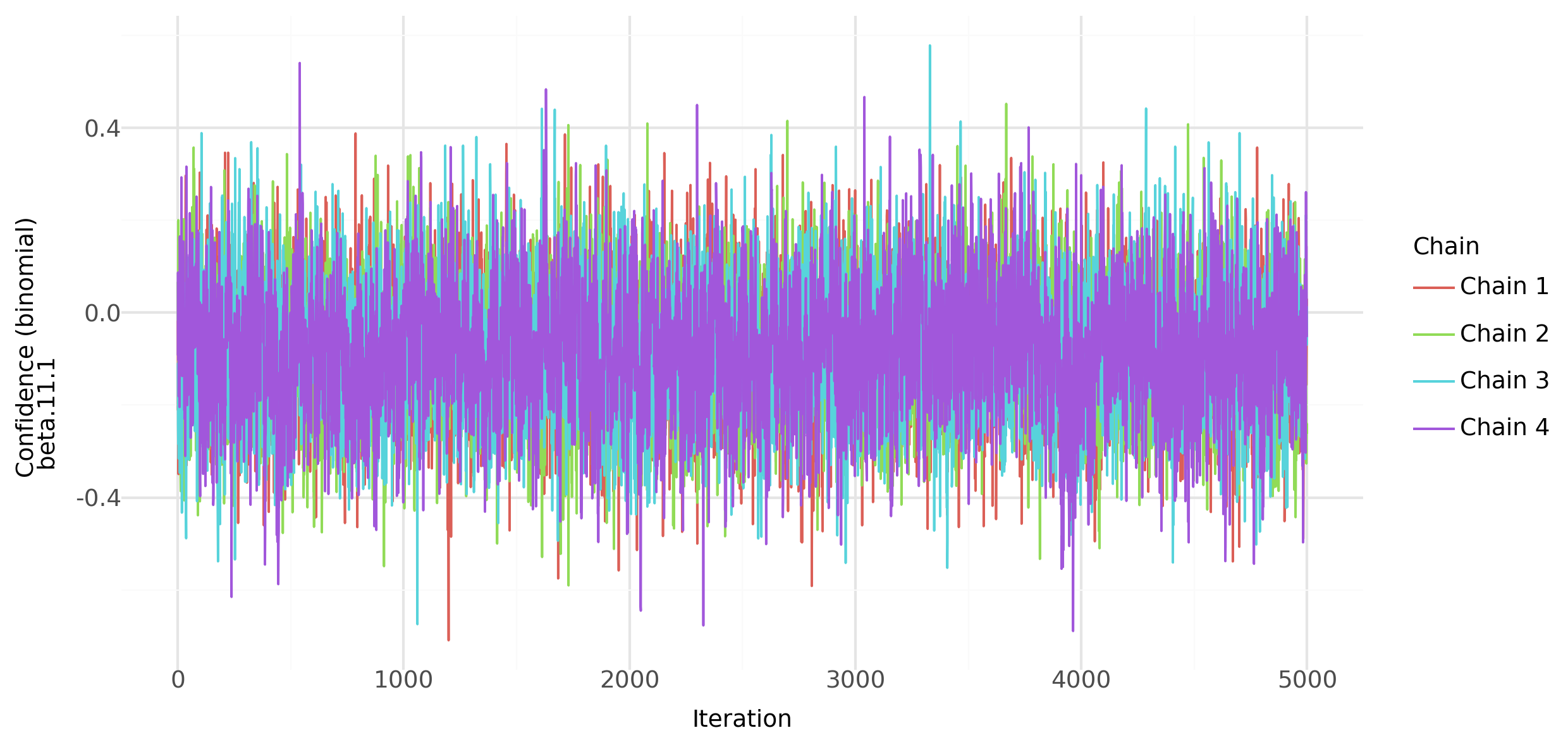


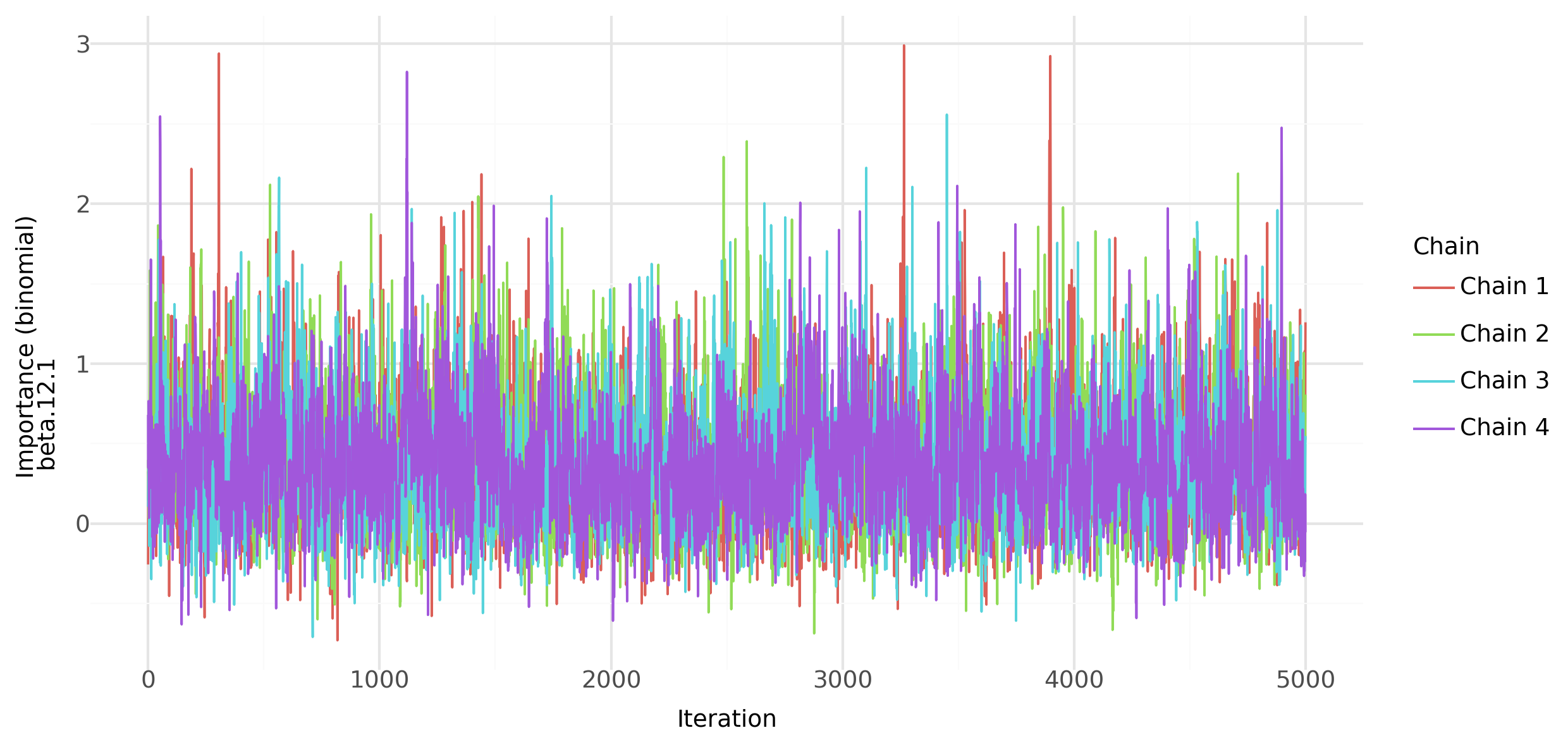


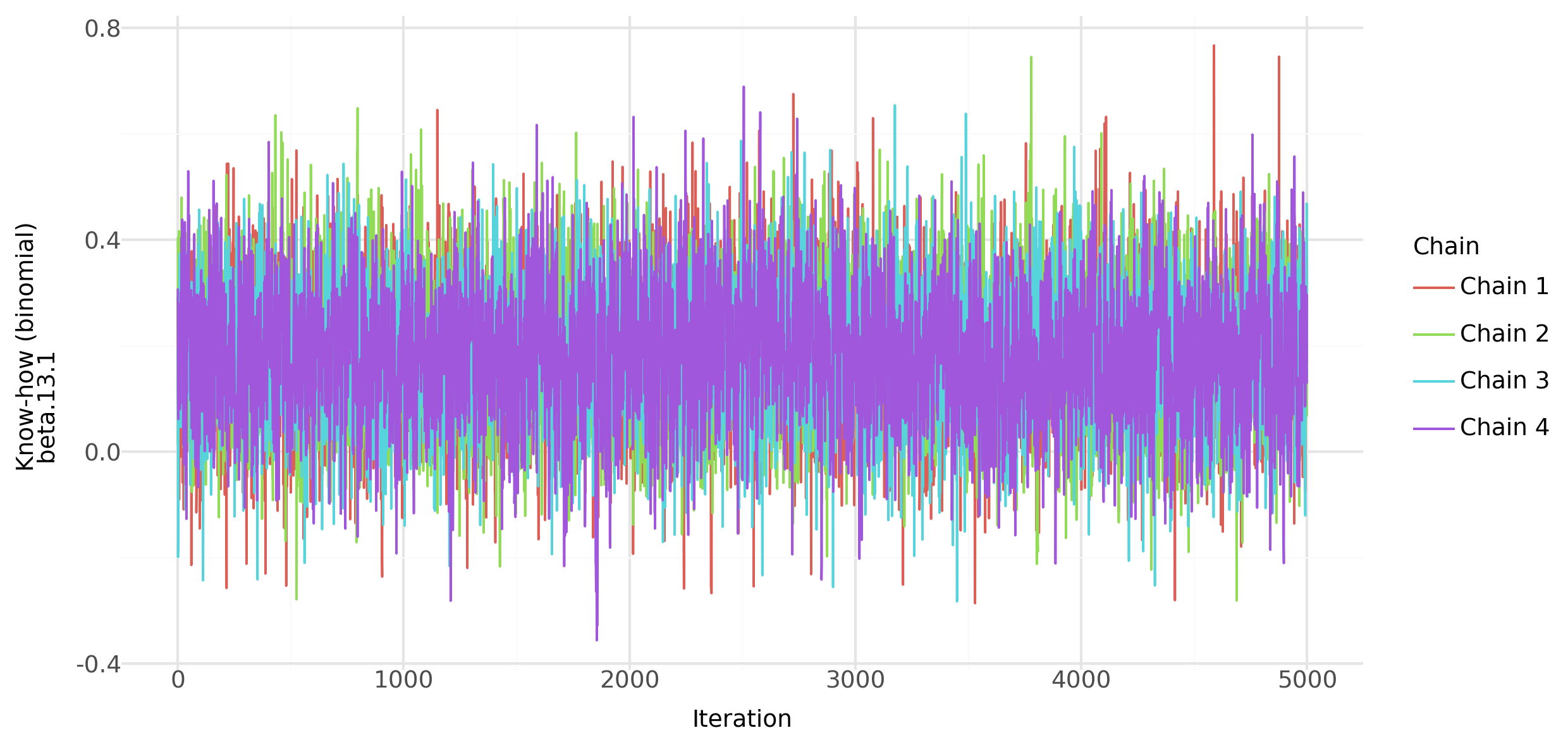


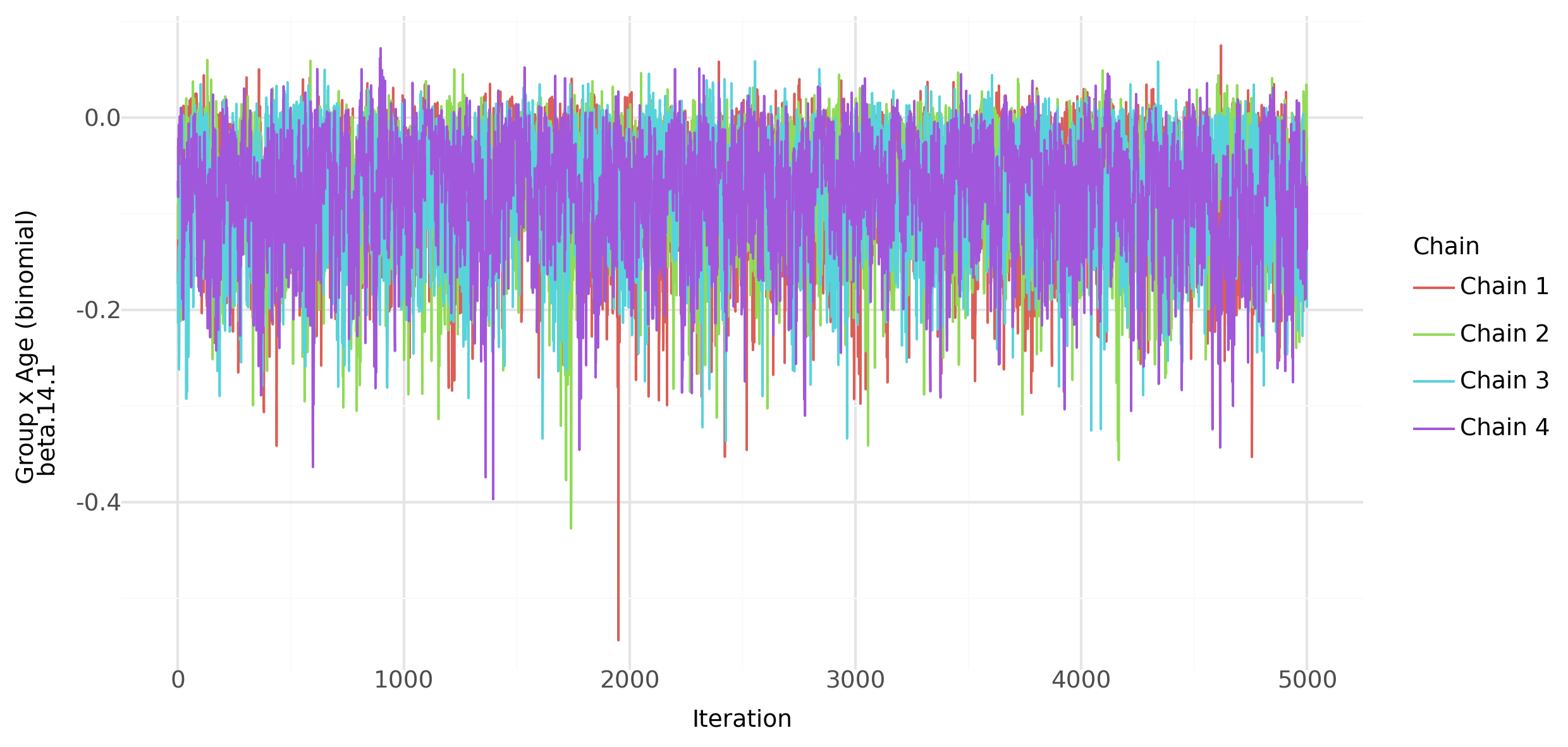


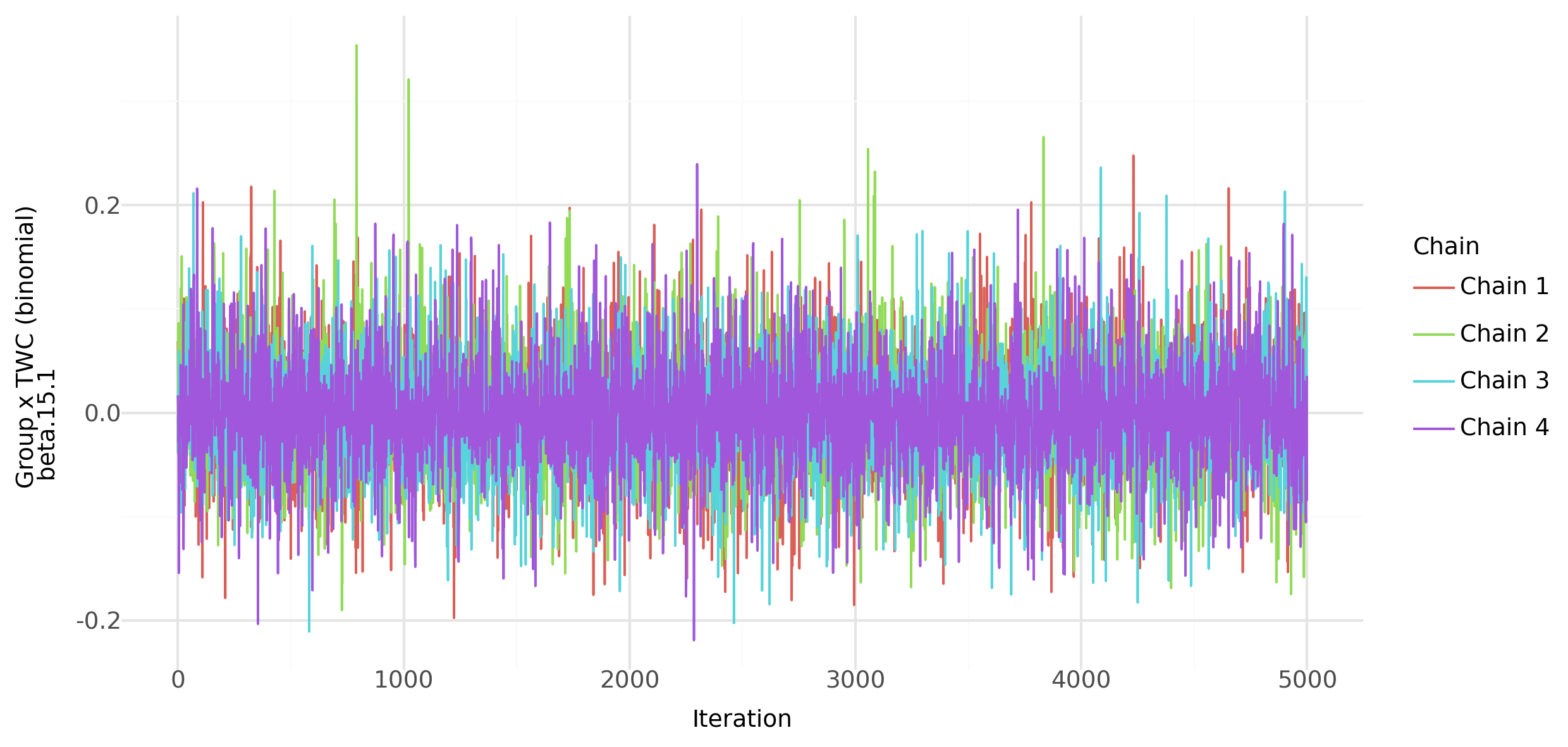


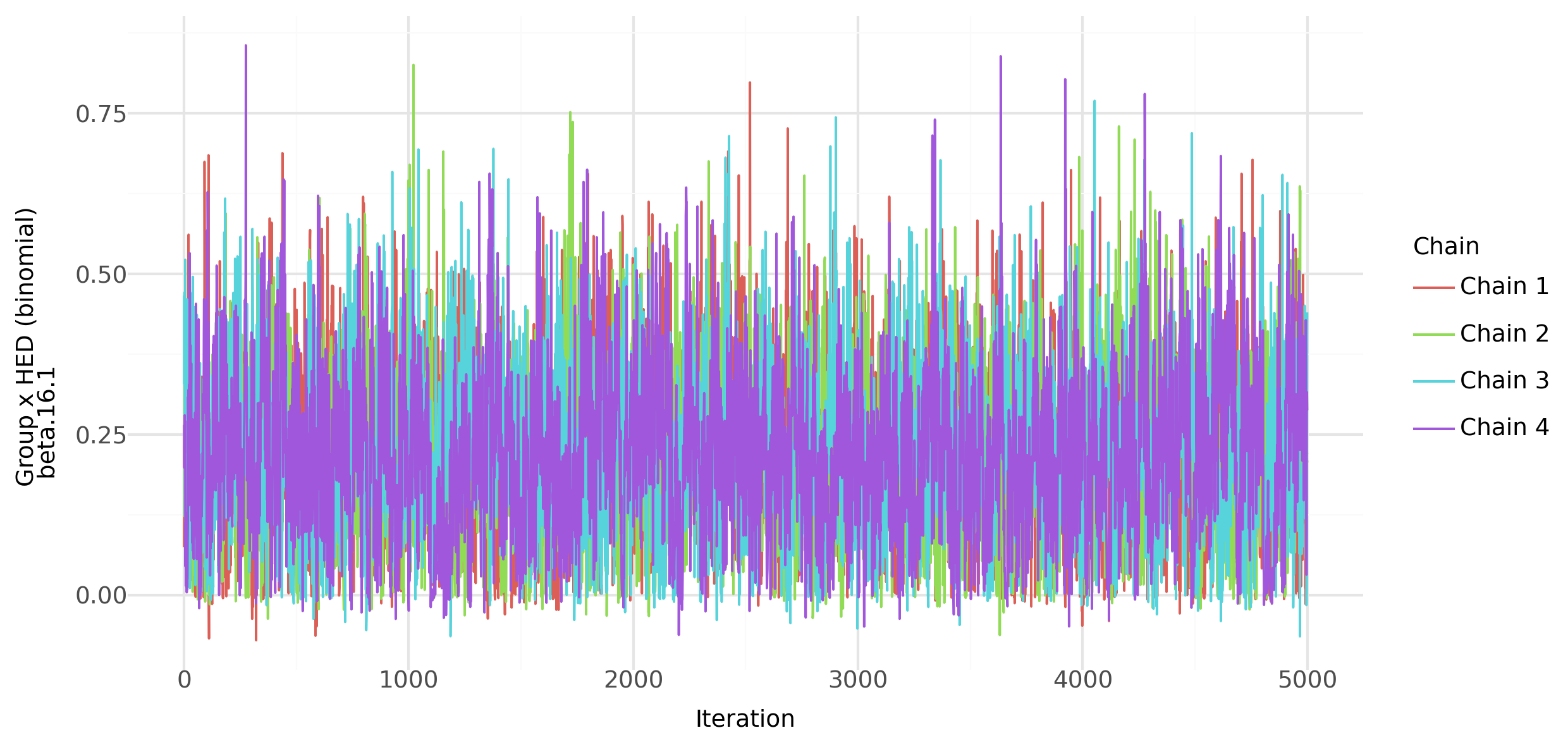


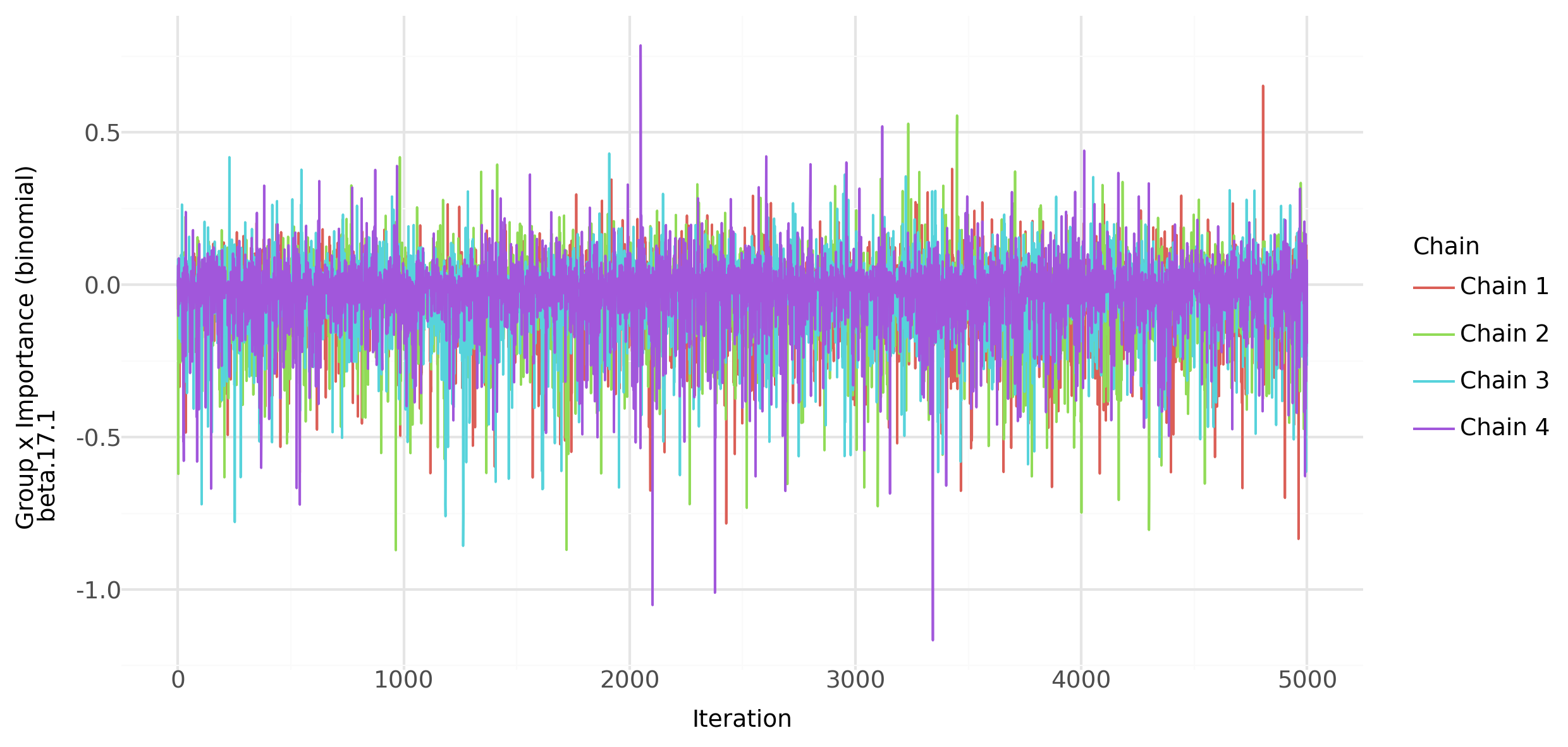


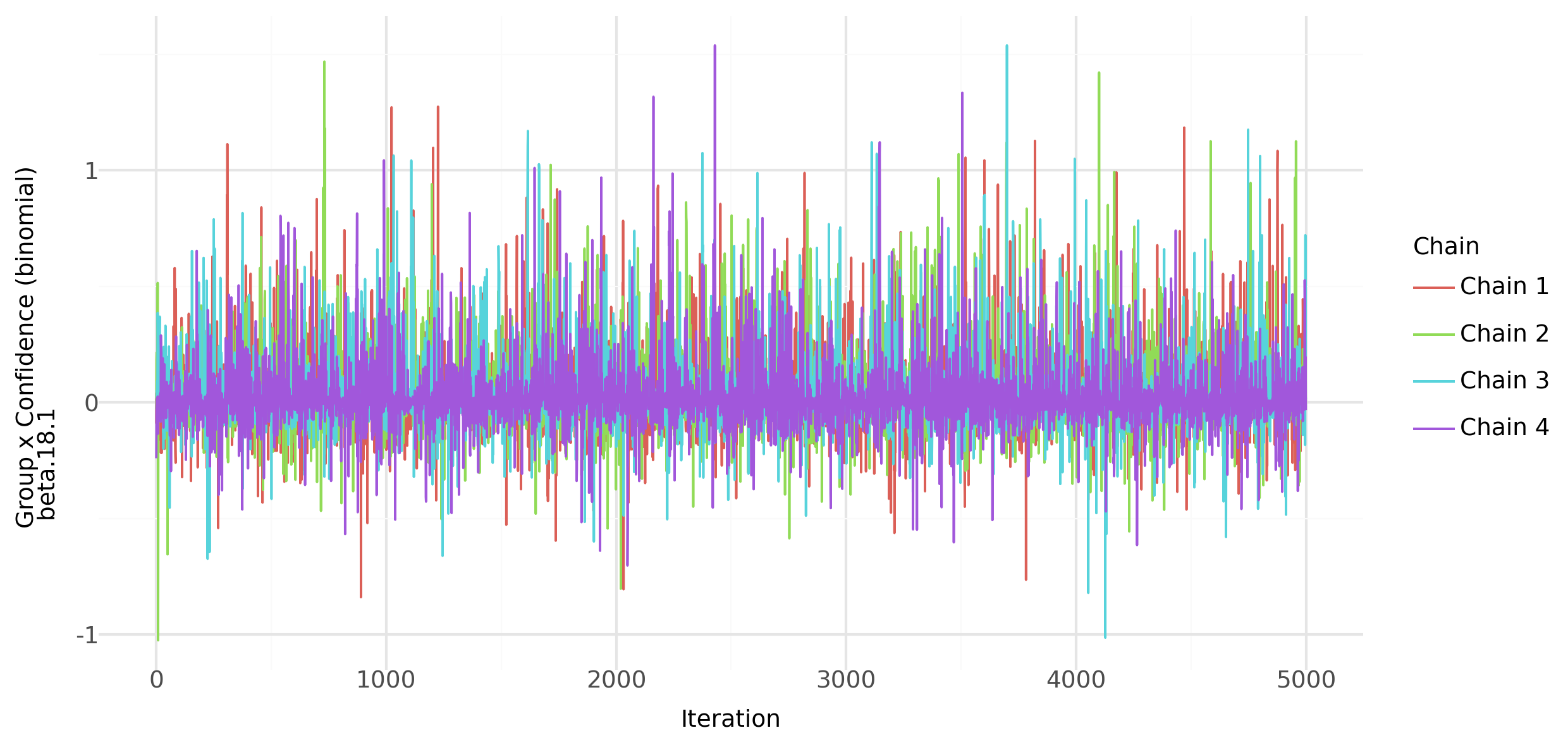


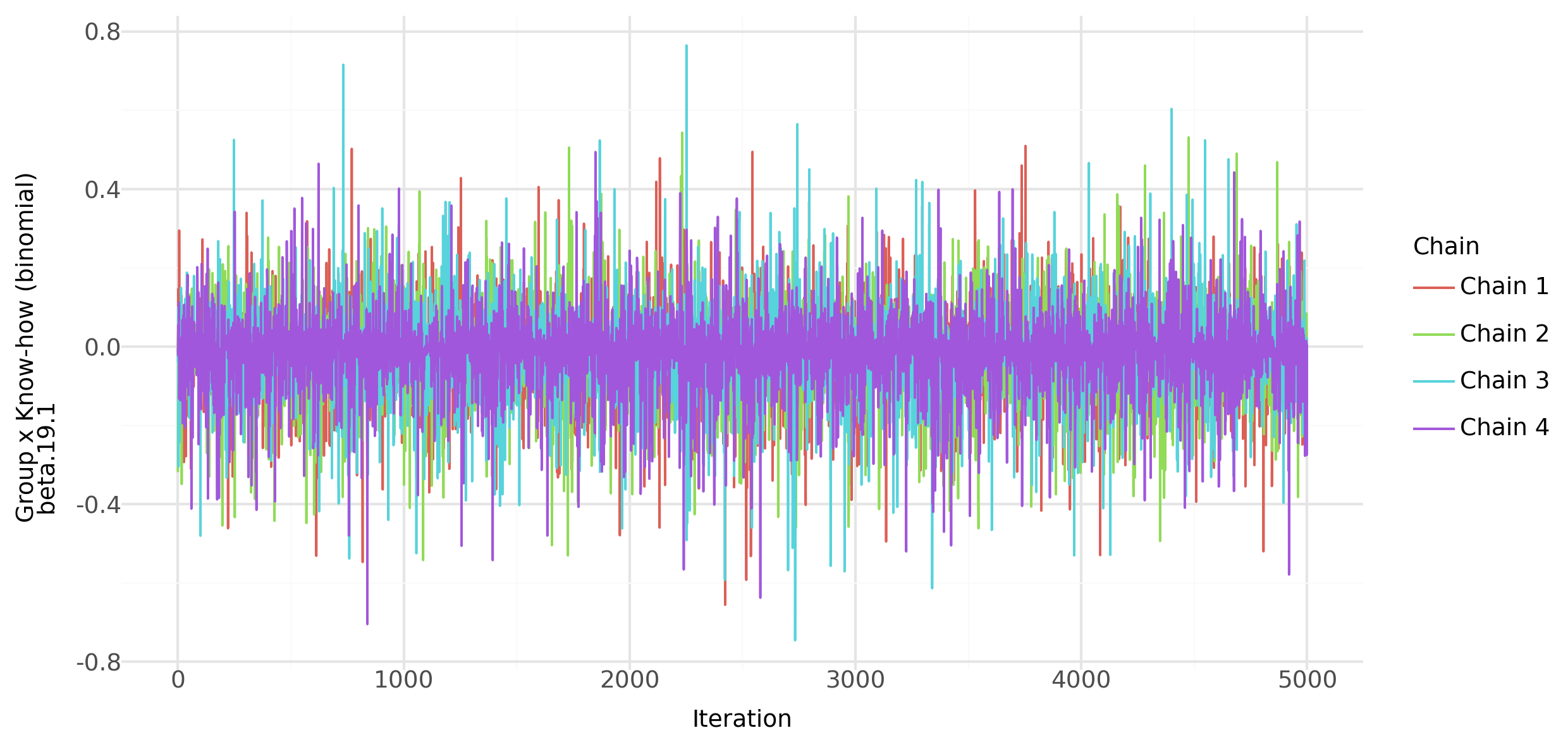


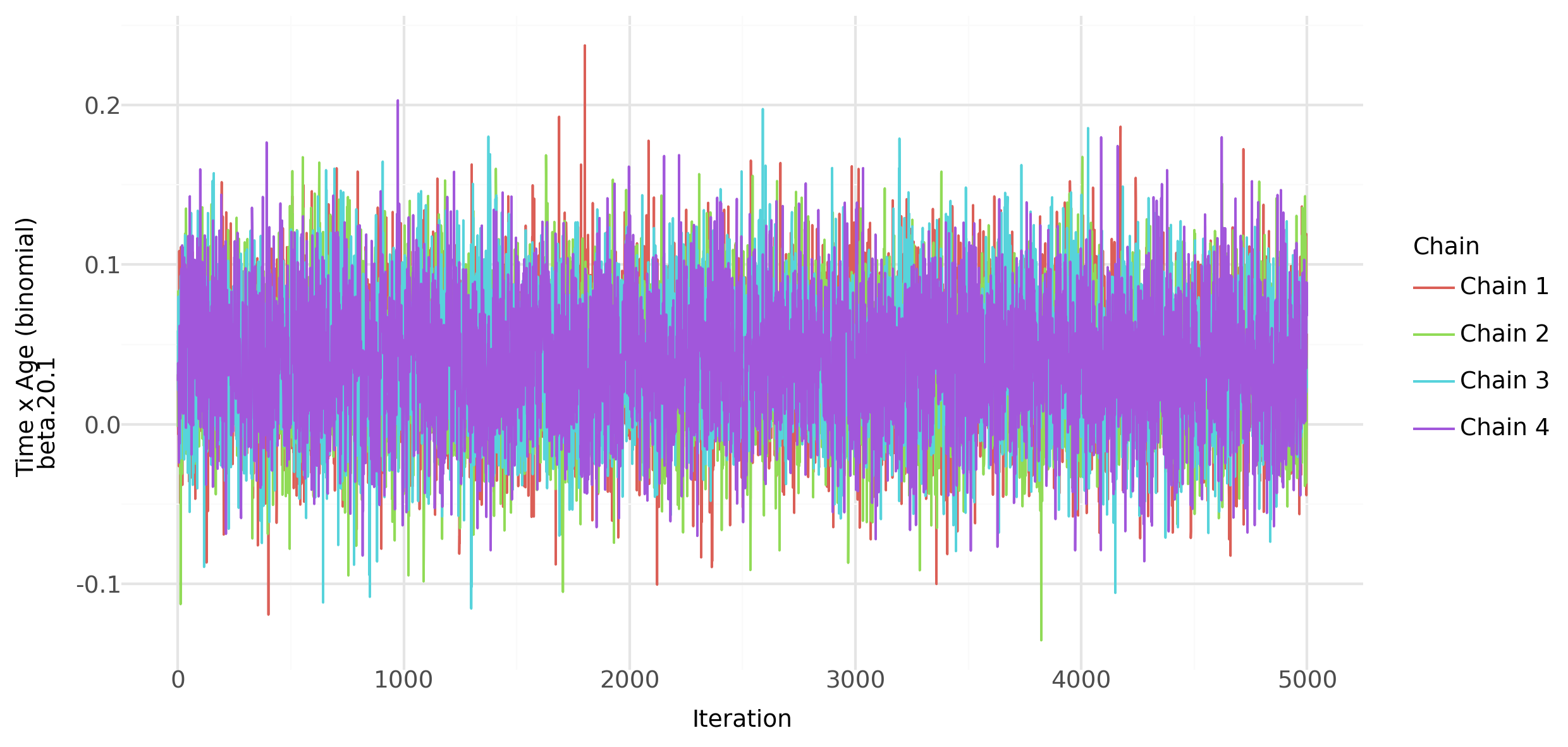


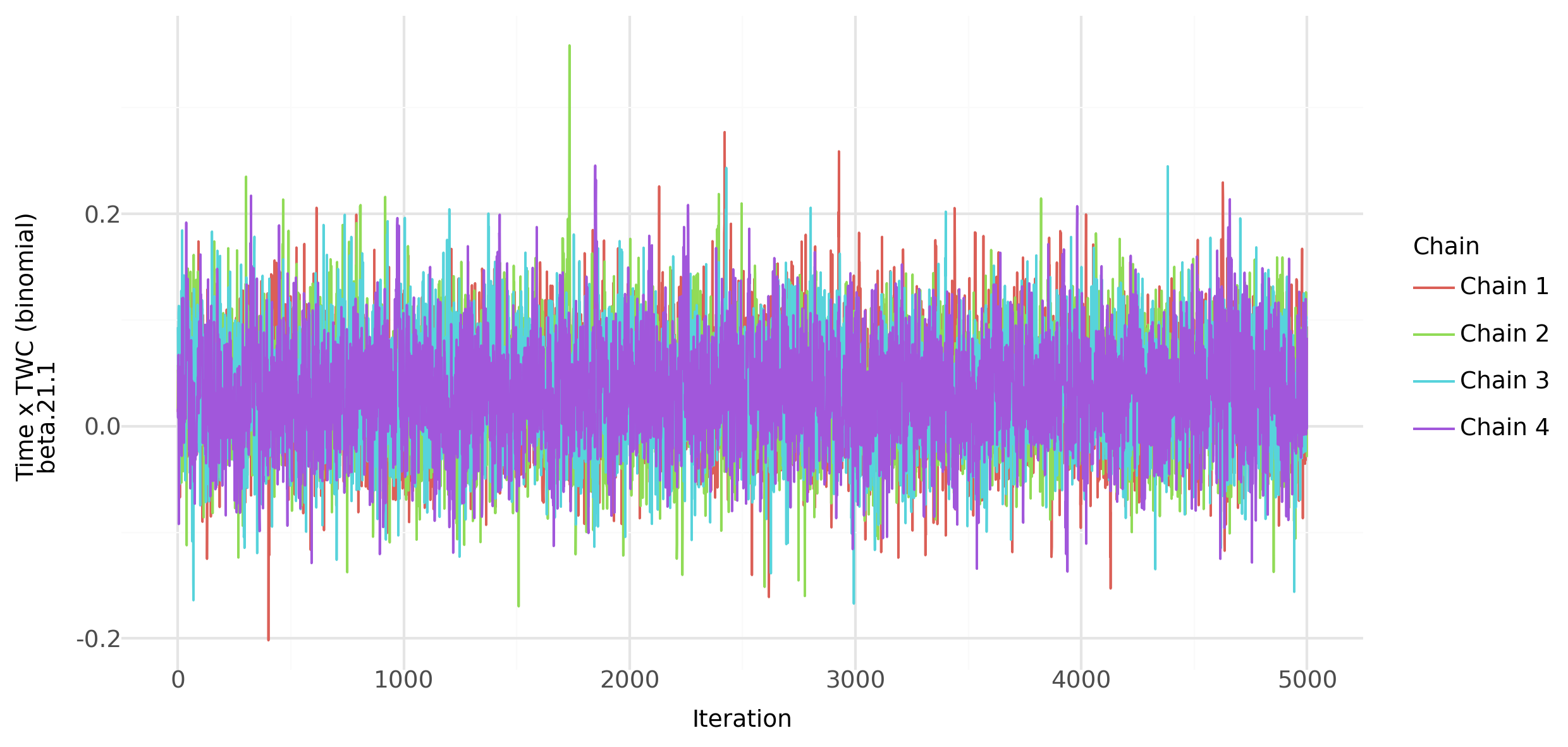


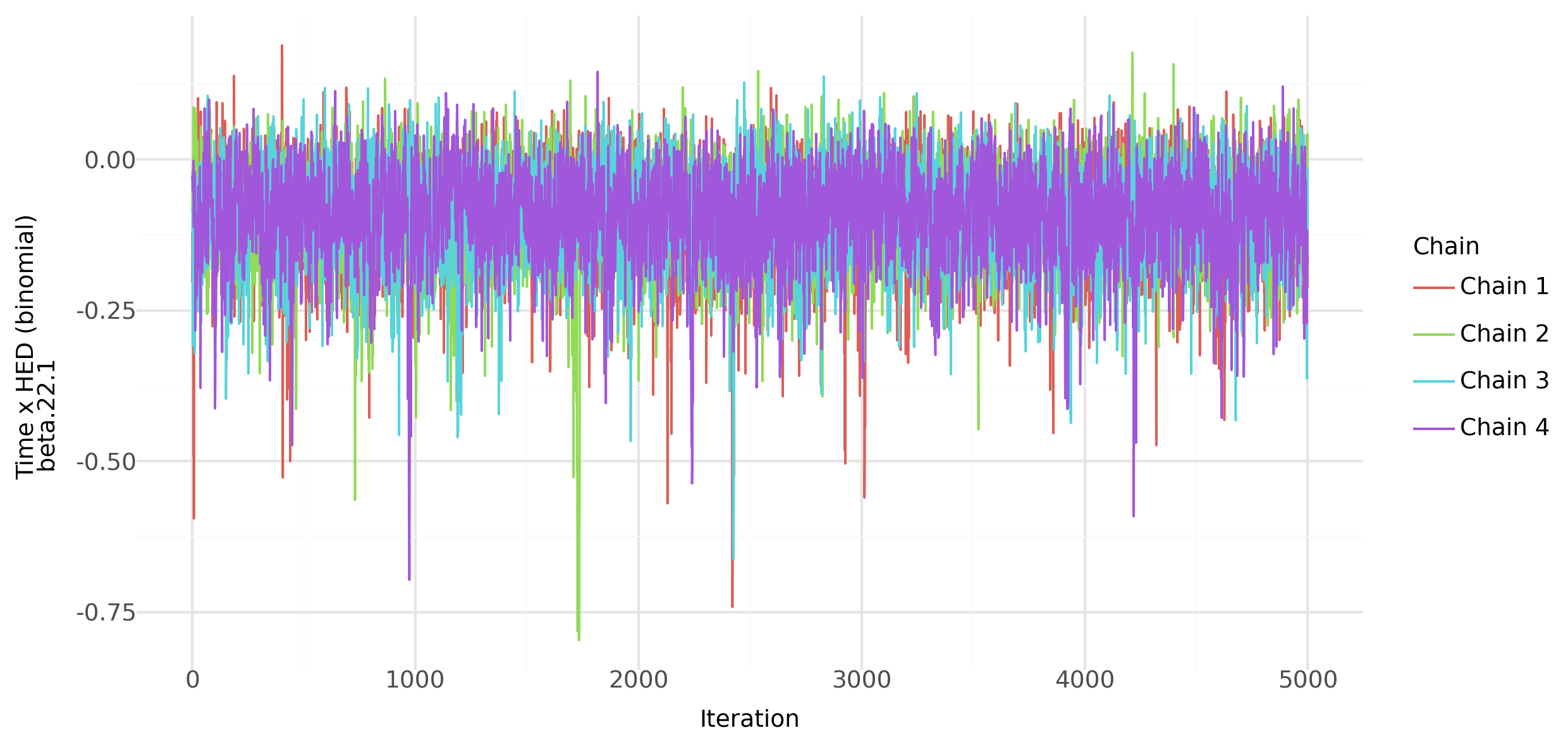


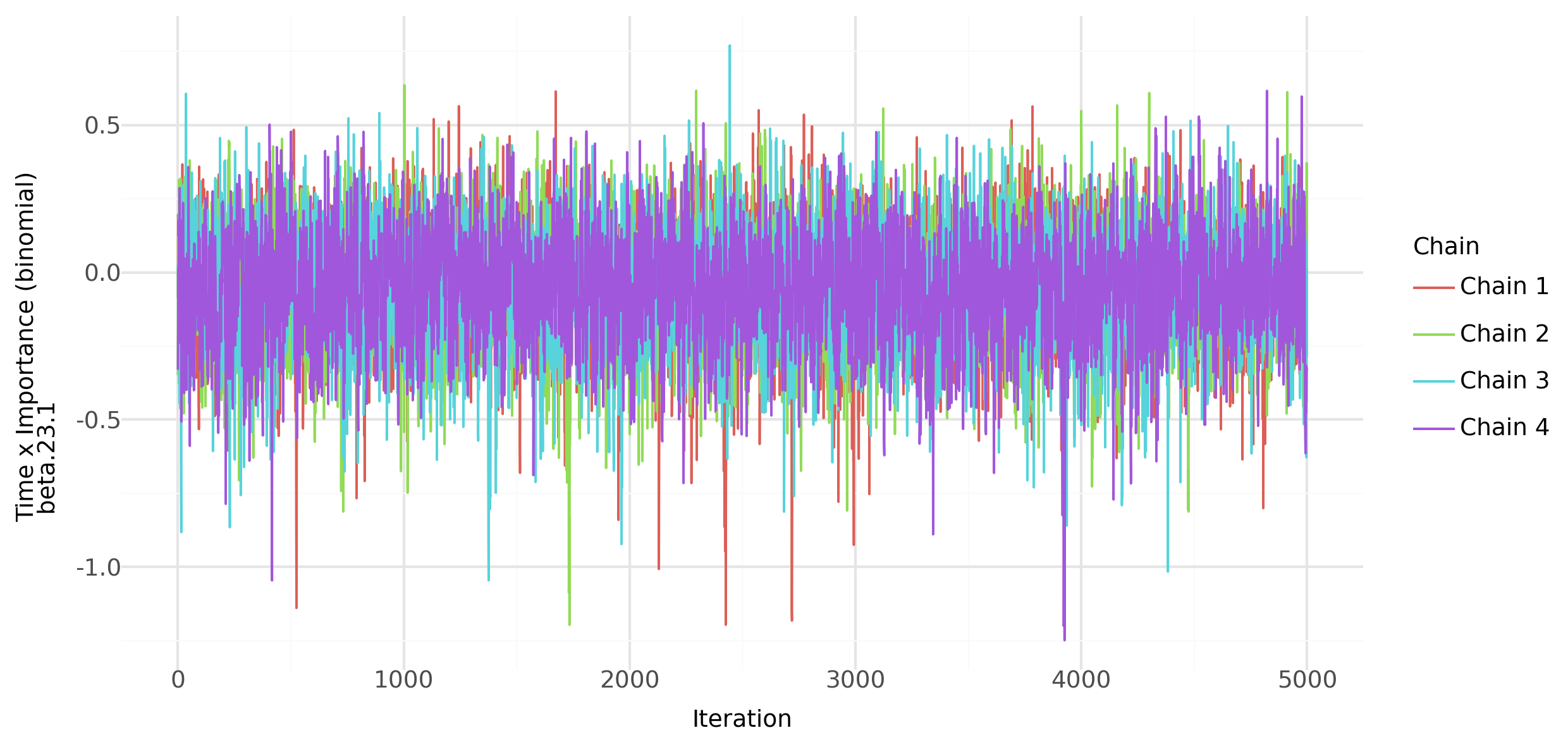


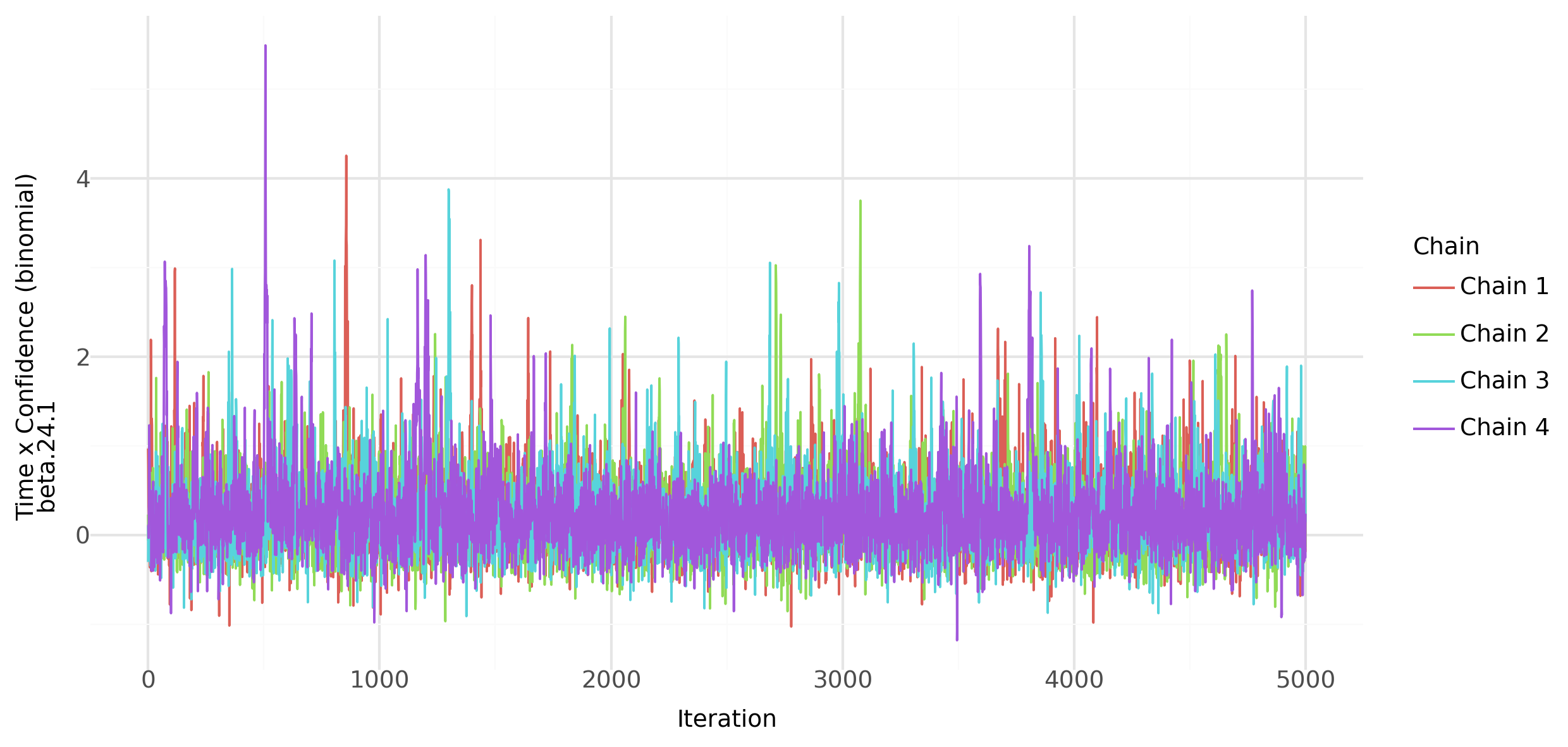


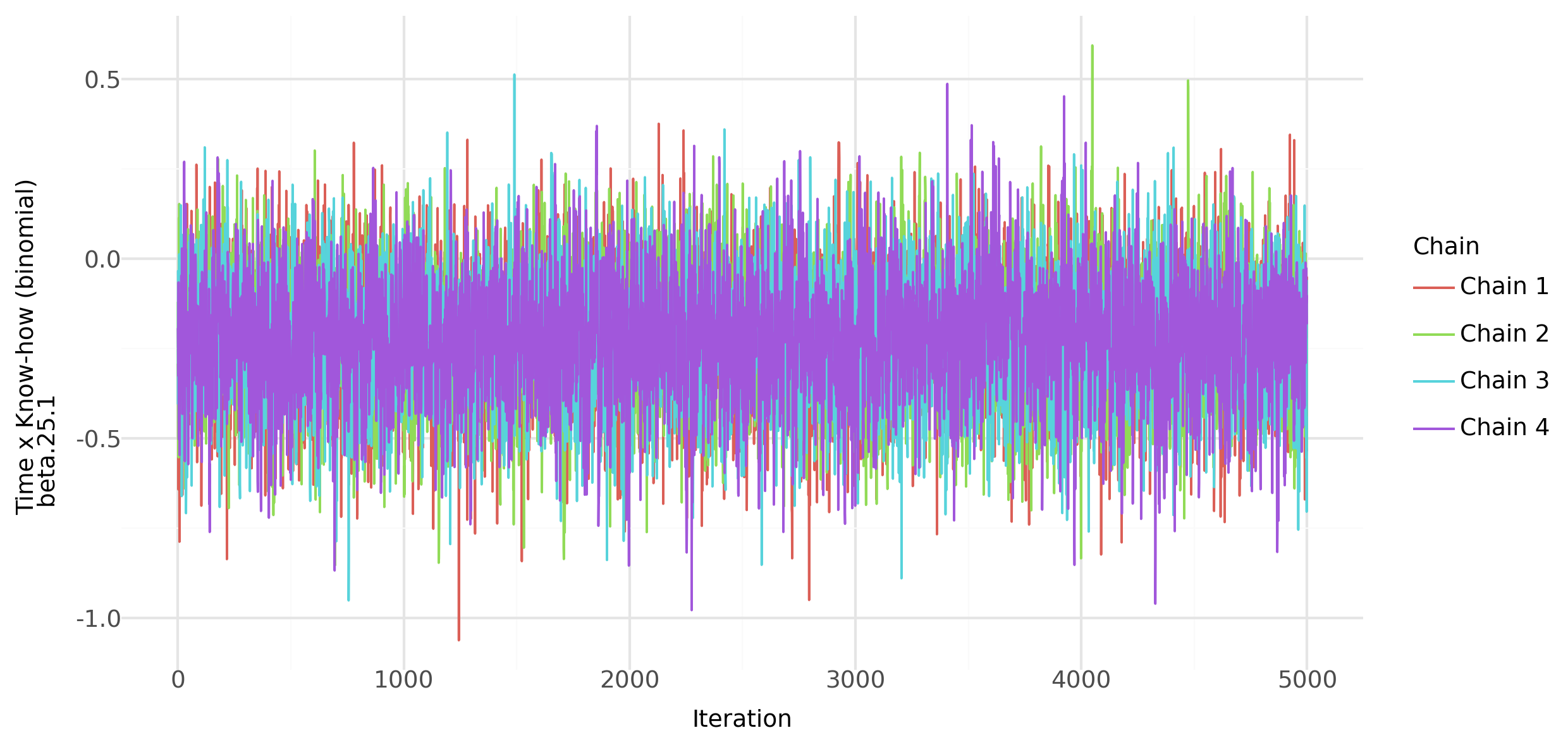


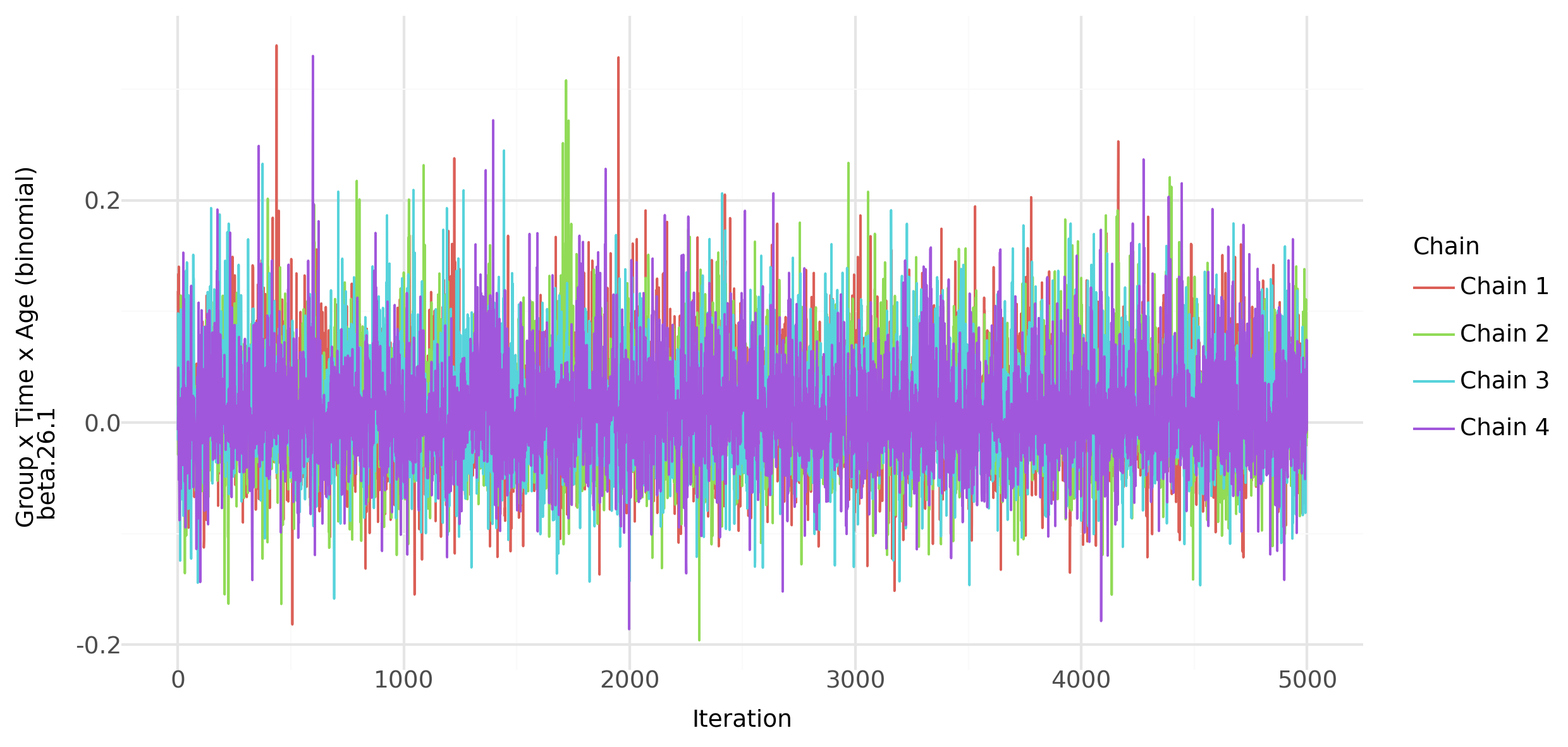


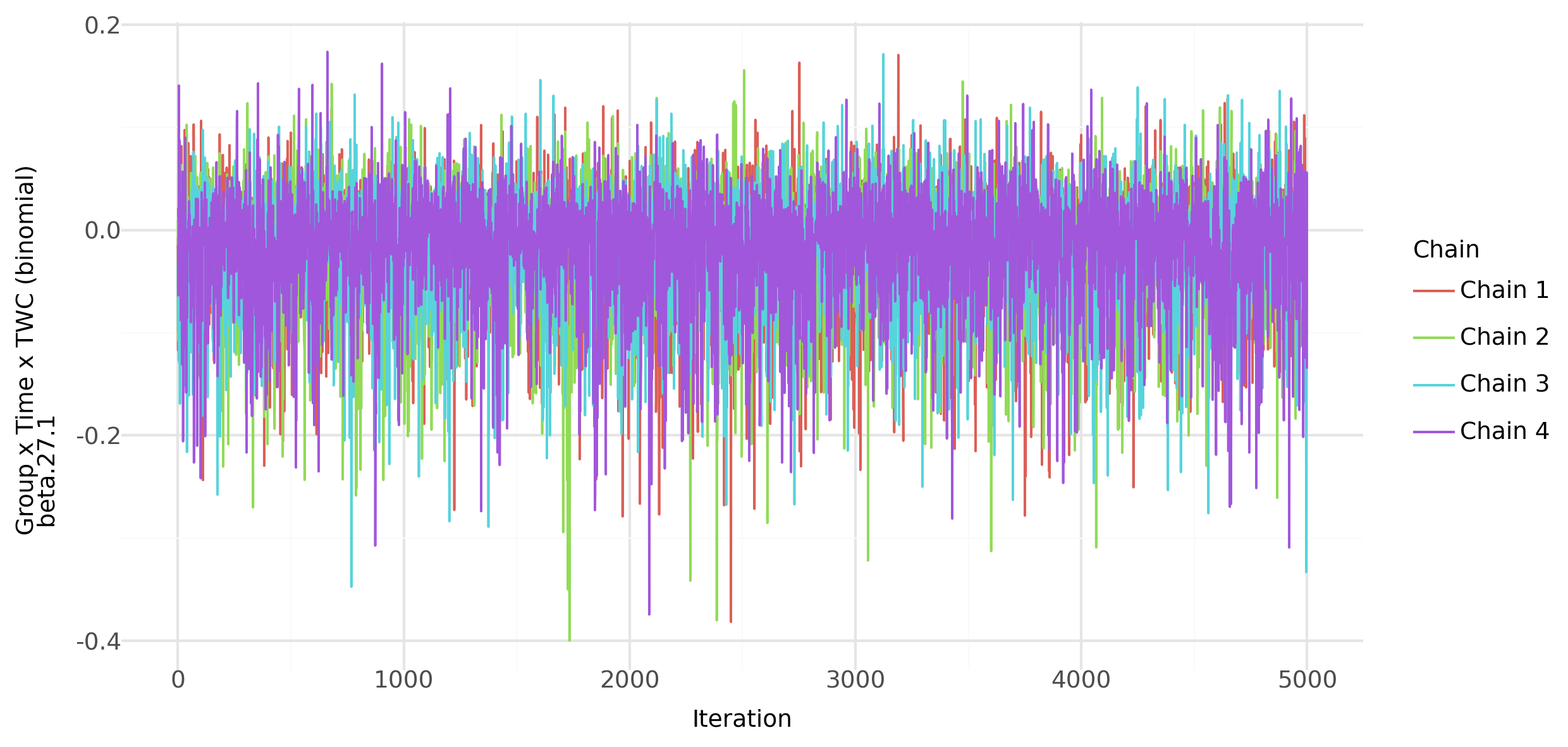


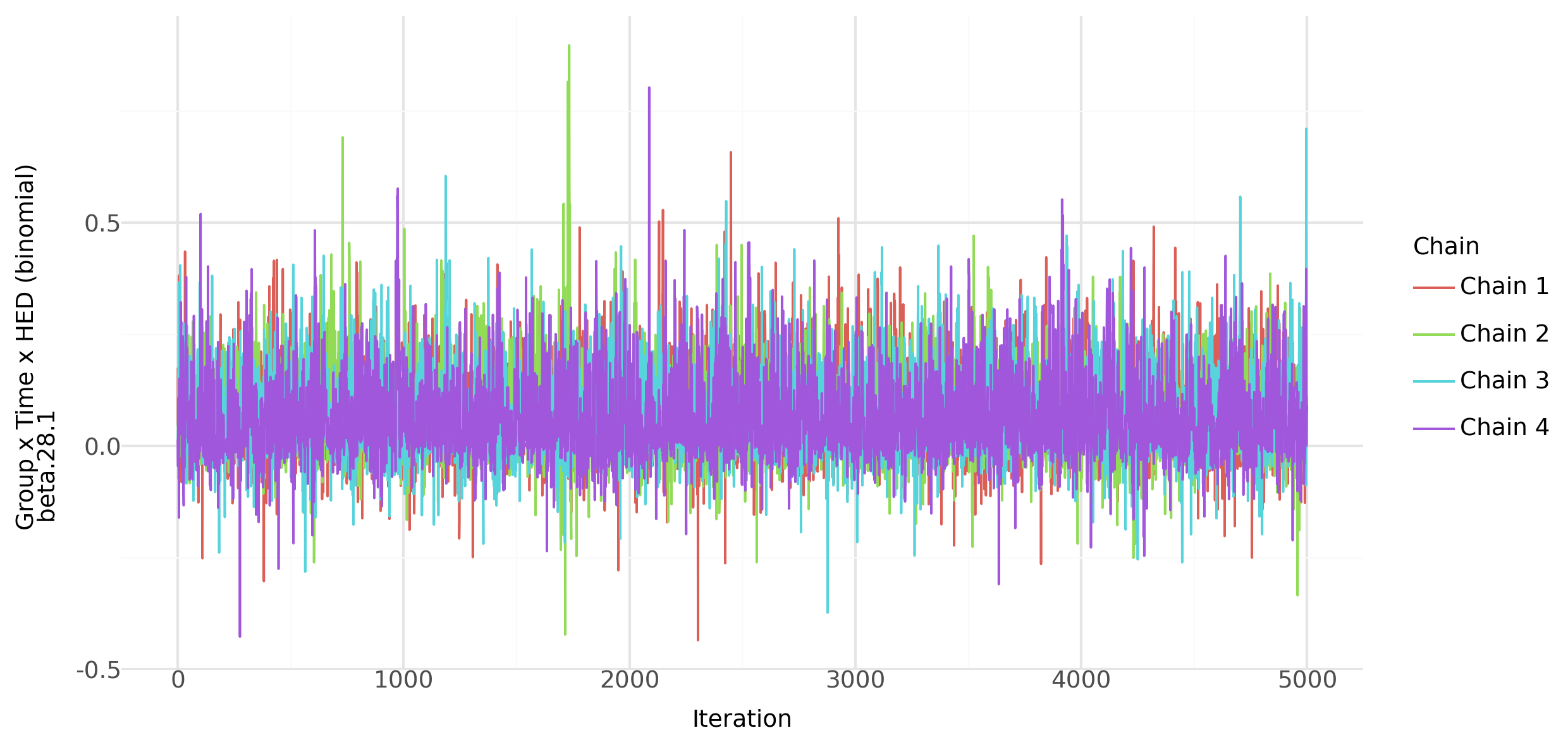


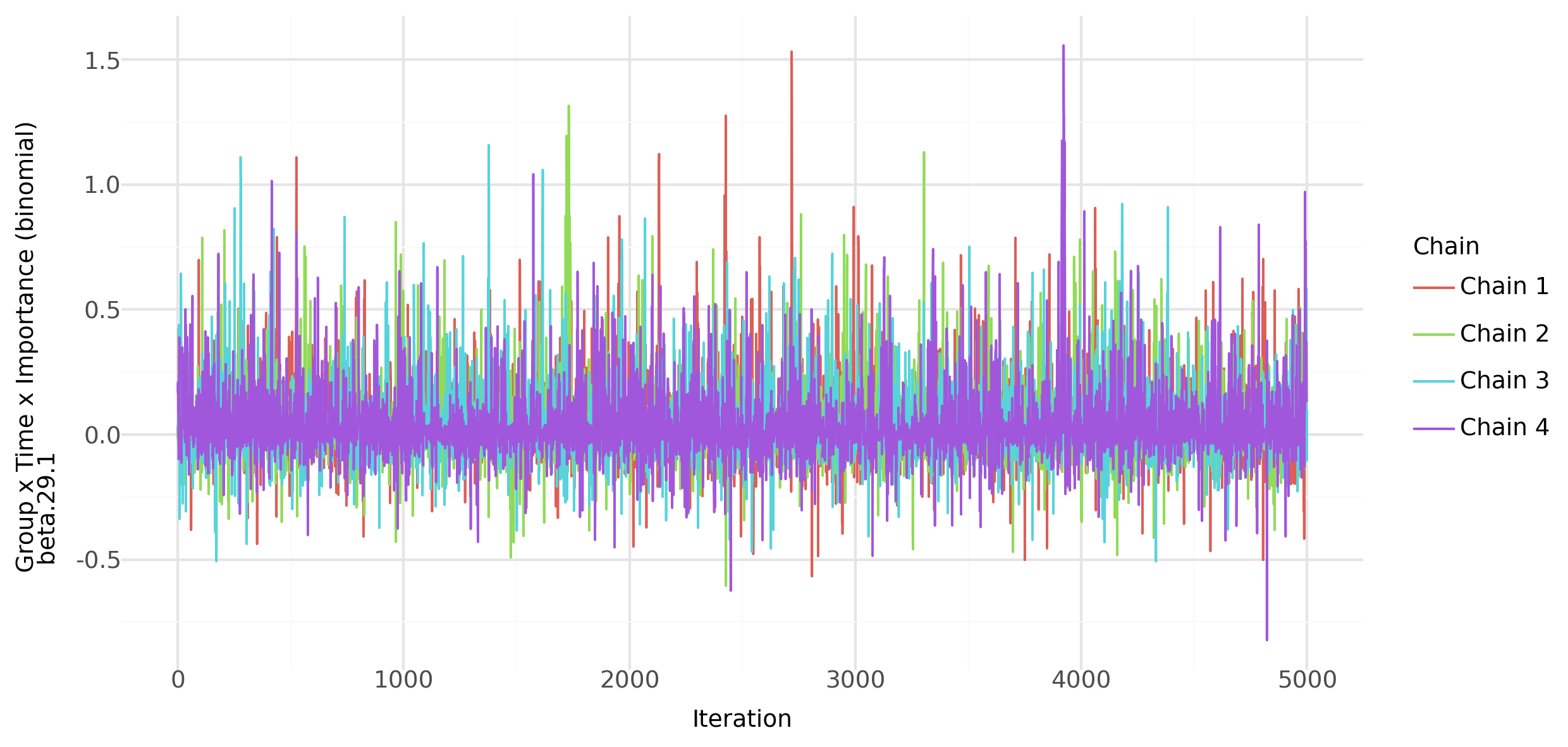


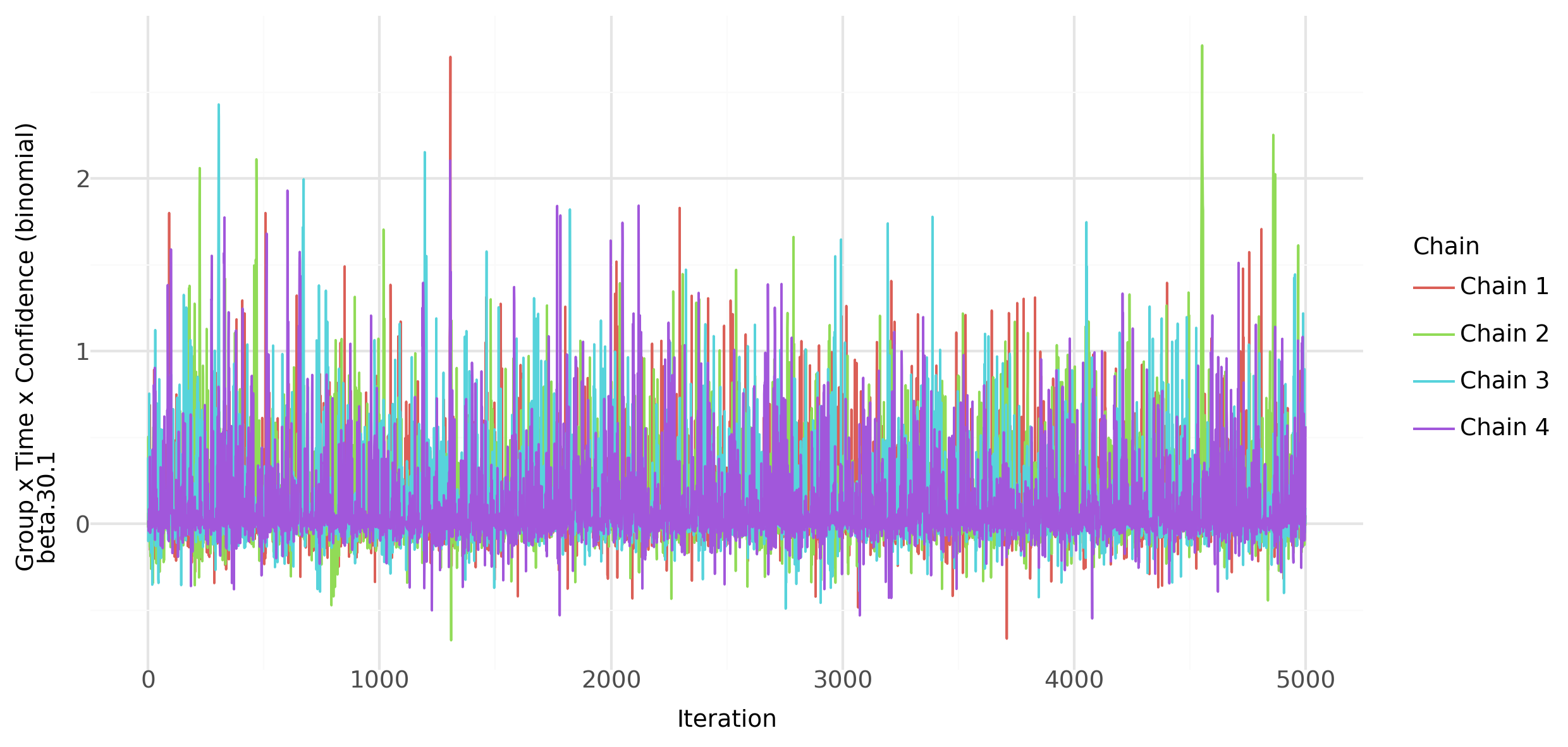


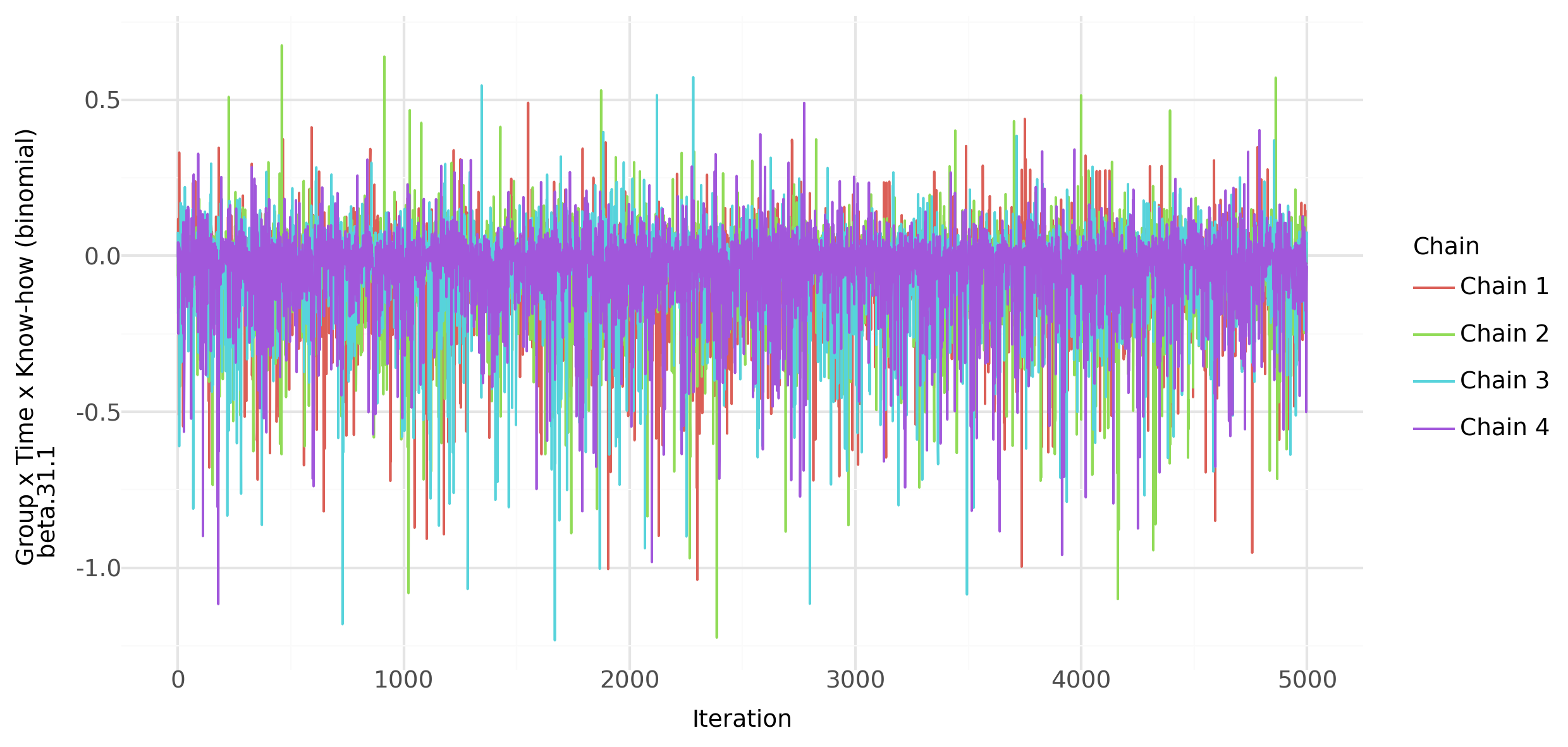


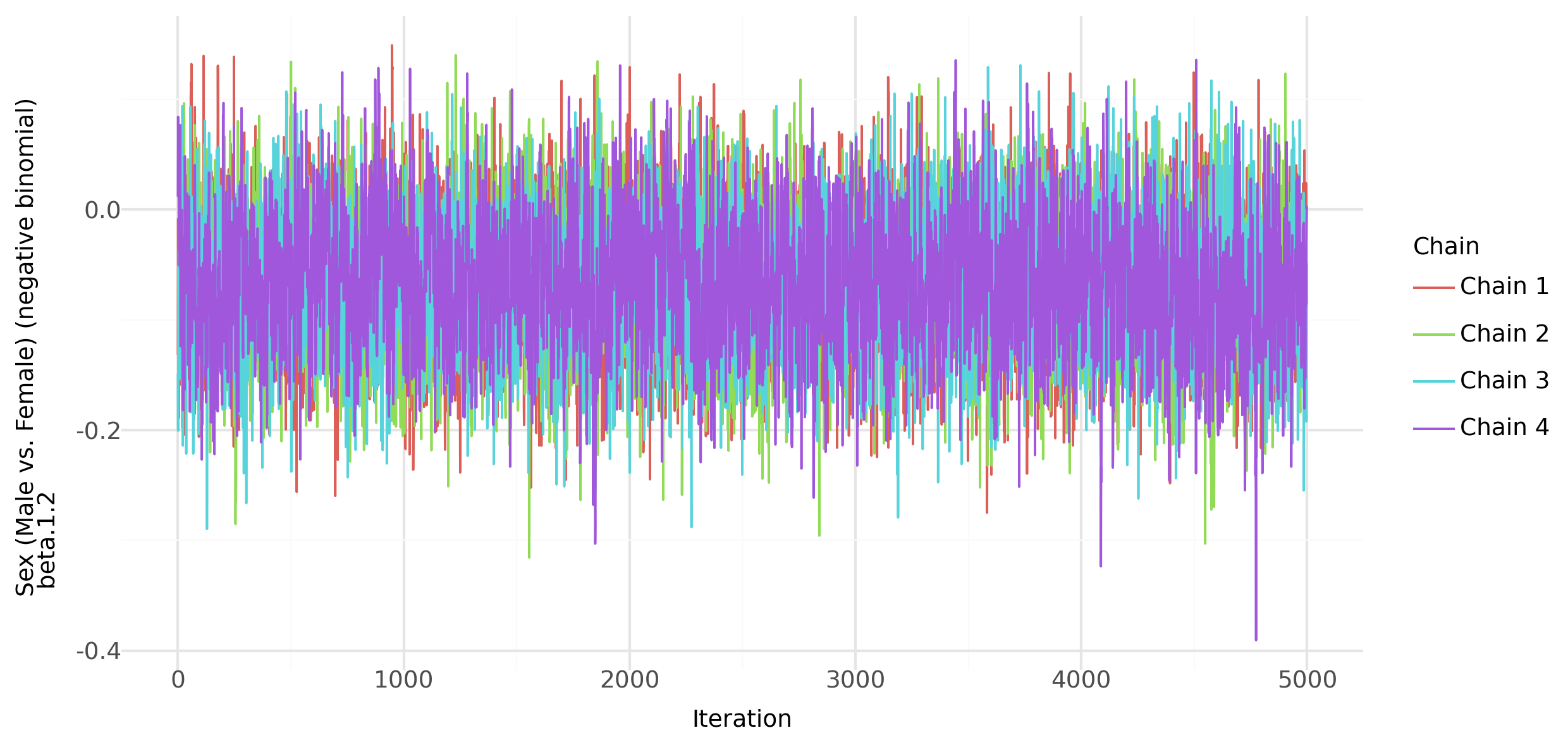


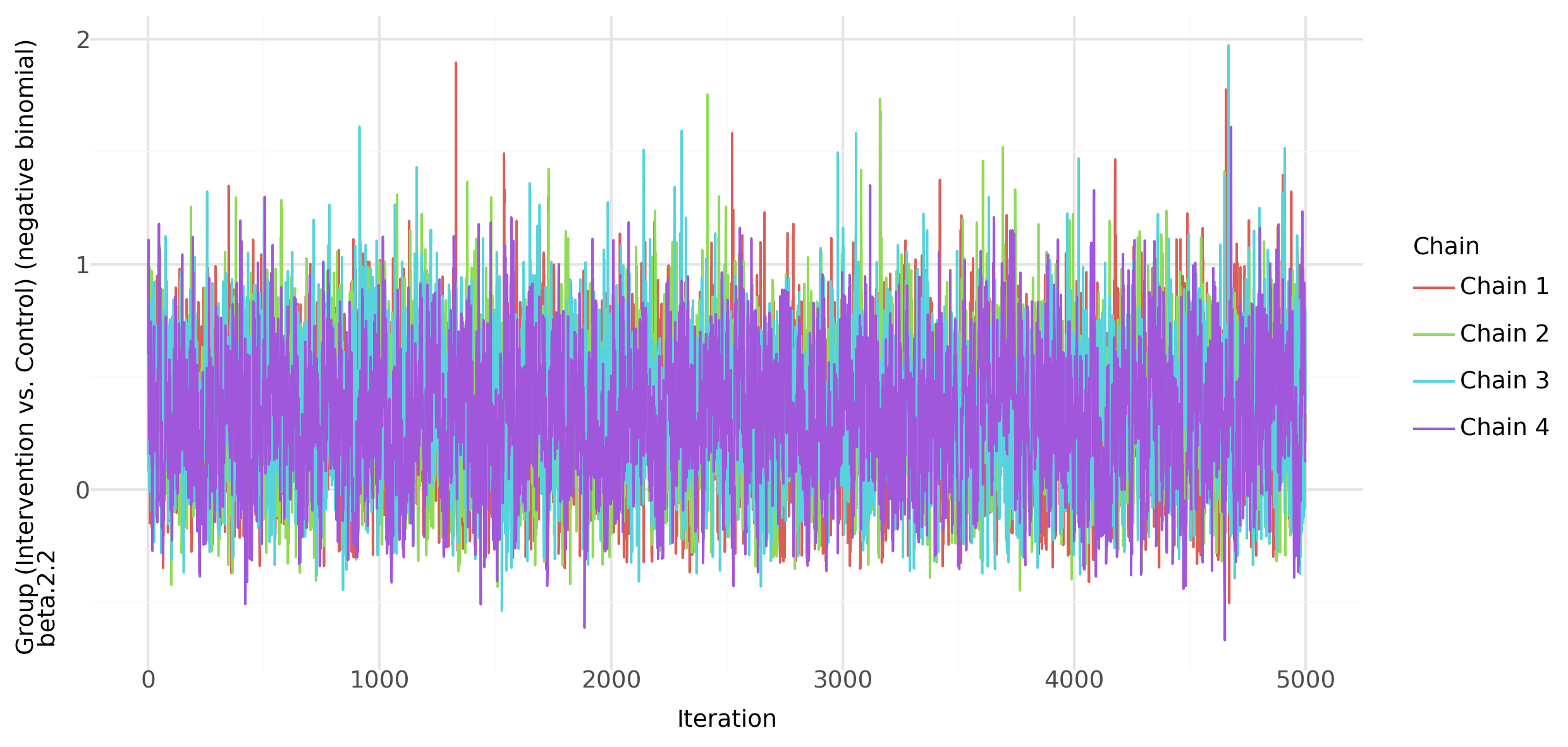


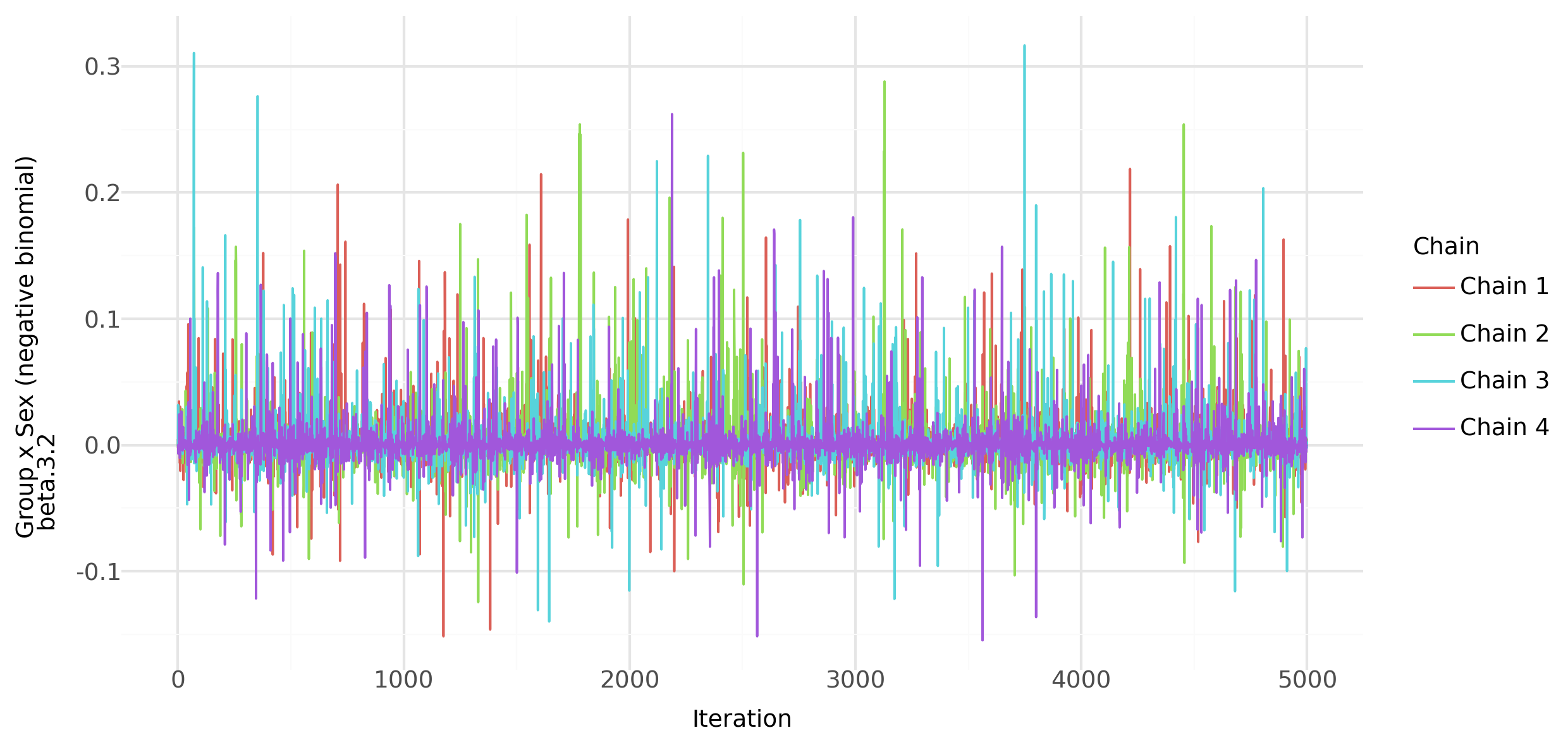


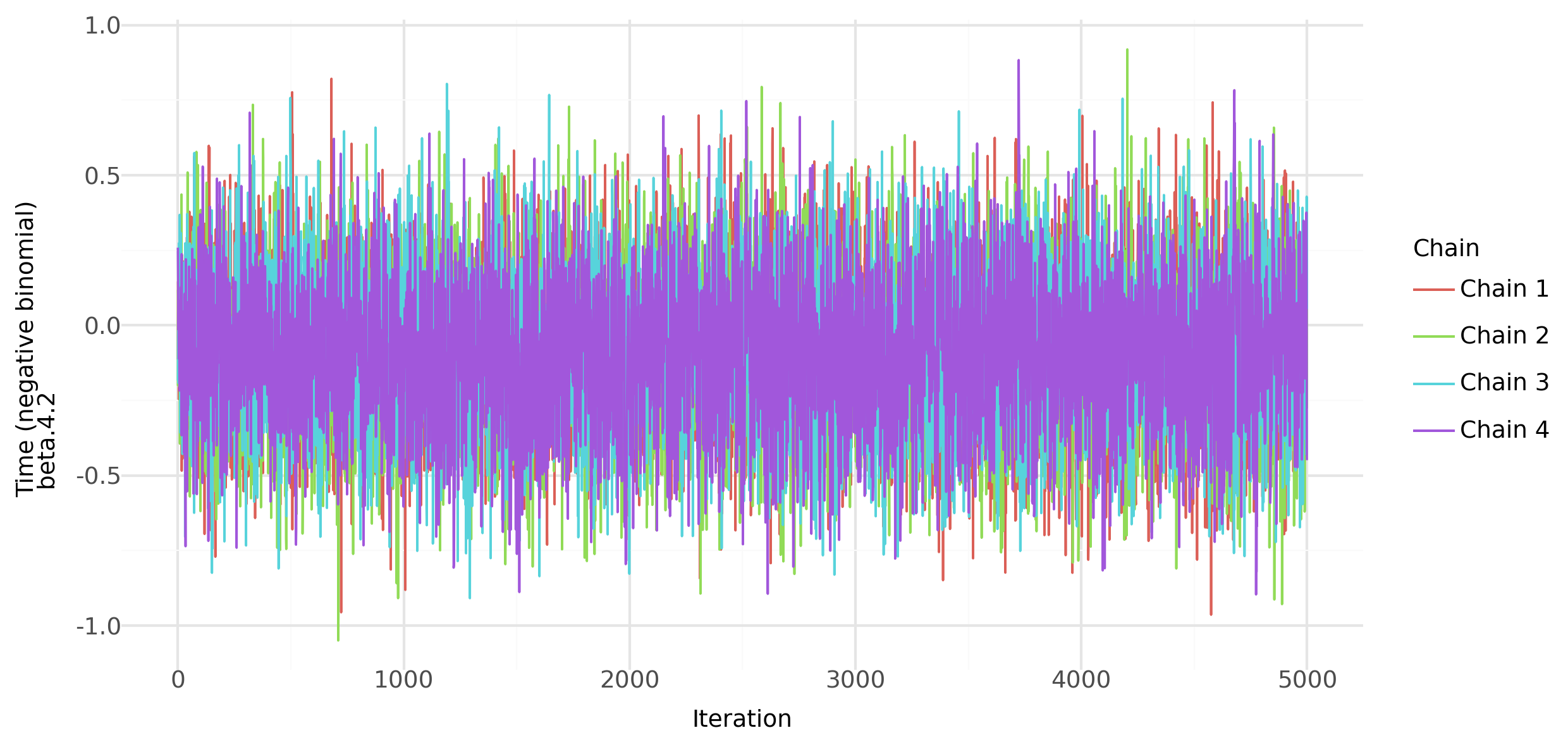


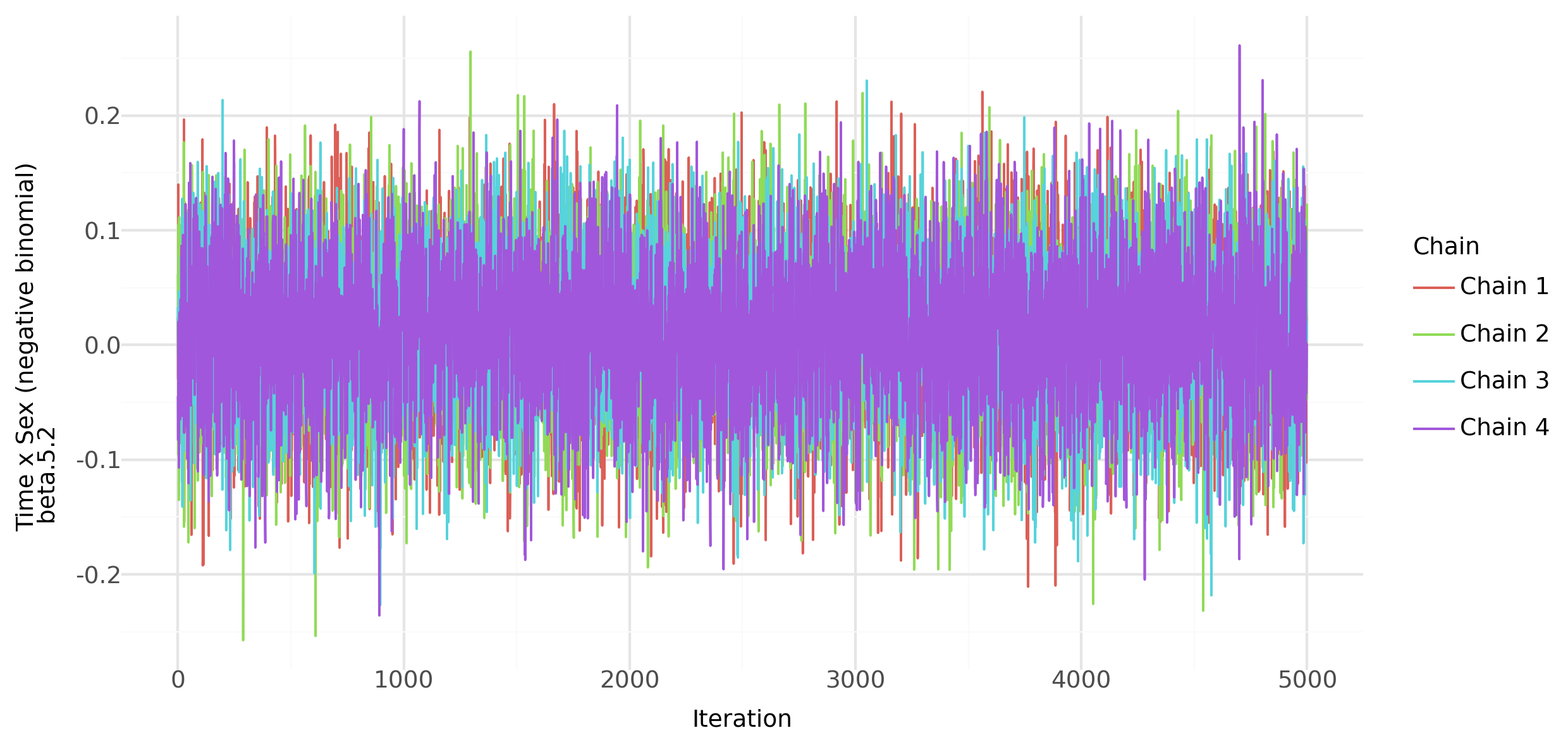


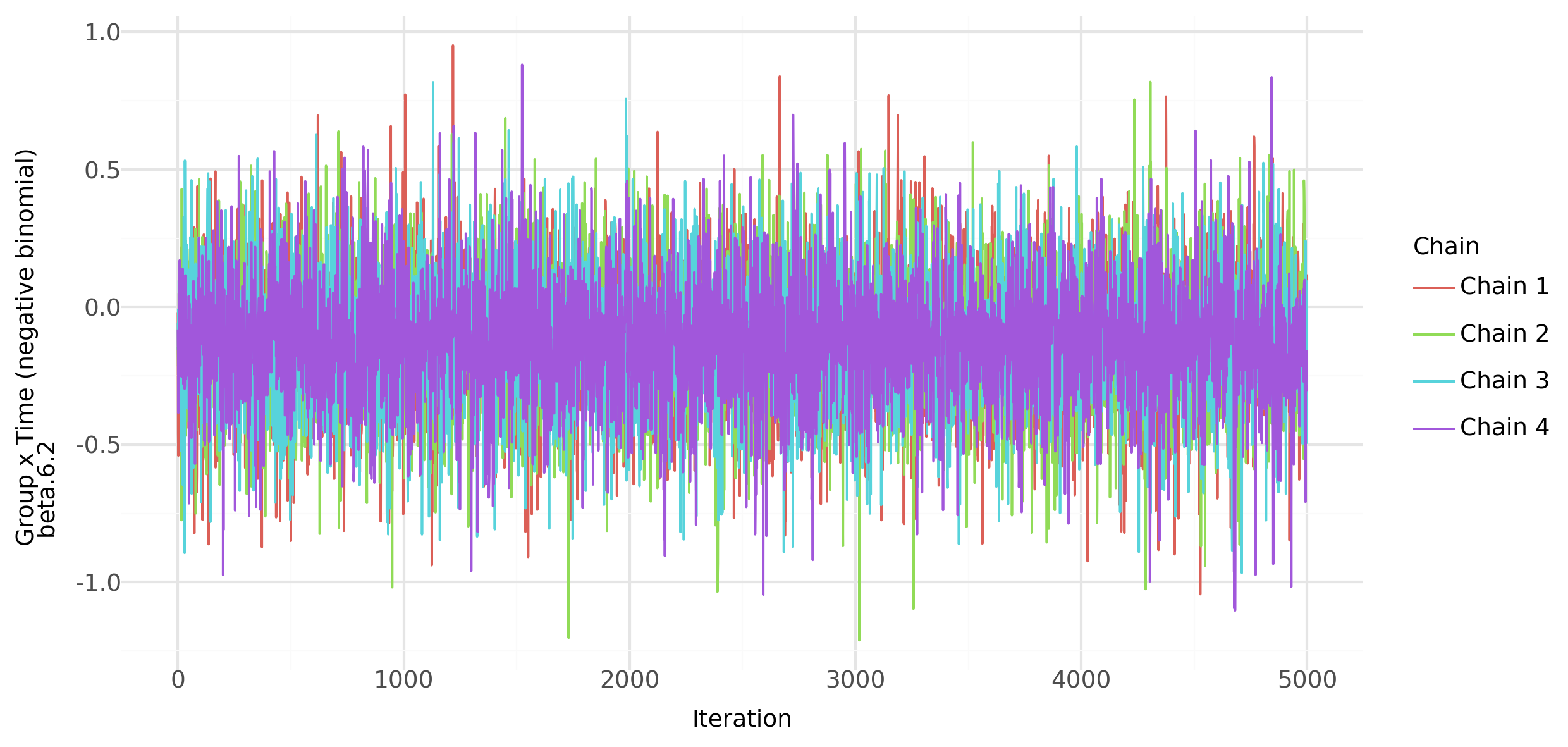


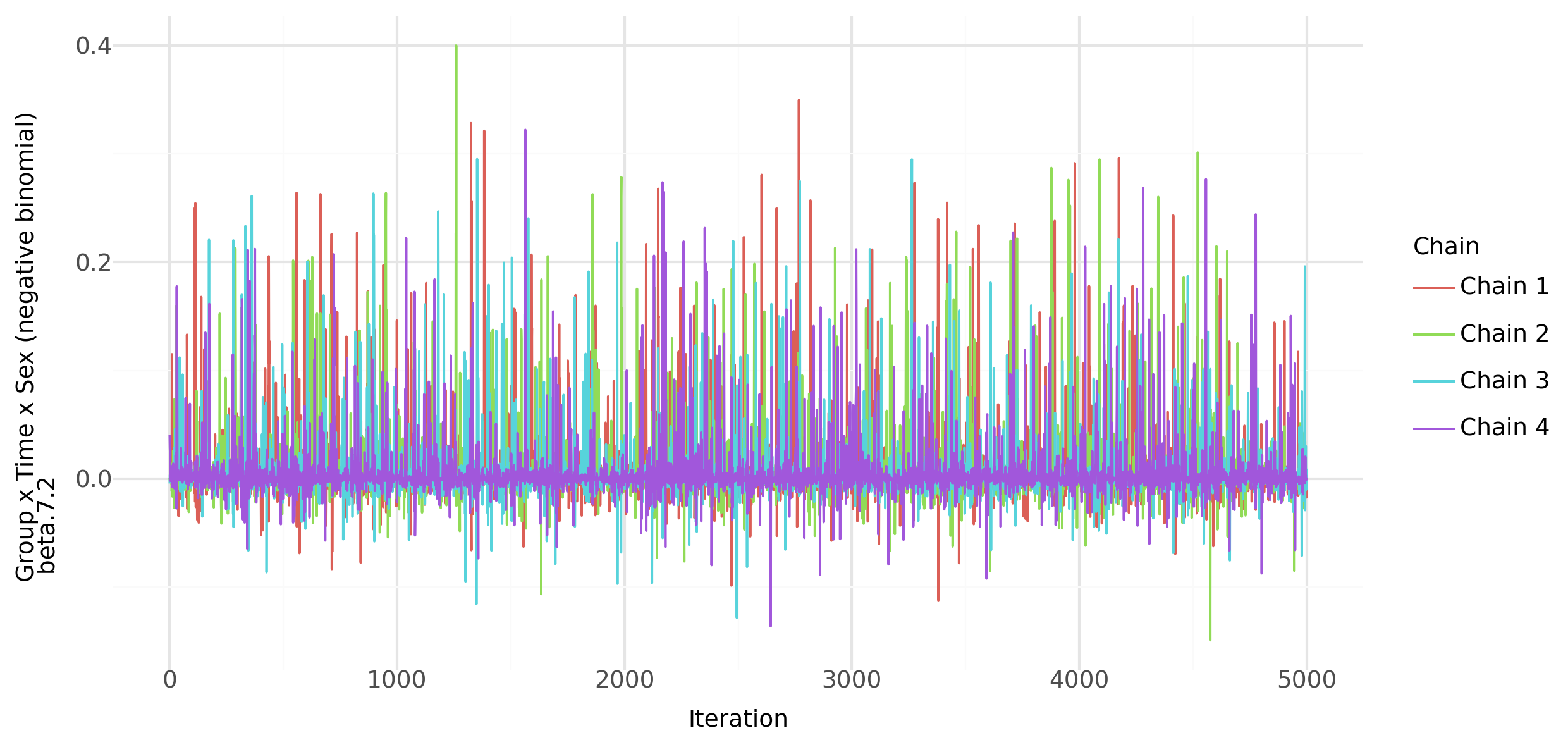


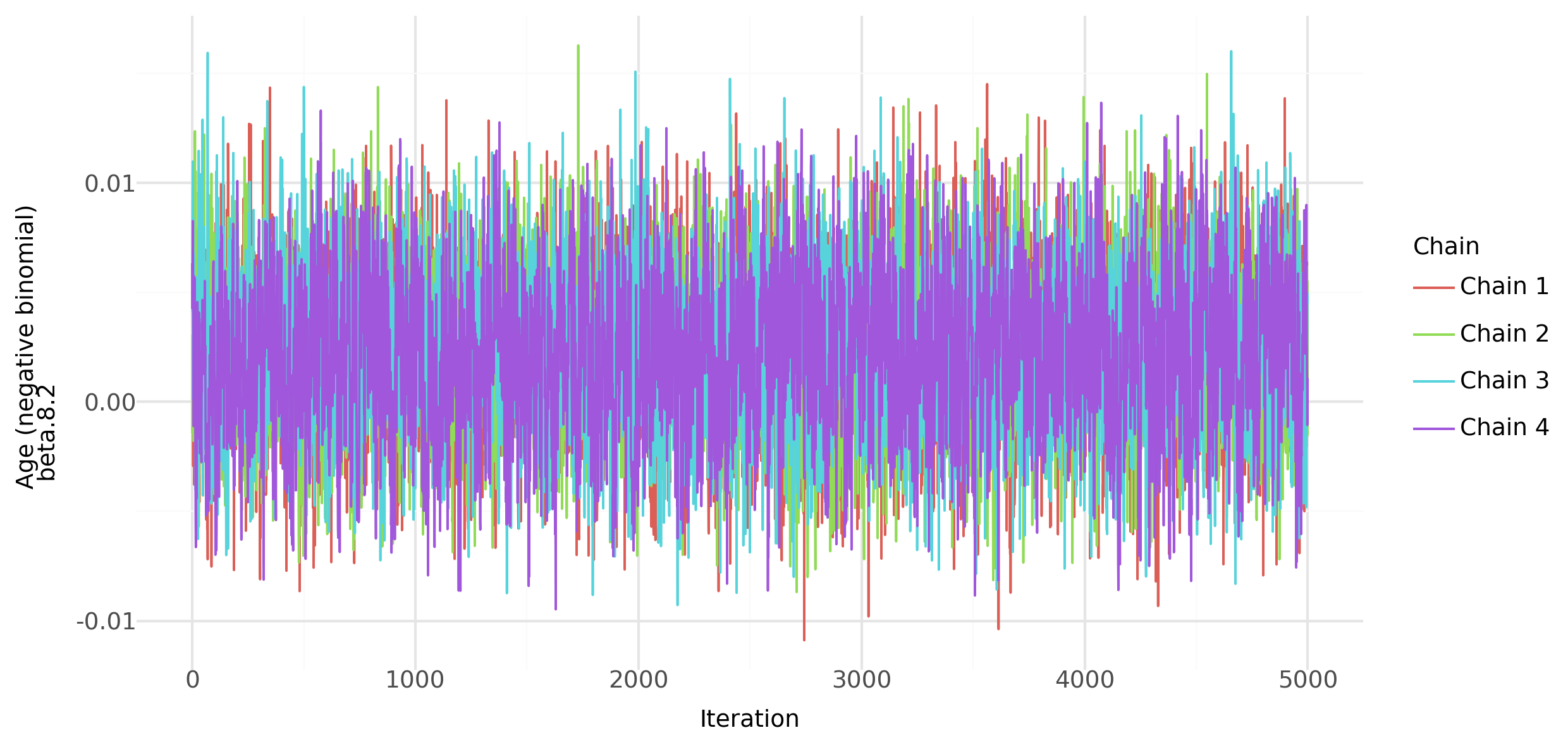


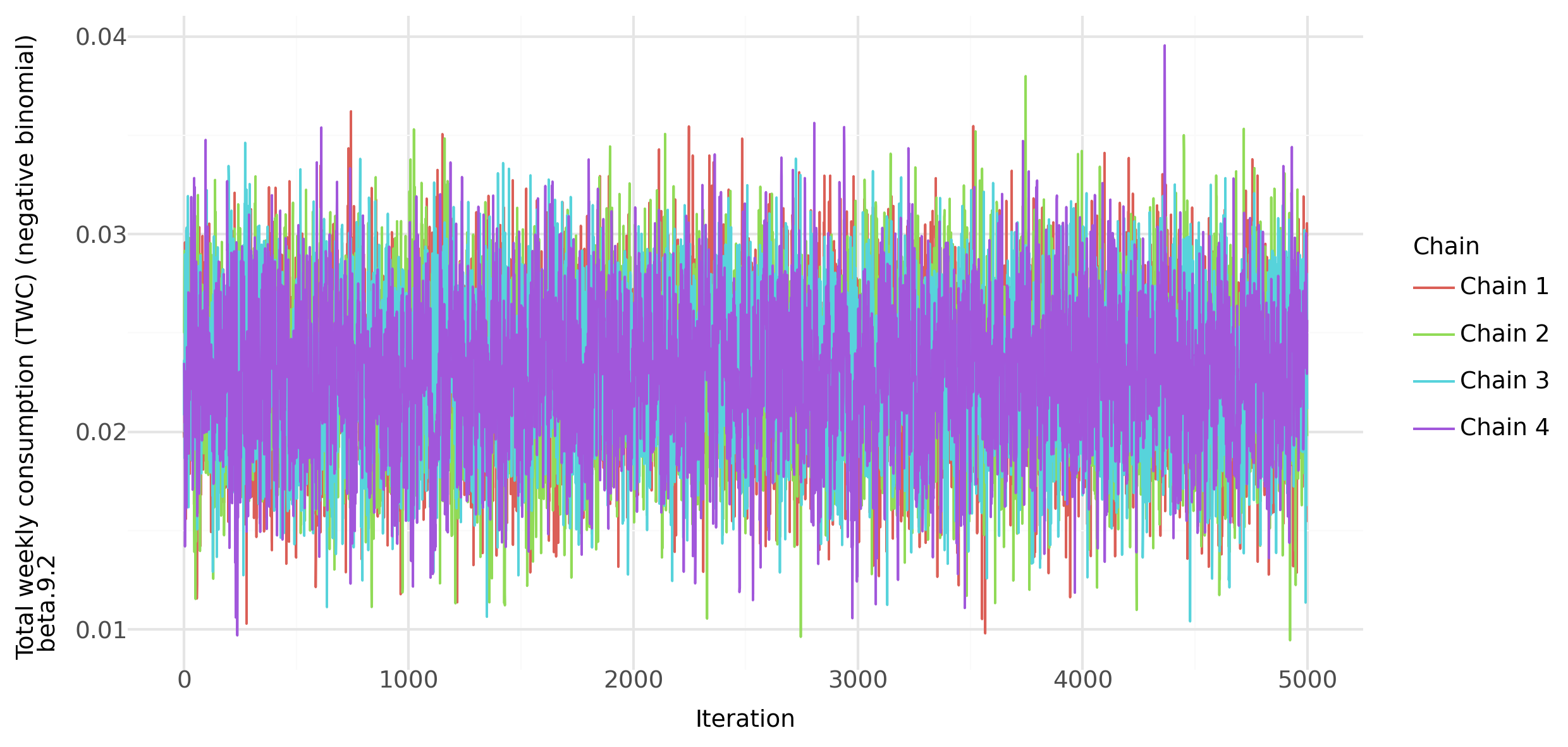


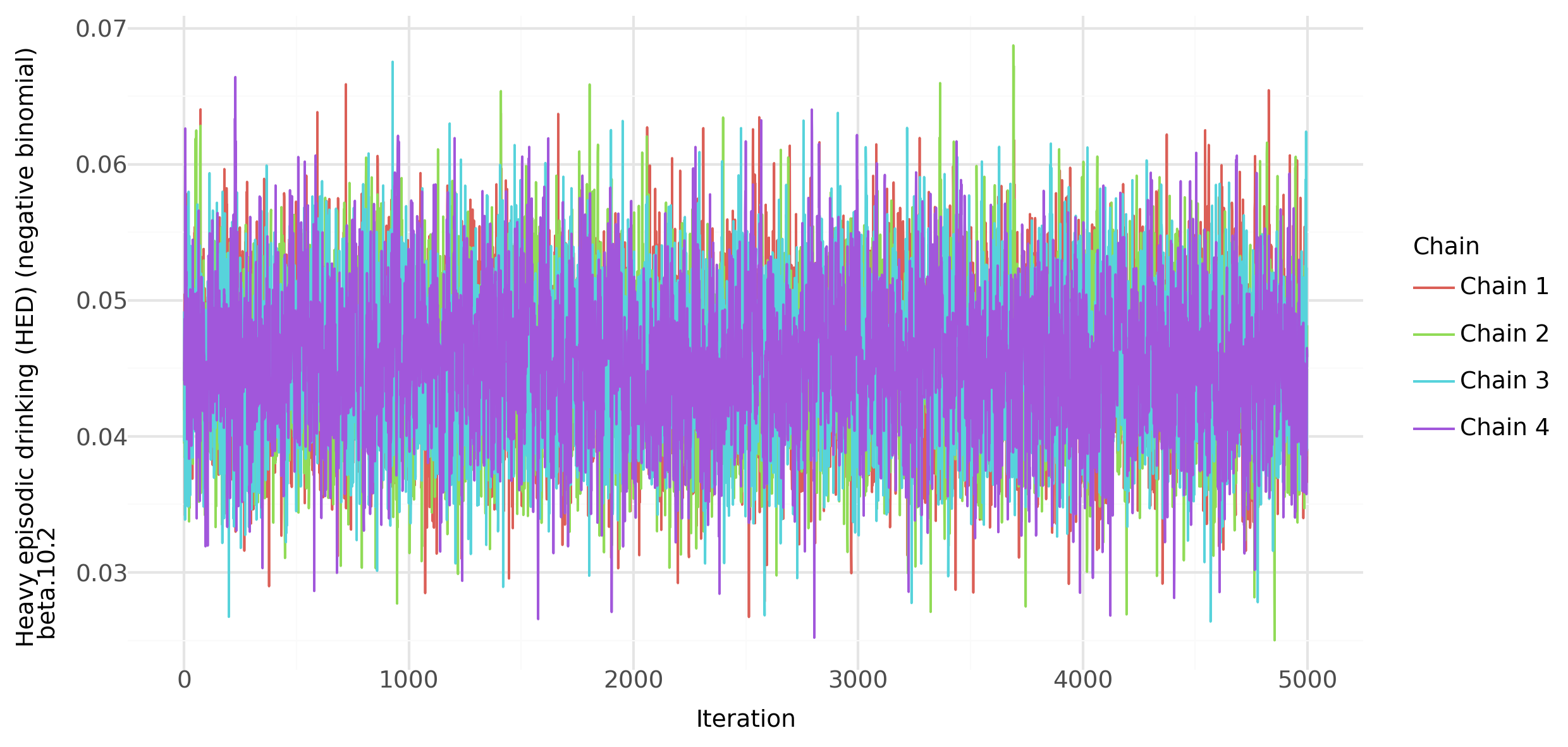


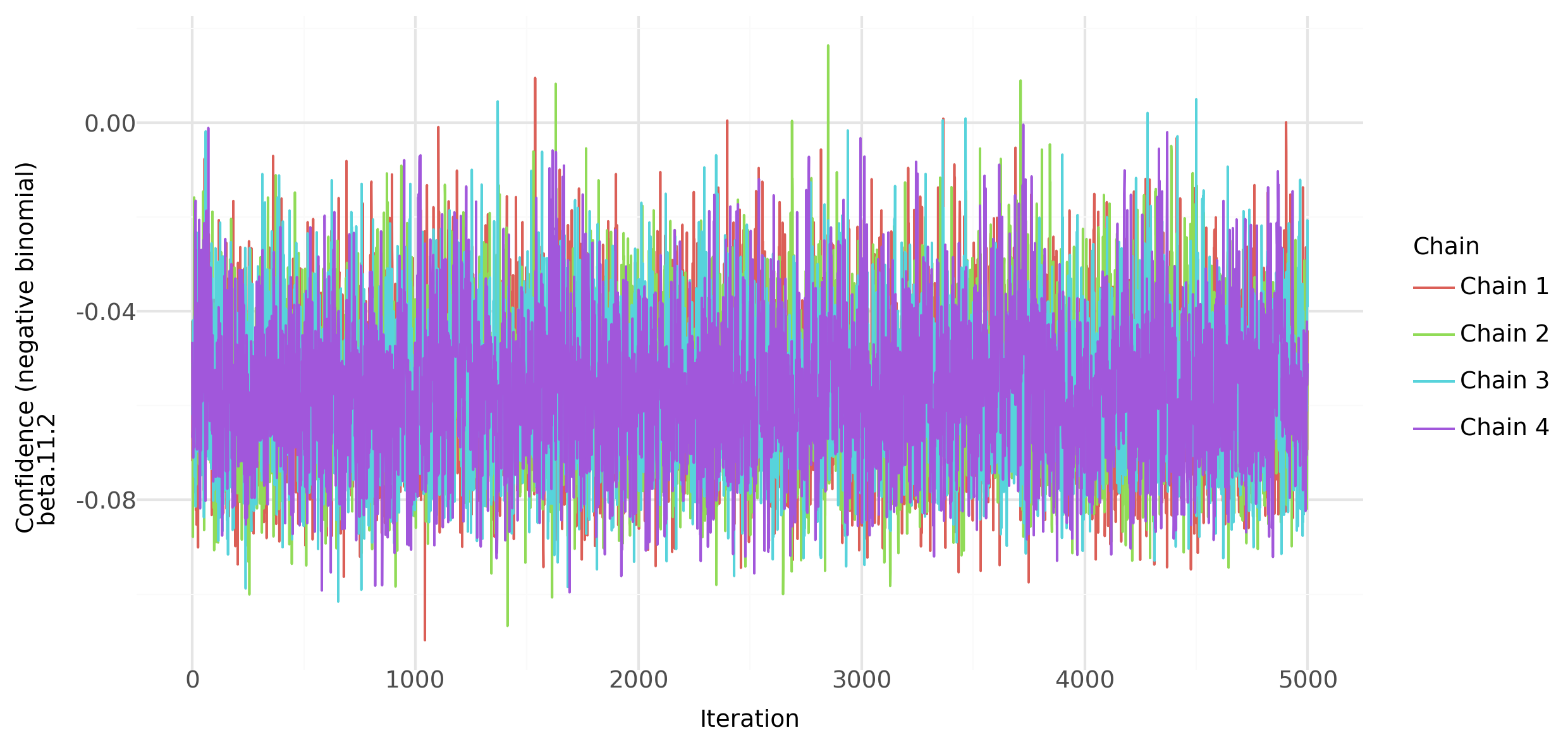


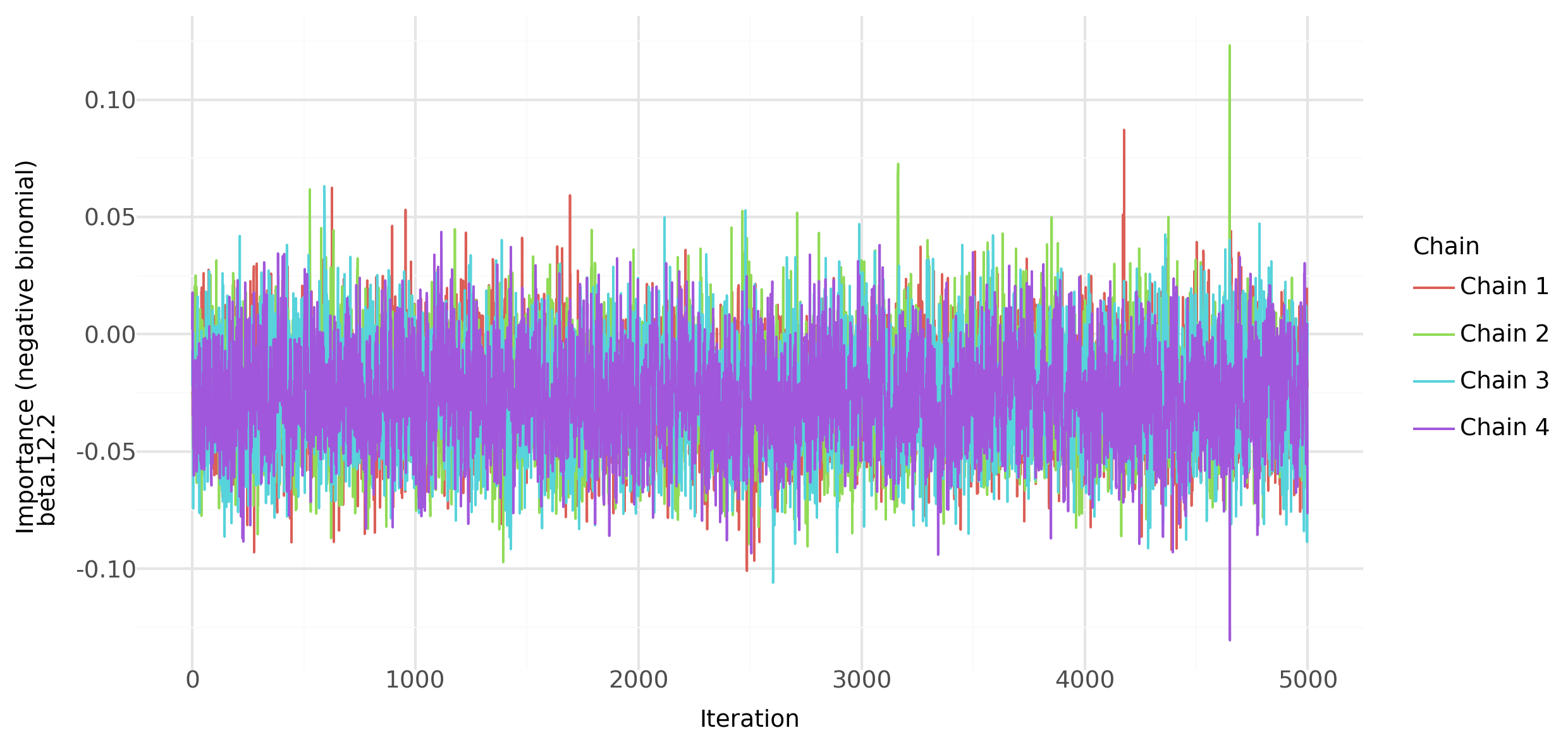


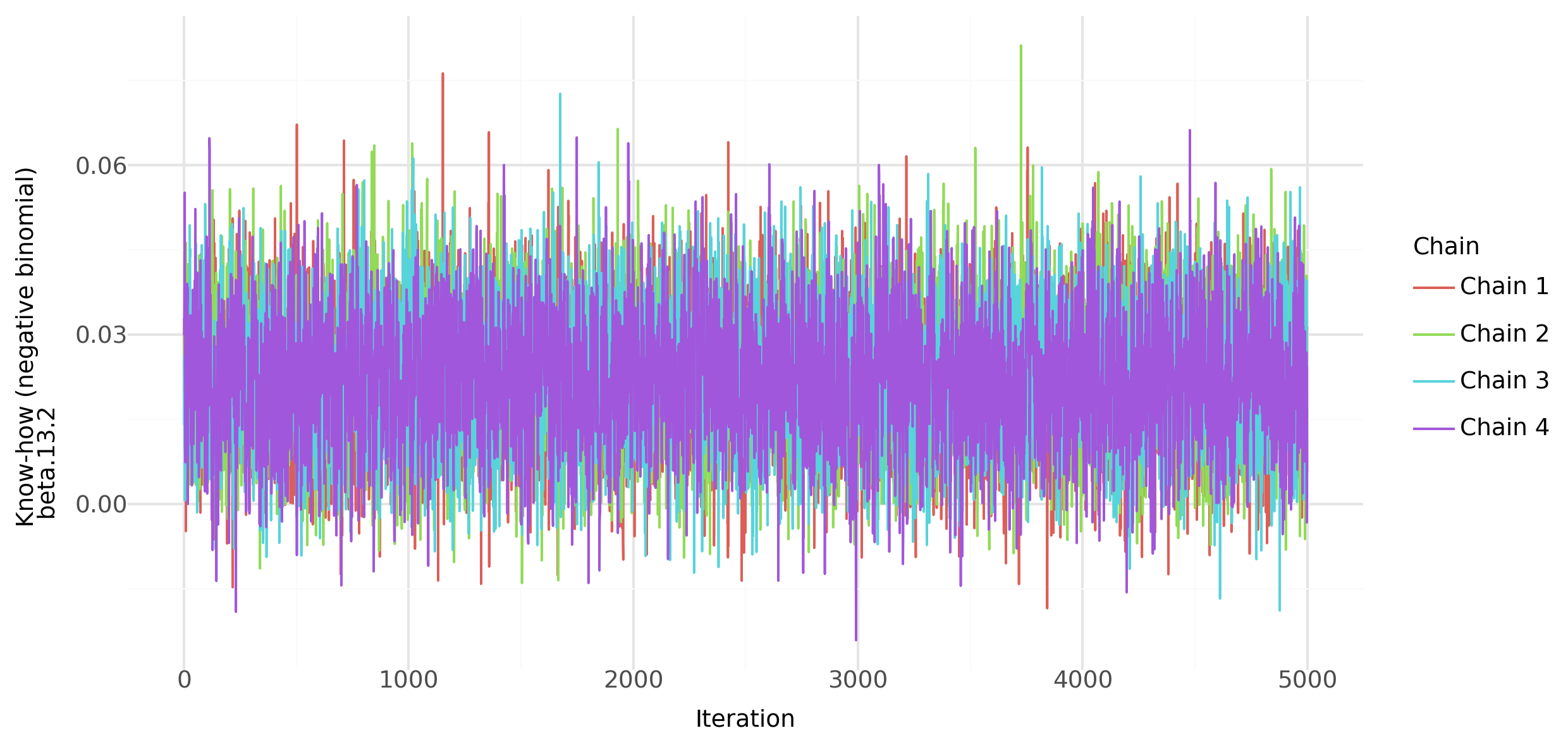


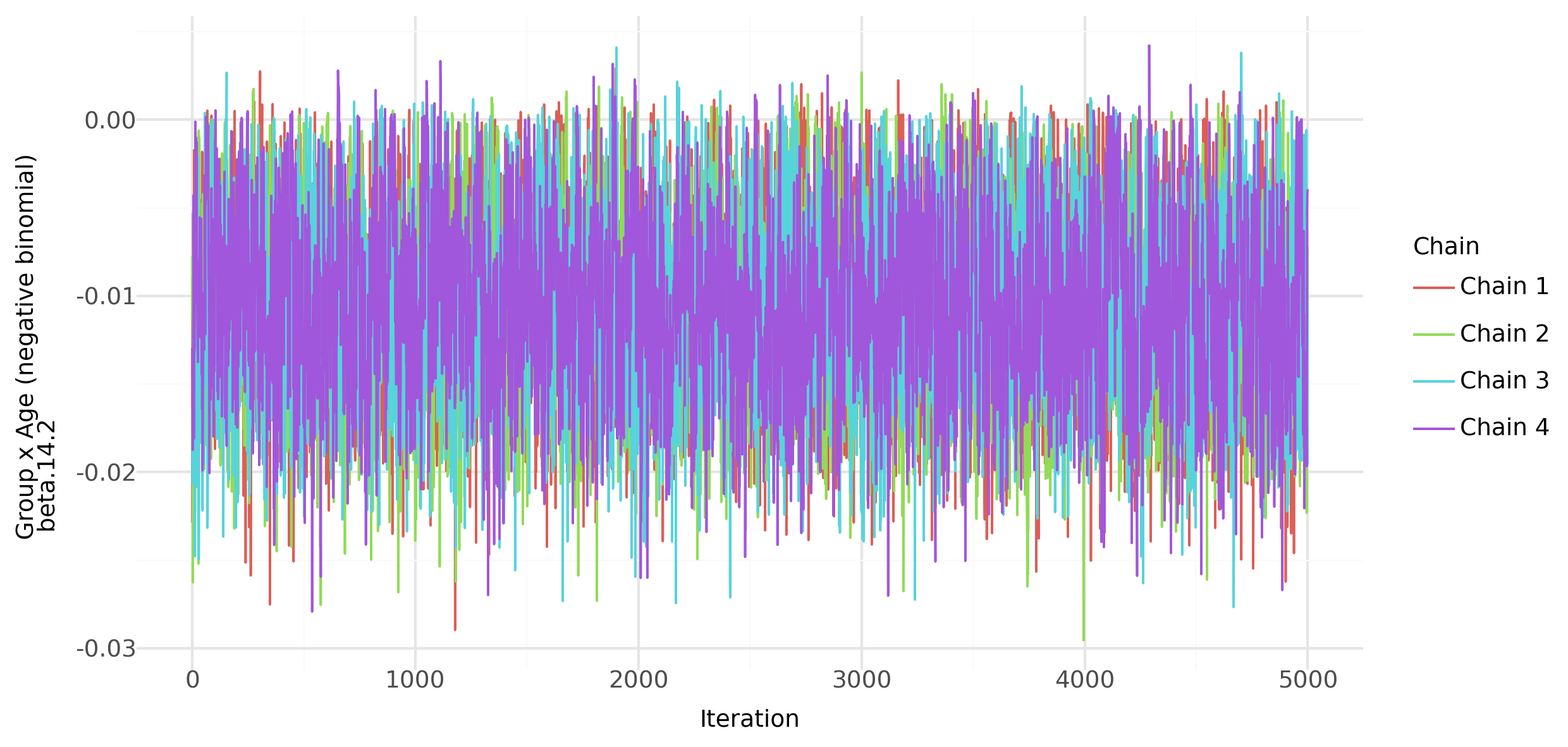


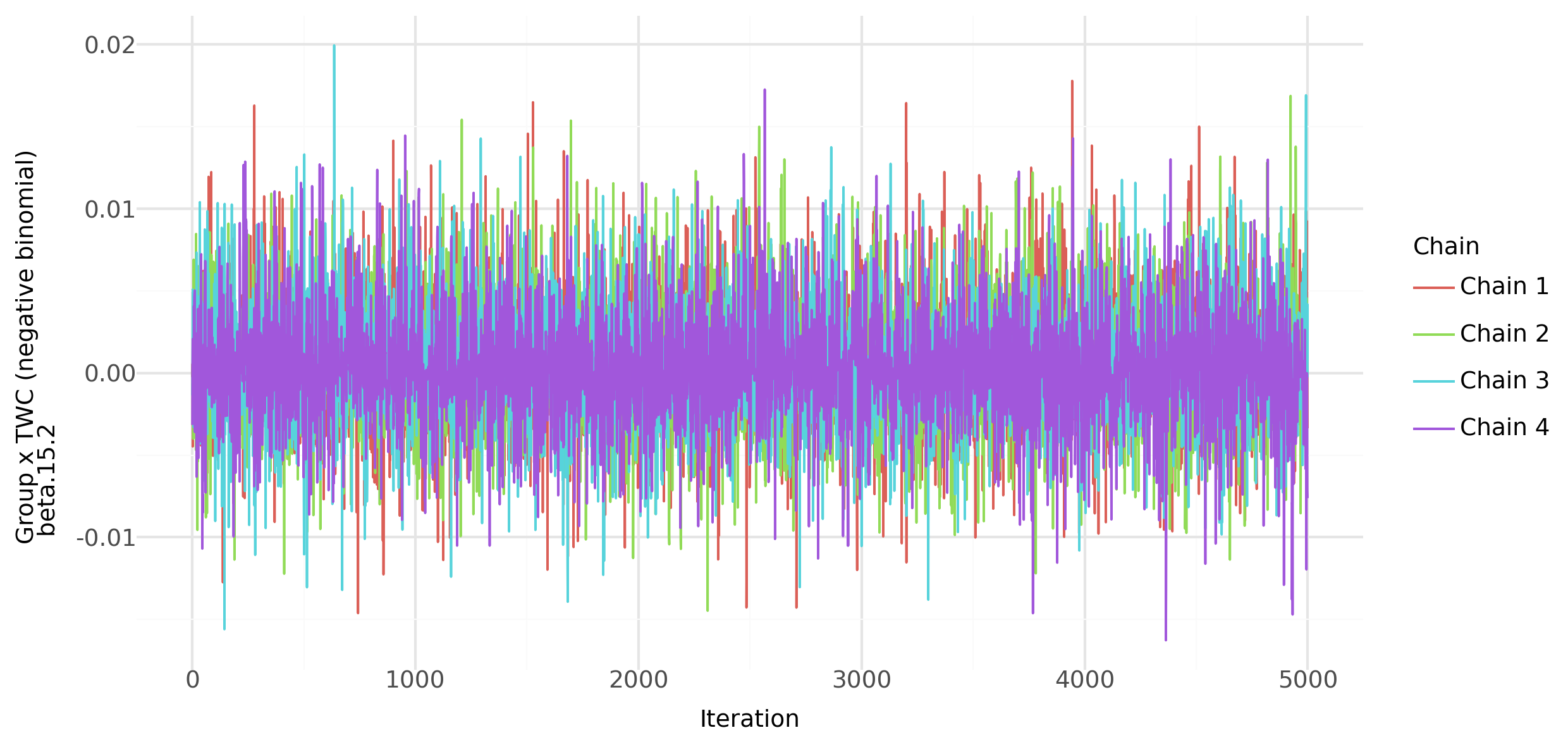


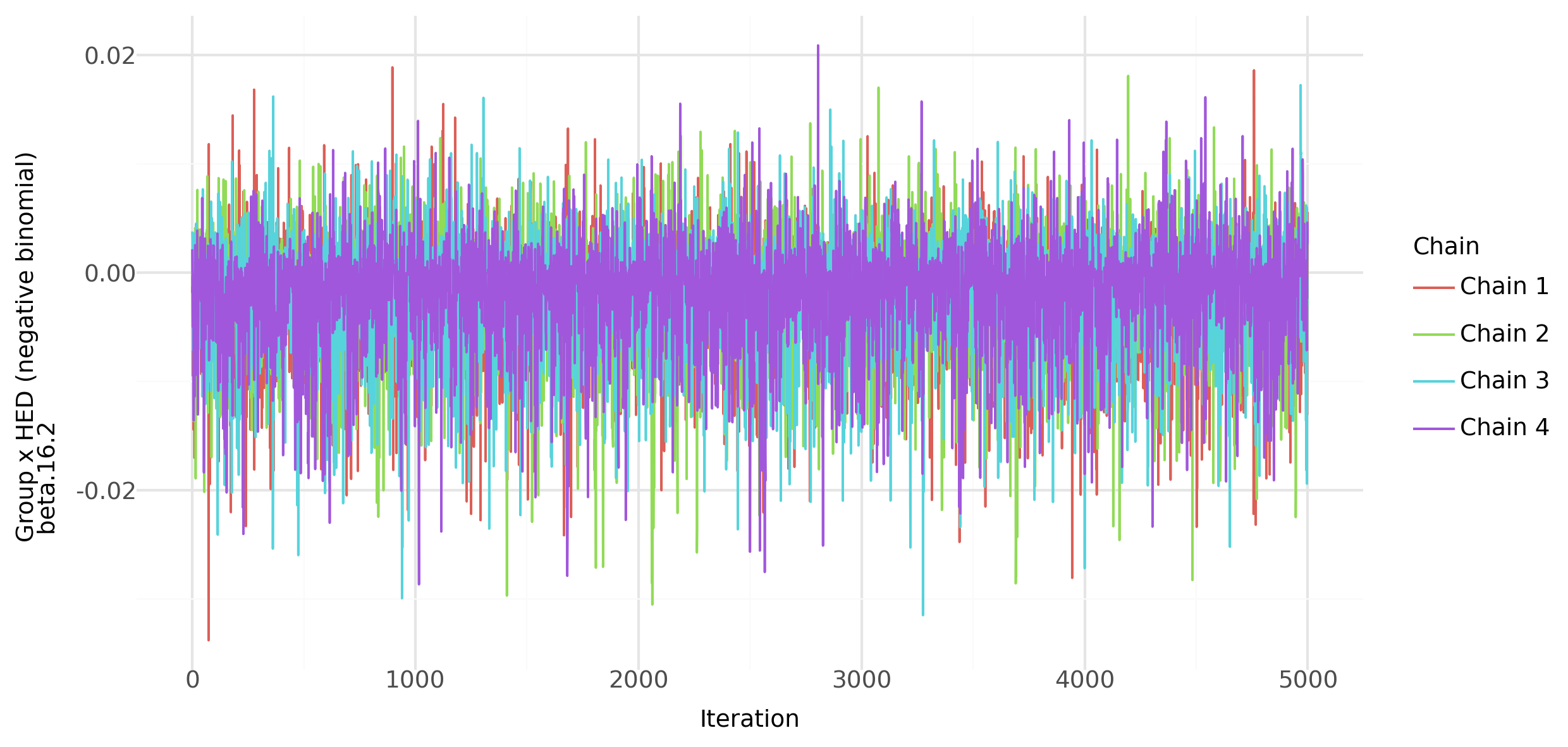


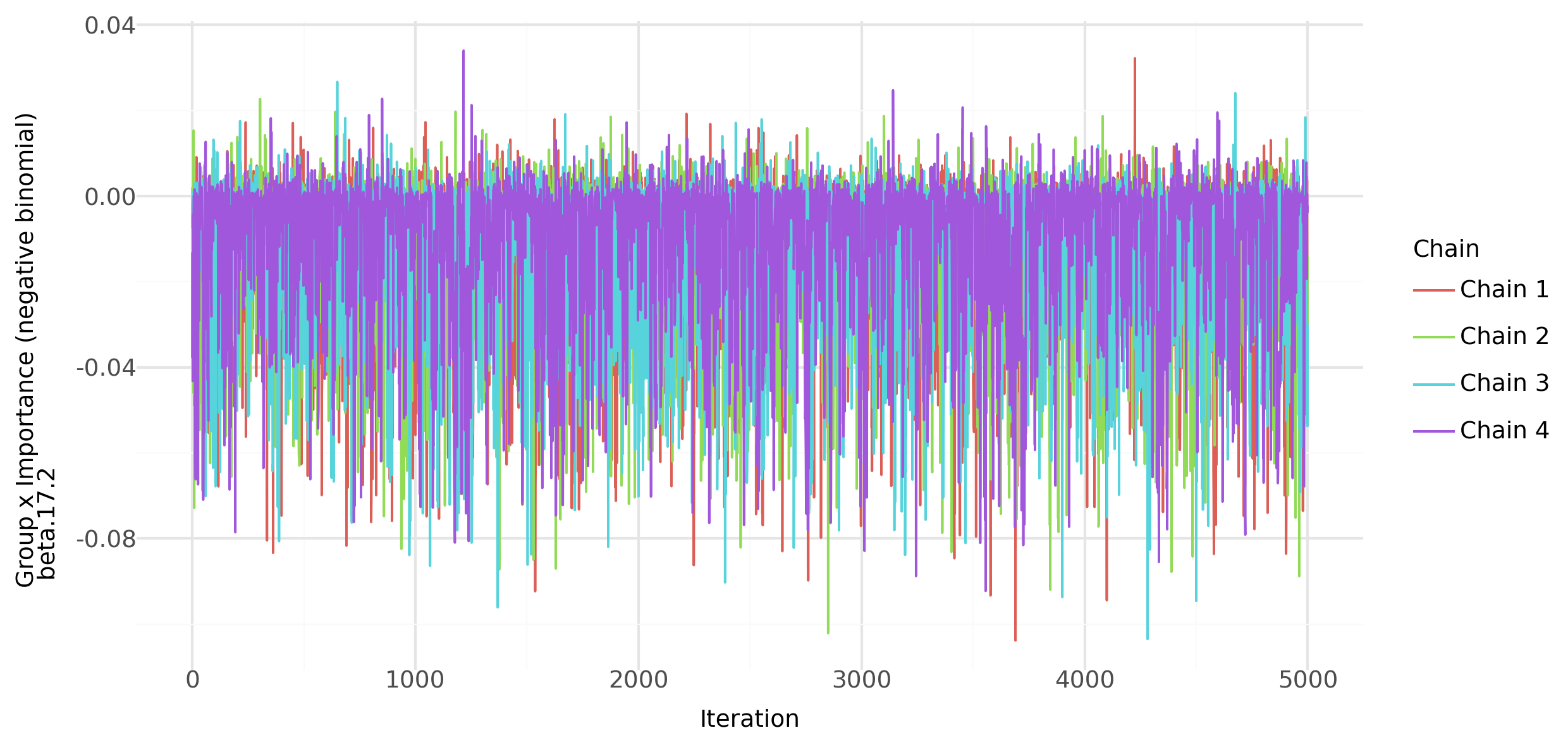


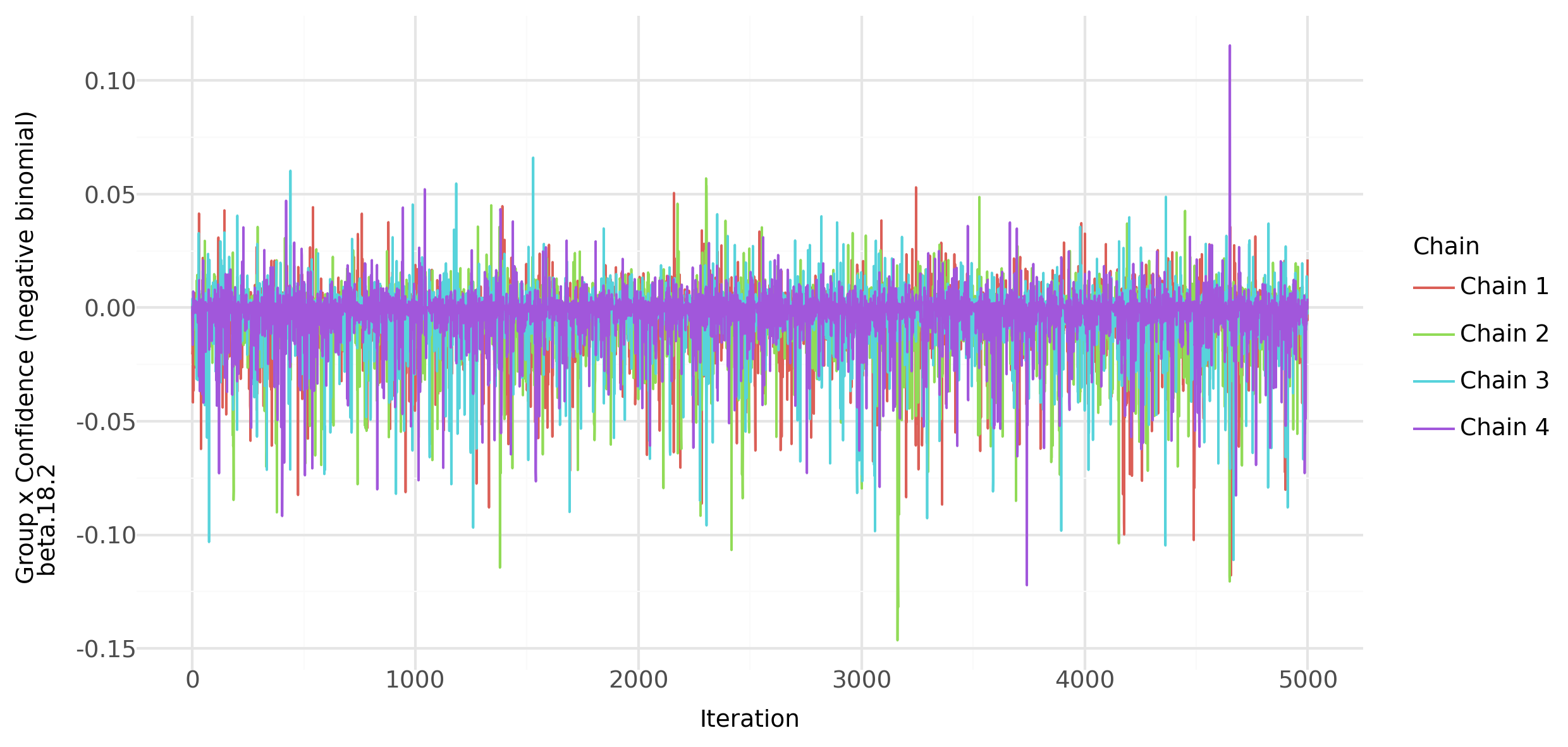


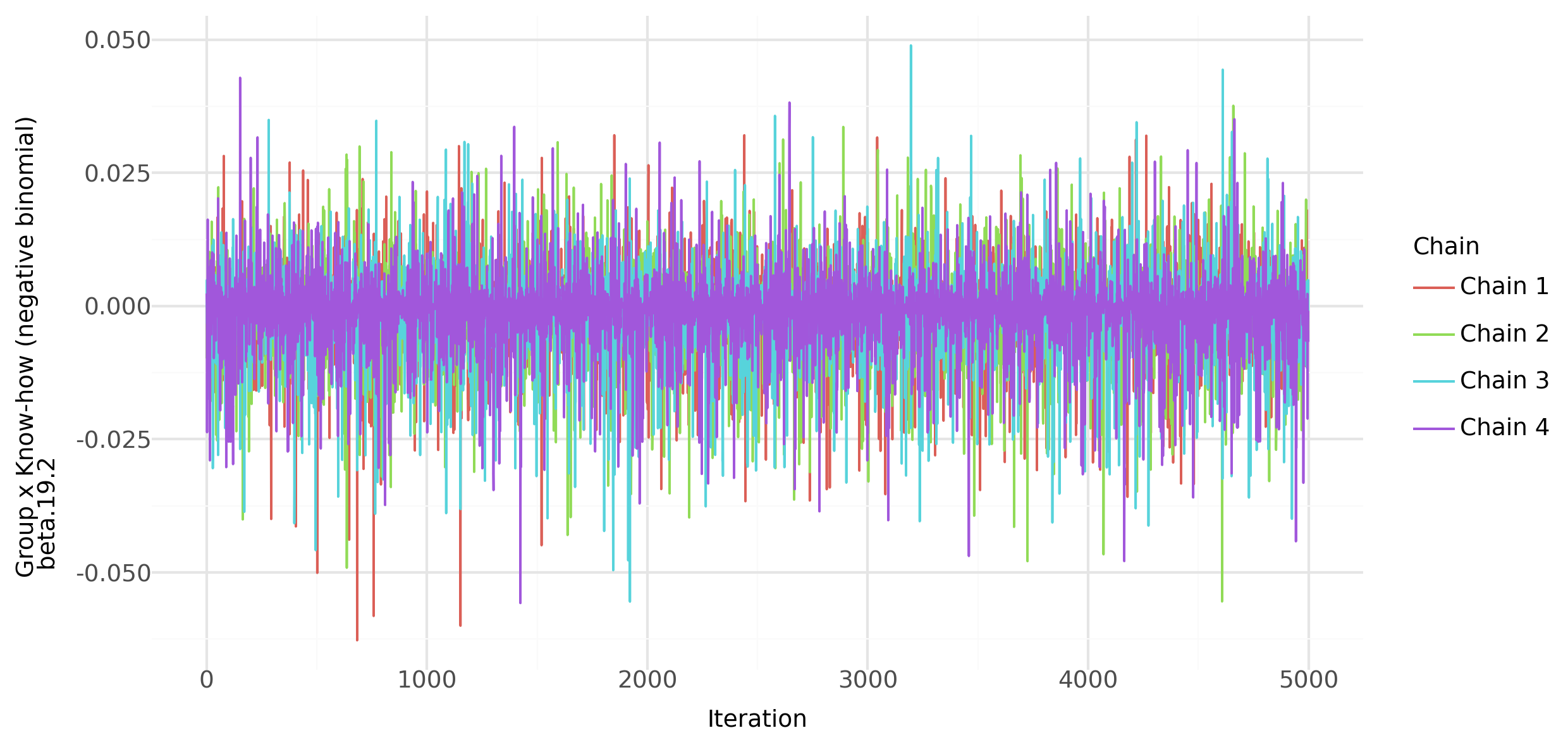


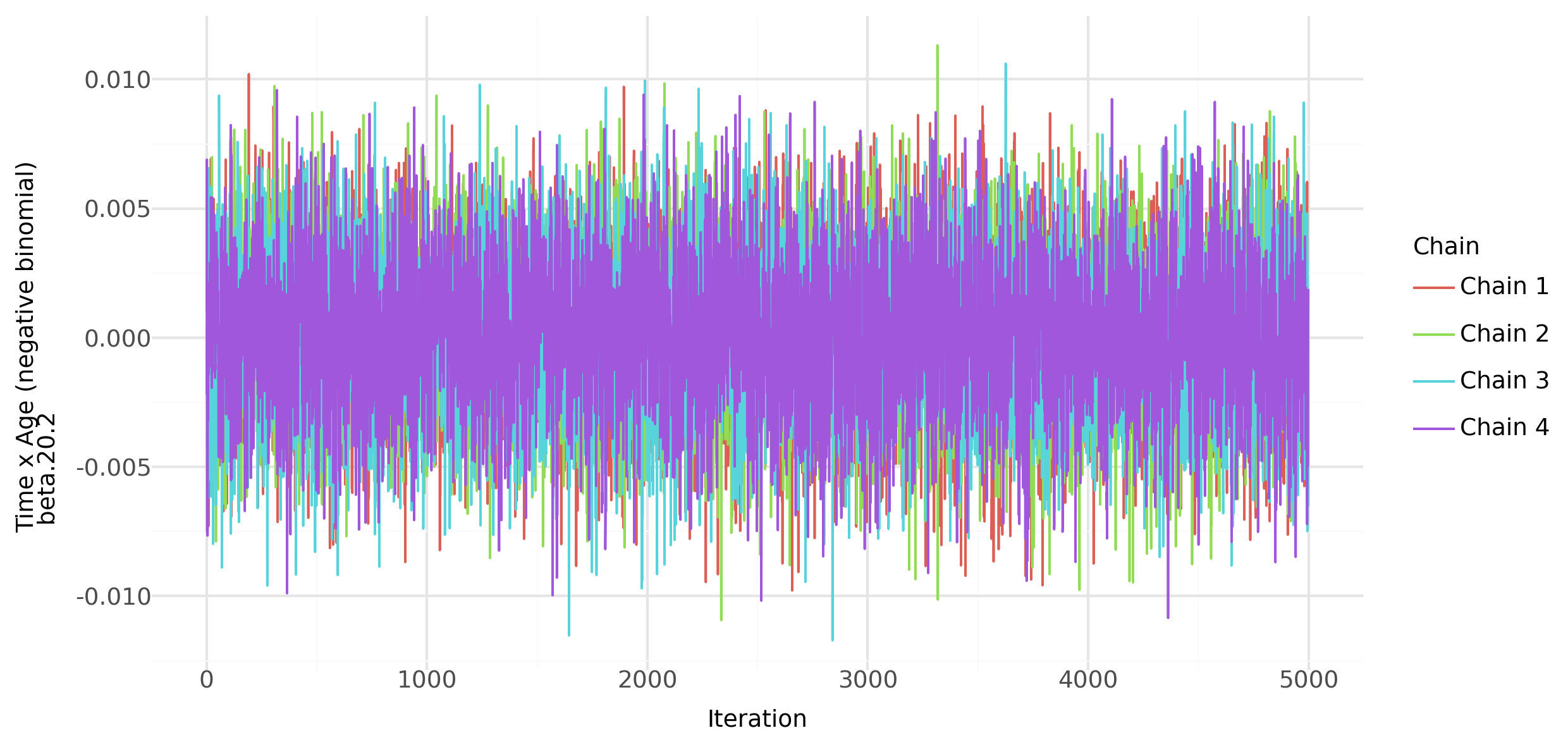


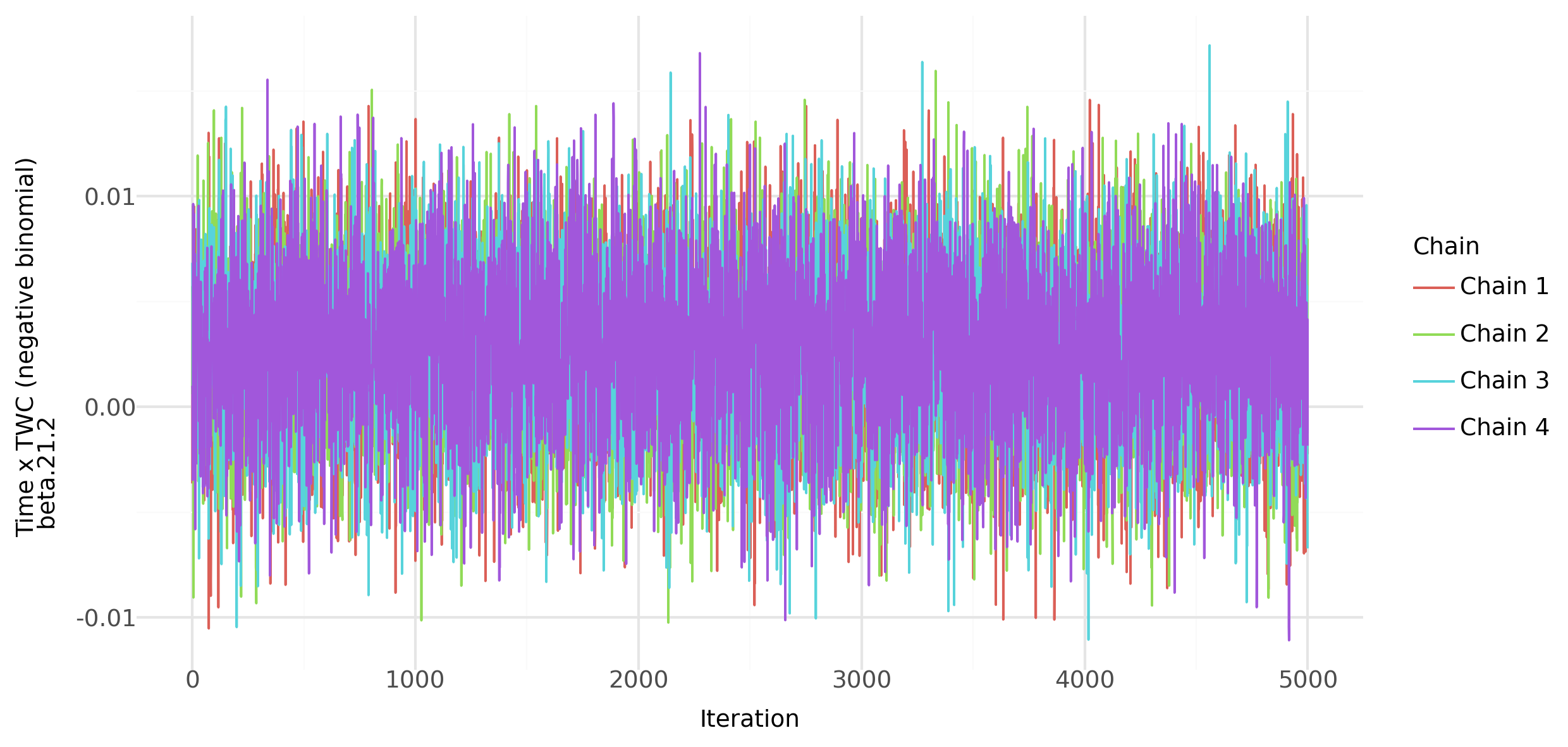


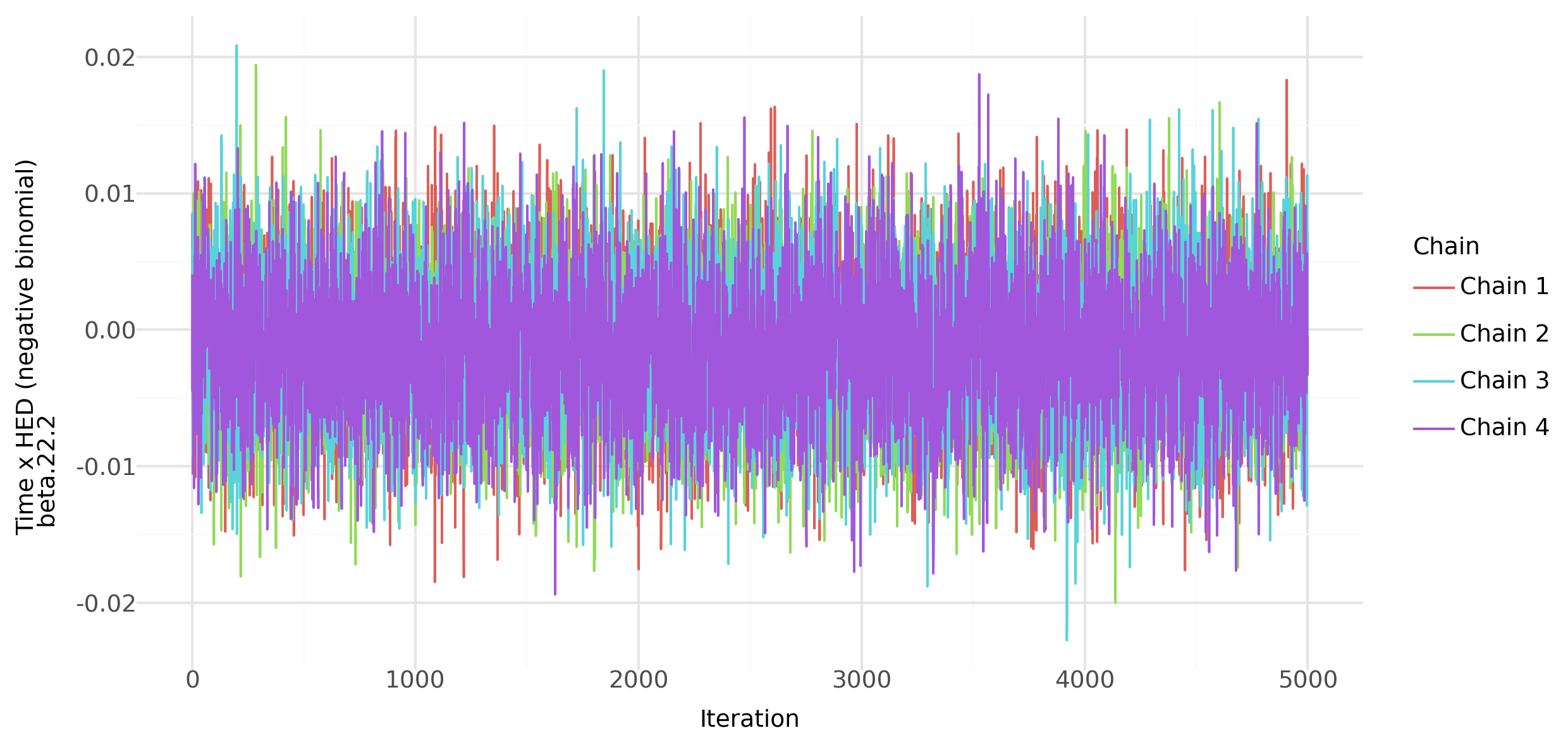


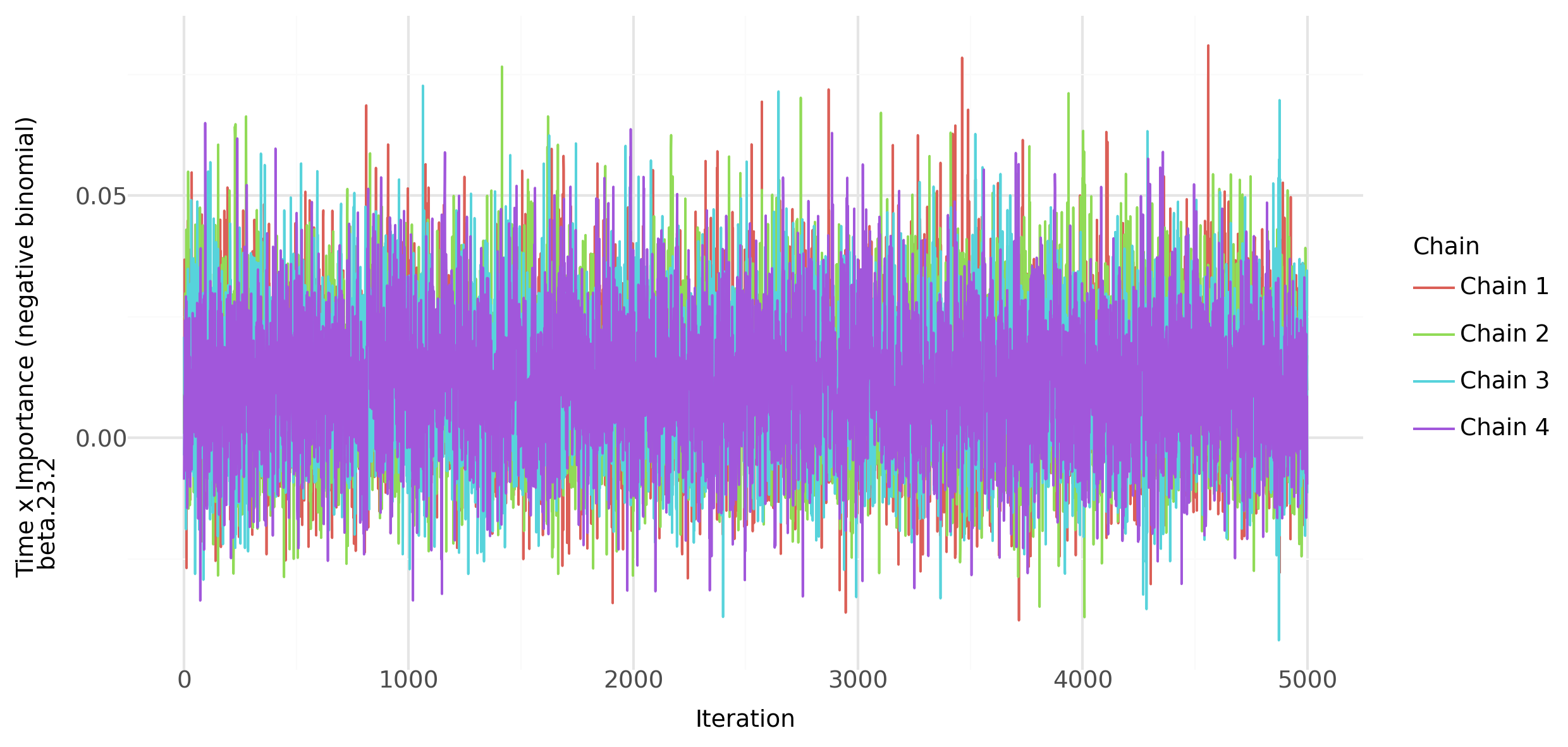


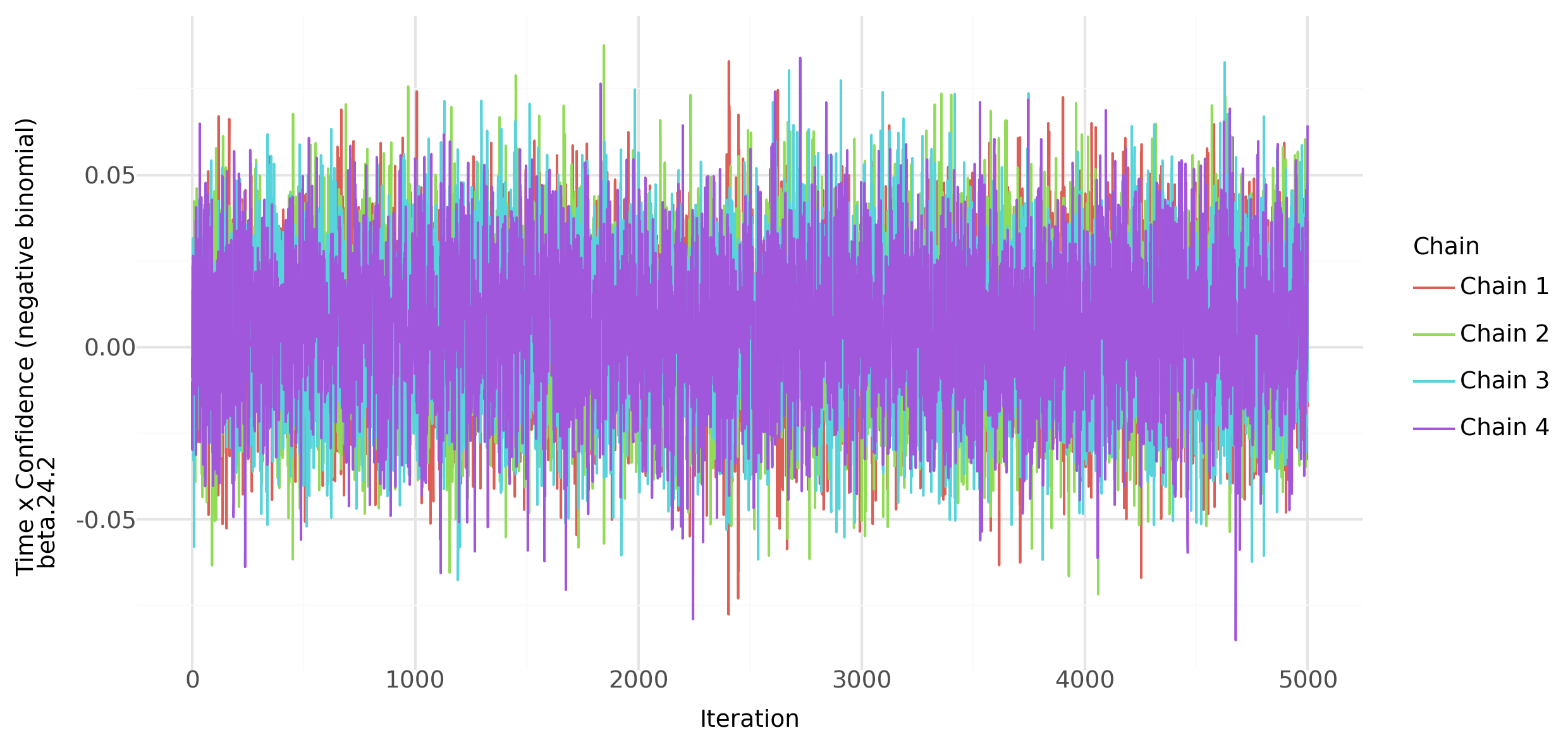


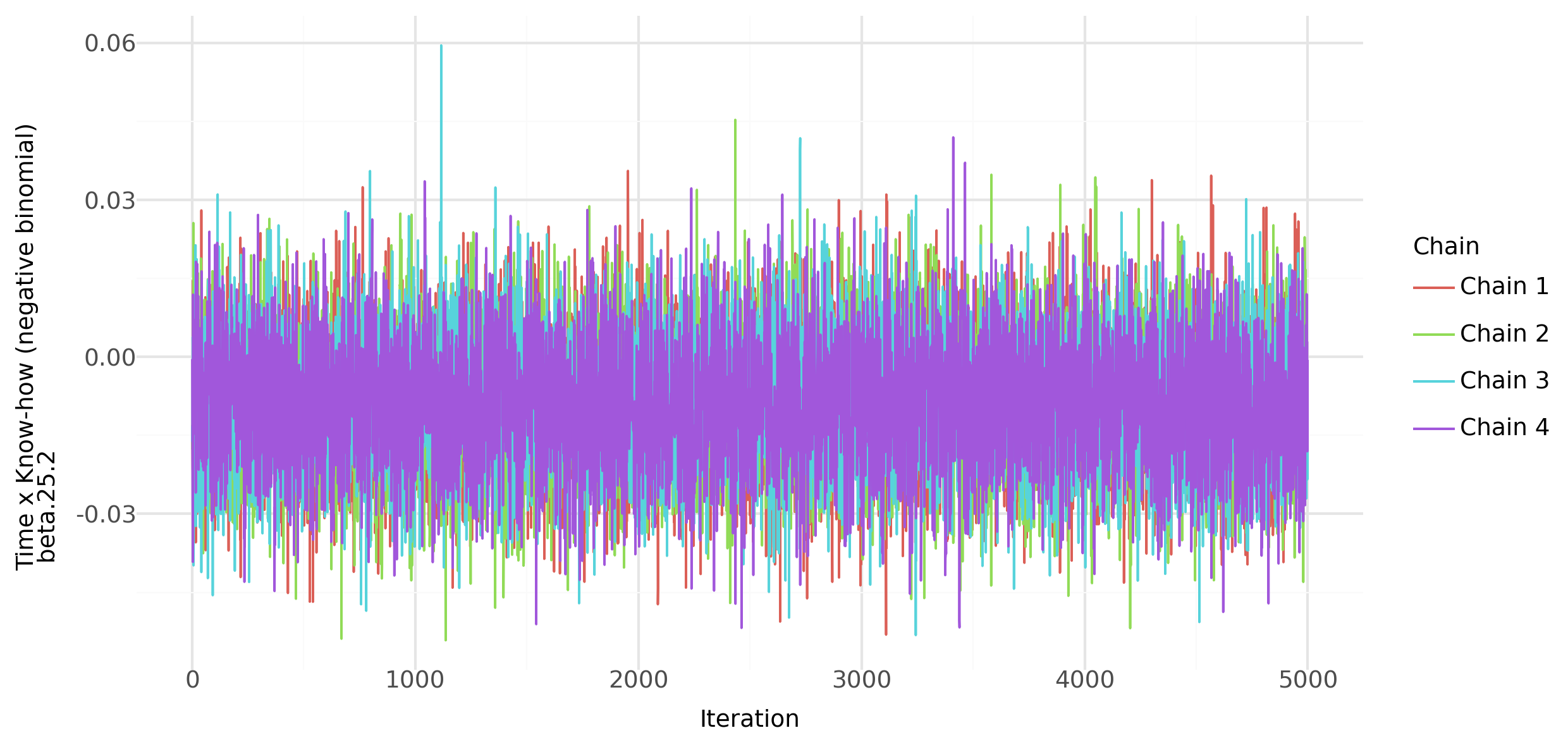


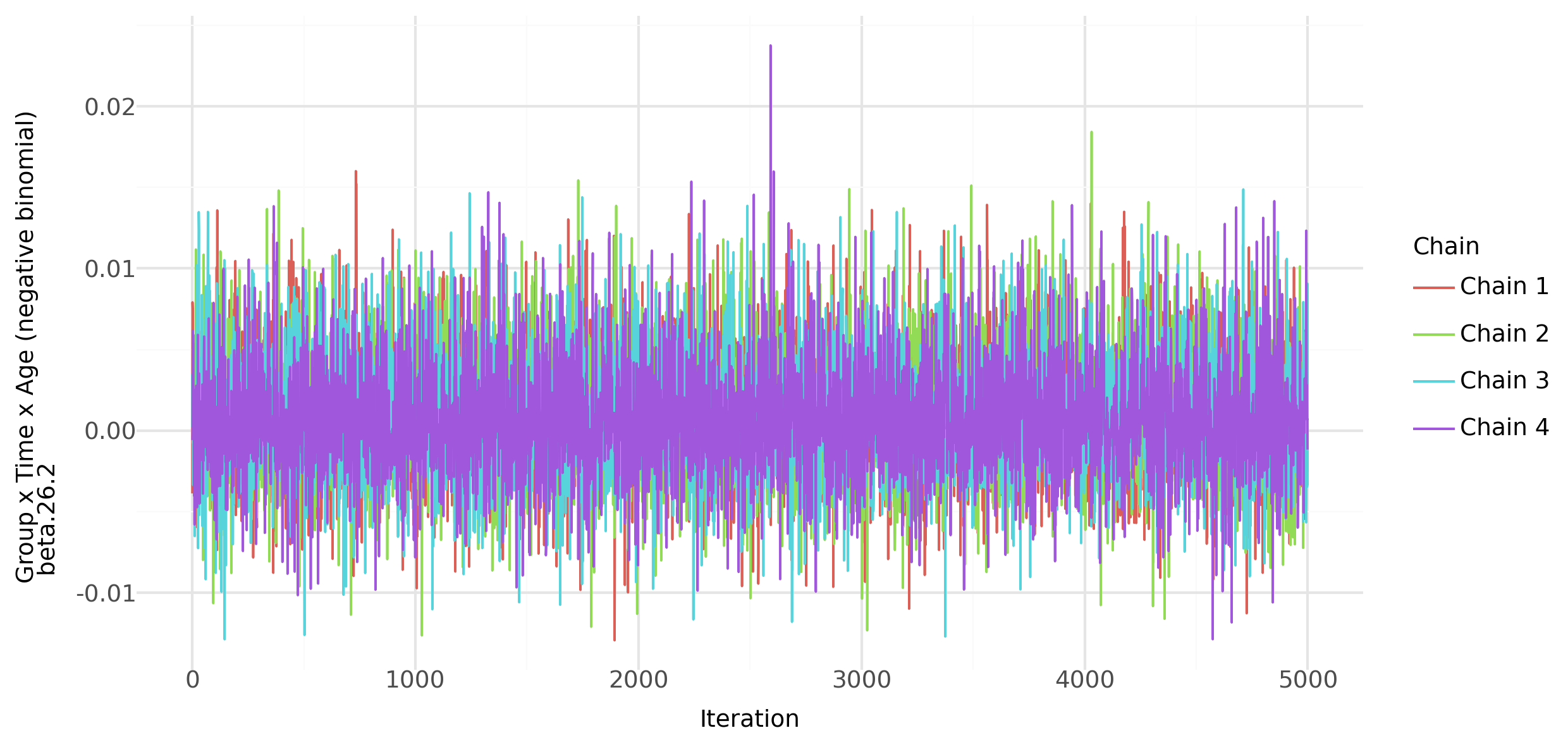


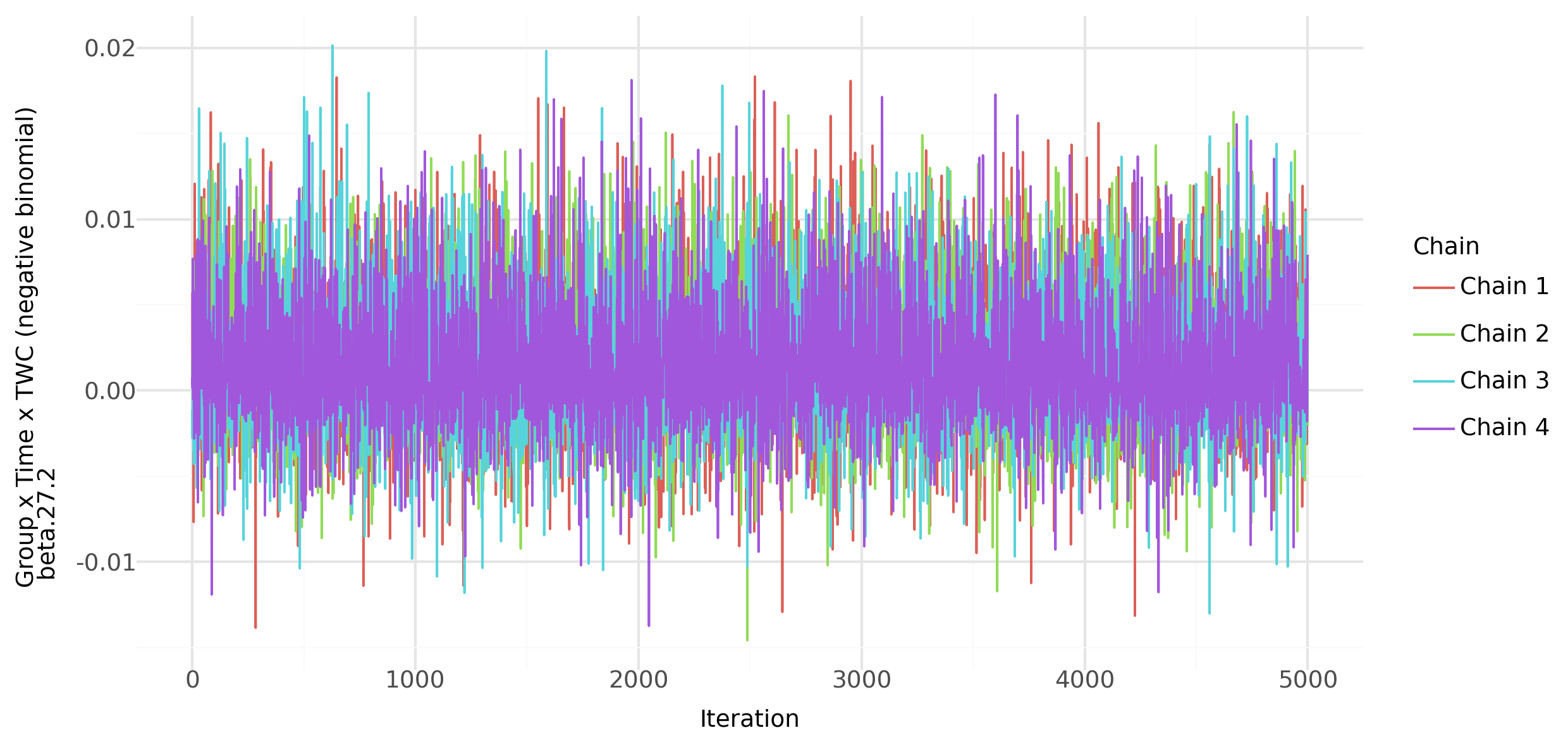


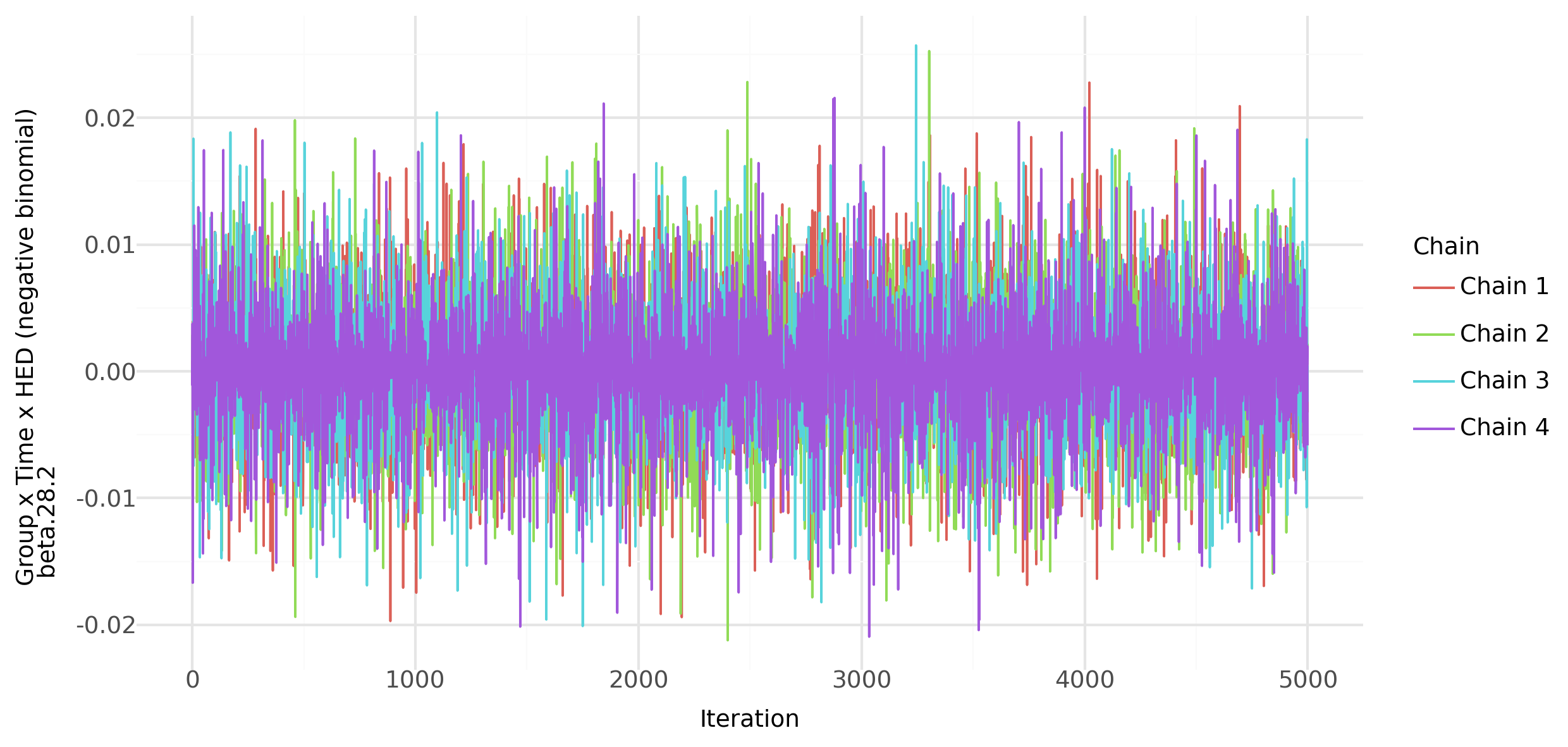


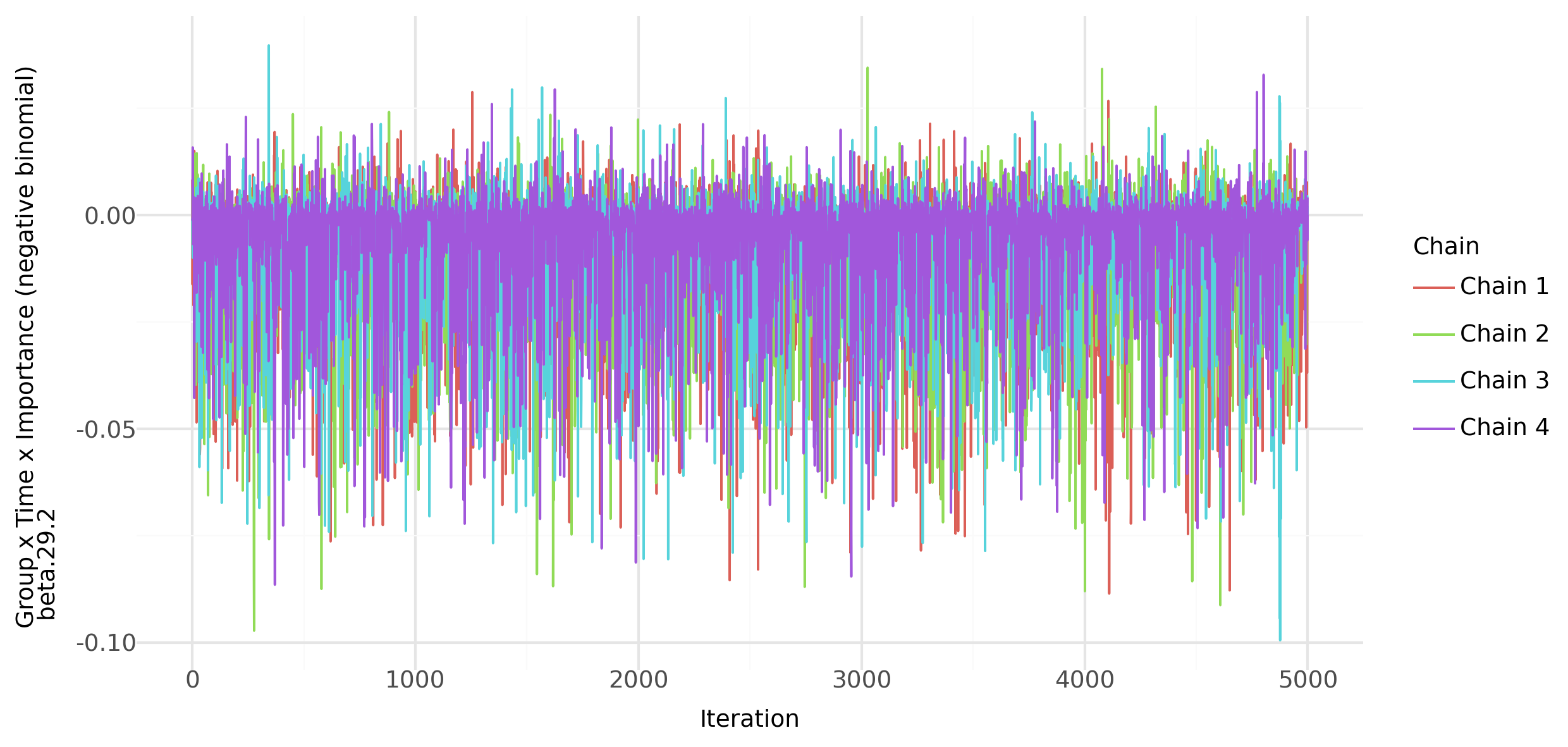


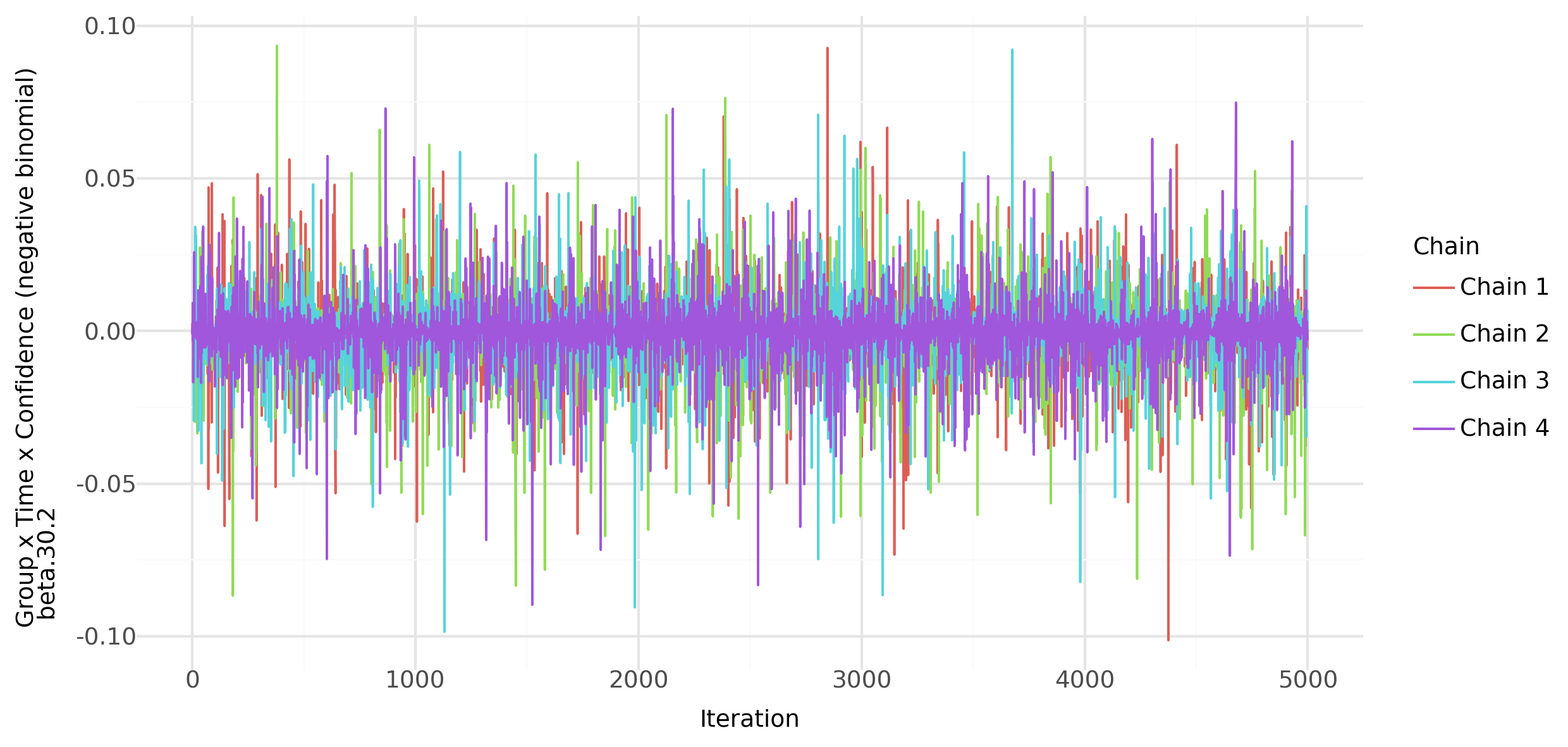


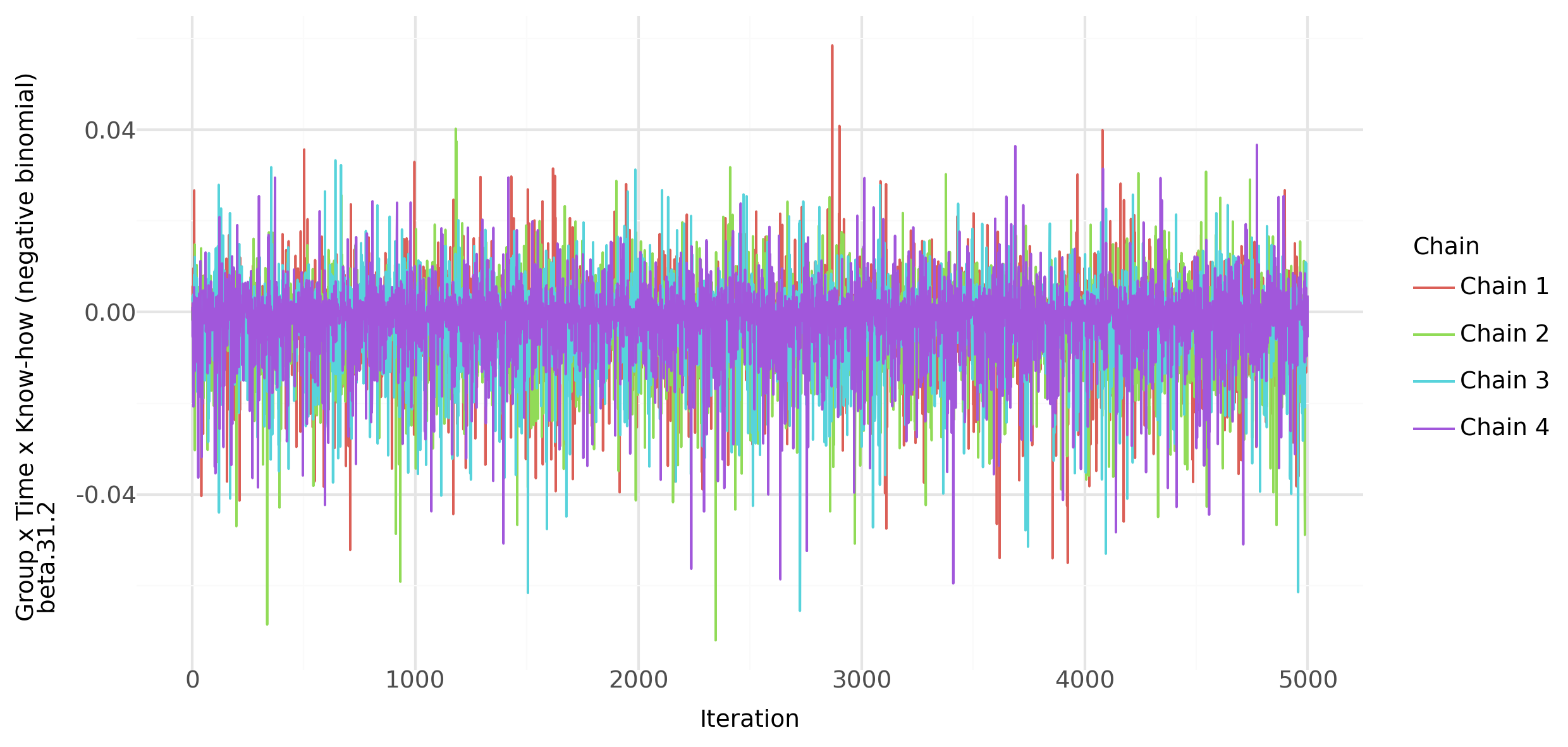


## Total weekly consumption

In Table 2, we provide an overview of the summary statistics, effective sample size, and R-hat for the estimation of the zero-inflated negative binomial regression mode for total weekly consumption. The R-hat value, which was within the acceptable bounds of 1, indicates the convergence of the Markov chains. Convergence is further illustrated in the trace plots following the table, which include samples after warmup.

| **Parameter** | **Mean (exp)** | **5% (exp)** | **50% (exp)** | **95% (exp)** | **N_eff** | **N_eff/s** | **R-hat** |
| --- | --- | --- | --- | --- | --- | --- | --- |
| alpha_theta | 0.0353308 | 0.0219059 | 0.0357216 | 0.0550232 | 7807 | 1.177 | 1 |
| alpha_eta | 9.39333 | 8.90845 | 9.39333 | 9.91452 | 8558 | 1.29 | 1 |
| beta[1,1] | 2.16106 | 1.42333 | 2.1417 | 3.38042 | 9965 | 1.503 | 1 |
| beta[2,1] | 0.955329 | 0.291126 | 0.935738 | 3.37367 | 6998 | 1.055 | 1 |
| beta[3,1] | 0.934317 | 0.588193 | 0.997426 | 1.04352 | 5235 | 0.7894 | 1.001 |
| beta[4,1] | 0.151981 | 0.0171802 | 0.158025 | 1.22116 | 11832 | 1.784 | 1 |
| beta[5,1] | 1.00956 | 0.585435 | 0.97917 | 1.99591 | 6494 | 0.9793 | 1 |
| beta[6,1] | 1.16149 | 0.22313 | 1.24234 | 4.71147 | 8383 | 1.264 | 1 |
| beta[7,1] | 0.821766 | 0.279711 | 0.994872 | 1.03269 | 3823 | 0.5764 | 1 |
| beta[8,1] | 0.982986 | 0.964631 | 0.983006 | 1.00178 | 10894 | 1.643 | 1 |
| beta[9,1] | 0.990381 | 0.964283 | 0.990651 | 1.01602 | 9908 | 1.494 | 1 |
| beta[10,1] | 0.949661 | 0.9091 | 0.950564 | 0.988675 | 10519 | 1.586 | 1 |
| beta[11,1] | 1.06685 | 0.980287 | 1.06556 | 1.16358 | 11028 | 1.663 | 1 |
| beta[12,1] | 1.31535 | 1.12142 | 1.31232 | 1.55768 | 11139 | 1.68 | 1 |
| beta[13,1] | 1.01736 | 0.939667 | 1.01786 | 1.10218 | 12273 | 1.851 | 1.001 |
| beta[14,1] | 0.99918 | 0.979846 | 0.999732 | 1.01766 | 12021 | 1.813 | 1 |
| beta[15,1] | 1.0081 | 0.988121 | 1.00469 | 1.03739 | 10627 | 1.602 | 1 |
| beta[16,1] | 1.01674 | 0.987341 | 1.00899 | 1.07049 | 11432 | 1.724 | 1 |
| beta[17,1] | 0.994747 | 0.937996 | 0.99932 | 1.03911 | 11705 | 1.765 | 1 |
| beta[18,1] | 0.990338 | 0.912087 | 0.99891 | 1.0398 | 7478 | 1.128 | 1 |
| beta[19,1] | 0.999946 | 0.955653 | 0.999992 | 1.04614 | 18974 | 2.861 | 1 |
| beta[20,1] | 1.01329 | 0.990701 | 1.01328 | 1.03665 | 15074 | 2.273 | 1 |
| beta[21,1] | 0.998144 | 0.96841 | 0.998328 | 1.02892 | 12480 | 1.882 | 1 |
| beta[22,1] | 1.00539 | 0.944169 | 1.00788 | 1.06161 | 9564 | 1.442 | 1 |
| beta[23,1] | 0.901856 | 0.790571 | 0.906658 | 1.00869 | 9087 | 1.37 | 1 |
| beta[24,1] | 1.16766 | 0.957021 | 1.16544 | 1.43505 | 12637 | 1.906 | 1 |
| beta[25,1] | 0.997159 | 0.908028 | 0.996759 | 1.0947 | 16821 | 2.536 | 1.001 |
| beta[26,1] | 1.00188 | 0.981739 | 1.00085 | 1.0246 | 15144 | 2.284 | 1 |
| beta[27,1] | 0.998157 | 0.968536 | 0.999788 | 1.02279 | 13760 | 2.075 | 1 |
| beta[28,1] | 1.04221 | 0.99495 | 1.03224 | 1.12671 | 7870 | 1.187 | 1 |
| beta[29,1] | 1.05371 | 0.985969 | 1.01274 | 1.26858 | 5713 | 0.8614 | 1 |
| beta[30,1] | 0.99103 | 0.909992 | 0.999198 | 1.04794 | 10552 | 1.591 | 1 |
| beta[31,1] | 1.00297 | 0.959205 | 1.00044 | 1.0591 | 19645 | 2.962 | 1 |
| beta[1,2] | 1.17339 | 1.0966 | 1.17363 | 1.25646 | 8974 | 1.353 | 1 |
| beta[2,2] | 1.75155 | 1.17574 | 1.72409 | 2.75936 | 4053 | 0.6111 | 1.001 |
| beta[3,2] | 1.00134 | 0.985506 | 1.00019 | 1.02113 | 16123 | 2.431 | 1 |
| beta[4,2] | 1.00988 | 0.747665 | 1.01263 | 1.35297 | 11620 | 1.752 | 1 |
| beta[5,2] | 1.0372 | 0.963638 | 1.03729 | 1.1165 | 16646 | 2.51 | 1 |
| beta[6,2] | 0.985417 | 0.755935 | 0.972797 | 1.34232 | 9631 | 1.452 | 1 |
| beta[7,2] | 1.00465 | 0.988012 | 1.00048 | 1.03725 | 13068 | 1.97 | 1 |
| beta[8,2] | 1.00421 | 1.00043 | 1.00422 | 1.00799 | 8099 | 1.221 | 1 |
| beta[9,2] | 1.02365 | 1.0192 | 1.02366 | 1.02809 | 9060 | 1.366 | 1 |
| beta[10,2] | 1.0127 | 1.00627 | 1.01261 | 1.01947 | 7877 | 1.188 | 1 |
| beta[11,2] | 0.972174 | 0.952791 | 0.972213 | 0.992063 | 4715 | 0.711 | 1.001 |
| beta[12,2] | 0.994256 | 0.968323 | 0.99308 | 1.02442 | 6398 | 0.9647 | 1 |
| beta[13,2] | 1.01596 | 1.00276 | 1.01593 | 1.02968 | 10348 | 1.56 | 1 |
| beta[14,2] | 0.993393 | 0.988072 | 0.993348 | 0.998835 | 7635 | 1.151 | 1 |
| beta[15,2] | 0.999821 | 0.99538 | 0.999832 | 1.00434 | 11658 | 1.758 | 1 |
| beta[16,2] | 0.995489 | 0.986729 | 0.996356 | 1.00146 | 7143 | 1.077 | 1.001 |
| beta[17,2] | 0.971455 | 0.943754 | 0.970416 | 0.999831 | 3671 | 0.5535 | 1.001 |
| beta[18,2] | 0.987124 | 0.945341 | 0.995963 | 1.00441 | 4227 | 0.6374 | 1.001 |
| beta[19,2] | 0.99964 | 0.989882 | 0.999871 | 1.00889 | 16469 | 2.483 | 1 |
| beta[20,2] | 0.999519 | 0.996011 | 0.999505 | 1.00307 | 14923 | 2.25 | 1 |
| beta[21,2] | 0.998549 | 0.994054 | 0.998546 | 1.00307 | 14529 | 2.191 | 1 |
| beta[22,2] | 1.00357 | 0.997379 | 1.00359 | 1.00974 | 15535 | 2.343 | 1 |
| beta[23,2] | 1.01199 | 0.996264 | 1.01186 | 1.02839 | 14511 | 2.188 | 1 |
| beta[24,2] | 0.991319 | 0.966746 | 0.991044 | 1.01727 | 13712 | 2.068 | 1 |
| beta[25,2] | 1.00376 | 0.989416 | 1.00382 | 1.01811 | 18112 | 2.731 | 1 |
| beta[26,2] | 0.999167 | 0.994661 | 0.999383 | 1.00324 | 13091 | 1.974 | 1 |
| beta[27,2] | 1.00076 | 0.996405 | 1.00052 | 1.00573 | 16657 | 2.512 | 1 |
| beta[28,2] | 0.999901 | 0.993918 | 0.999918 | 1.00588 | 20258 | 3.055 | 0.9999 |
| beta[29,2] | 0.997399 | 0.981906 | 0.999102 | 1.00712 | 14442 | 2.178 | 1 |
| beta[30,2] | 0.996873 | 0.976794 | 0.999166 | 1.00842 | 11380 | 1.716 | 1 |
| beta[31,2] | 1.0005 | 0.991404 | 1.00019 | 1.01092 | 20693 | 3.12 | 0.9999 |


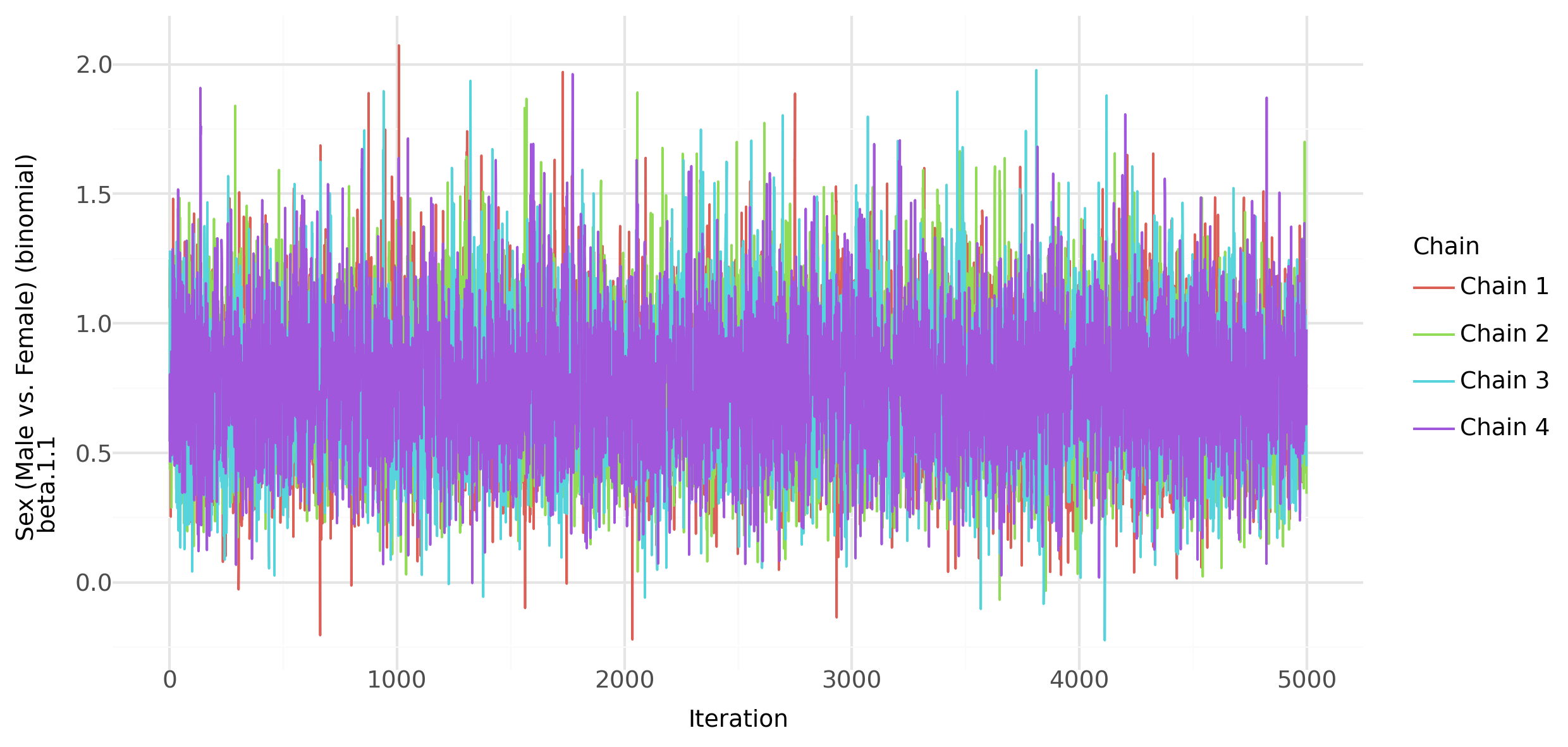


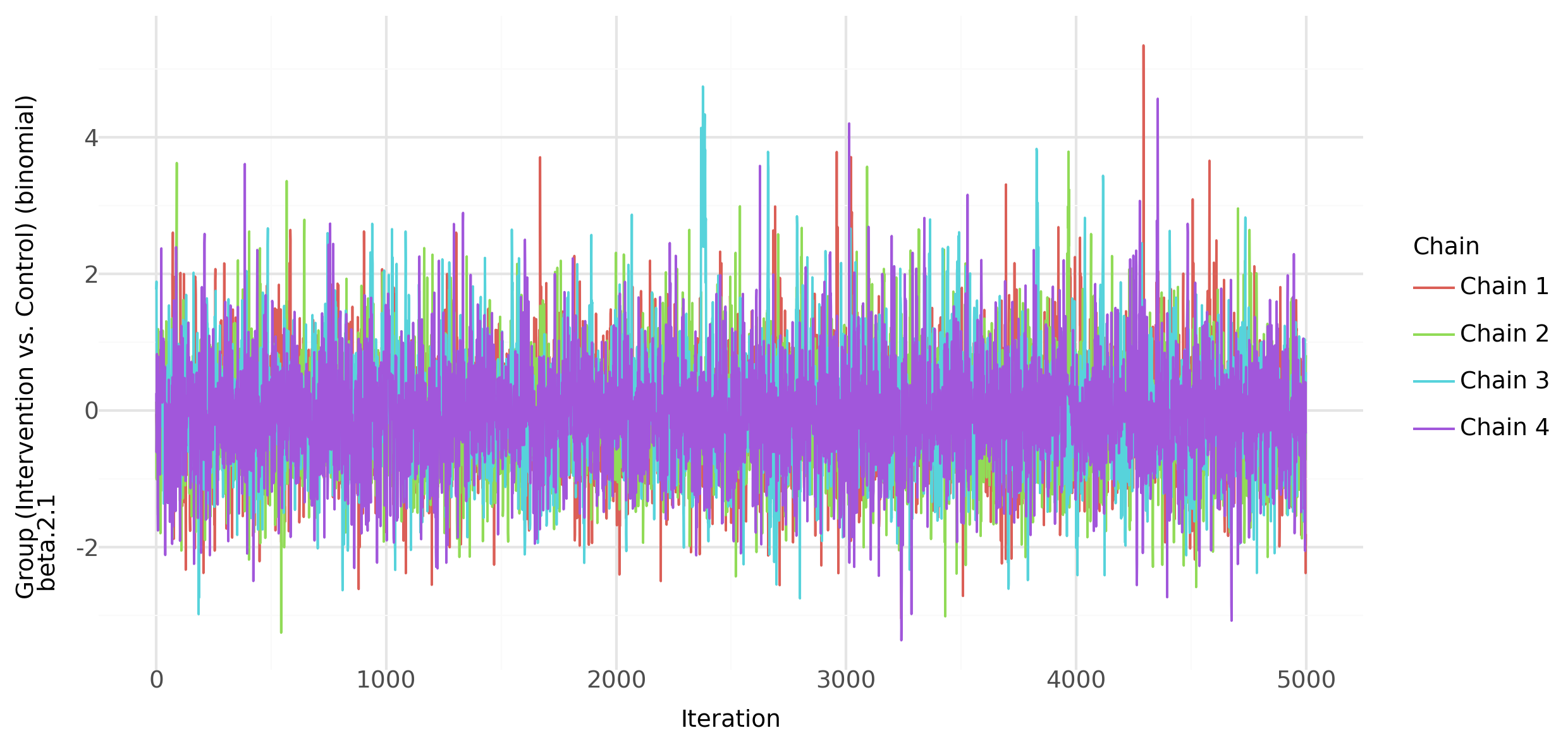


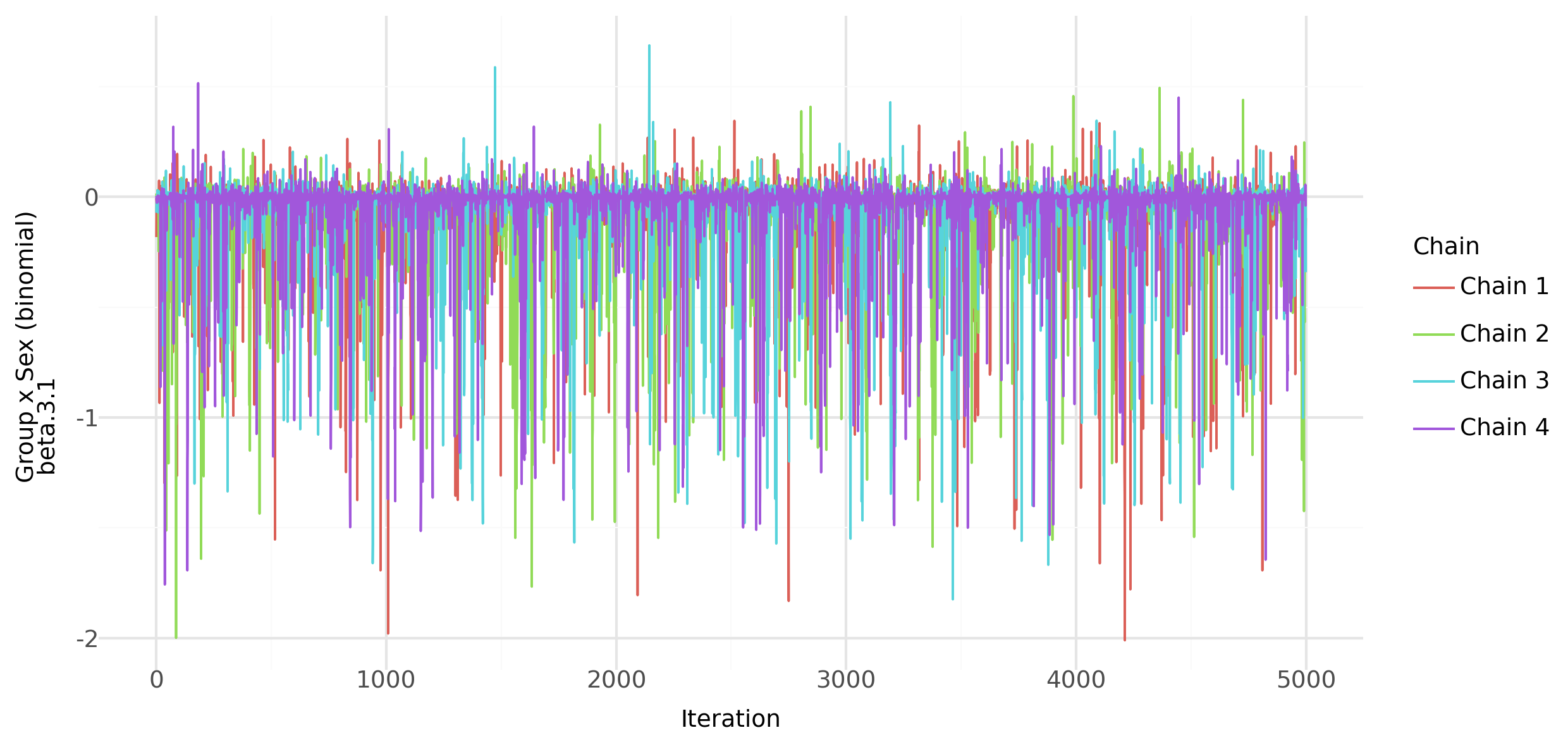


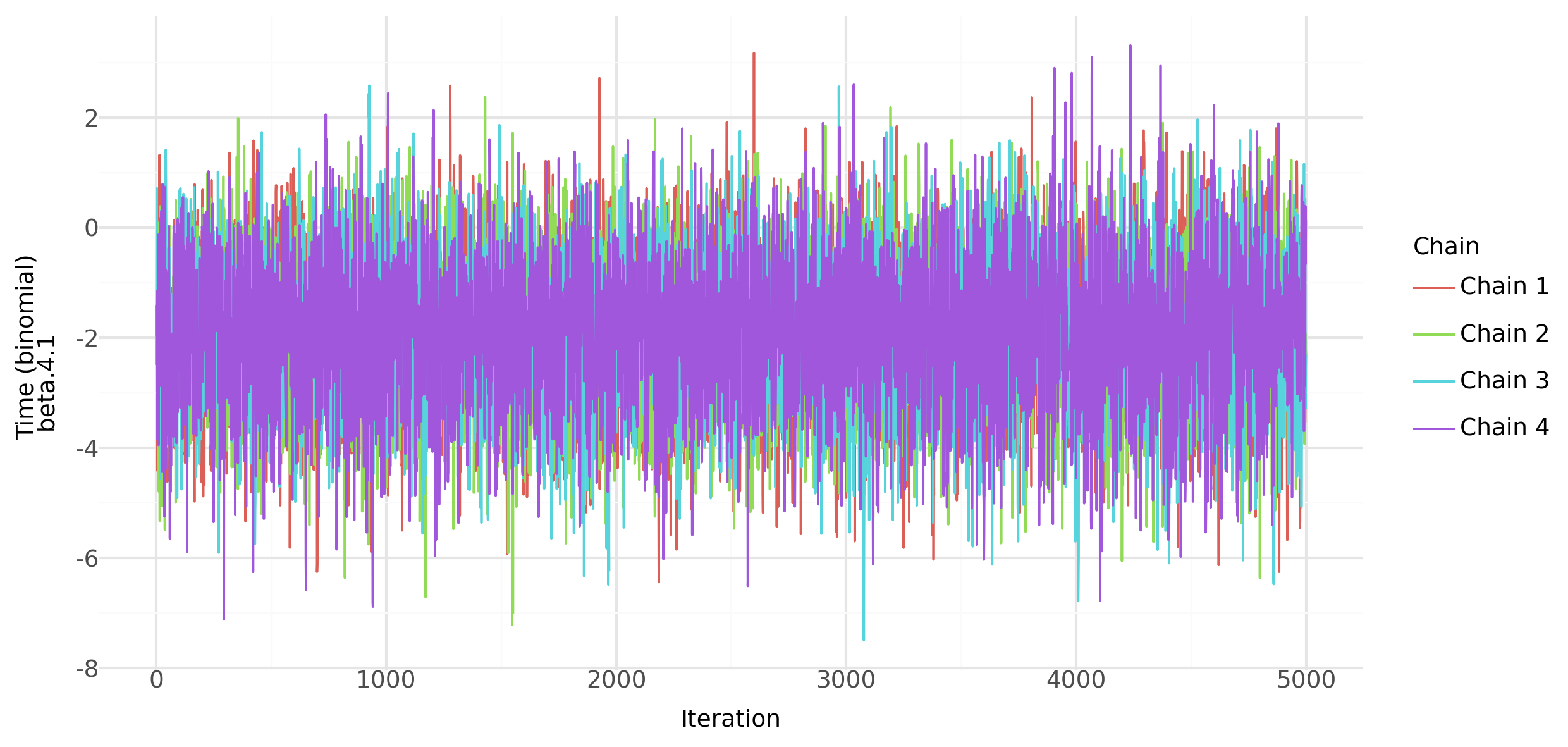


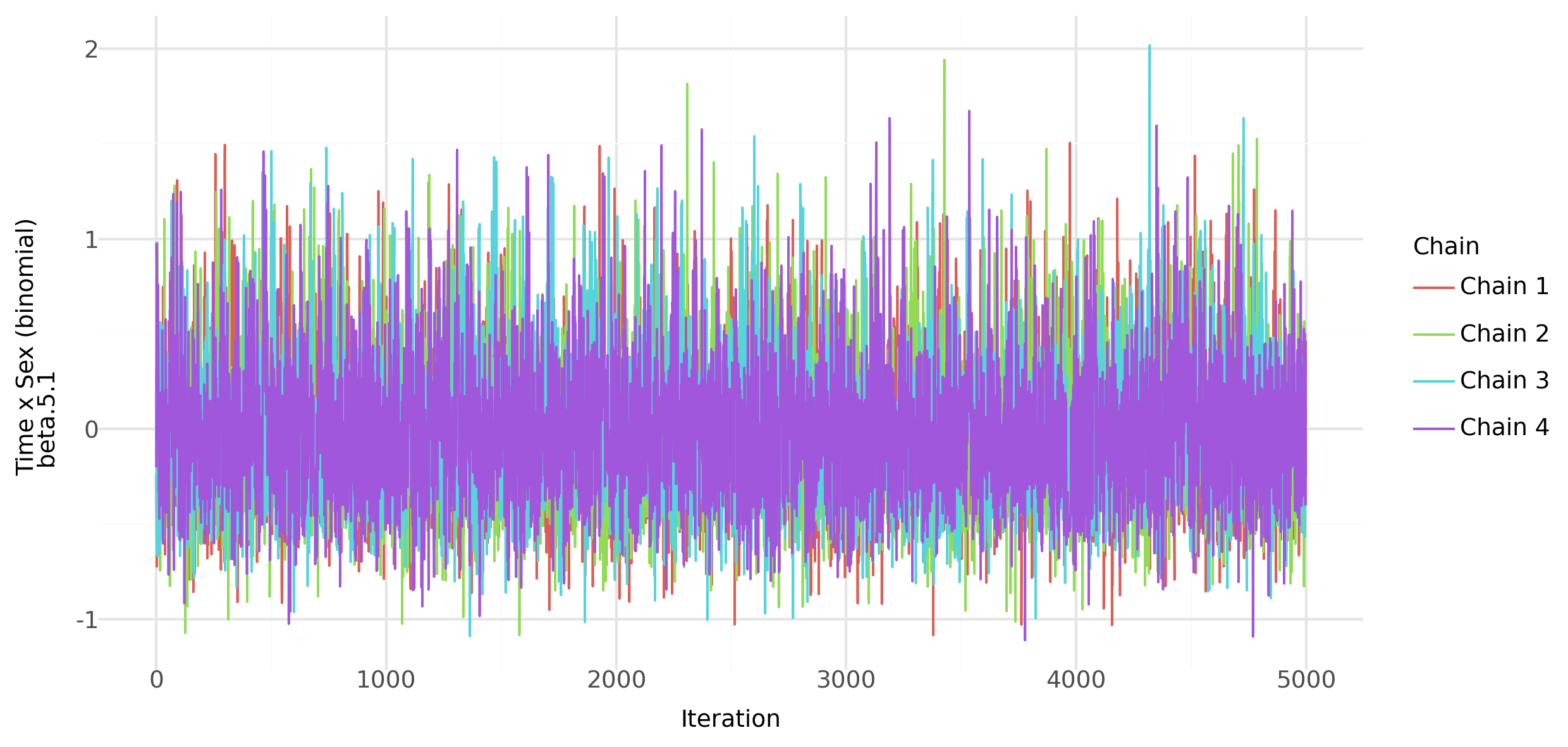


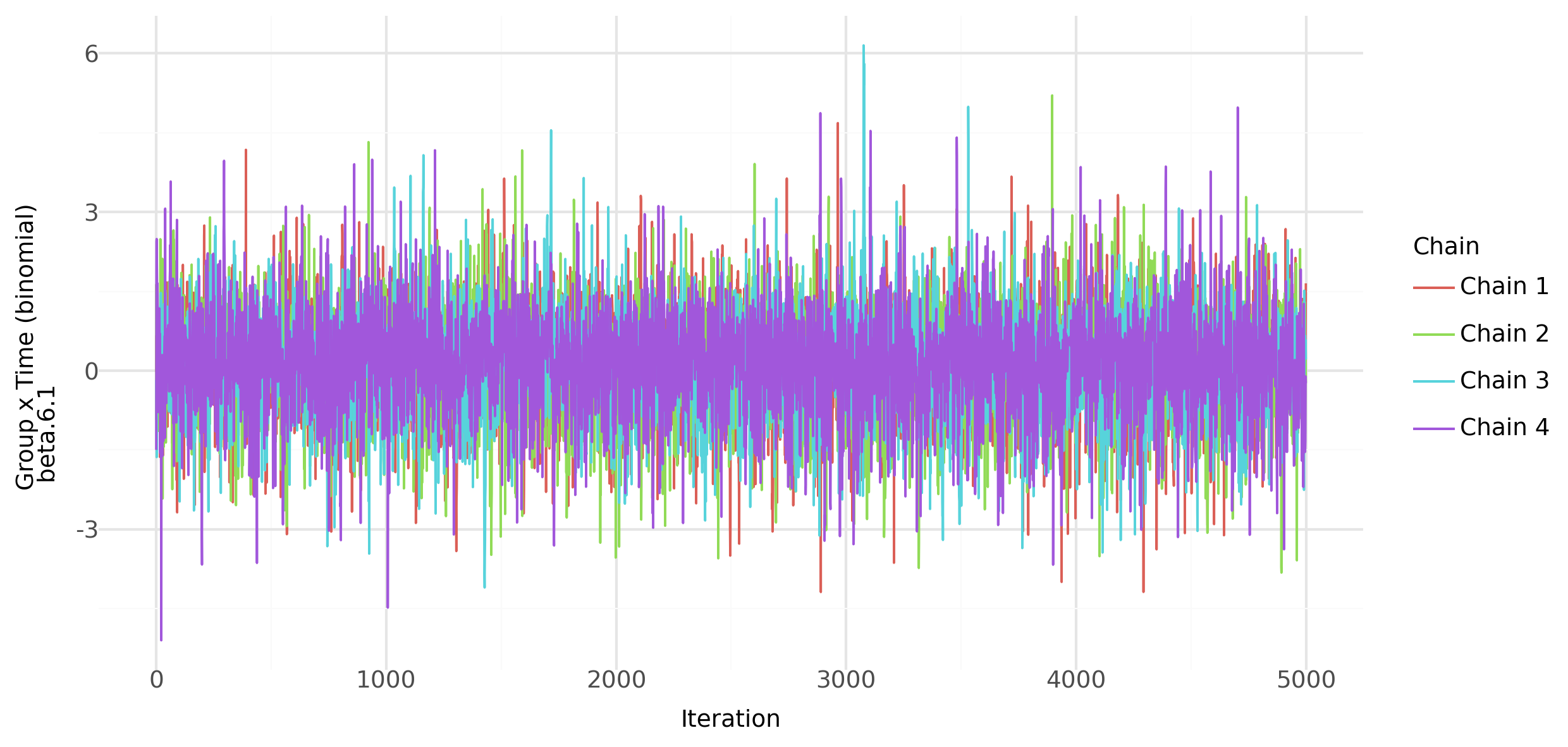


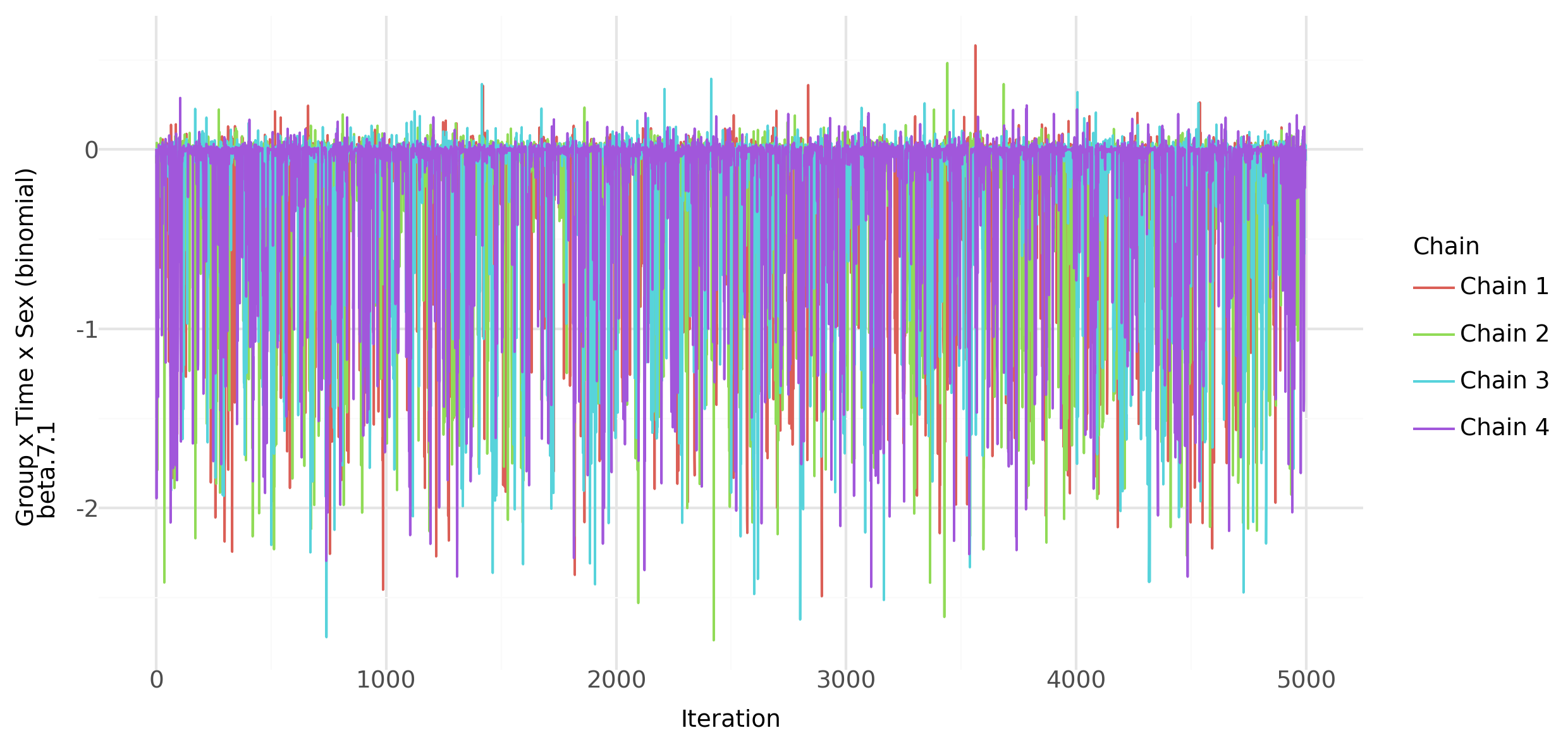


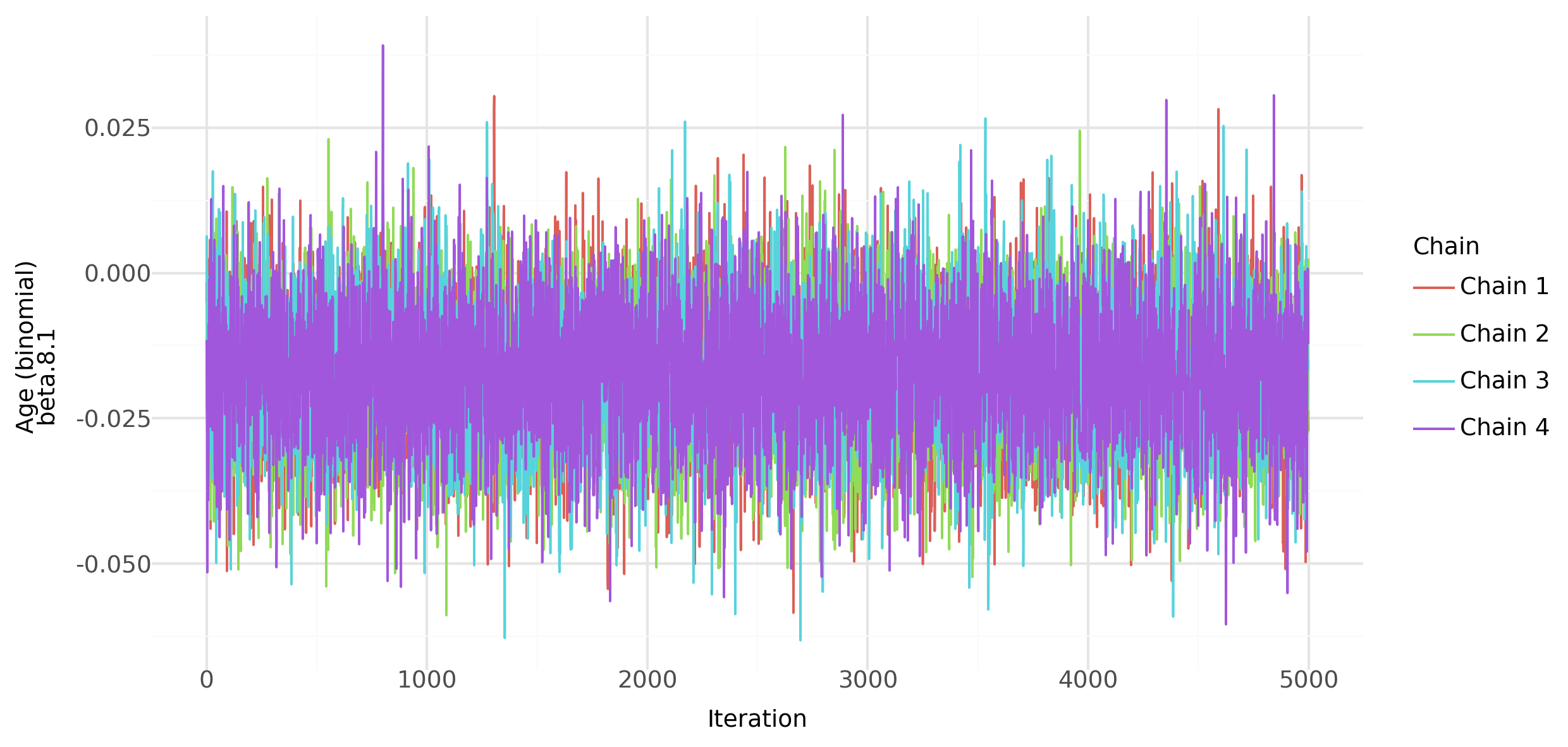


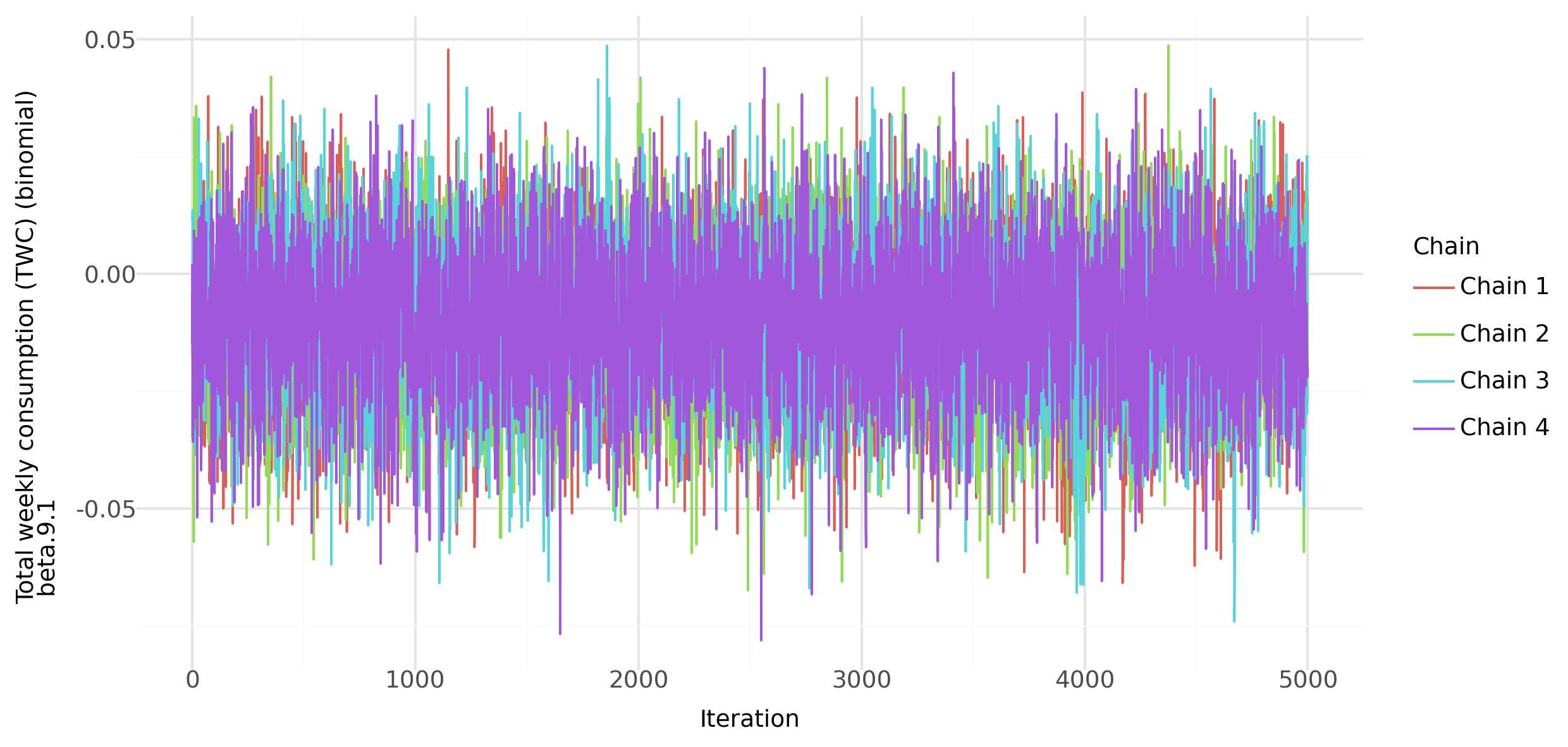


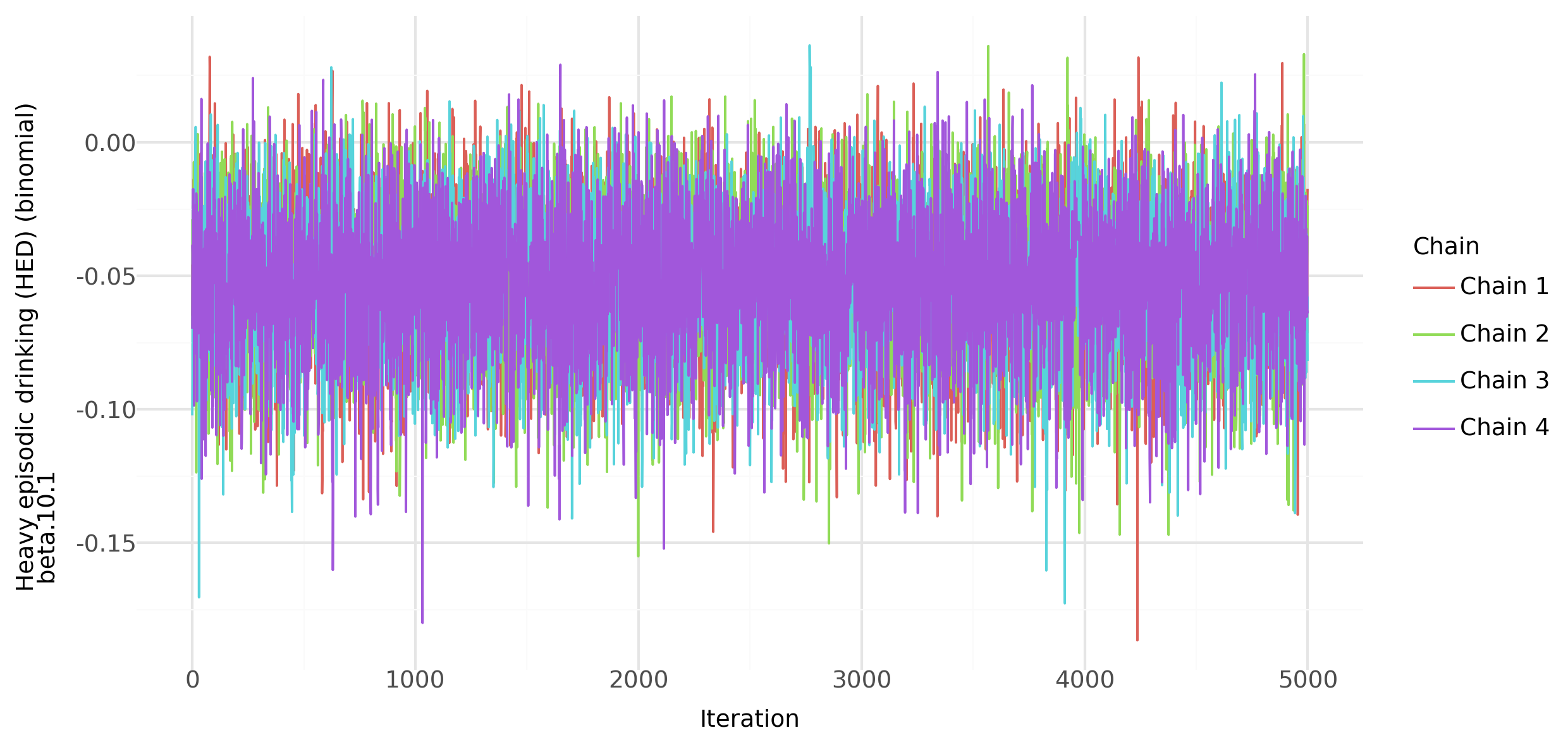


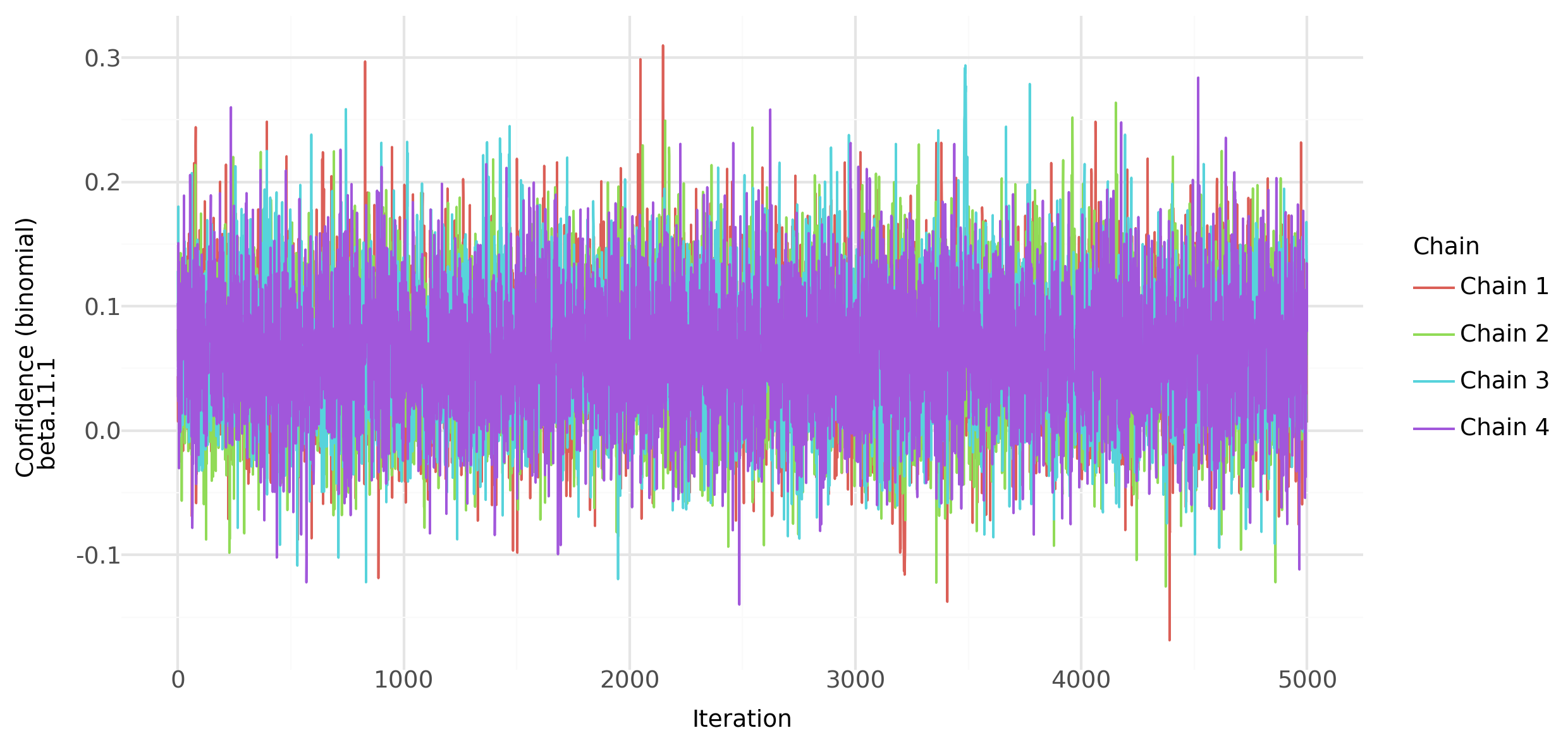


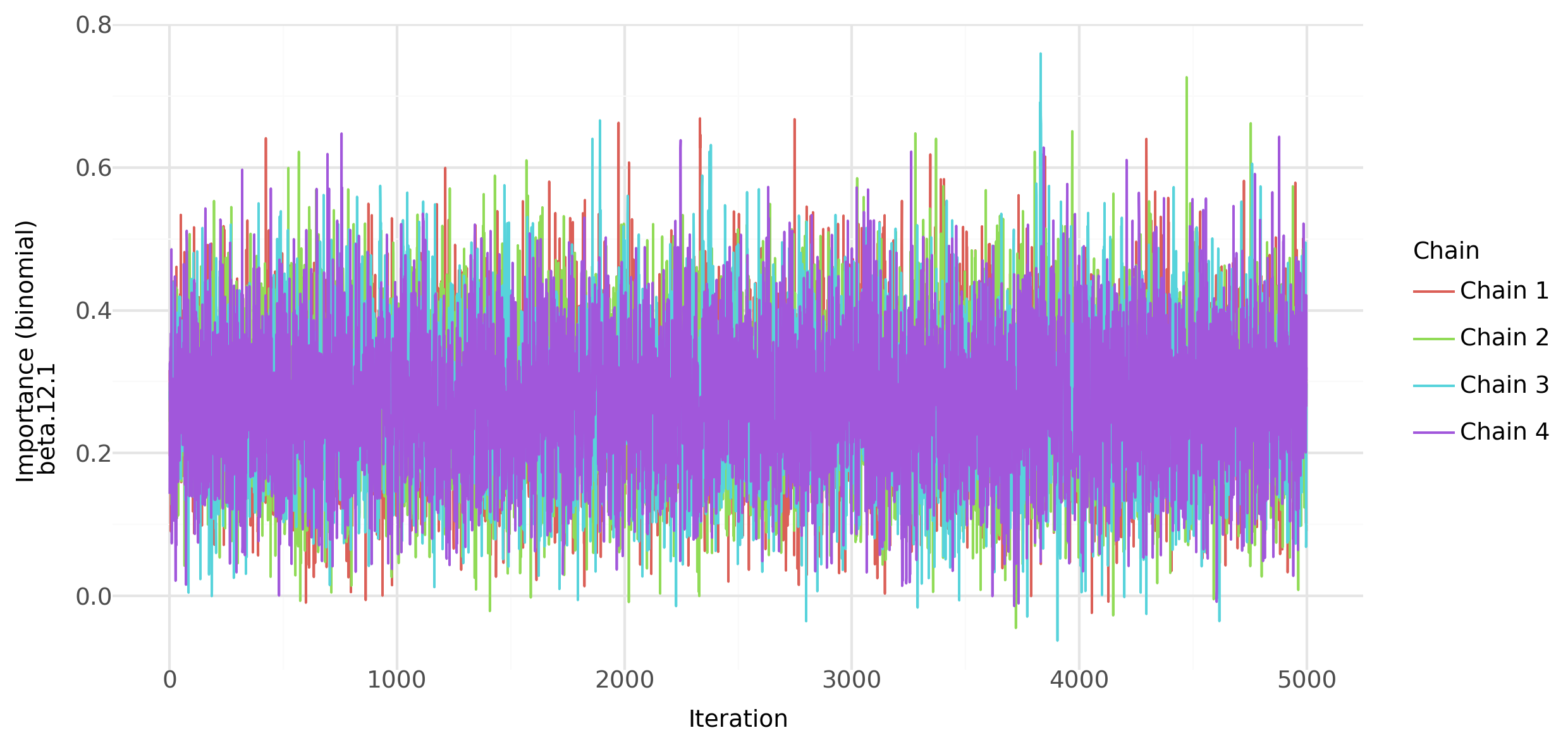


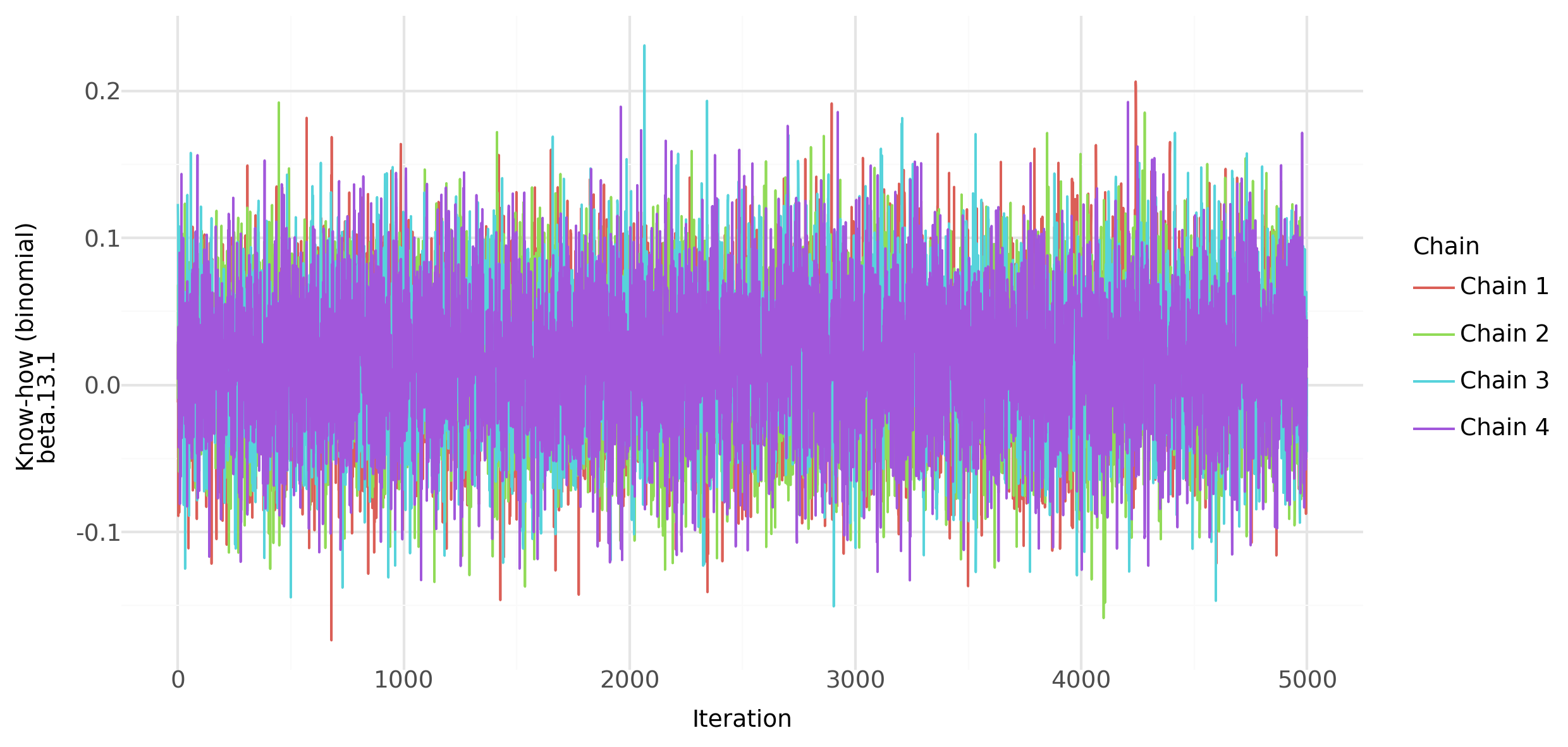


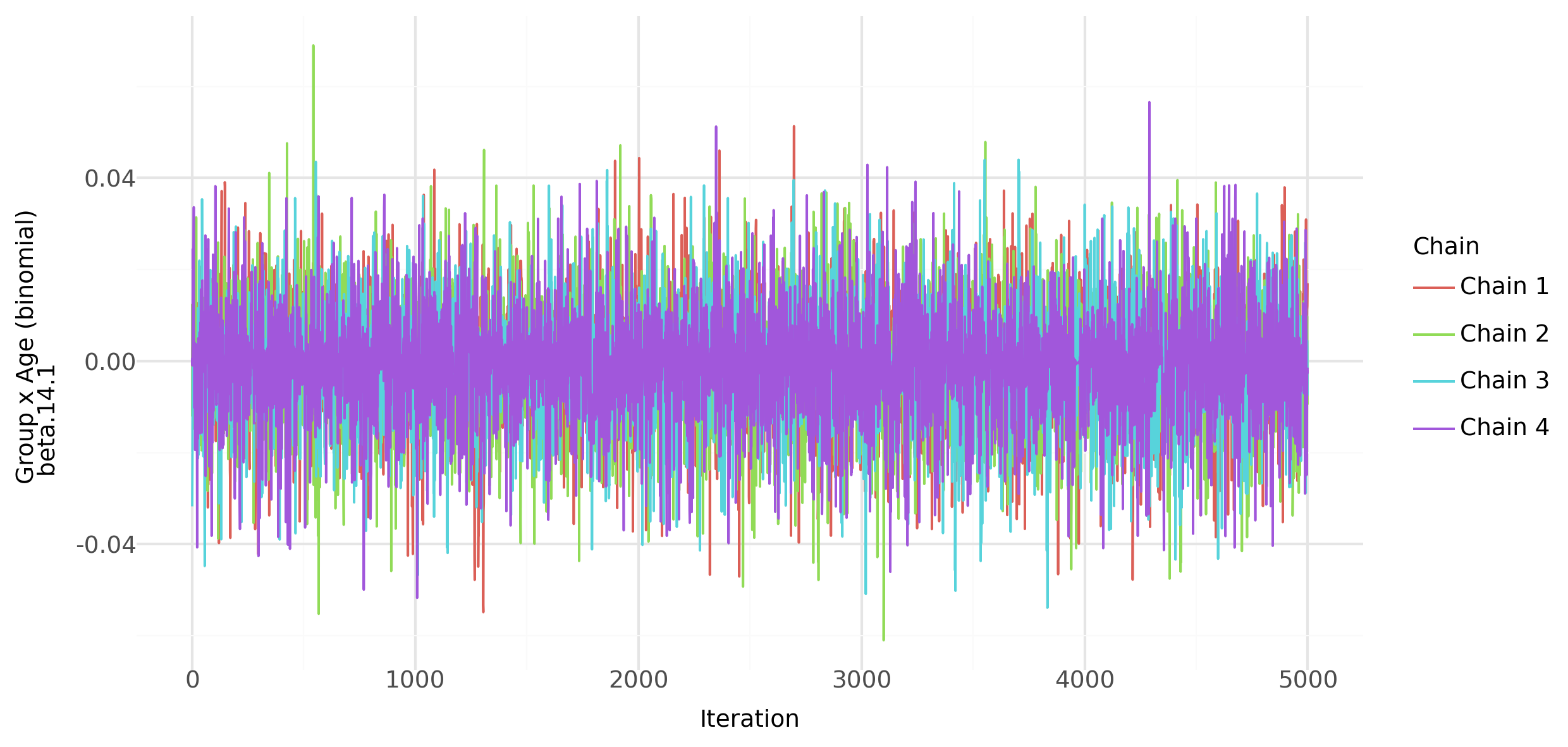


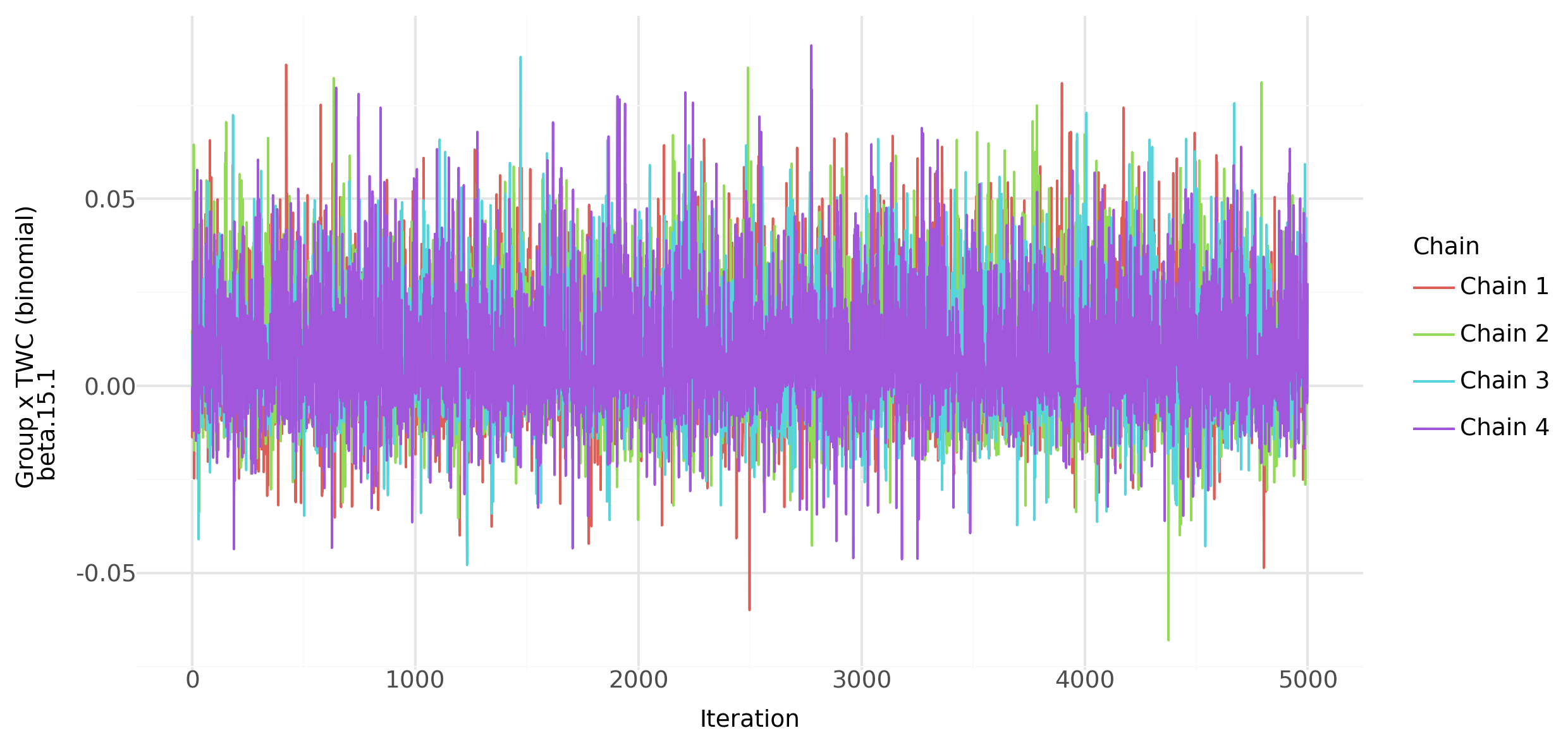


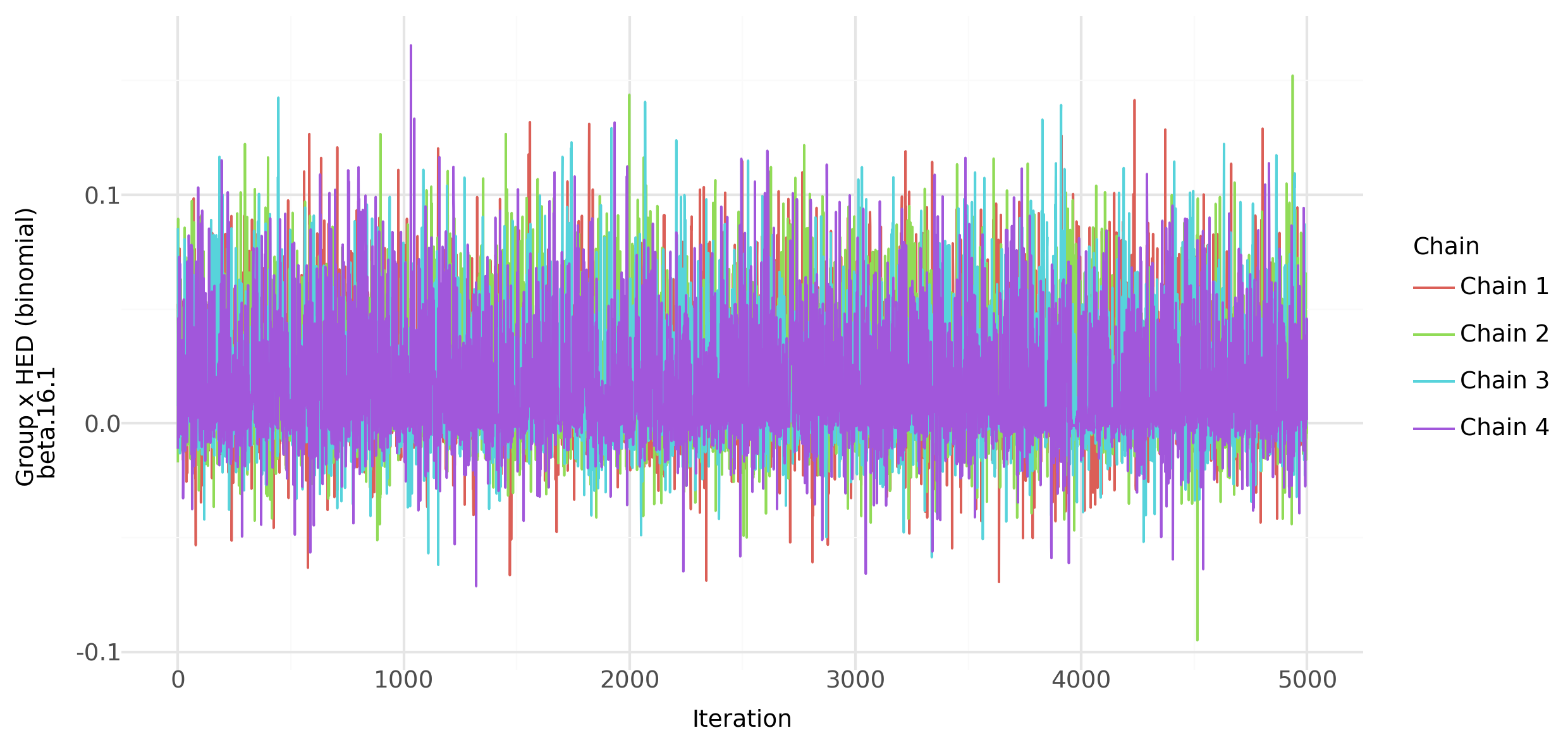


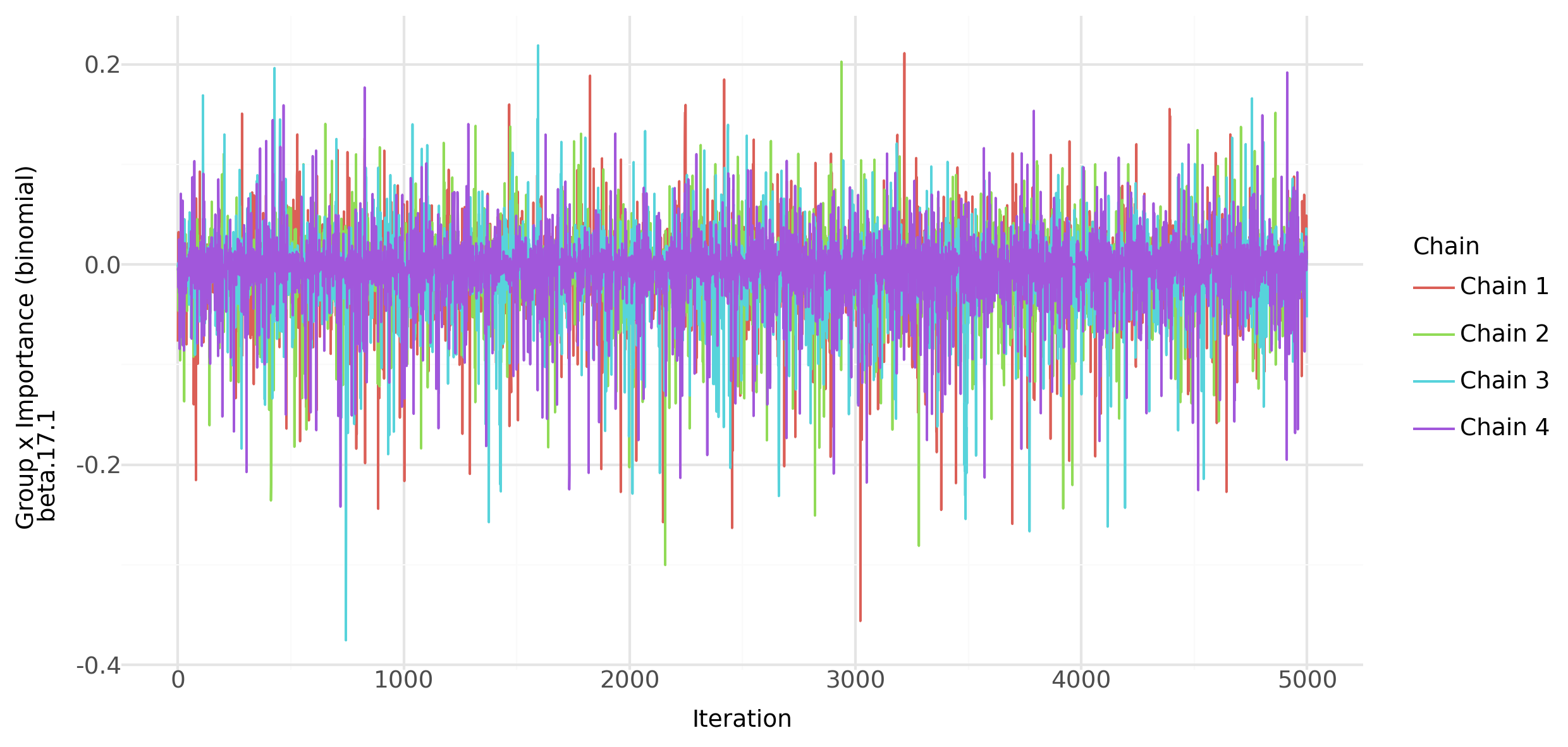


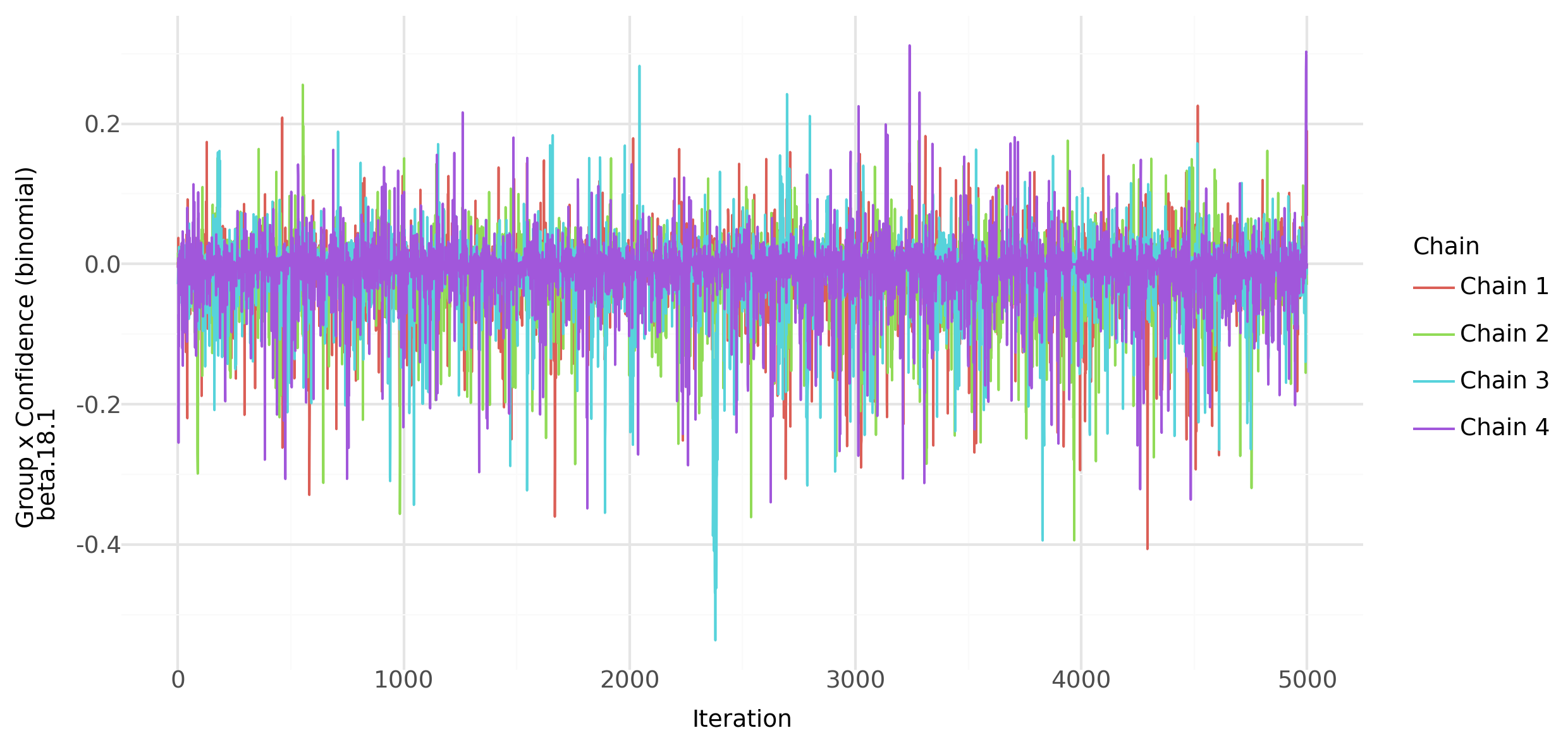


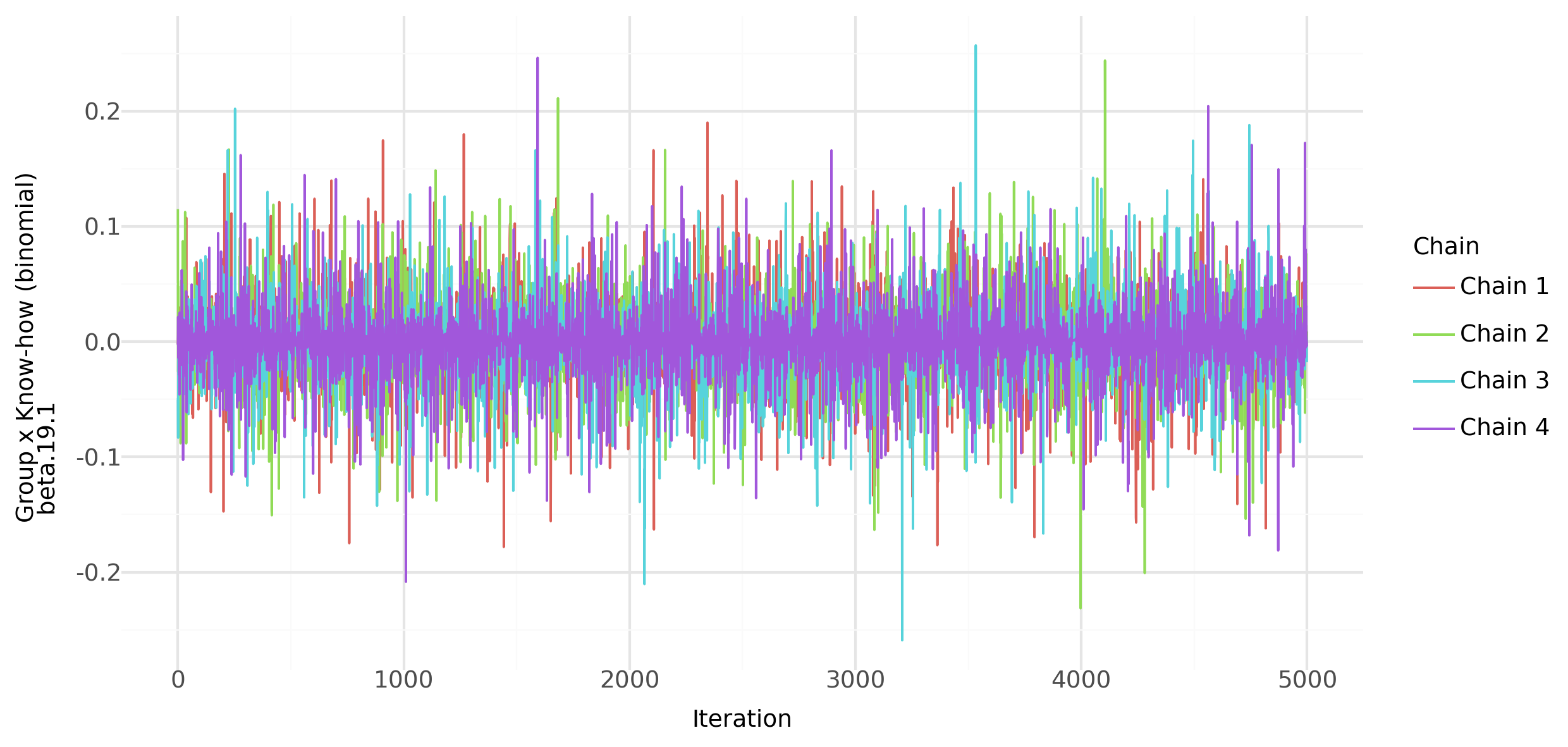


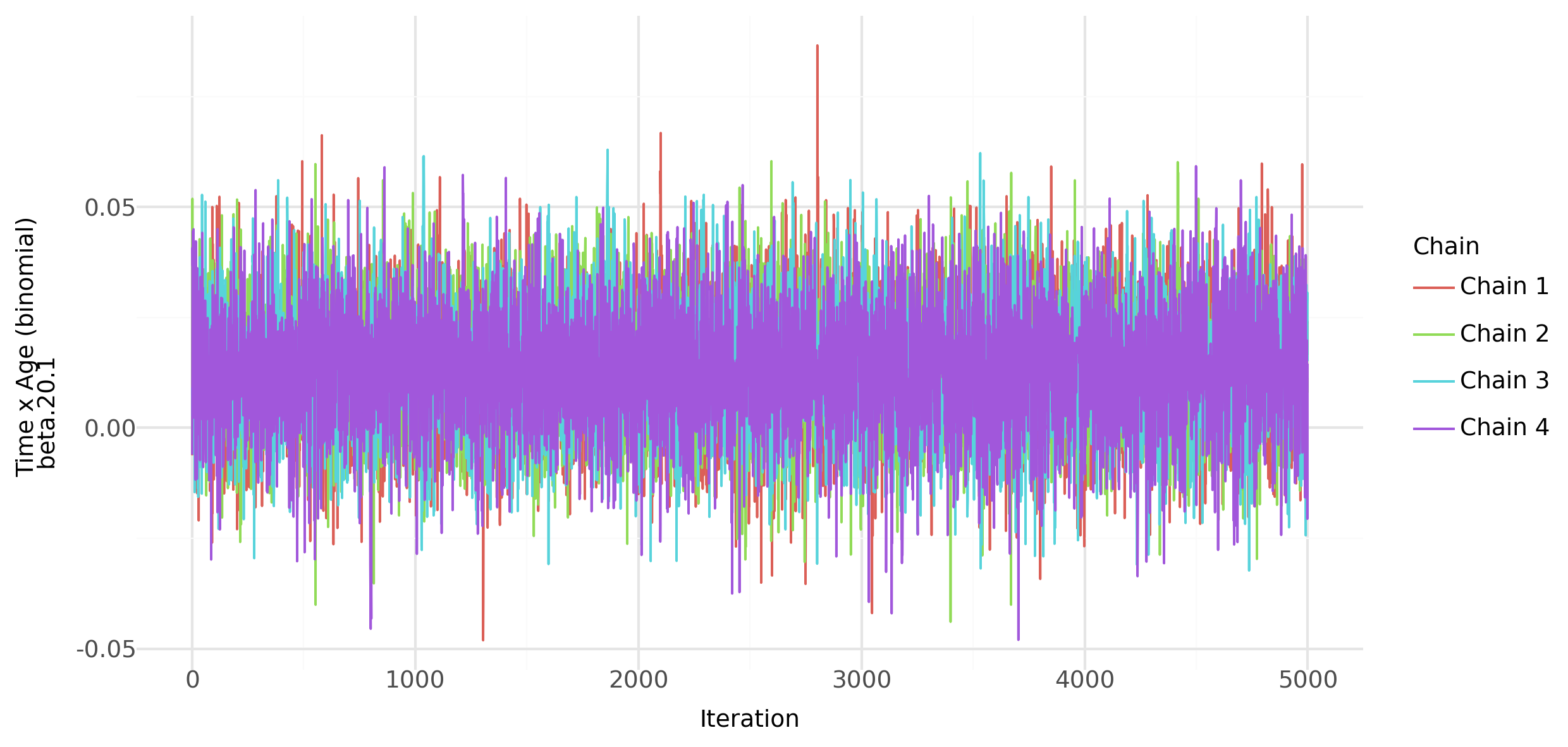


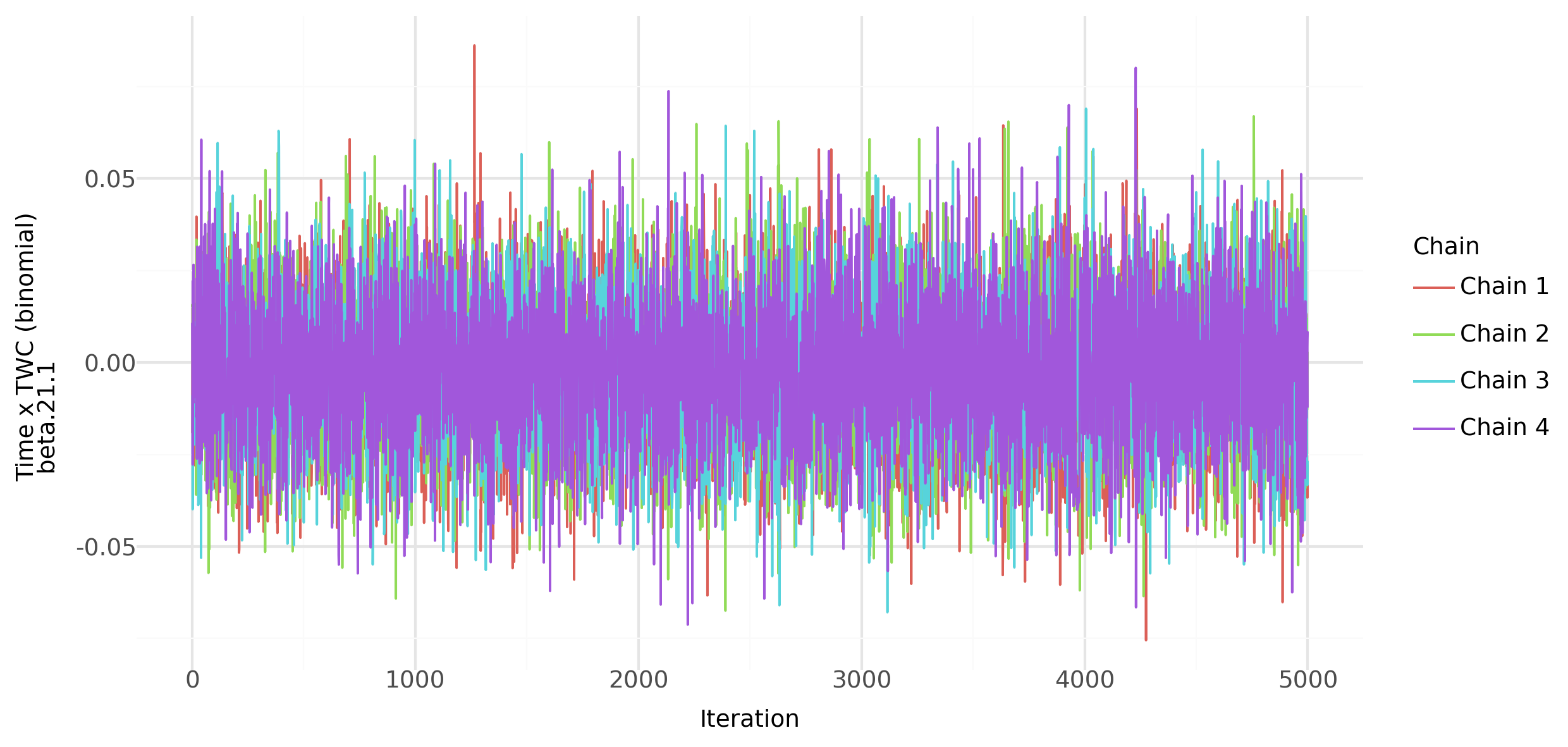


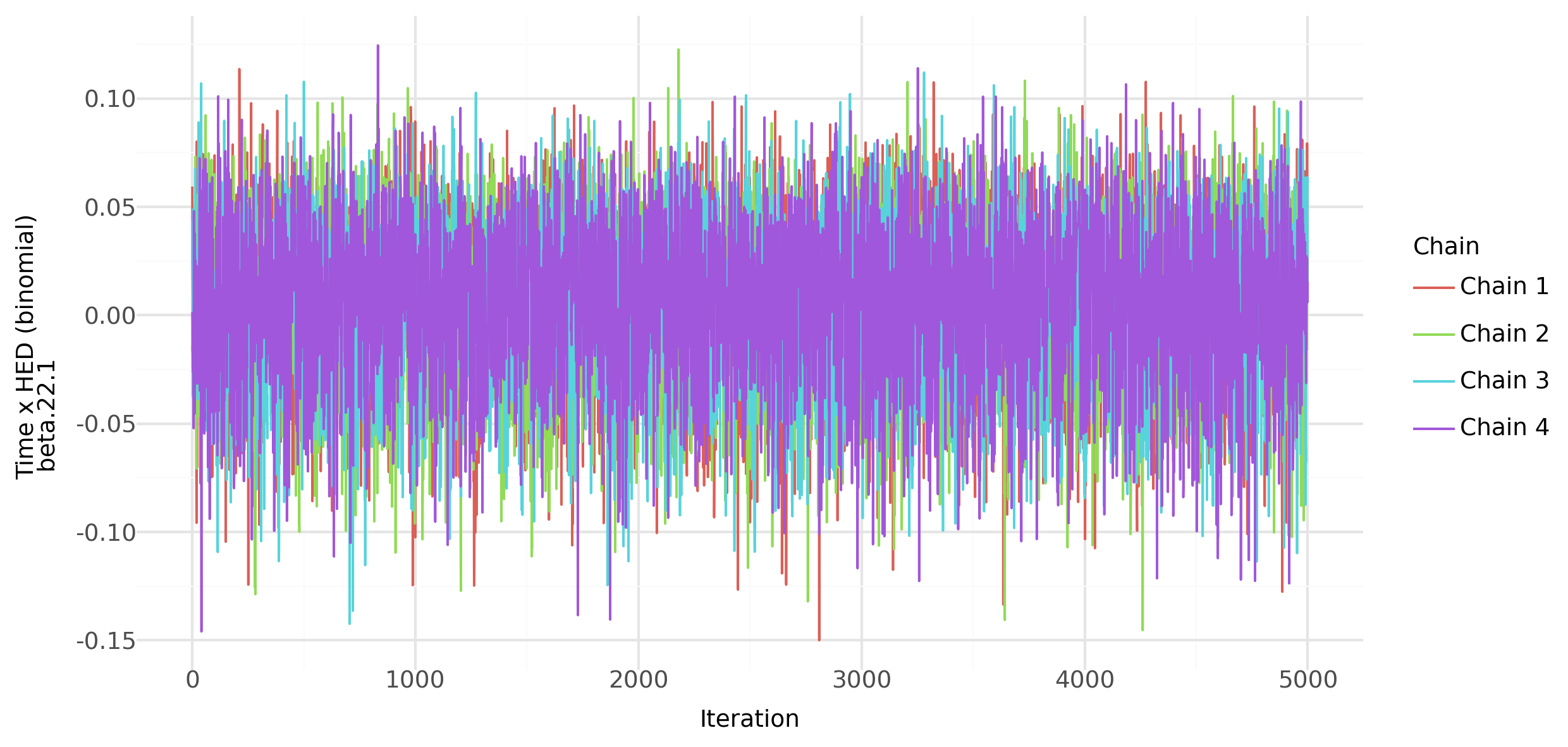


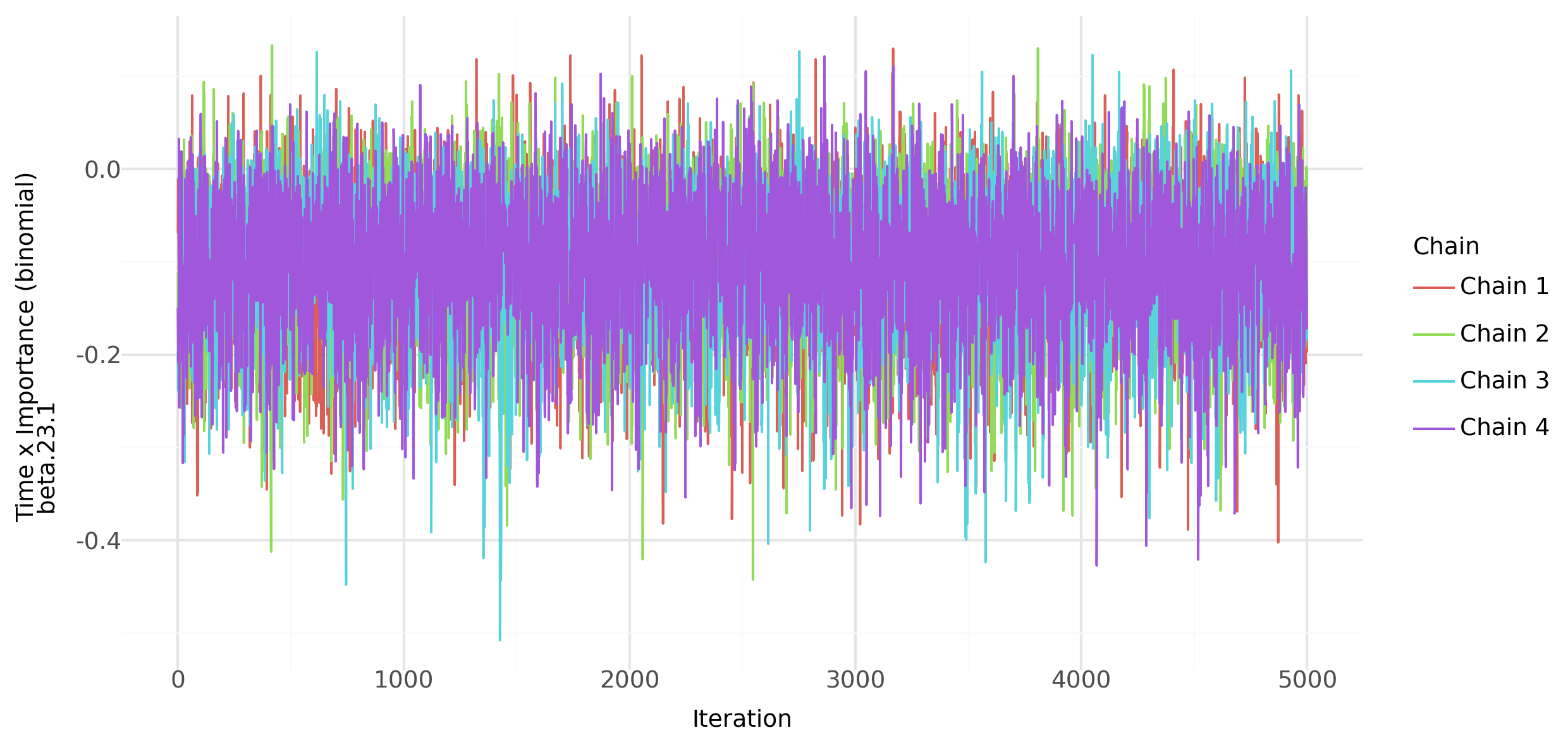


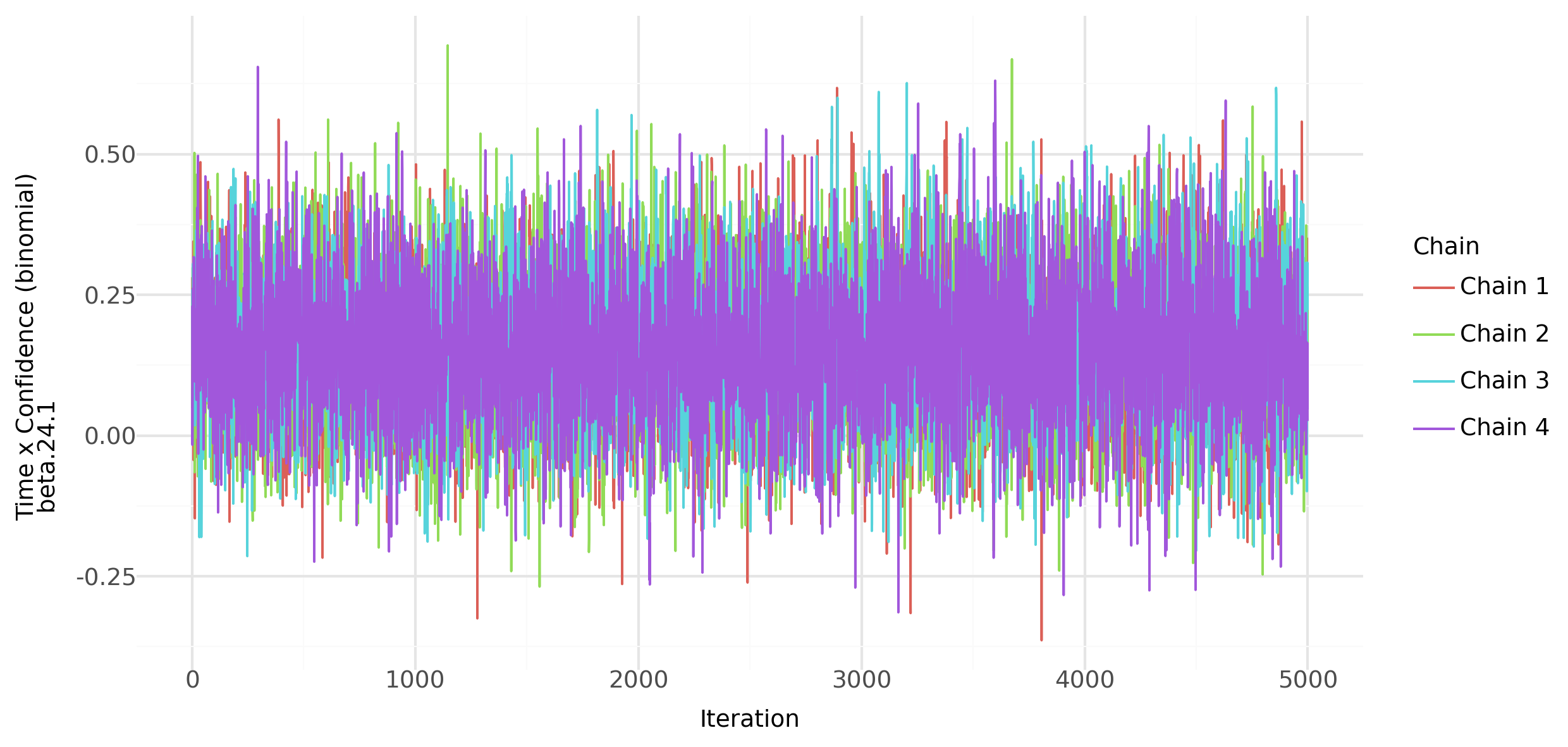


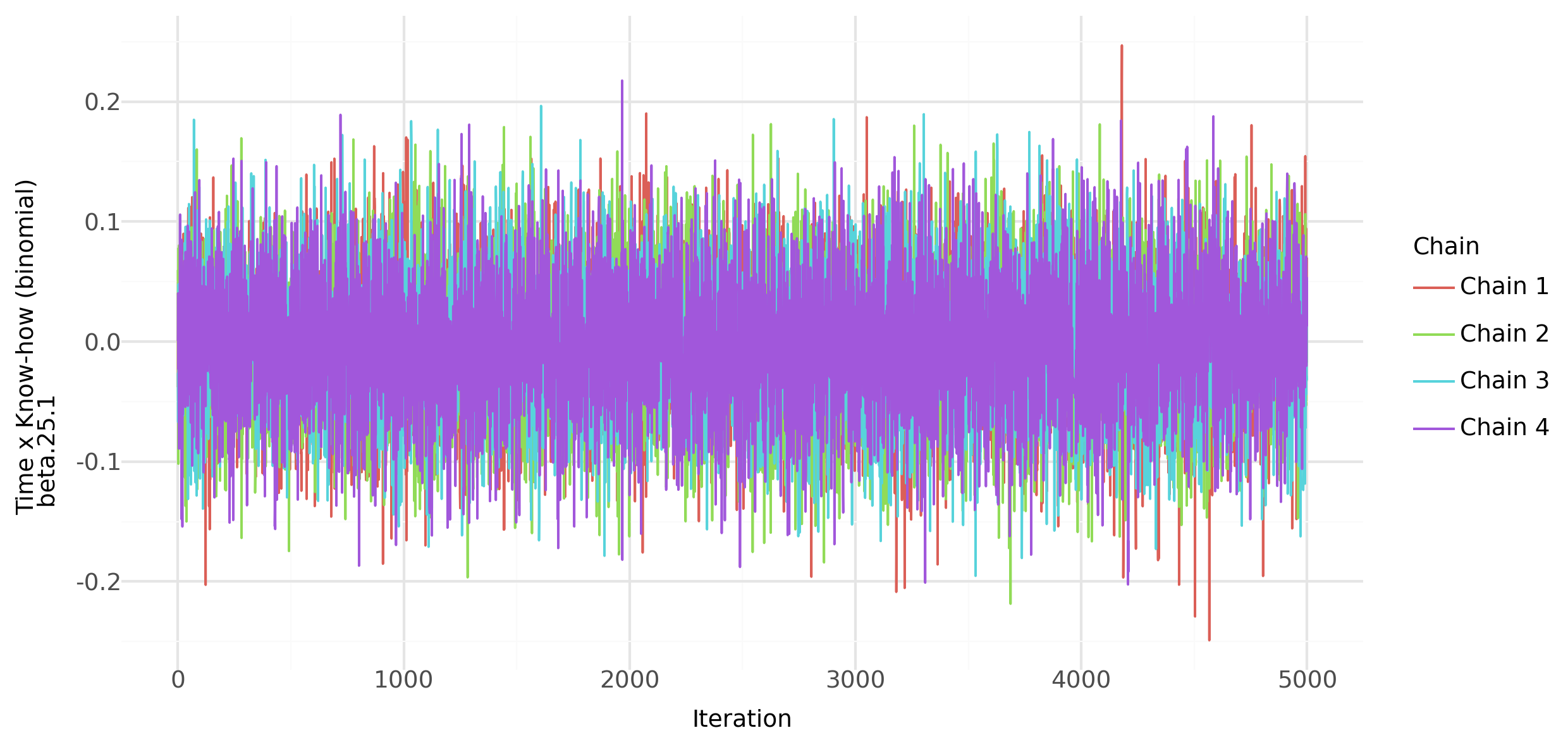


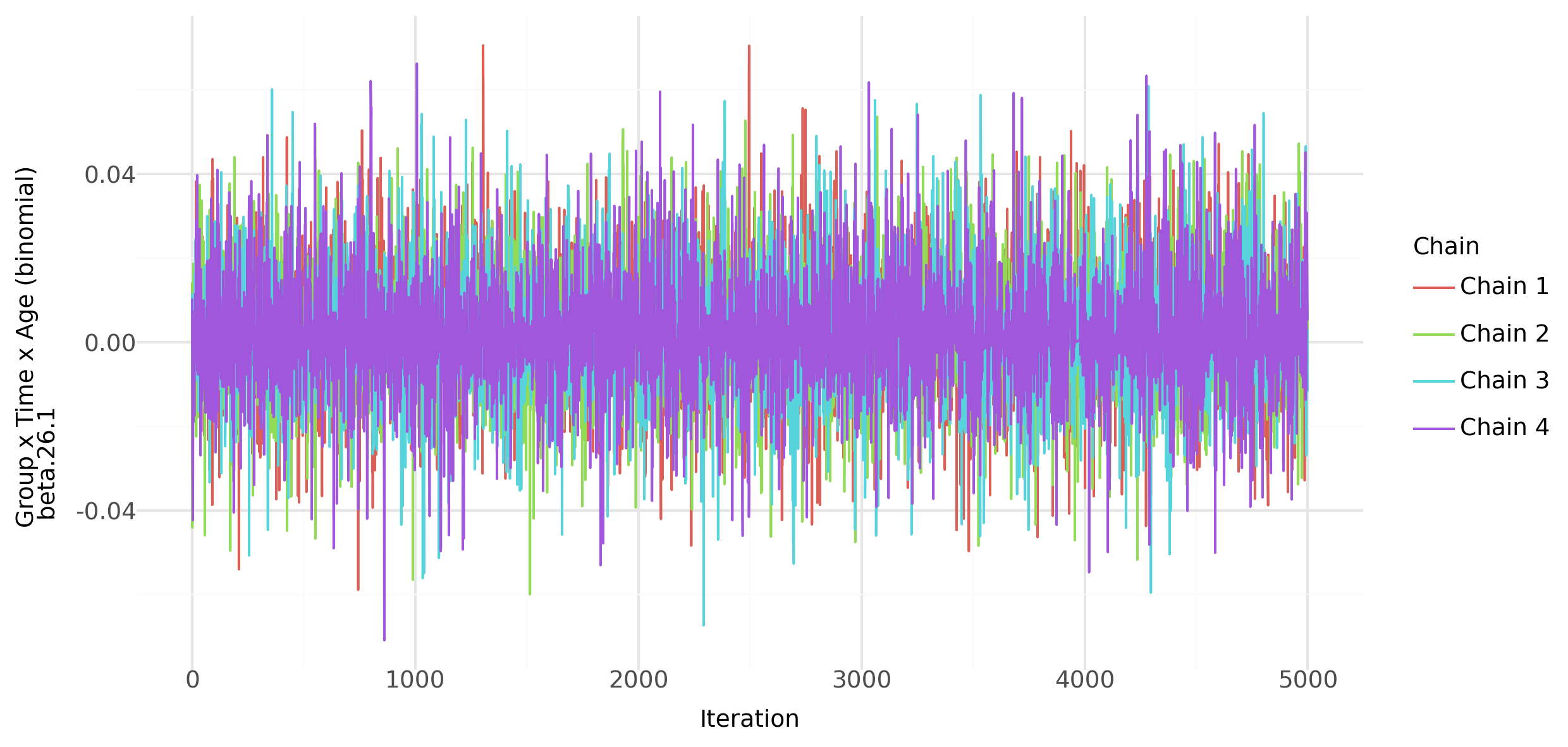


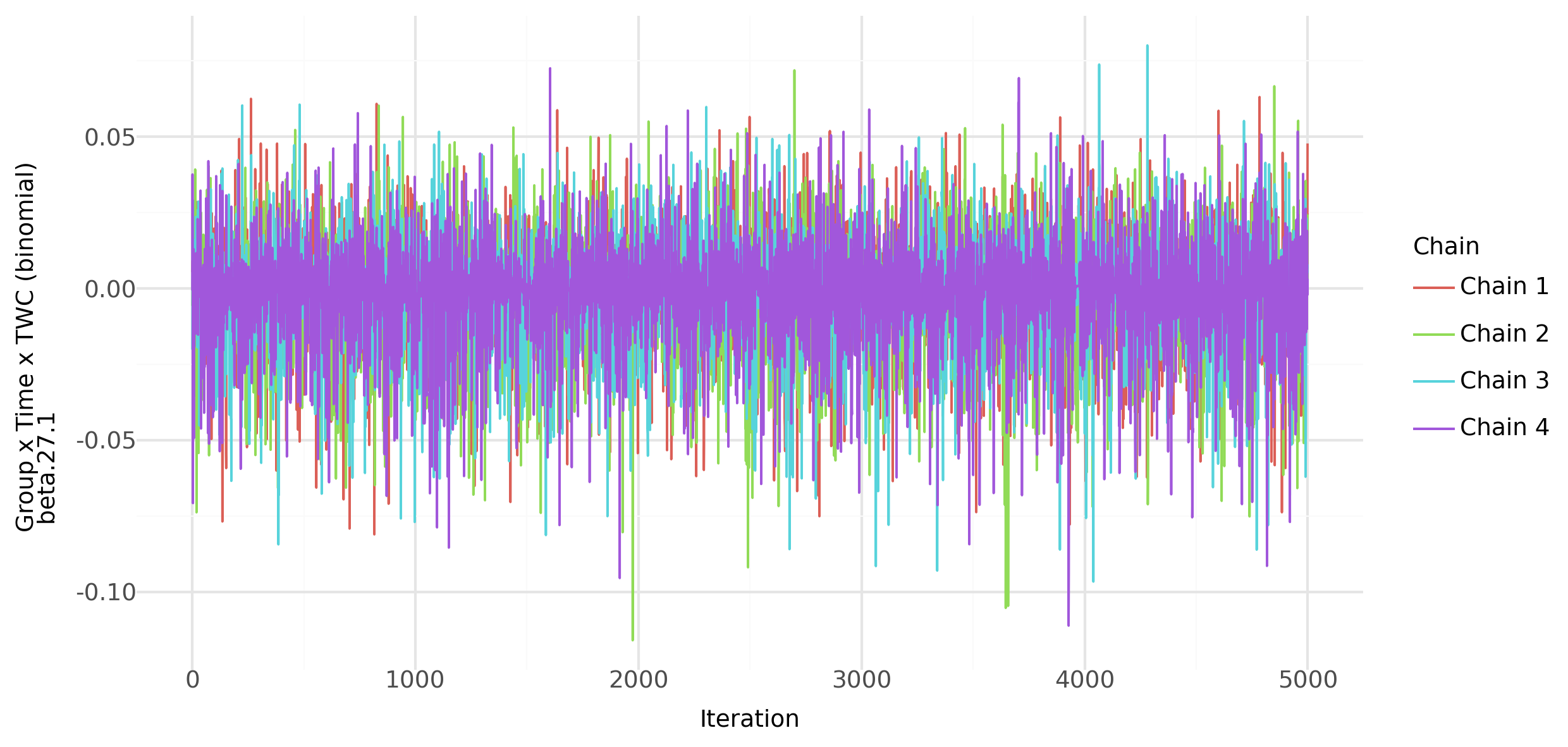


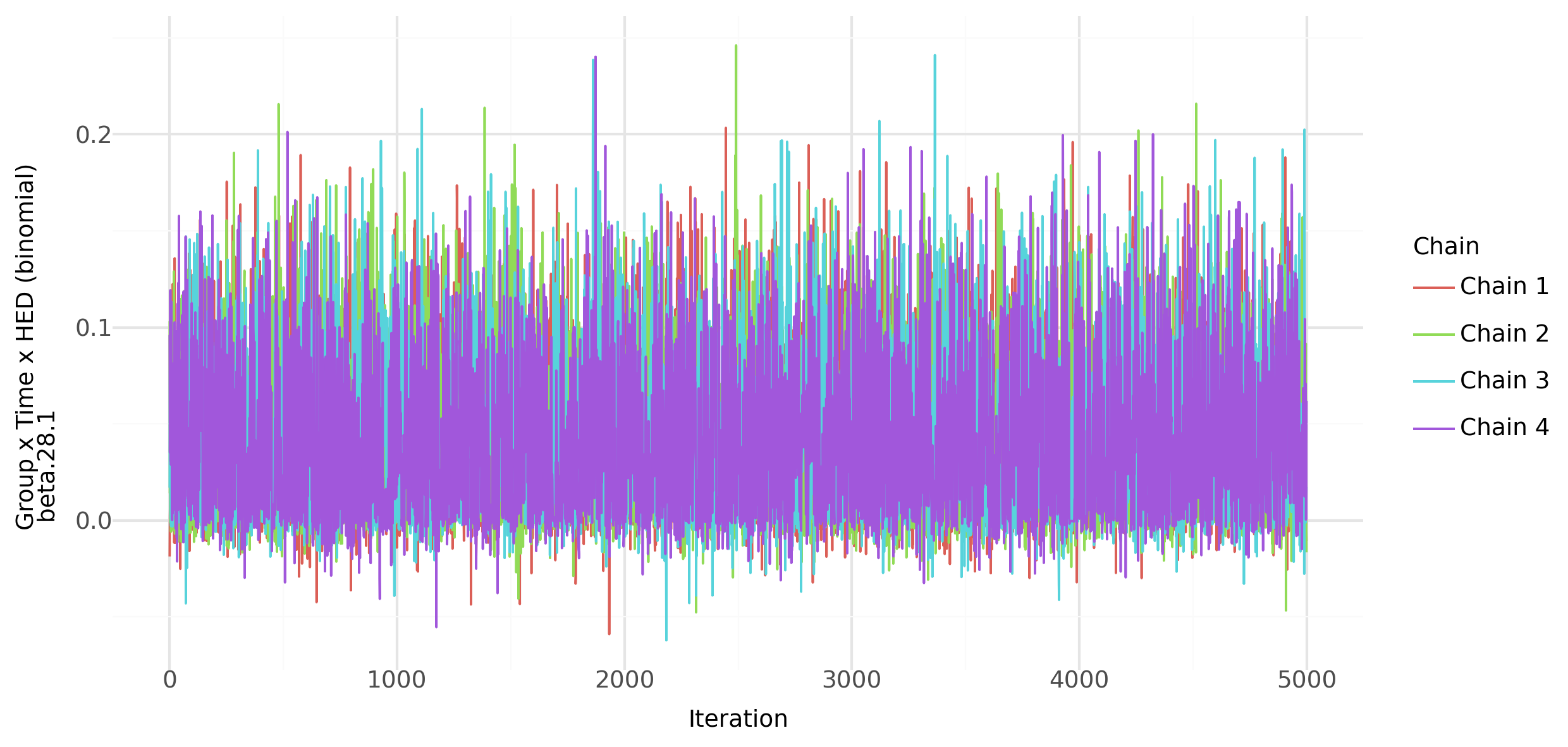


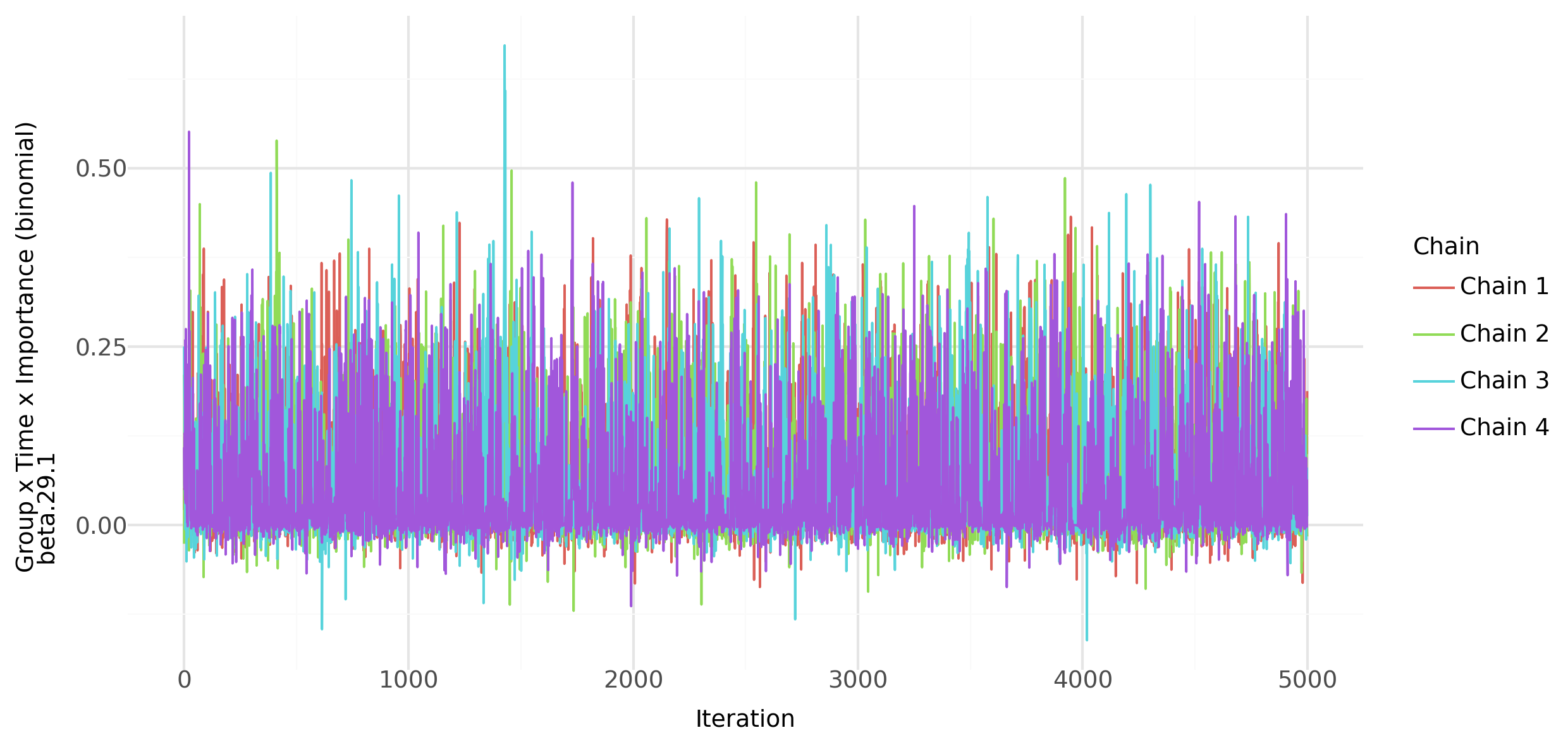


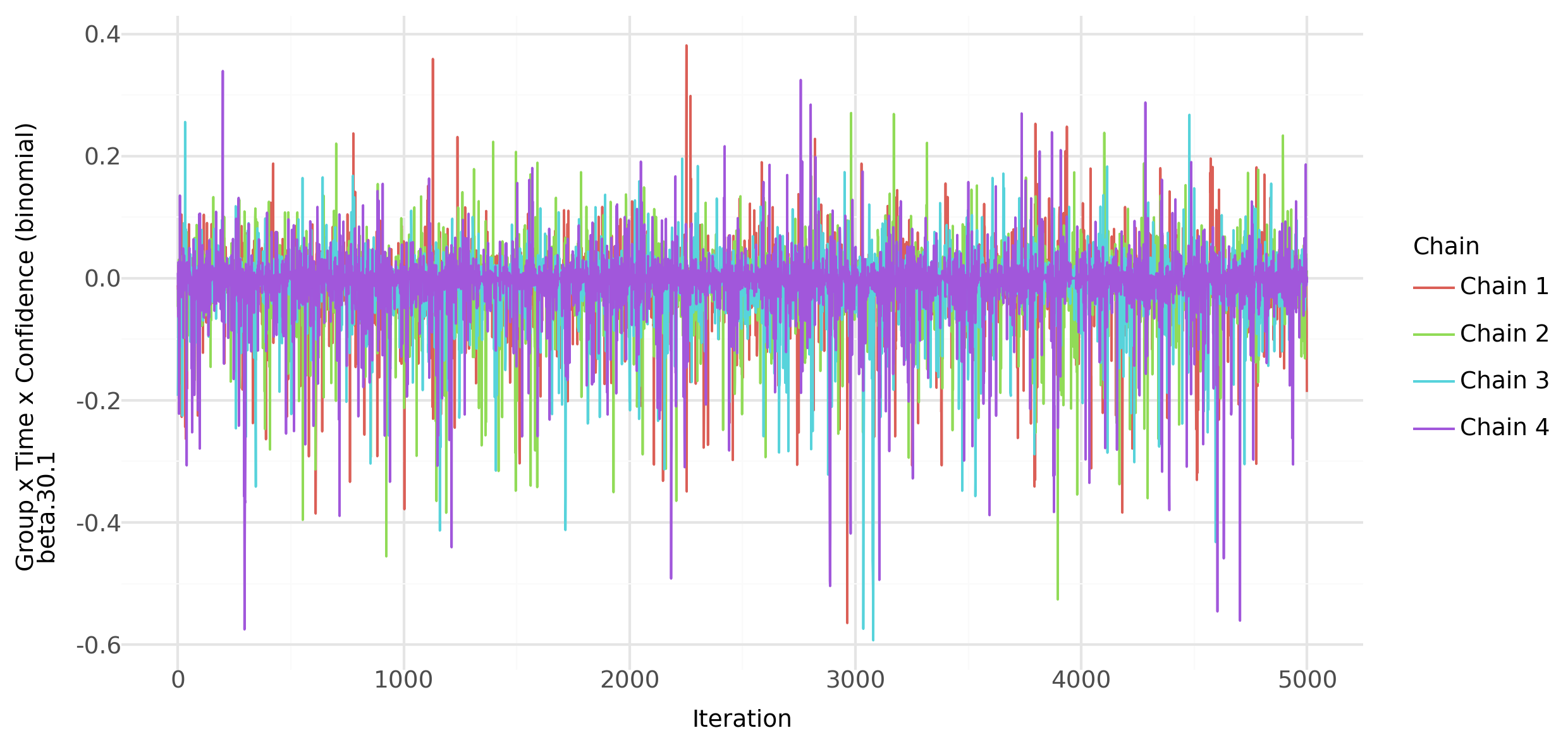


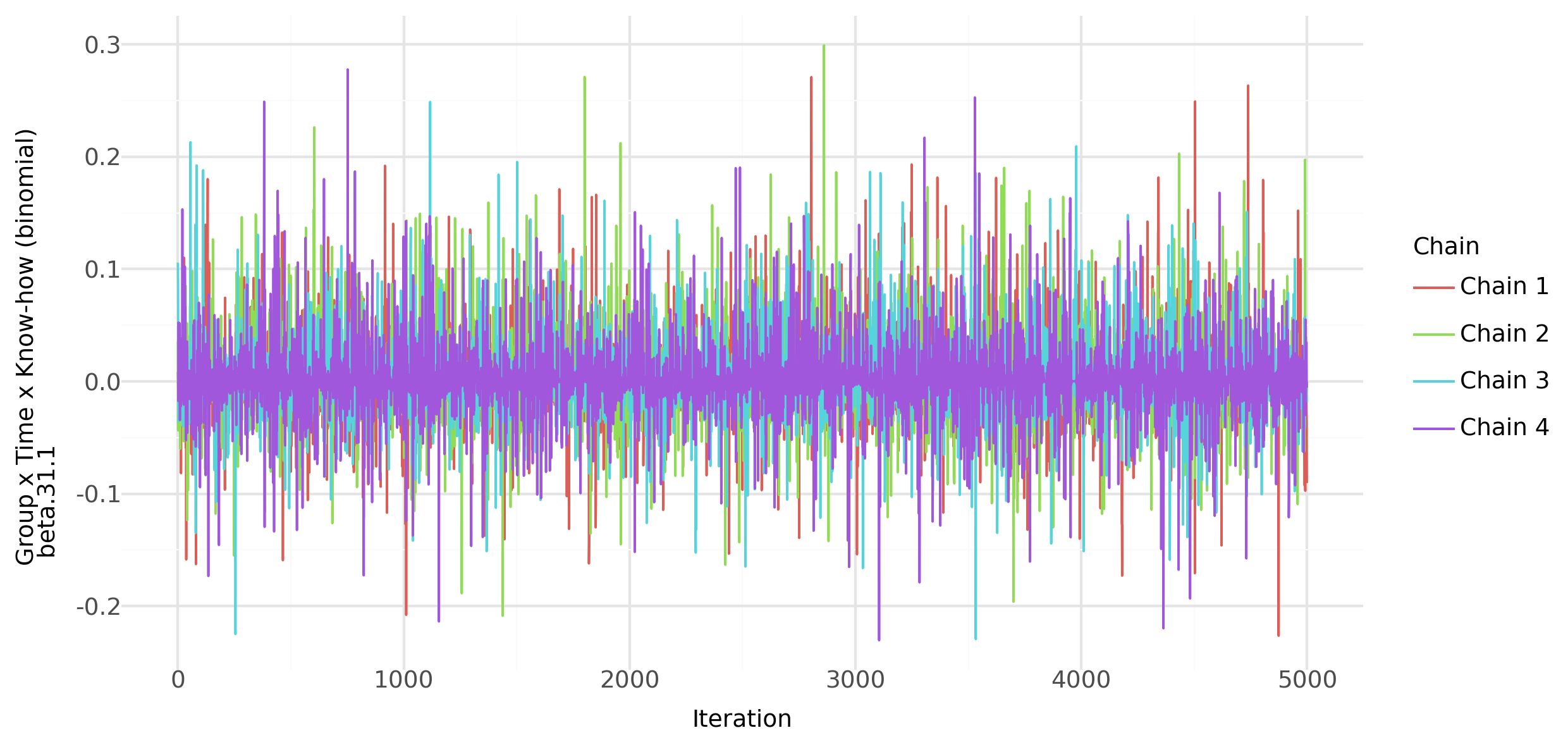


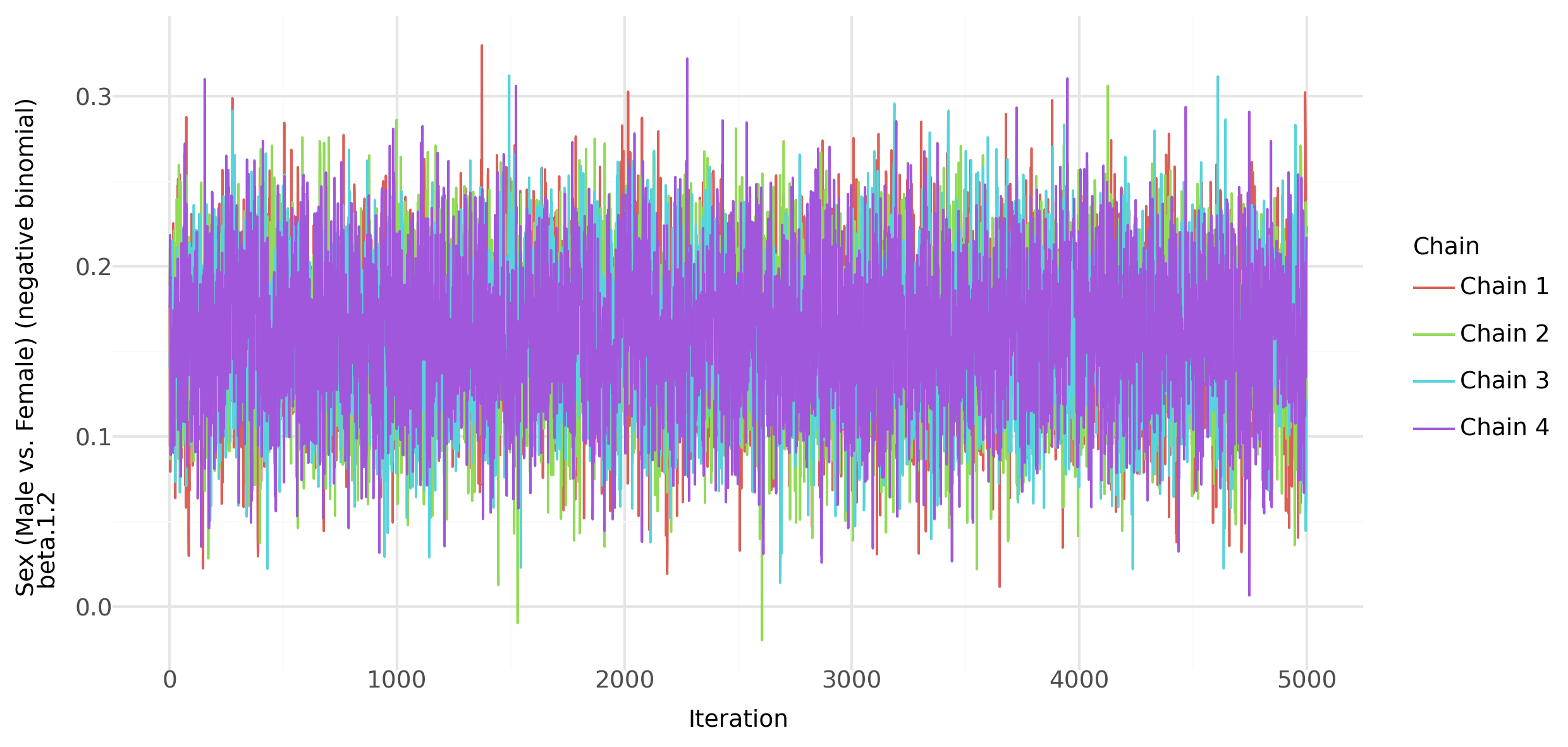


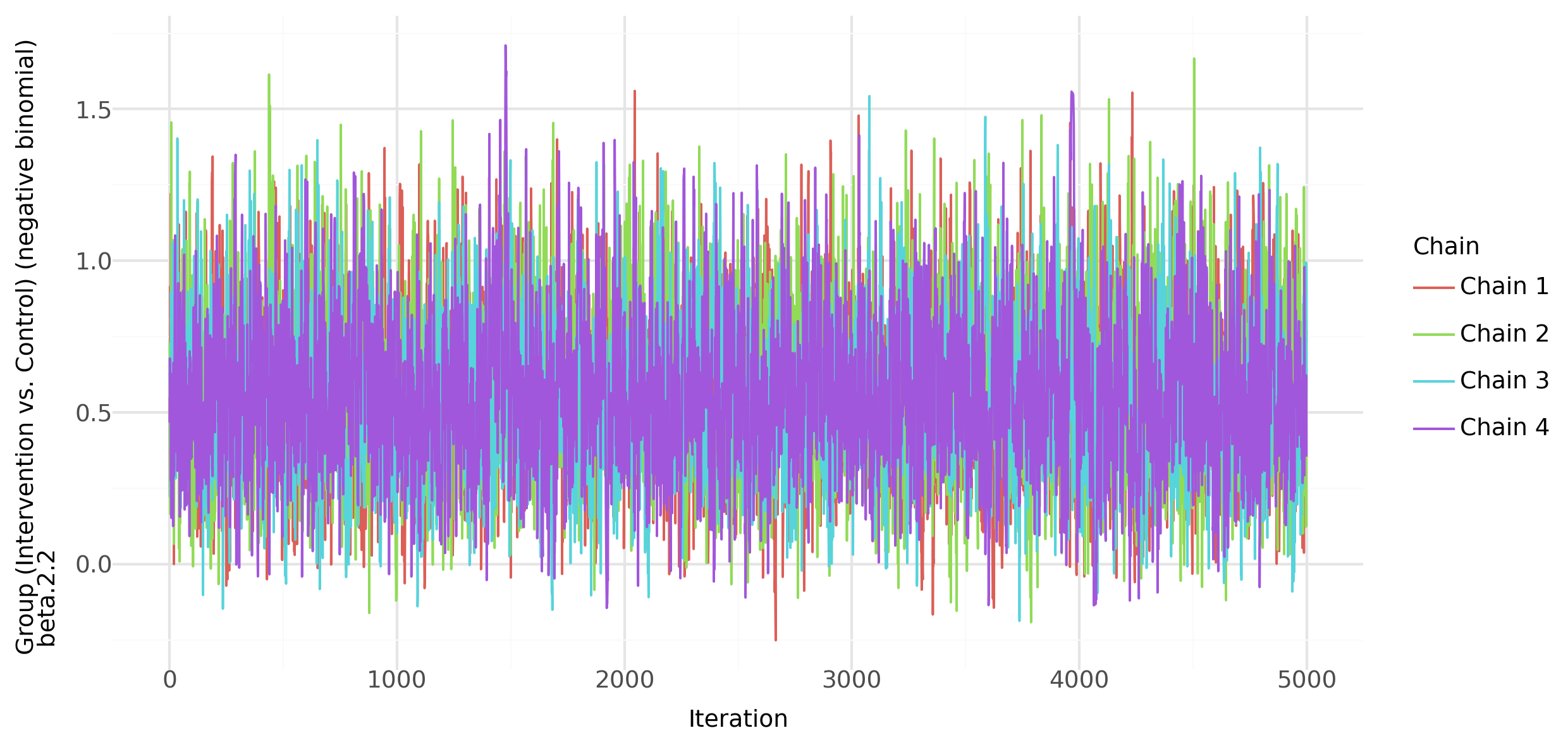


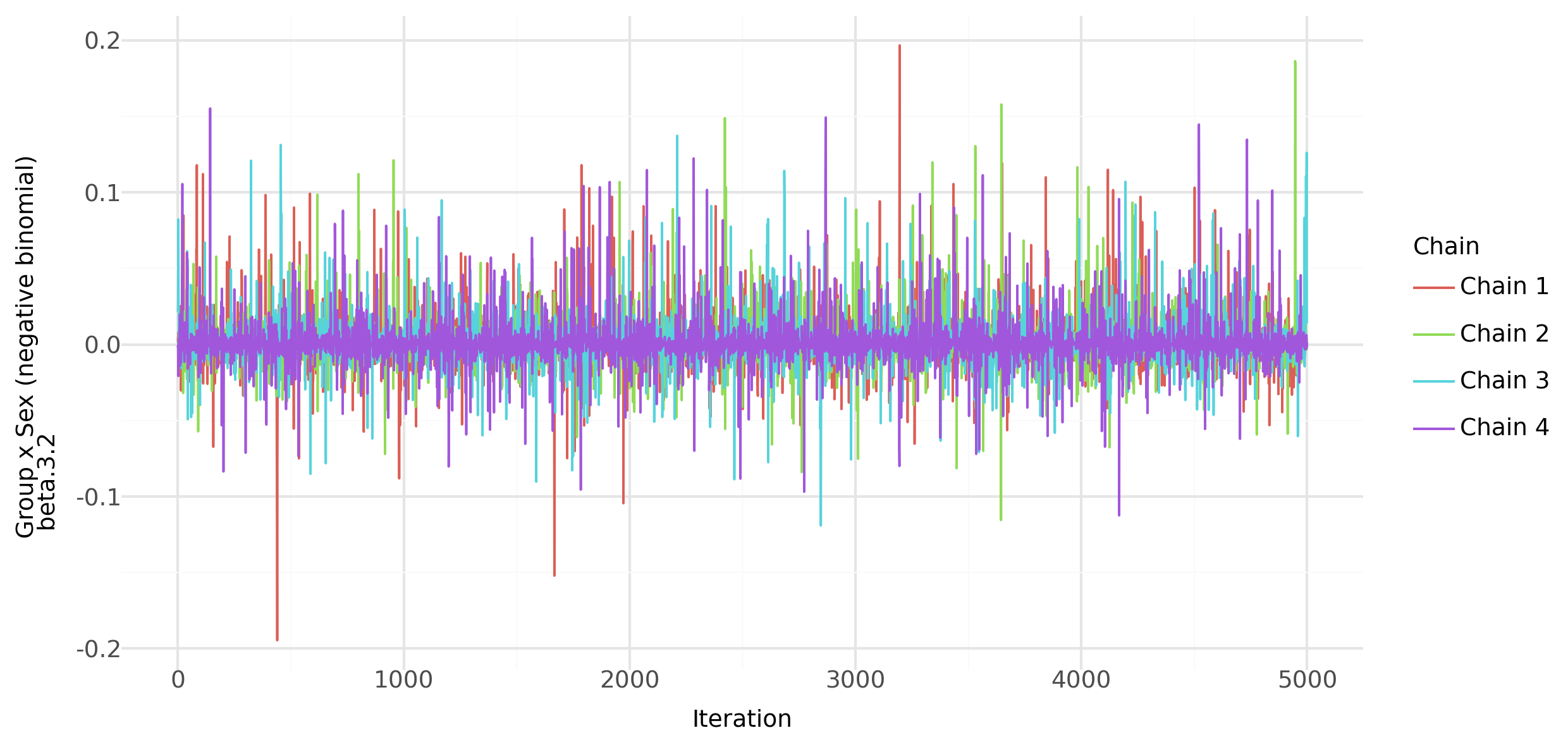


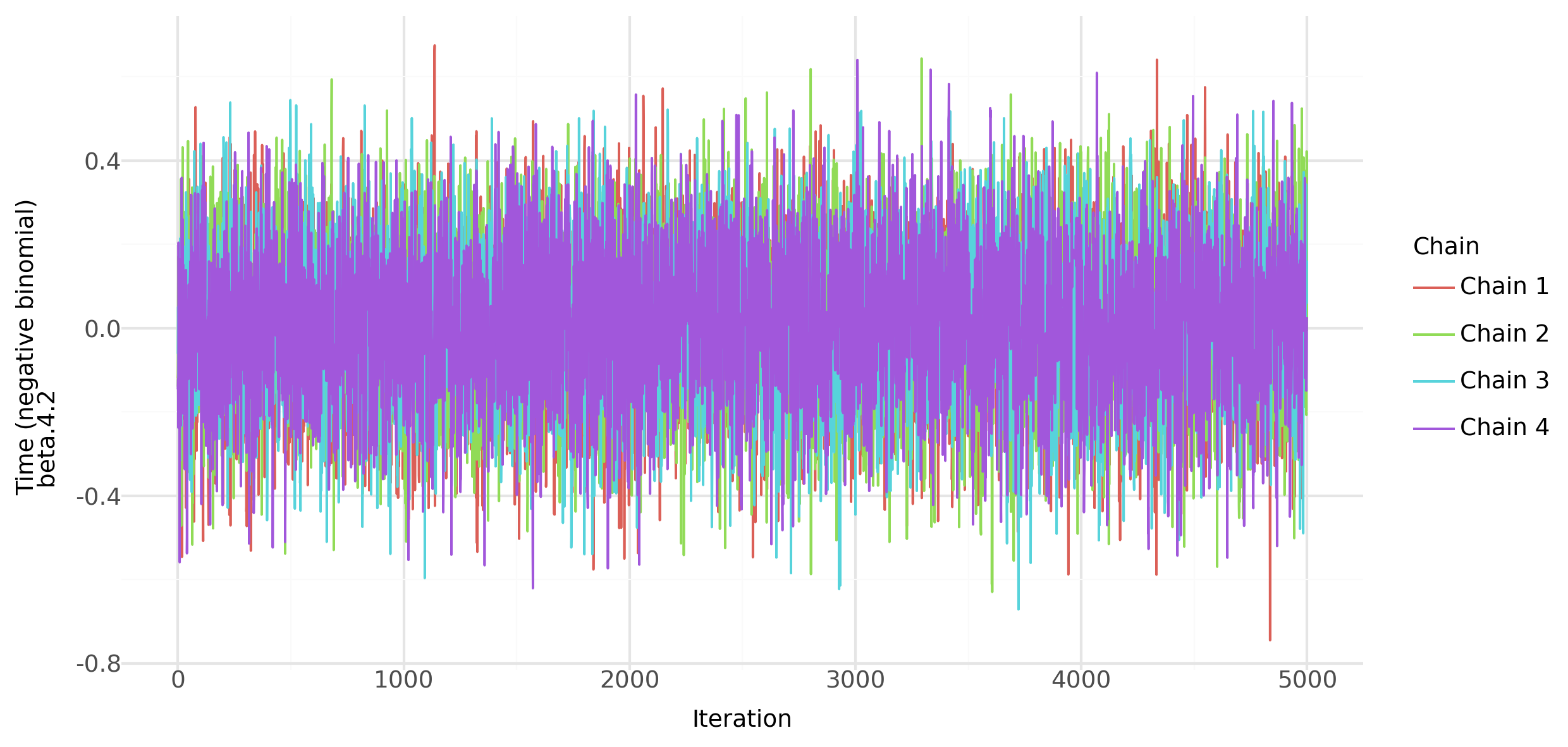


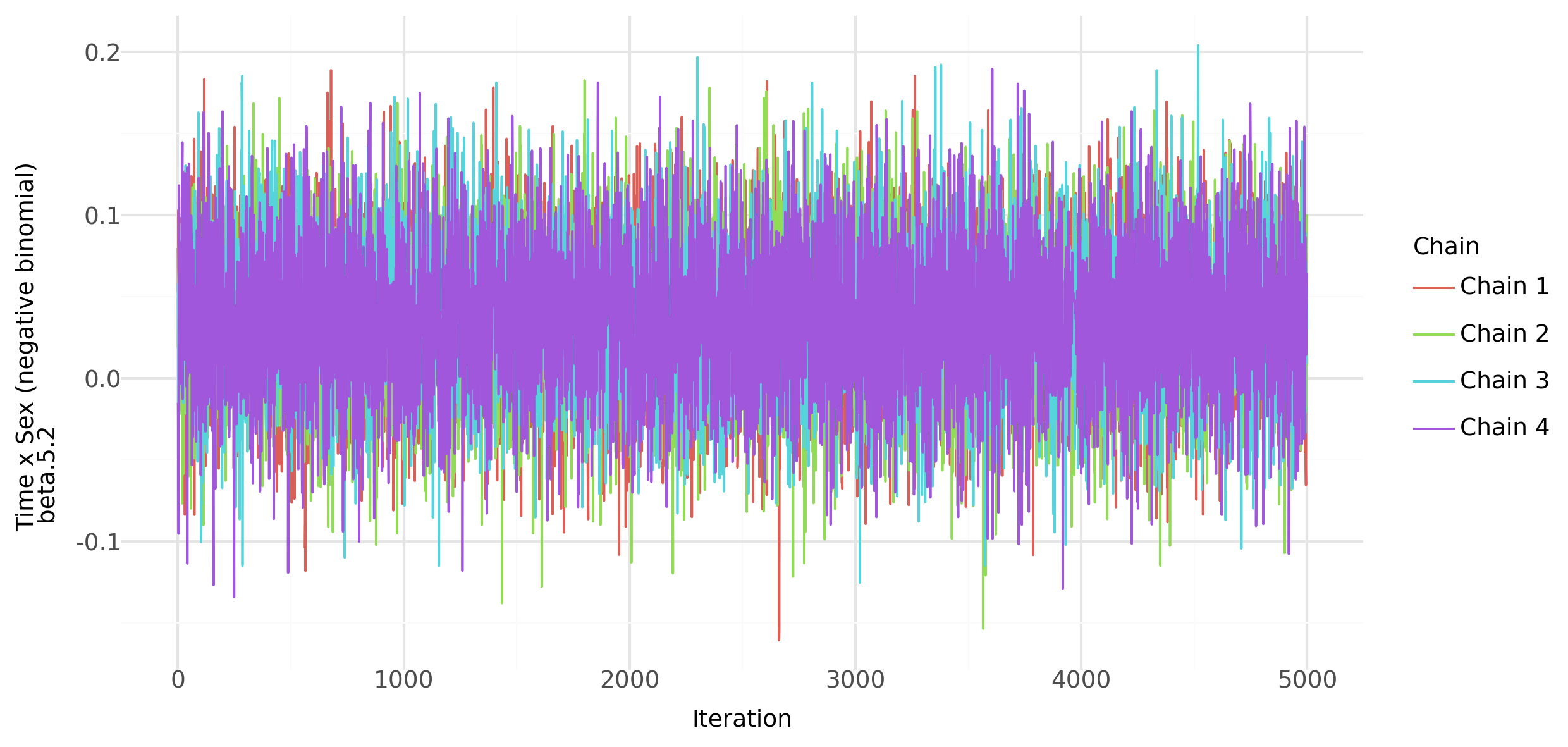


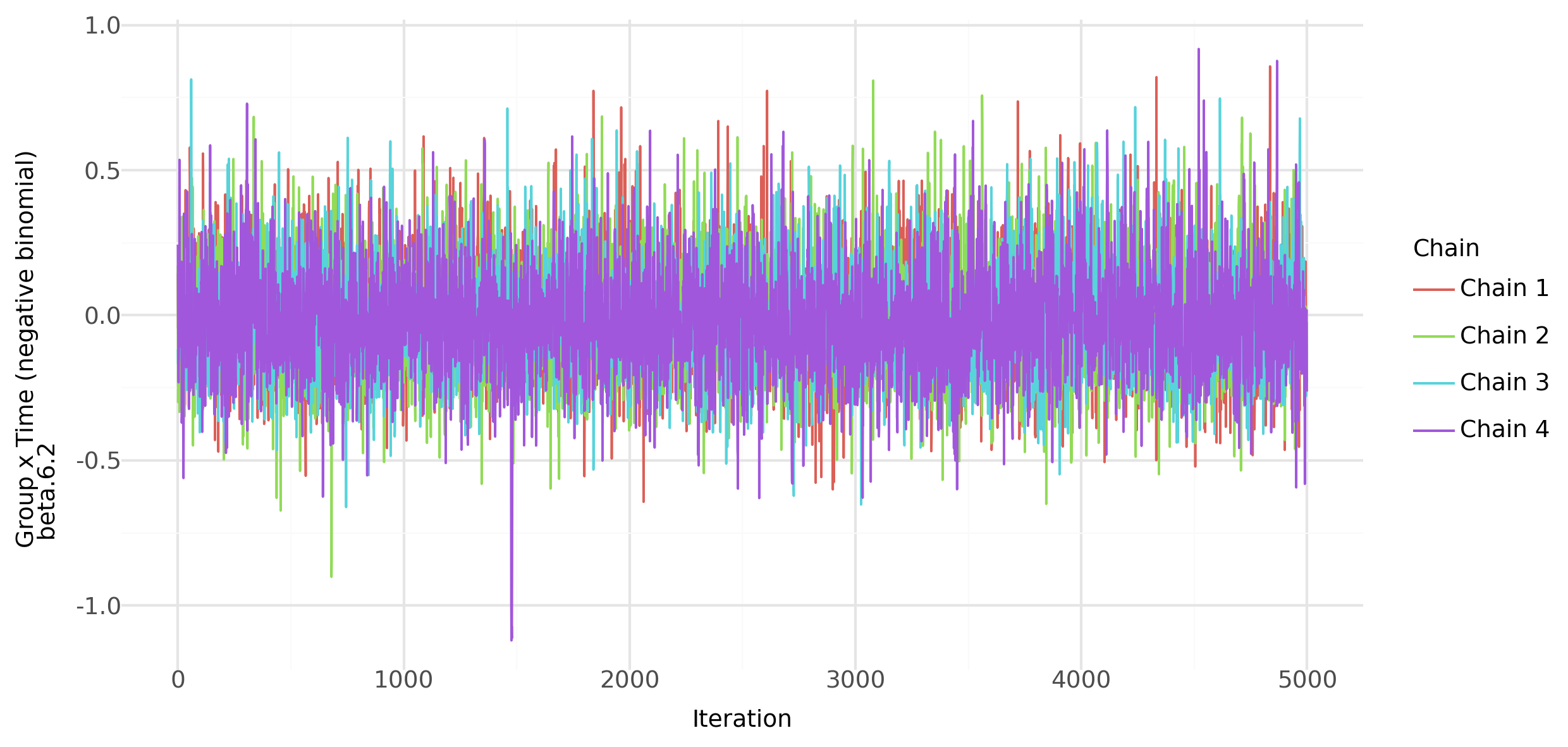


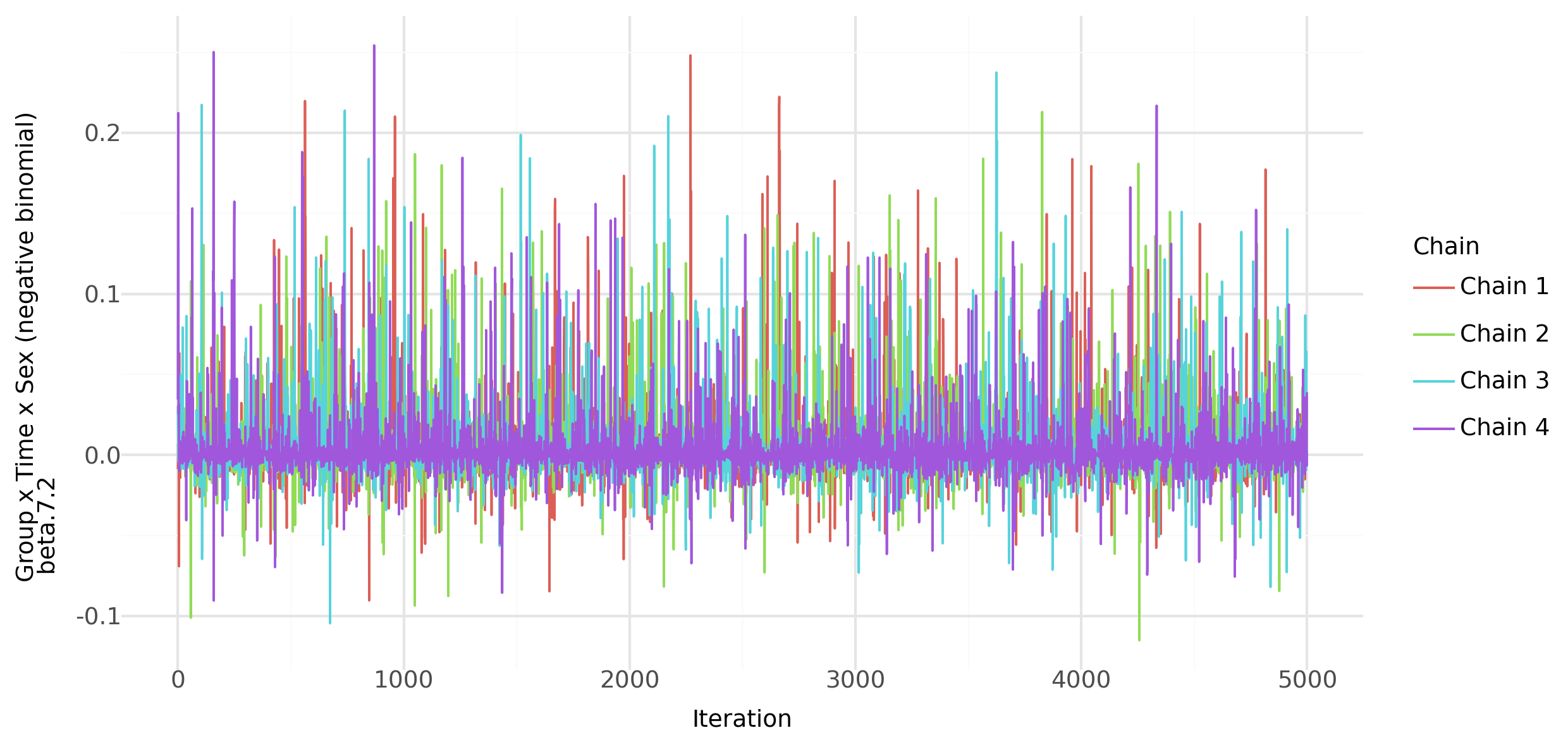

Supplement: Individualised_effects_Appendix_C_agae049 [file individualised_effects_appendix_c_agae049.docx]
