# Supplementary material for: Individualized treatment effects of a digital alcohol intervention and their associations with participant characteristics and engagement
Source: Alcohol Alcohol. 2024 Jul 21;59(5):agae049. doi: 10.1093/alcalc/agae049 (PMC11260484; doi:10.1093/alcalc/agae049)
Supplement: Individualised_effects_Appendix_D_agae049 [file individualised_effects_appendix_d_agae049.docx]

# Appendix d – Sensitivity analyses

To see if our choice of Student’s t priors in the main analyses affected estimated individualised effects, we re-estimated the multilevel zero-inflated negative binomial regression models using standard normal priors (mean = 0, standard deviation = 1) and Cauchy priors (location = 0 and scale = 1). As seen in Table 1, findings were no different between choices of priors. Figure 1 and Figure 2 illustrate the individualised effects under standard normal and Cauchy-priors.

|  | **2-months** | **4-months** |
| --- | --- | --- |
|  | Mean (standard deviation) | Mean (standard deviation) |
| **Total weekly consumption (standard drinks per week)** | | |
| Student’s t priors (primary) | -0.6 (1.4) | -1.7 (2.2) |
| Standard normal priors | -0.6 (1.4) | -1.7 (2.2) |
| Cauchy priors | -0.6 (1.4) | -1.7 (2.2) |
| **Heavy episodic drinking (episodes per month)** | | |
| Student’s t priors (primary) | -0.7 (1.2) | -1.3 (1.9) |
| Standard normal priors | -0.7 (1.2) | -1.3 (1.9) |
| Heavy episodic drinking | -0.7 (1.2) | -1.3 (1.9) |

|  |  |
| --- | --- |
| (a) Total weekly consumption | (b) Heavy episodic drinking |
| Figure 1 - Distribution of individualised treatment effects estimated using standard normal priors on (a) total weekly consumption and (b) heavy episodic drinking. | |
|  |  |
| (a) Total weekly consumption | (b) Heavy episodic drinking |
| Figure 2 - Distribution of individualised treatment effects estimated using Cauchy priors on (a) total weekly consumption and (b) heavy episodic drinking. | |
