# Supplementary material for: Individualized treatment effects of a digital alcohol intervention and their associations with participant characteristics and engagement
Source: Alcohol Alcohol. 2024 Jul 21;59(5):agae049. doi: 10.1093/alcalc/agae049 (PMC11260484; doi:10.1093/alcalc/agae049)

# Appendices e to G

## Appendix E - Associations between baseline characteristics and individualised treatment effects on total weekly alcohol consumption.

Figure 1 – Age and weekly consumption


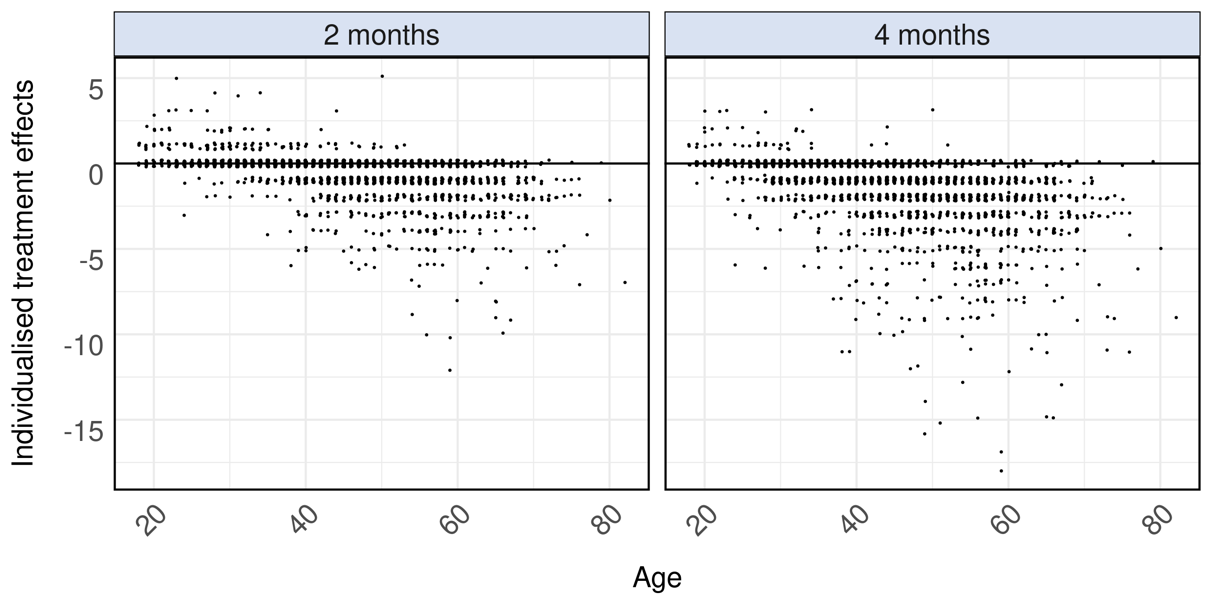


Figure 2- Total baseline consumption and weekly consumption


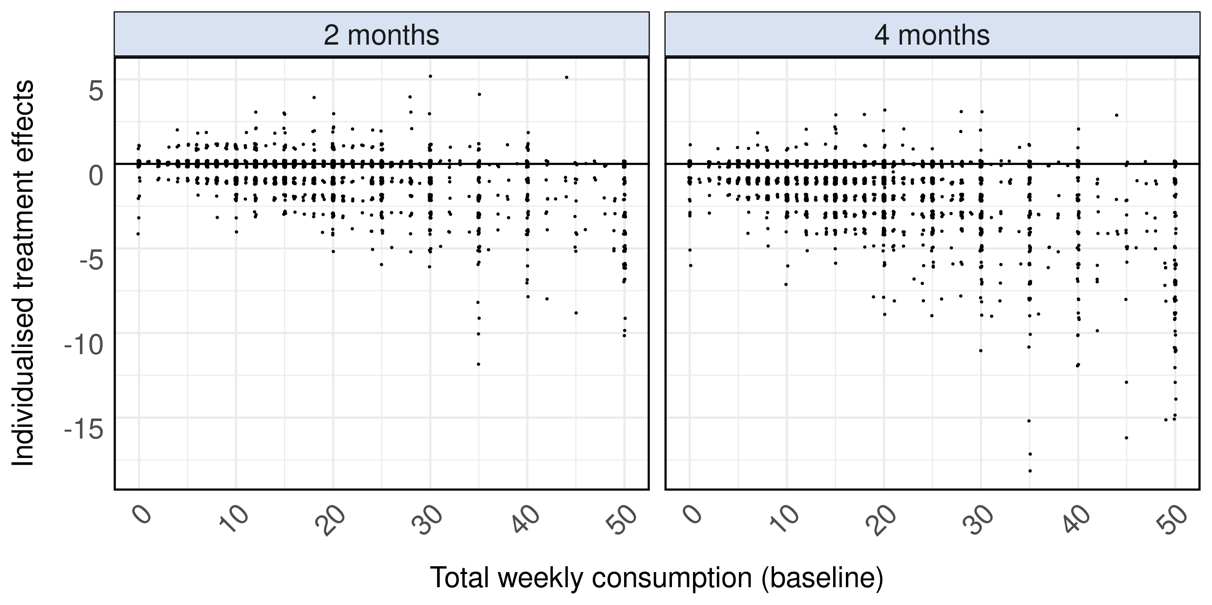


Figure 3 – Baseline HED and weekly consumption


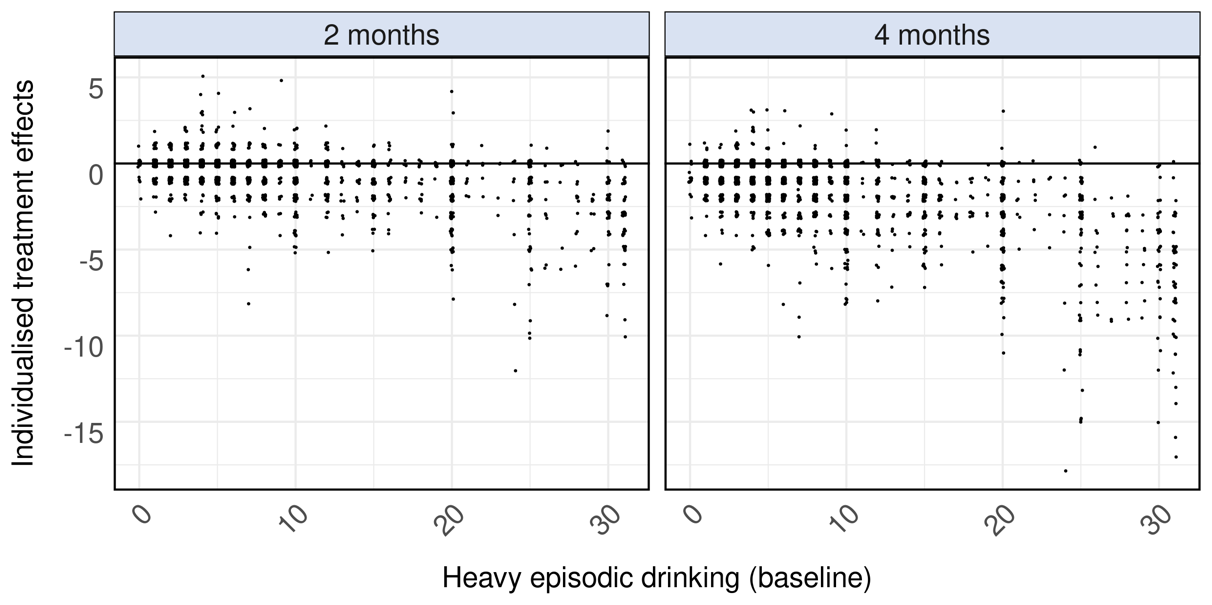


Figure 4 – Confidence and weekly consumption


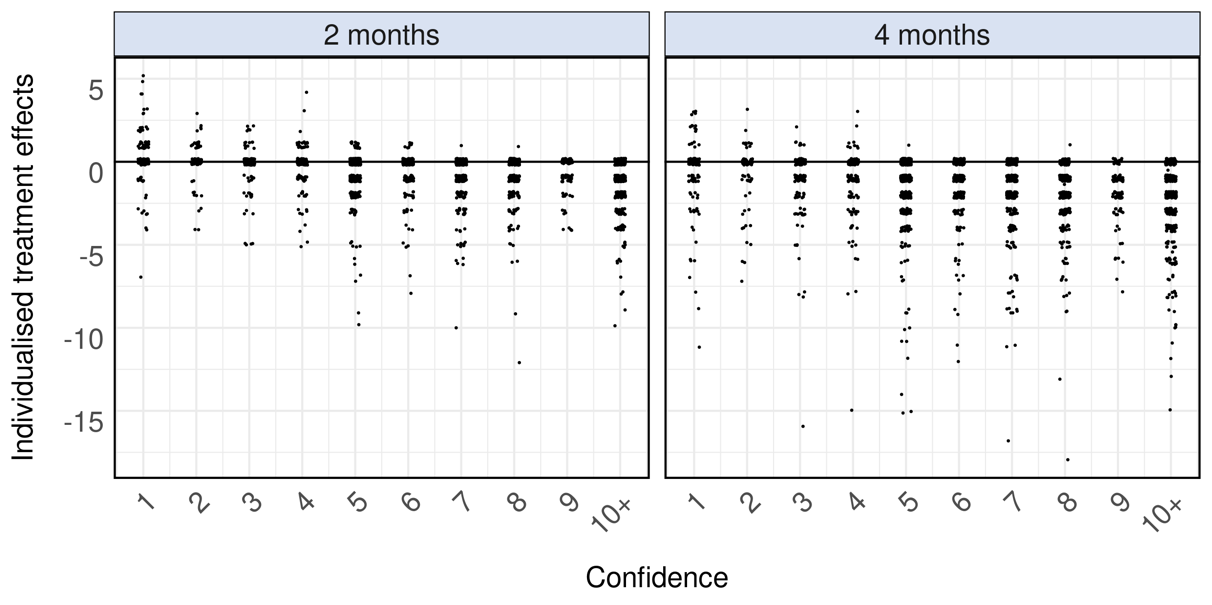


Figure 5 - Importance and weekly consumption


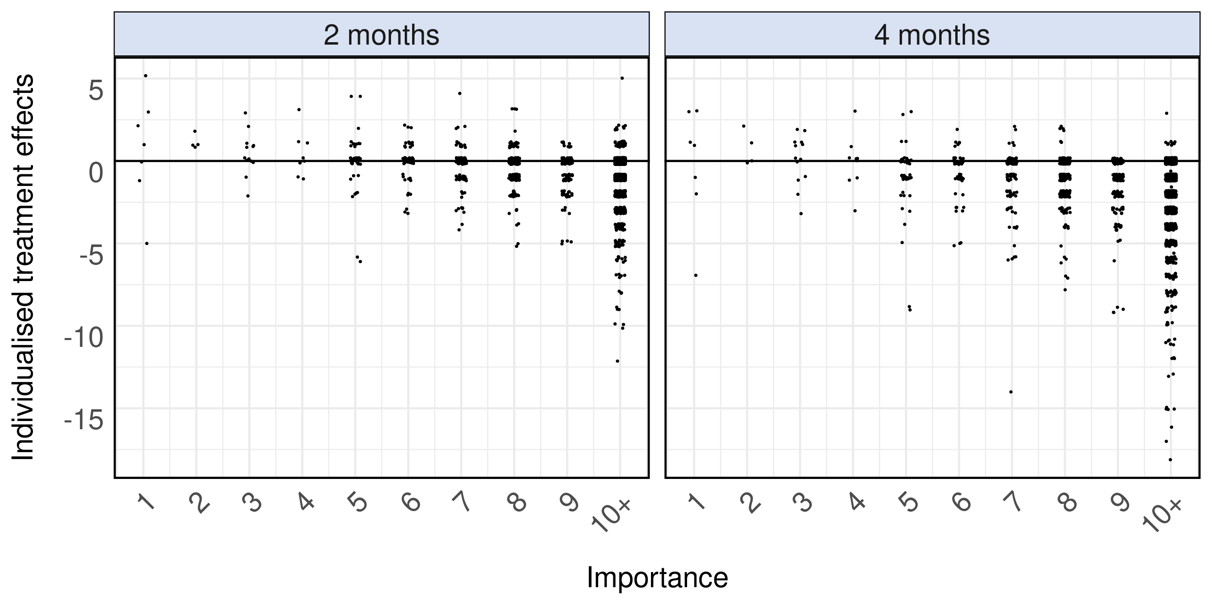


## Appendix F - Associations between baseline characteristics and individualised treatment effects on heavy episodic drinking.

Figure 1 – Sex and HED


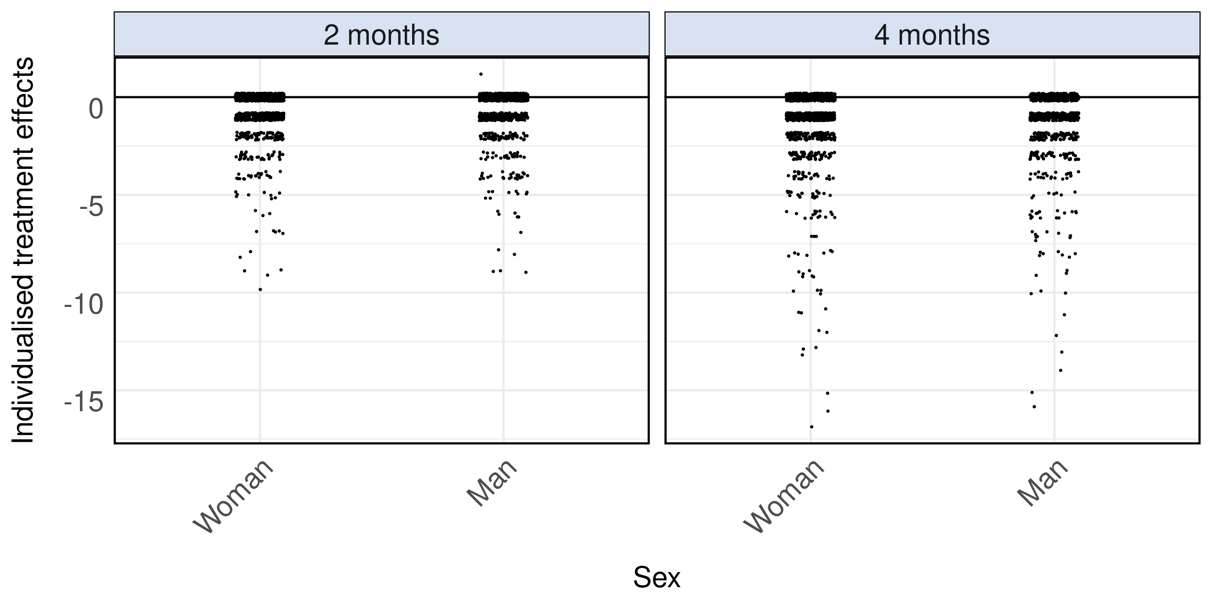


Figure 2 – Age and HED


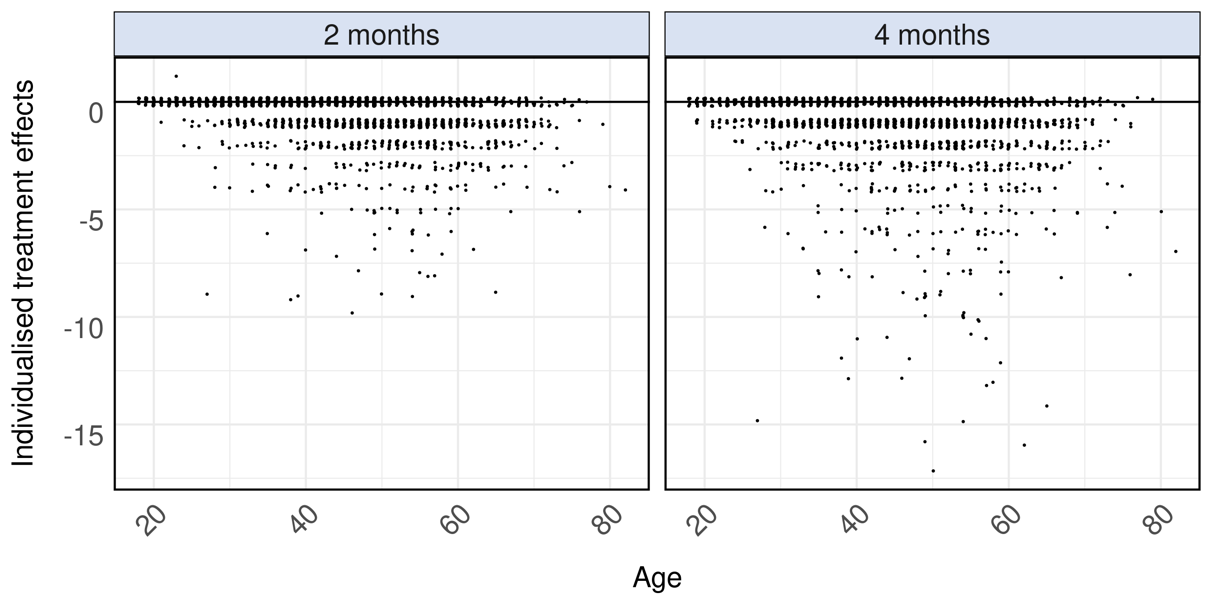


Figure 3 – Baseline total consumption and HED


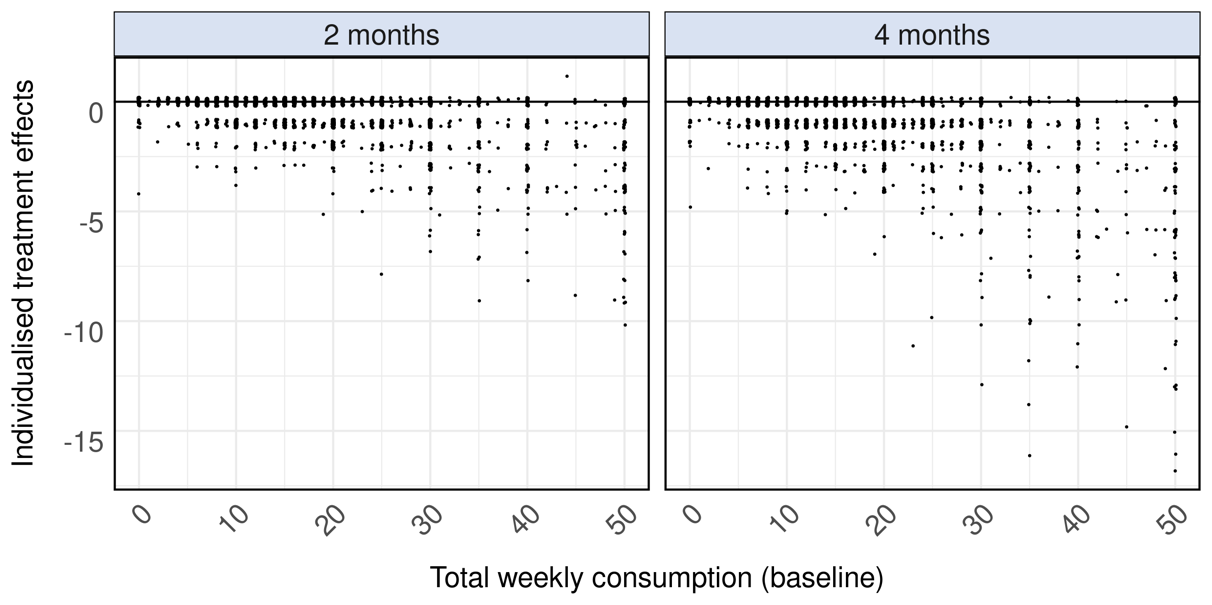


Figure 4 – Baseline HED and HED


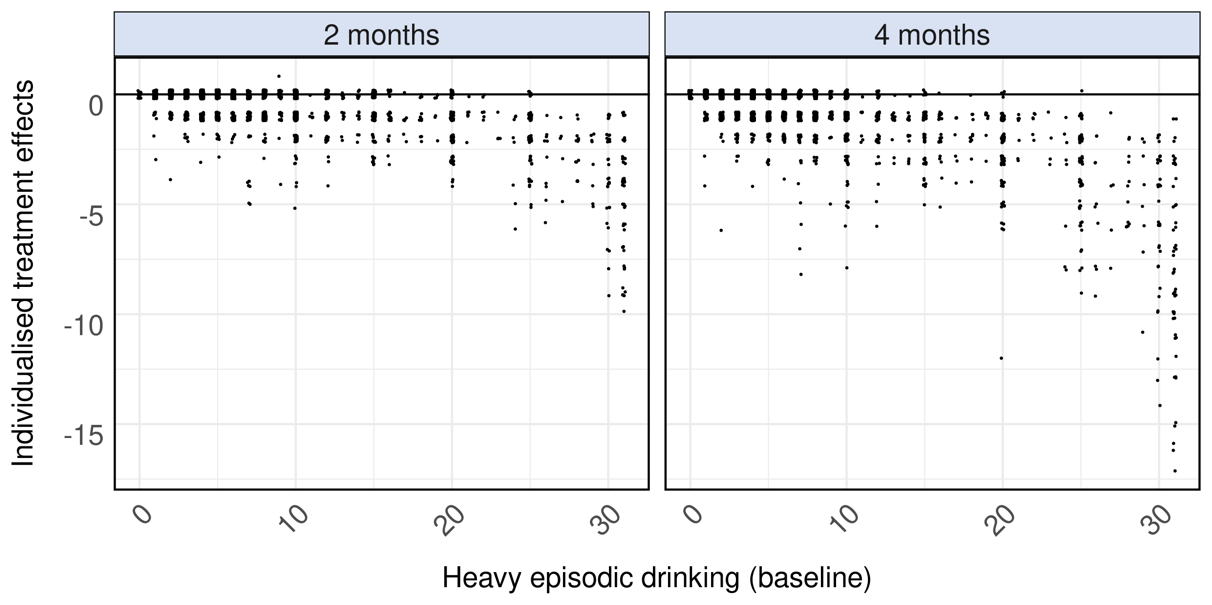


## Appendix G – Association between Engagement and Weekly Consumption

Figure 1 – Goal setting and week consumption


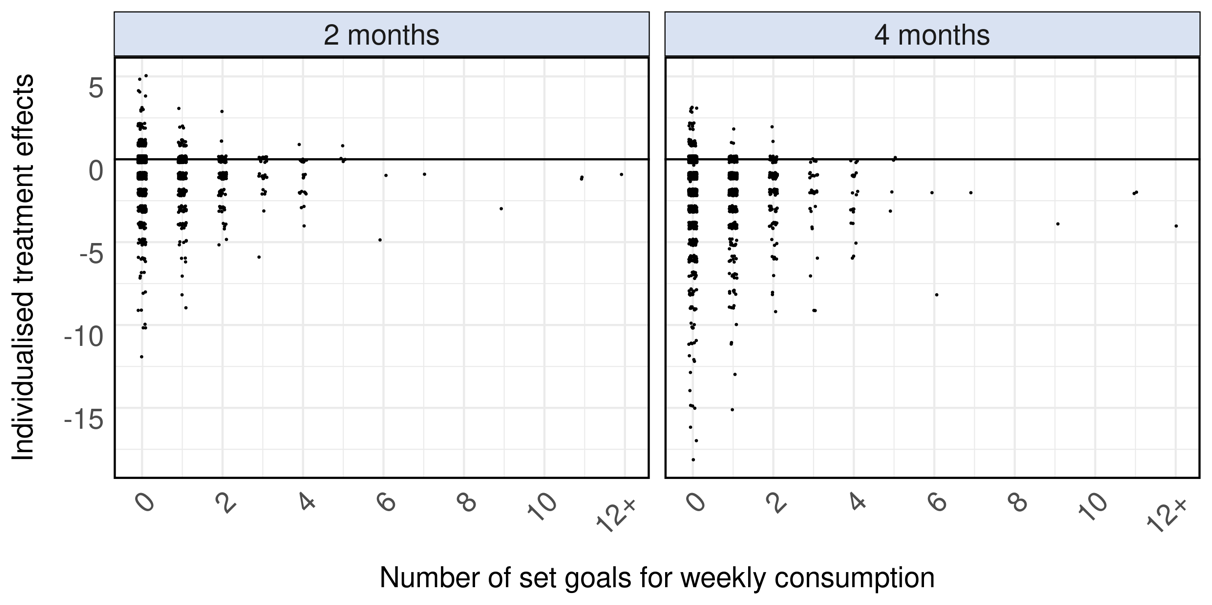


Figure 2 – Weekly screening and total weekly consumption


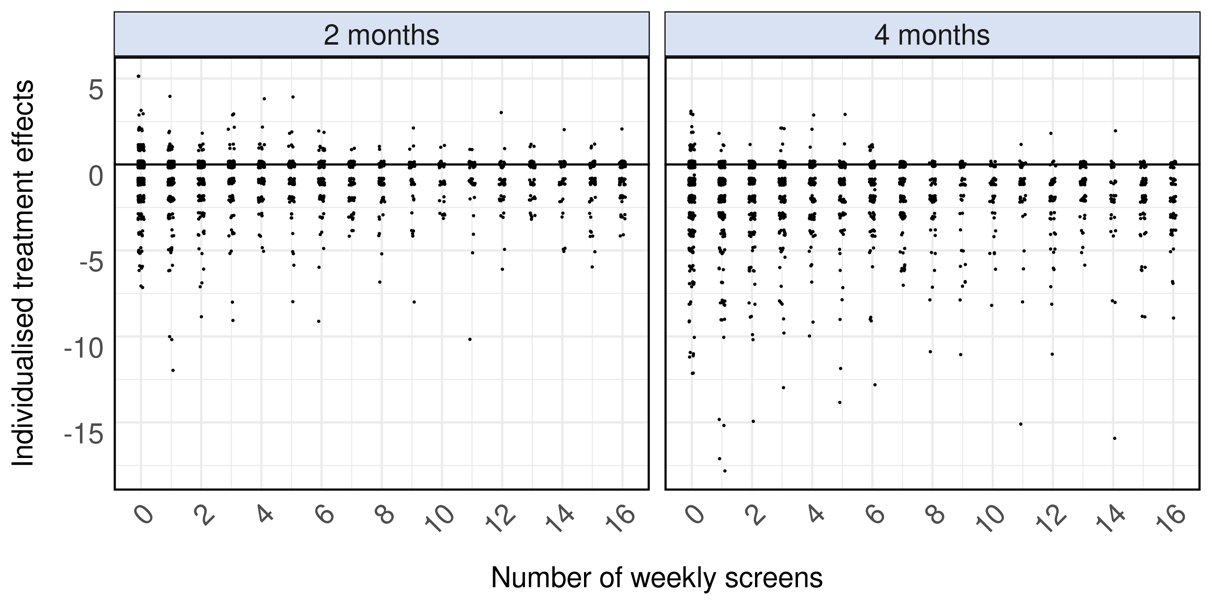


Figure 3 – Risk module and weekly consumption


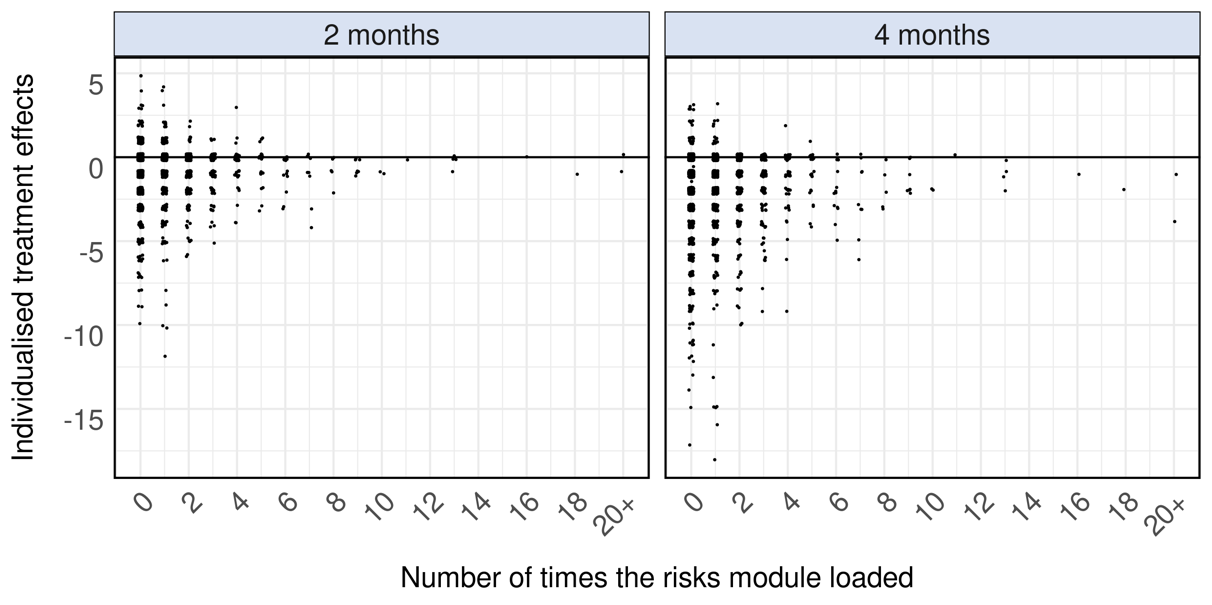


## Appendix H – Association between Engagement and Heavy Episodic Drinking

Figure 1 – Weekly screening and HED


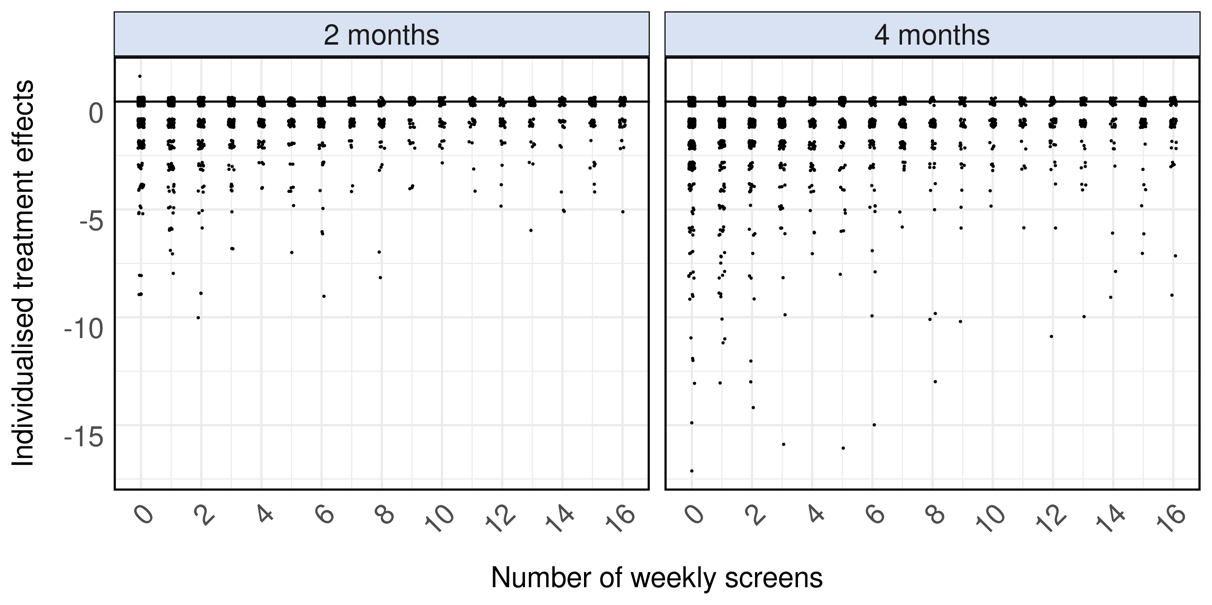


Figure 2 – Risk module and HED


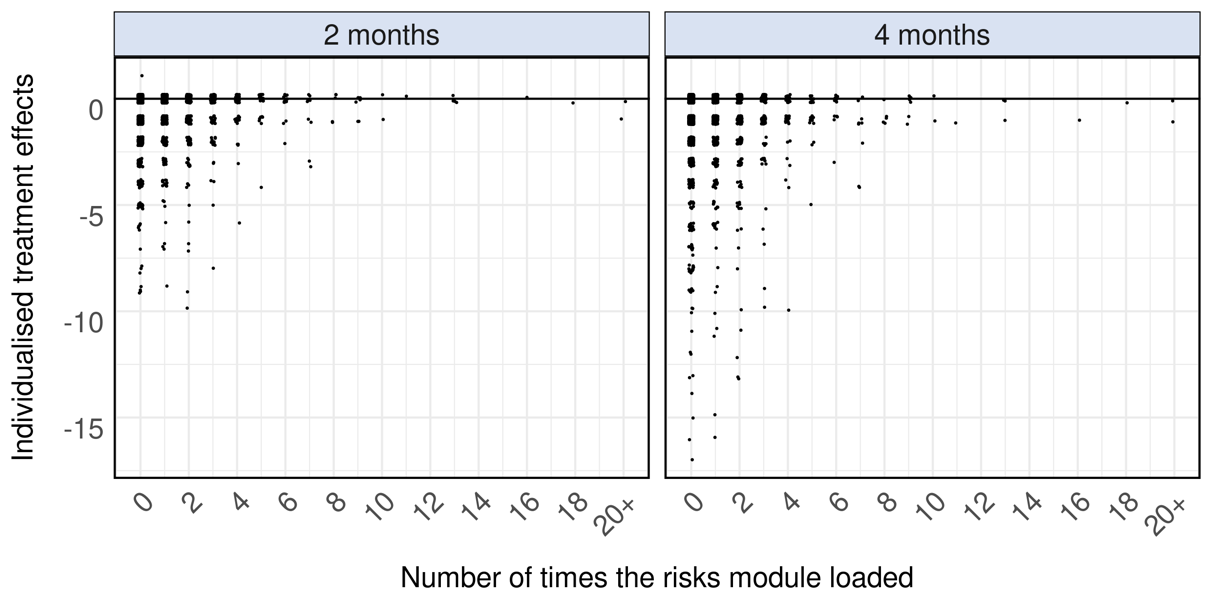

Supplement: Individualised_effects_Appendices_E_to_H_agae049 [file individualised_effects_appendices_e_to_h_agae049.docx]
